# Clarifying Problems in the Synthetic Pathway Towards Methylene‐Type Levodopa Acylals as Potential Prodrugs

**DOI:** 10.1002/ardp.70376

**Published:** 2026-10-26

**Authors:** Tobias Keydel, Yann Theele, Andreas Link

**Affiliations:** ^1^ University Medical Center Rostock Rostock Germany; ^2^ Pharma Center Bonn, Pharmaceutical Institute, Pharmaceutical & Medicinal Chemistry University of Bonn Bonn Germany; ^3^ Department of Pharmaceutical/Medicinal Chemistry Institute of Pharmacy, University of Greifswald Greifswald Germany

**Keywords:** acylals, levodopa, Parkinson's disease, prodrugs

## Abstract

Here, we report a practical synthetic pathway towards levodopa (l‐DOPA) acylals with a special emphasis on problems occurring during preparation, low yields or unmanageable side reactions, and how to circumvent them. The key step can be performed rapidly by microwave irradiation in a green chemistry manner. Final compounds can be achieved after deprotection approaches but exhibit inadequate properties in terms of stability, thwarting the adaptation of the double ester concept discussed here to the important active pharmaceutical ingredient levodopa. However, a future outlook is provided, discussing potential structural and chemical changes for stable levodopa acylals.

## Graphical abstract

Methylene‐type acylals of the well‐known active ingredient levodopa were synthesized, with each step described in detail. Problems encountered during the synthesis process and in relation to the final products were investigated. Based on these findings, an outlook for the future is provided.

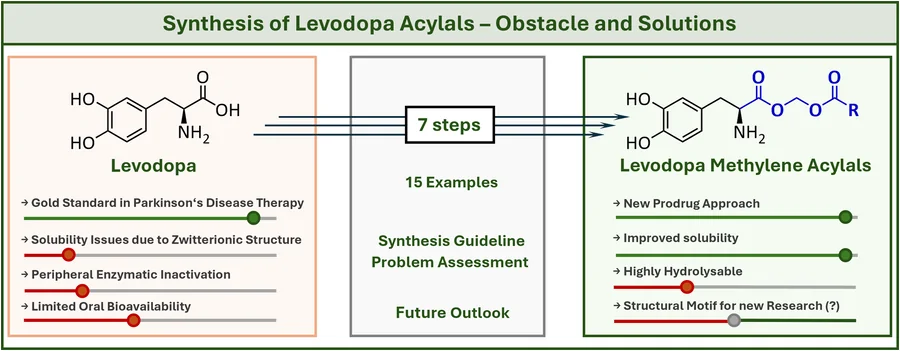

**Abbreviations**
3‐OMD: 3‐*O*‐methyldopa
Atm: atmosphere
CNS: central nervous system
COMT: catechol‐*O*‐methyl transferase
DCVC: Dry column vacuum chromatography
DDCI: dopa decarboxylase inhibitor
DIPEA: *N*,*N*‐diisopropylethylamine
eq.: equivalents
HPLC: high‐performance liquid chromatography
LC/MS: liquid chromatography/mass spectrometry
NMR: nuclear magnetic resonance
PD: Parkinson's disease
PTC: phase transfer catalysis
TEA: triethylamine
TLC: thin‐layer chromatography

## 1 Introduction

Parkinson's disease (PD) is one of the most common neurodegenerative disorders. Worldwide, the prevalence is estimated at around 300–600 affected per 100,000 people, with older people being affected much more frequently than younger people and men more frequently than women [1, 2]. Due to the general increase in life expectancy—especially in developing countries—a further increase in the number of people impaired can be expected in the future [3]. The literature estimates that the global prevalence will more than double between 2005 and 2030 [4]. Levodopa (**I**) is considered the gold standard of PD therapy due to its very high effectiveness [5]. Although being a prodrug, the therapy with levodopa comprises a multitude of pharmacokinetic problems (see review ref [6]) with the most prominent being its conversion to dopamine in the periphery leading to an increase of side effects (e.g. nausea) and a dramatic decrease of effective concentration in the central nervous system (CNS). Therefore, the therapeutic administration of levodopa must be accompanied by concomitant treatment with a dopa decarboxylase inhibitor (DDCI), e.g. benserazide or carbidopa [7] (see Figure 1). The most predominant phase II‐metabolism levodopa undergoes, is the *O*‐methylation by the enzyme catechol‐O‐methyl transferase (COMT) [8]. The resulting 3‐*O*‐methyldopa (3‐OMD) competes with levodopa for the uptake transporter leading to an additional decrease in bioavailability of residual levodopa. For this reason, inhibition of COMT represents an attractive target, with tolcapone being the first COMT‐inhibitor. Today, entacapone [9], due to reduced liver toxicity, and opicapone [10] with prolonged effect, resulting in once‐a‐day use, are the common COMT‐inhibitors, always used in combination with levodopa and a DDCI (see Figure 1). Unfortunately, this combination therapy does not prevent the on‐off phenomenon, i.e. the episodic cessation of the symptom‐relieving effect of levodopa, which occurs particularly during prolonged treatment. Even if the exact mechanism of this treatment failure is not fully understood, the erratic absorption behavior of levodopa appears to play a central role [11]. As a highly polar molecule, levodopa cannot be absorbed by passive diffusion through membranes. Instead, the amino acid transporter b0,+AT‐rBAT is responsible for absorption from the intestinal lumen [12]. However, the capacity of this transporter is limited, which, together with competition from dietary amino acids, can hinder the absorption of levodopa [13, 14]. Nagatsu and Sawada reviewed substantial aspects of levodopa‐based therapy of PD with historical insights and future perspectives [15]. They concluded that levodopa remains the gold standard, but explicitly refer to the development of neuroprotective strategies accompanied with low‐dose levodopa administration. The pharmacokinetic variabilities have led to numerous attempts to improve those properties using various prodrug strategies, where, in theory, levodopa can be derivatised in multifaceted at three obvious structural intervention sites: the carboxyl group, the amino group, and the catechol structure [16, 17] (see Figure 2).

**Figure 1:**
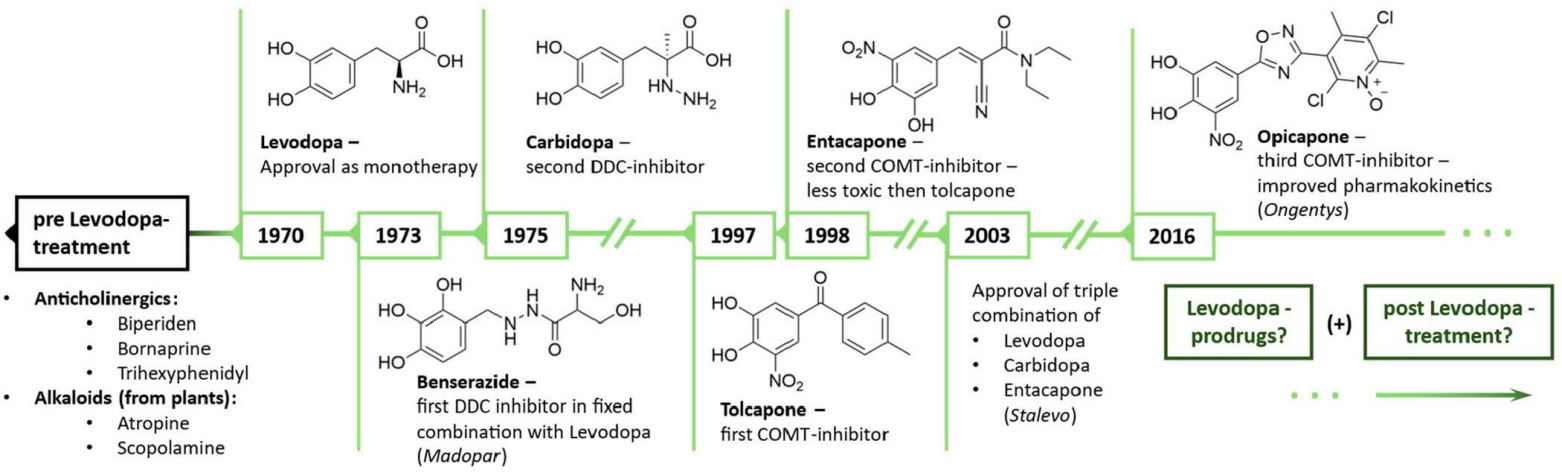
Historical timeline with key dates of levodopa treatment and further developments.

**Figure 2:**
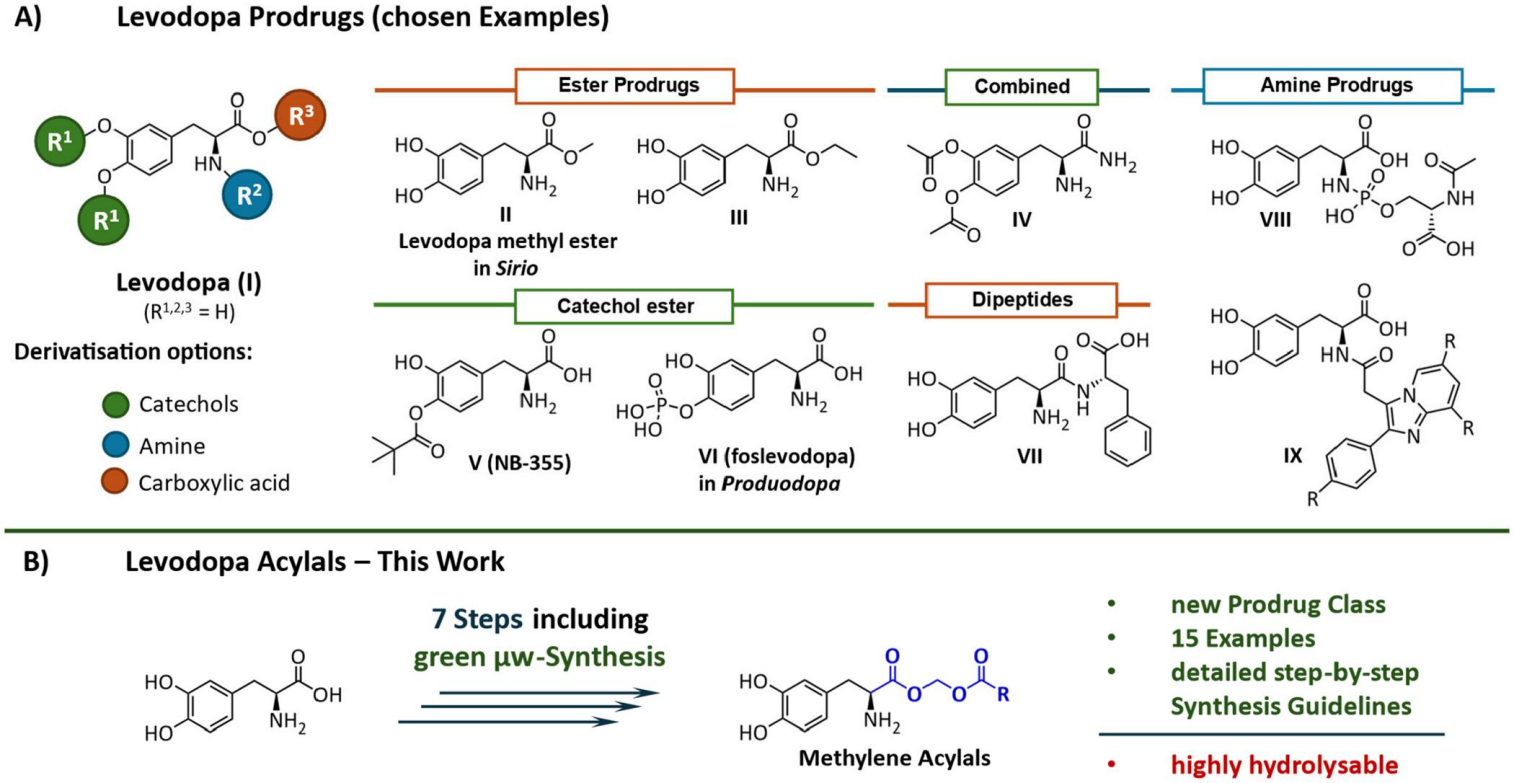
(A) Levodopa prodrugs described in the literature. (B) This work: Detailed and error‐focused description of the synthesis and evaluation of levodopa methylene acylals.

Improving lipophilicity and thus membrane permeation is a key aim in prodrug approaches and can often be realized, in case of carboxylic acid‐bearing drug molecules, by esterification [18]. In theory, these esters are capable of passive diffusion through biological membranes and are subject to rapid intracellular hydrolyzation by ubiquitous esterases, releasing the drug in its biologically active form or solutions of levodopa and carbidopa for a partial replacement therapy. By esterification of levodopa, a second advantage comes into play: the zwitterionic nature is eliminated, resulting in a dramatic increase in water solubility, a structural change applicable for the development of fast‐releasing drug forms [19]. A combination of levodopa methyl ester (Figure 2, **II**) has proven superior to a combination of levodopa and carbidopa in some aspects, such as the robustness of the pharmacokinetic profile, and is marketed under the trade name *Sirio* [20].

Next to the development of entirely new routes of administration, such as nasal application [11], esterification of the hydroxy groups of the catechol function seems to be a more promising approach. NB‐355 (**V**), the monoester of pivalic acid with levodopa showed a prolonged duration of action in animal experiments [21]. A real benefit was given by esterification of levodopa catechol with phosphoric acid, yielding foslevodopa (**VI**). Bearing drastically improved solubility and chemical stability compared to levodopa, it can be administered using continuous pump‐based infusion. With this continuous dopaminergic stimulation, a moderate reduction of daily OFF time by 43% versus 16% in the placebo group could be obtained [22]. This translates to an improving life quality, and thus advantage is provided to patients with advanced PD (see review [23]), however, the less innovative add‐on use of parental application a solution of levodopa and carbidopa combined with oral therapy reached even higher success rates in reduction of daily OFF time, namely 92% vs 31% in the placebo group. An additional advantage of the individual partial replacement complemented with an oral levodopa formulation is avoidance of the need to infuse large solution volumes of Produodopa necessary to fully substitute oral levodopa intake, while compromising skin tolerability [24].

Amides of levodopa have also been described in the literature. For instance, Zhou et al. [25] synthesized a multitude of different substances where the carboxy group of levodopa was converted to amides. In some cases, they additionally used the hydroxy groups to create esters. Especially the compound **IV** showed a dramatically increased area under the curve compared to levodopa. Peptides in which levodopa is coupled to proteinogenic amino acids via amide bonds can be regarded as a special case of amides. What is particularly interesting is that these peptides open up a new transport mechanism. In cell culture models, uptake is increased by amide formation with l‐phenylalanine (levodopa‐L‐phe, **VII**), due to transport via the peptide transporter PepT1 [26]. A possibility to use the amino group for derivatization was presented by Denora et al. [27]. They created a series of prodrugs containing a 2‐phenyl‐imidazopyridine moiety (**IX**). These molecules showed an increased permeation through the blood‐brain barrier. Olatunji et al. [28] presented multiple phosphoramidate derivates of levodopa (**VIII**), which were able to release levodopa in a controlled manner depending on pH.

A functional group that—to the best of our knowledge—has so far not been presented in the peer‐reviewed literature as part of prodrugs of levodopa are acylals. The utilization of acylals in drug design bears a certain resemblance to ester prodrugs, as they enable polar carboxyl groups to be converted into more lipophilic structural elements, which significantly improves membrane permeation. In addition, acylals are typically more sensitive to hydrolysis than simple carboxylic esters, which makes it easier to release the actual active substance from the prodrug [29].

In this work, we present a robust synthetic pathway yielding levodopa acylals. For tethering two carboxylic acids a microwave‐assisted synthesis in the sustainable solvent dihydrolevoglucosenone (*Cyrene*) was used, which has been described by our group previously [30]. A small library of structurally diverse compounds was generated. Our study primarily aims on observations and problems during every synthetic step. Insufficient purity due to excessive hydrolysability prevented in vitro testing of the substances; we therefore discuss here, in particular, the feasibility of their synthesis and highlight possibilities for future work on structurally related compounds as potential prodrugs.

## 2 Results and Discussion

The synthetic approach was initiated with the incorporation of orthogonal protecting groups into levodopa (**I**), which was principally designed to stabilize the oxidation‐prone catechol structure and to mask the amino group, as this has been demonstrated to impede subsequent synthetic steps. First, a boc group was introduced to the amino function to obtain *N*‐boc‐levodopa (**1**). In this case, triethylamine (TEA) was utilized as a base in lieu of the conventional sodium hydroxide (Figure 3, upper part).

**Figure 3:**
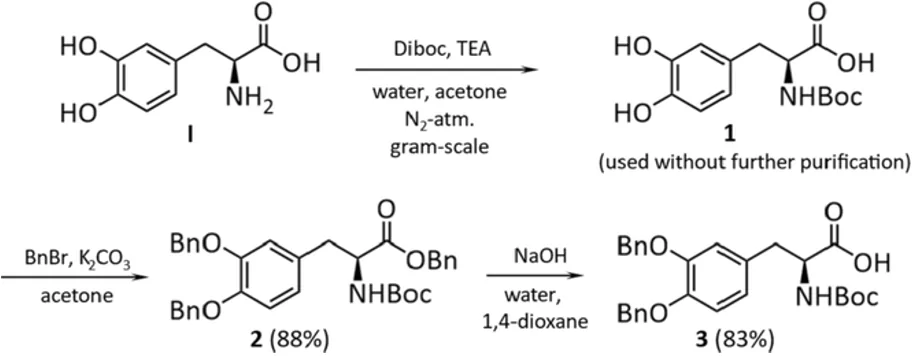
3‐Step‐synthesis towards bench‐stable catechol‐ and amine protected levodopa (**3**).

As a less nucleophilic base, TEA does not tend to deprotonate the catecholic hydroxyl groups to the same extent, which would be undesirable because it would favor oxidation [31]. The solvents utilized for this purpose (water and acetone) were degassed in advance using ultrasound, and the reaction was carried out in an argon atmosphere, which suppresses oxidation. Following a rapid acidic work‐up, the crude product was then benzylated using benzyl bromide to obtain product **2**. This one‐pot synthesis, performed on a gram scale, proceeded smoothly and generated a bench‐stable solid with very good yields (Figure 3, bottom part).

Although several papers and textbooks report the feasibility of acetonide protection of catechols, all attempts to obtain the desired compound via this route proved unsuccessful. This finding is consistent with the work of Yang et al. [32], who have documented inconsistencies in the reported procedures. The subsequent stage of the process pertains to the cleavage of the benzyl ester. This process is conducted through a conventional saponification reaction, employing sodium hydroxide as the saponifying agent. It is noteworthy that this method is not detrimental to either the boc group or the catecholic benzyl ethers. Following an acidic work‐up, the free acid **3** is converted under phase transfer catalysis (PTC) with chloromethyl chlorosulfate [33, 34] to the chloromethyl ester **4**, which was designed to serve as a precursor for the acylals (Figure 4A).

**Figure 4:**
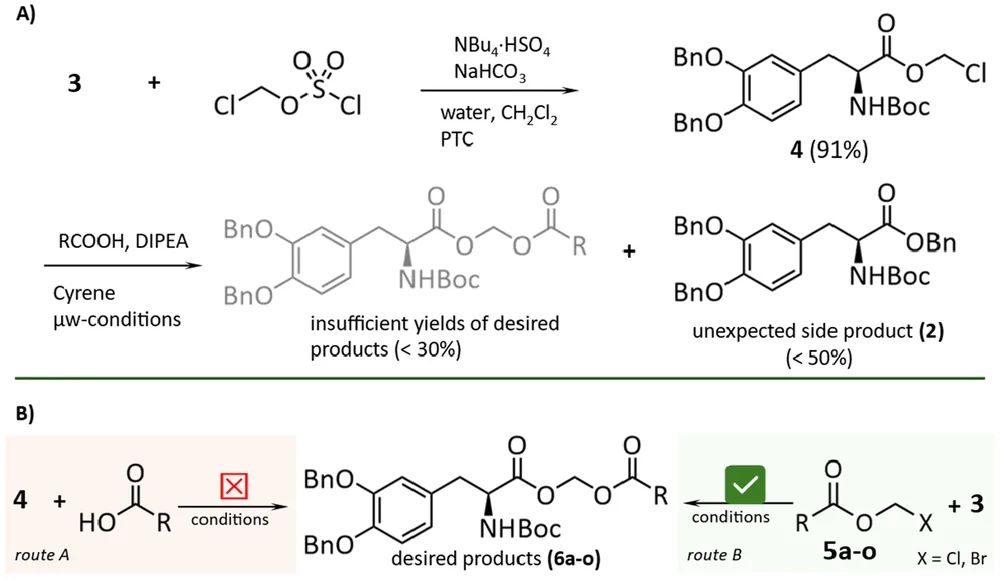
(A) Synthesis of the chloromethyl ester **4** as the desired precursor of predefined acylal synthesis and failure of its application due to unexpected occurrence of **2** as a side product. (B) Circumvention of by‐product formation with synthesis via route B.

Due to problems in the subsequent step (Figure 4A), bottom part), next to **4**, a series of chloromethyl esters were synthesized (see Figure 5) and used for acylal synthesis. The esterification of **4** with various carboxylic acids to form the corresponding acylals should follow a similar process to that which we recently published. In this method, the reaction takes place under microwave irradiation in the green solvent *Cyrene*. It was a surprising discovery that, in addition to the product and next to a mixture of unidentified side‐products, an unexpected by‐product was also obtained in high yield (> 50%). Following isolation and analytical identification by NMR and HRAMS, it was found that this by‐product was the tribenzylated *N*‐boc‐levodopa (**2**) (see Figure 4). Here, we assume an intra‐ and intermolecular rearrangement reaction, where the migration of benzyl groups from the catechol ether oxygen to substitute the chloromethyl ester forming the benzylic ester occurred. We believe that the mixture of not isolated side‐products consists of mono‐, di‐ and non‐benzylated *N*‐boc‐levodopa formerly acting as benzyl group donators. This unwanted side‐reaction prevented a satisfactory yield of the acylals following route A (Figure 4B), so an attempt was made to synthesize the acylals from the chloro‐ and bromomethyl esters of the carboxylic acids with compound 3 (see route B in Figure 4B).

**Figure 5:**
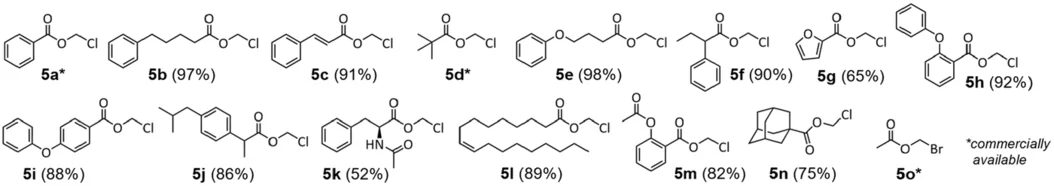
Scope of chloro‐ and bromomethyl esters. While the compounds **5a**, **5d** and **5o** were commercially available, the other chloromethyl esters were prepared similarly, as shown in Figure 4A).

To our delight, no tribenzylated by‐product could be identified, and the reaction proceeded quickly and efficiently (Figure 4B). However, some products (**6a**, **6e**, **6g**, **6k**, see Figure 6) were obtained in unexpectedly low yields. During purification, which was carried out using dry column vacuum chromatography, a solvent‐sparing, fast and efficient method [35], some fractions revealed a gel‐like substance. We assume that this observation is based on a special chemical behavior of levodopa, leading to lower yields in some cases. A cross‐linking of levodopa leads to the formation of polymers, which can be seen in nature as a part of a “protein glue” used by mussels to stick to rocks underwater [36]. This phenomenon led to its own research area for its usage in biomedical applications [37, 38].

**Figure 6:**
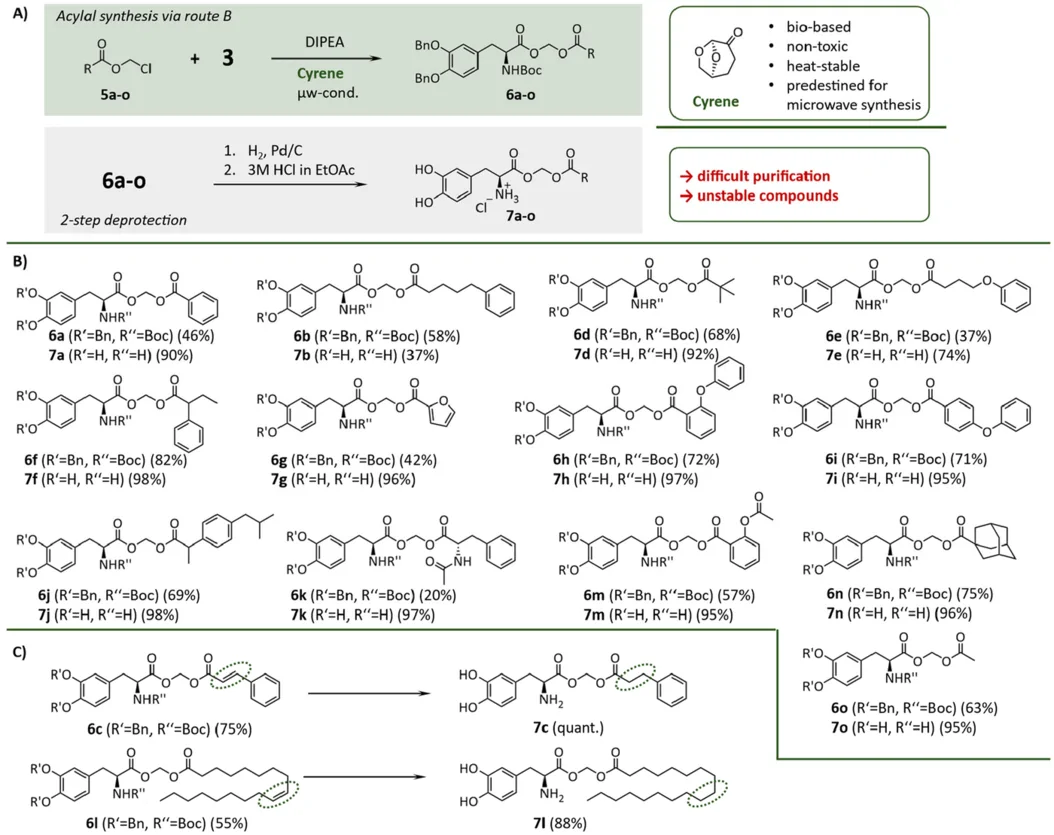
(A) Synthetic pathway and (B) Structures of protected levodopa acylals (**6a**‐**o**) and corresponding deprotected compounds (**7a**‐**o**). (C) Limit of the synthetic procedure in terms of predicted hydrogenation of alkenes (**6c**, **6l**) during benzyl cleavage.

The final and most critical step in this process is the removal of the protecting groups to obtain the finished output stages. In a manner analogous to the protection procedure, the protective groups are to be removed in a two‐step process, eschewing a time‐consuming intermediate cleaning step. Initially, the catechol‐protecting benzyl groups are cleaved off hydrogenolytically under palladium catalysis (Figure 6A). The reaction is carried out in an argon atmosphere, with methanol as solvent, which was degassed beforehand. Subsequent to hydrogenolysis, the reaction mixture is expeditiously filtered through a thin layer of diatomaceous earth to remove the catalyst, and then concentrated in a partial vacuum. The residue is then subjected to immediate mixing with a freshly prepared 3 molar HCl in ethyl acetate (the solution is prepared from a mixture of methanol and acetyl chloride in ethyl acetate). Following the completion of boc deprotection (monitored by thin‐layer chromatography (TLC)), the liquid components are subjected to rotary evaporation, resulting in the production of the desired products (Figure 6). Standard instrumental analytics of final compounds revealed insufficient purity, where no substance exceeded 95% purity as is demanded in medicinal chemistry. Unfortunately, swapping of the order of deprotection reactions as well as utilization of commercially available 4 molar HCl in dioxane (common boc deprotection reagent) does not improve the outcome. Column chromatography was no suitable option either, as no increase in purity was detected. Even preparative HPLC could not enable isolation of pure compounds due to degradation of the compounds in aqueous solution. During any purification approach, a certain amount of levodopa acylal gets hydrolyzed, which was demonstrated by the detection of free levodopa and the corresponding auxiliary acid by TLC and HPLC.

The synthetic pathway explored during our study is suitable for the synthesis of a fully protected levodopa derivative, which further can easily be converted into protected methylene acylals using chloromethyl esters of various carboxylic acids as coupling partners. These reactions went smoothly without the need of harsh conditions obtaining acylals with satisfactory to very good yields. The key coupling step was successfully performed under green chemistry conditions.

Problems occurred during the last step aiming to remove the protecting groups. While the boc‐deprotection is usually well tolerated by either acylal‐ and catechol structure as well as the auxiliary acid motifs, hydrogenolytical abstraction of benzyl ethers will reduce, as expected, double bonds of other parts of the molecule, if present. In cases of compounds **6c** and **6 l**, the utilized cinnamic acid and oleic acid as coupling partners were converted into corresponding saturated products (**7c**, **7 l**). At this point, future approaches should aim for other deprotection methods or utilization of catechol protection groups, which can be cleaved under different conditions. While the specific synthetic steps went as expected, the isolated compounds showed unsatisfactory purity. To our upset, this incident couldn't be healed with any purification method. This is attributable to the substances' high tendency to hydrolyze and, sometimes in parallel event, due to oxidation processes.

The high instability convinced us to perform an experiment to evaluate the possible degradation products of levodopa acylals. Therefore, freshly synthesized and isolated compound **7a** was dissolved in water, and the solution was evaporated at 80°C in a dry oven subsequently. The residue was analyzed by LC/MS and NMR. MS analytics revealed a compound with an *m*/*z* of 209 which corresponds to the mass of levodopa plus an additional methylene group (‐CH_2_‐). ^1^H NMR shows a newly generated signal at 4.1 ppm which can be referred to the methylene group bridging the nitrogen atom to the benzene core of the possibly formed tetrahydroisoquinoline (*m*/*z* = 209) (see Figure 7, the results are roughly equivalent to the work of Chelopo et al., who reported the NMR spectra of the tetrahydroisoquinoline in D_2_O [39]). Despite not mimicking physiological conditions the formation of a potentially toxic tetrahydroisoquinoline exhibits a chemical liability which might justify further investigation under physiologically relevant conditions. If observed, the formation of such potentially toxic compounds marks a strict constraint for levodopa methylene acylals, especially due to a possible contribution of tetrahydroisoquinolines to PD [40].

**Figure 7:**
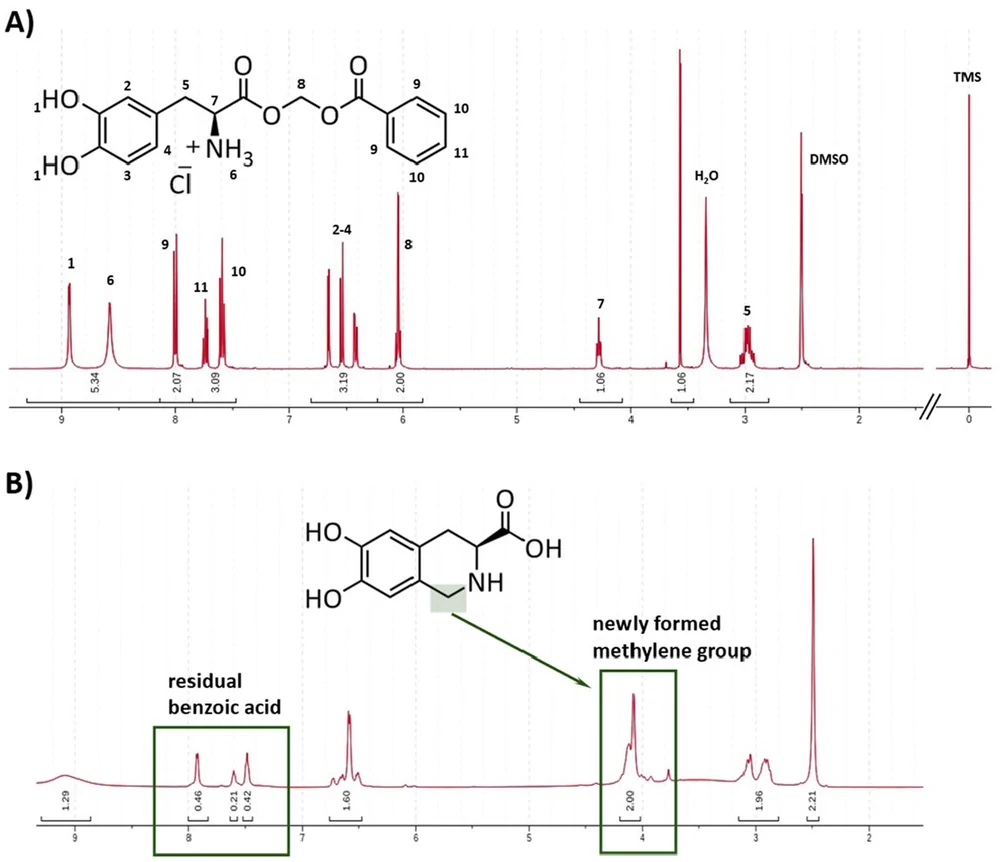
^1^H NMR spectra of freshly isolated **7a** in comparison with spectra of an artificially aged, decomposed sample (B). The signal at 4.1 ppm belongs to the newly incorporated methylene group, presumably of the resulting tetrahydroisoquinoline.

Due to their high risk of hydrolyzation, occurring even at normal humidity levels, and oxidation of the catechol to the corresponding 1,2‐benzoquinone, levodopa methylene acylals seem unsuitable for further biological evaluation. Nevertheless, the idea of double ester prodrugs of levodopa remains an important basis for further research. Structural changes could include possible biocompatible protecting groups, especially for the catechol structure to suppress oxidation while synthesis and in vitro testing.

Utilization of sterically demanding auxiliary acids, e.g. pivalic acid, do not decrease the hydrolyzation rate of the acylal structure. A promising prodrug candidate could be built on higher substituted acylals, meaning additional alkyl‐ or aryl substituents at the linking carbon atom—a strategy found in well‐known cefuroxime axetil. Acylal related structure are also a promising strategy: [(Alkoxycarbonyl)oxy]alkyl esters, as found in candesartan cilexetil [41] or [(alkoxycarbamoyl)oxy]alkyl esters (gabapentin encarbil [42]) have been clinically tested and, in certain cases, have demonstrated significant benefits. Combinations of different prodrug strategies are also conceivable, such as acylals and related structures with esters of the catechol structure. Coupling of levodopa to specific recognition motifs of transport proteins might lead to molecules acting as substrates of this specific protein, especially in case of promiscuous proteins like the human peptide transporter hPepT1 [43, 44].

In Figure 8, examples of such structures are given. *CAVE*: During levodopa prodrug research, the possible generation of tetrahydroisoquinolines should be part of degradation product analysis. However, it is conceivable that derivatization of the amino group would prevent the degradation reaction described in this study and, consequently, the formation of the tetrahydroisoquinoline.

**Figure 8:**
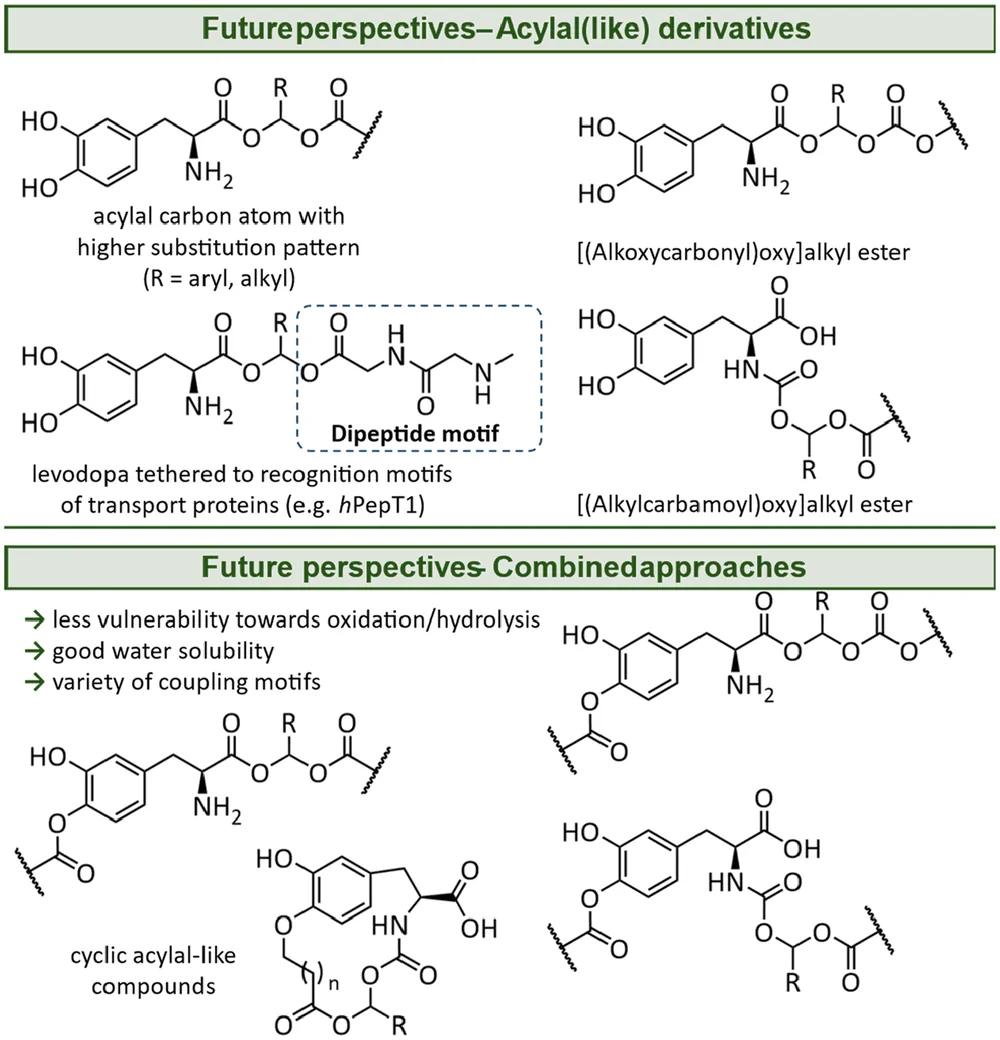
Possible prodrugs of levodopa for future efforts.

## 3 Conclusion

In conclusion, our study defines both the synthetic feasibility and the limitations of the applicability of the established double ester prodrug concept to the important example levodopa. We prepared 15 levodopa acylals in seven steps. Every step was extensively evaluated and occurring problems were discussed and possible circumventions were given. With two synthetic parts being realized in one‐pot synthesis, the purification efforts have been minimized. The acylal synthesis, representing the key step of the approach, was performed in a fast microwave synthesis procedure supported by the green solvent dihydrolevoglucosenone (*Cyrene*). Deprotection reactions were carried out as known in the literature, obtaining new levodopa derivatives. Unfortunately, two major concerns must be noted here: the final compounds exhibit insufficient purity due to their high tendency of hydrolysis, and there is some evidence that levodopa acylals may be converted into toxic tetrahydroisoquinolines in vivo. For this reason, future research must aim for structural differentiation of the acylal motif and, for instance, in combination with biocompatible protecting groups for oxidation sensible catechol structure and/or the amino group, avoiding possible tetrahydroisoquinoline formation.

## Conflicts of Interest

The authors declare no conflicts of interest.

## Supporting information


Supporting File 1

Supporting File 2

## Data Availability

The data that support the findings of this study are available in the supporting material of this article.
