# Supplementary material for: Clarifying Problems in the Synthetic Pathway Towards Methylene‐Type Levodopa Acylals as Potential Prodrugs
Source: Arch Pharm (Weinheim). 2026 Oct 26;359(10):e70376. doi: 10.1002/ardp.70376 (PMC13699375; doi:10.1002/ardp.70376)
Supplement: Supplementary file 1 [file ARDP-359-e70376-s001.pdf]

Clarifying Problems in the Synthetic Pathway towards Methylene-Type Levodopa Acylals as Potential Prodrugs

Tobias Keydel[a], **Yann Theele**[b], and **Andreas Link**[c]

[a] University Medical Center Rostock
[b] University of Bonn
[c] University of Greifswald

Supporting Information

Contents

1 General Information **2**
1.1 Melting Point Determination . . . 2
1.2 Thin-Layer Chromatography (TLC) . . . 2
1.3 Dry Column Vacuum Chromatography . . . 2
1.4 Flash Chromatography . . . 3
1.5 Microwave-assisted Synthesis . . . 3
1.6 NMR Spectroscopy . . . 3
1.7 Mass Spectrometry . . . 3

2 Synthetic Procedures **4**
2.1 General Synthesis Procedures . . . 4

3 Description and Analytical Characterisation of the Compounds **5**
3.1 Protected Levodopa Building Blocks . . . 5
3.2 Chloromethyl Esters . . . 6
3.3 Protected Acylals . . . 10
3.4 Deproteced Acylals . . . 16

4 ^{1}H and ^{13}C NMR spectra **23**

1 General Information

Unless otherwise specified, all chemicals were used as supplied by the manufacturer. Whenever the text refers to “water”, fully deionized water was used.

1.1 Melting Point Determination

For melting point determination, the Büchi M-565 melting point apparatus was used. The melting point can be determined either manually or automatically. The temperature range extends from +10 °C above room temperature to 400 °C. Manual determination is performed using a precision magnifying glass with 2.5x magnification; automatic determination is performed using a video camera with video recording and 6x magnification. The melting point is determined with a measurement accuracy of ±0.3 °C up to 250 °C and of ± 0.3 °C to ± 0.5 °C from 250 °C to 400 °C, at a heating rate of 0.5 °C/min. The reproducibility of the melting point at a heating rate of 0.5 °C/min is ± 0.1 °C. All analyses were prepared using the M-569 sample compactor. For all substances, the approximate melting range was first determined by a rapid measurement (5 °C/min) and then measured starting 10 °C below the lowest melting temperature at a rate of 0.5 °C/min.

1.2 Thin-Layer Chromatography (TLC)

All analyses were performed on Merck silica gel TLC plates (silica gel on aluminum foil, layer thickness 165-235 µm, pore size 60 Å, particle size 10–12 µm). All R_F values were determined on the spot center. Eluents used (all values *V*/*V*):

- Eluent I: hexane/ethyl acetate (7:3)
- Eluent II: hexane/ethyl acetate (1:1)
- Eluent III: hexane/ethyl acetate (8:2)
- Eluent IV: hexane/ethyl acetate/acetic acid (6:3:1)
- Eluent V: hexane/ethyl acetate (9:1)
- Eluent VI: hexane/ethyl acetate (6:4)

Detection following completion of TLC was performed under UV light (λ = 254 and 366 nm). In case of insufficient quenching of UV-light, the following spray reagent was used:

- Potassium permanganate reagent: 1.5 g of potassium permanganate, 10 g of potassium carbonate, and 0.1 g of sodium hydroxide were dissolved in 200 mL of water.

1.3 Dry Column Vacuum Chromatography

The crude products to be purified were loaded onto diatomaceous earth using dichloromethane and purified using a previously hand-packed silica gel column (Silica Gel 60 from Carl Roth GmbH + Co. KG, Karlsruhe, particle size 0.02-0.045 mm, pore size 53–80 Å) with a length of approximately 6 cm and a diameter of 2.7 cm.

1.4 Flash Chromatography

The puriFlashRXS 520 Plus from Interchim was used. The columns employed were—as recommended by the instrument software—the SI-HP columns F0040, F0080, and F0160 (each with a particle size of 30 µm). The crude products to be purified were eluted onto silica gel using dichloromethane and applied to the separation columns as a dry load. For each flash chromatography run conducted as part of this work, a hexane-ethyl acetate gradient automatically generated by the instrument software was used as the mobile phase.

1.5 Microwave-assisted Synthesis

The Monowave 400 microwave synthesis reactor from Anton Paar was used. All syntheses were carried out using the closed-vessel method in G10 vials (max. 6 mL). Temperature was controlled using an IR sensor at the bottom of the reaction chamber. Pressure was controlled using a sensor located above the septum of the reaction vessel. All syntheses were carried out under stirring (800 rpm). The maximum values for temperature (θ_{max}), working pressure ($_{pmax}$), and power (P_{max}) were set using the reactor's internal software (version 3.20). Prior to the actual reaction time, there is a heating phase required to reach the target temperature. All syntheses were carried out without continuous compressed air countercooling.

1.6 NMR Spectroscopy

An Avance III with an Ultrashield 400 (^{1}H: 400.2 MHz, ^{13}C: 100.6 MHz) from Bruker Biospin was used. Software: TopSpin (Version 2.1) from Bruker Biospin. The measurement temperature was always 25 °C. Information on the coupling constant J applies to H–H couplings unless otherwise noted (generally: XJY,Z). All chemical shifts are given in ppm (δ scale) and are relative to tetramethylsilane (TMS, δ: 0 ppm). The solvents used are specified in the respective spectral data. The assignment of signals in all ^{13}C NMR spectra was verified using a DEPT135 experiment. The assignment of signals in all ^{1}H and ^{13}C NMR spectra was verified using HSQC and HMBC experiments.

1.7 Mass Spectrometry

A Bruker Elute UHPLC with Bruker Compact QTOF-MS (Bruker Daltonics, Bremen, Germany), operated with ESI or APCI was used to measure HRAM-MS data. Methanol and water for HPLC-MS/MS were obtained from Fisher Scientific.

2 Synthetic Procedures

2.1 General Synthesis Procedures

General Synthetic Procedure A: Synthesis of Chloromethyl Esters

An amount of 5 mmol (1.0 eq.) of the corresponding carboxylic acid were reacted with 1.680 g (20.0 mmol, 4.0 eq.) sodium bicarbonate and 0.170 g (0.5 mmol, 0.1 eq.) of tetra-n-butylammonium hydrogen sulfate. Subsequently, 7.5 mL of dichloromethane and 28 mL of water were added. While cooling on ice and stirring vigorously, a solution of 629 µL (1.24 eq., 6.2 mmol, 1.026 g) of chloromethyl chlorosulfate in 14 mL of dichloromethane was slowly added dropwise to the resulting emulsion. After removing the ice bath and allowing the reaction to proceed at room temperature overnight, the aqueous and dichloromethane phases were separated using a separatory funnel. The aqueous phase was extracted twice with dichloromethane. The combined dichloromethane phases were washed with saturated sodium bicarbonate solution and then concentrated as much as possible in a rotary evaporator under reduced pressure.

General Synthetic Procedure B: Synthesis of Protected Acylals

An amount of 1.0 mmol (1.0 eq.) of the corresponding chloromethyl ester was mixed with 0.525 g (1.1 mmol, 1.1 eq.) of **3**, 204 µL (1.2 eq., 1.2 mmol, 0.151 g) of DIPEA, and 2 mL of dihydrolevoglucosenone. The reaction was carried out as a microwave-assisted synthesis at a target temperature of 150 °C. The progress of the synthesis was monitored by TLC. Typically, complete conversion of the chloromethyl ester was achieved within a maximum of 12 minutes. Subsequently, the reaction mixture was dissolved in 20 mL of ethyl acetate. The resulting solution was washed twice with 15 mL of saturated sodium bicarbonate solution and concentrated as much as possible on a rotary evaporator. The resulting residue was purified either by flash chromatography or by DCVC.

General Synthetic Procedure C: Deprotection of the Acylals

The protected acylal and a spatula tip of palladium on carbon were supended in 20 mL of dry methanol. After an argon atmosphere was established, hydrogen was slowly introduced into the methanolic solution. Once complete conversion of the reactant was confirmed by TLC (which occurred within a maximum of two hours), the reaction mixture was filtered through celite to remove the hydrogenation catalyst and concentrated as much as possible under partial vacuum in a rotary evaporator. The resulting residue was dissolved in 10 mL of a freshly prepared 3-molar solution of hydrogen chloride in ethyl acetate. (This solution was prepared beforehand by slowly adding 1.214 mL of methanol and 2.160 mL of acetyl chloride to 10 mL of ethyl acetate under ice cooling and vigorous stirring.) After TLC confirmed complete conversion of the reactant, which typically occurred after 10 to 15 minutes, this reaction mixture was concentrated to dryness under reduced pressure on a rotary evaporator.

3 Description and Analytical Characterisation of the Compounds

3.1 Protected Levodopa Building Blocks

Benzyl (2*S*)-3-[3,4-bis(benzyloxy)phenyl]-2-[(*tert*-butoxy)carbonyl]aminopropanoate (2)

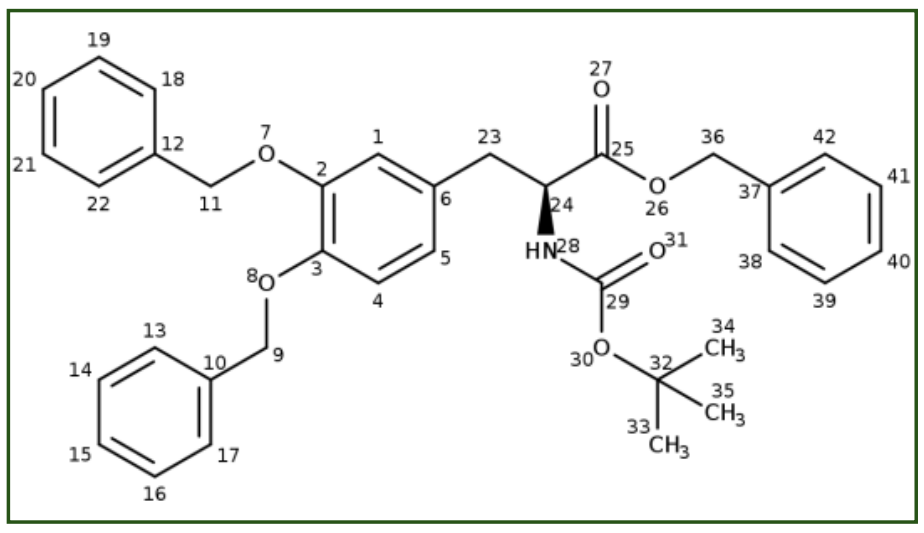

Under an argon atmosphere, 2.958 g (1.0 eq., 15.0 mmol) of levodopa were dissolved in 30 mL of degassed water. To this solution, 3.2 mL (1.5 eq., 22.5 mmol) of triethylamine and then 3.929 g (1.2 eq., 18.0 mmol) of di-*tert*-butyl dicarbonate, dissolved in 30 mL of acetone, were slowly added. Immediately afterward, the ice bath was removed. After five hours of reaction time, this reaction mixture was concentrated under reduced pressure using a rotary evaporator to remove the acetone, adjusted to a pH of approximately 2 with concentrated hydrochloric acid under ice cooling, and extracted three times with 15 mL of ethyl acetate each time. The combined ethyl acetate phases were concentrated as much as possible in the rotary evaporator under reduced pressure until a pale yellow oil remains. This oil was dissolved together with 3.27 g (1.6 eq., 23.7 mmol) of potassium carbonate and 3.7 mL (3.1 eq., 31.2 mmol) of benzyl bromide in 60 mL of acetone. This reaction mixture was heated under reflux for about 12 hours. Subsequently, 15 mL of water were added to the reaction mixture, and it was concentrated in a rotary evaporator under reduced pressure to remove the acetone, causing a pale yellow solid to precipitate. The resulting suspension was extracted three times with 15 mL of dichloromethane. The combined dichloromethane phases were washed with 15 mL of brine solution and then concentrated to dryness in a rotary evaporator under reduced pressure. The resulting pale yellow solid was recrystallized from ethyl acetate/hexane. Yield: 7.469 g of a colorless solid (13.2 mmol, 88%); m.p. = 110.2 °C. TLC: R_F = 0.54 in eluent I; ^{1}H NMR ($(CD_3)_2SO$): 7.35 – 6,73 (m, 19H, C(1)H, C(4)H, C(5)H, C(13)H, C(14)H, C(15)H, C(16)H, C(17)H, C(18)H, C(19)H, C(20)H, C(21)H, C(22)H, N(28)H, C(38)H, C(39)H, C(40)H, C(41)H, C(42)H), 5.07 (s, 4H, C(9)H, C(11)H), 5.06 (s, 2H, C(36)H), 4.23 – 4.17 (q, 1H, C(24)H, ^3J = 7.6 Hz), 2.95 – 2,77 (m, 2H, C(23)H), 1.33 (s, 9H, C(33)H, C(34)H, C(35)H); ^{13}C NMR ($(CD_3)_2SO$): 155.4 (C25), 148.1 (C29), 137.4 bis 101.1 (C1, C2, C3, C4, C5, C6, C10, C12, C13, C14, C15, C16, C17, C18, C19, C20, C21, C22, C37, C38, C39, C40, C41, C42), 78.26 (C32), 70.2 (C9, C11), 65.8 (C36), 55.5 (C23), 36.3 (C24), 28.1 (C33, C34, C35).

(2*S*)-3-[3,4-Bis(benzyloxy)phenyl]-2-[(*tert*-butoxy)carbonyl]aminopropanoic acid (3)

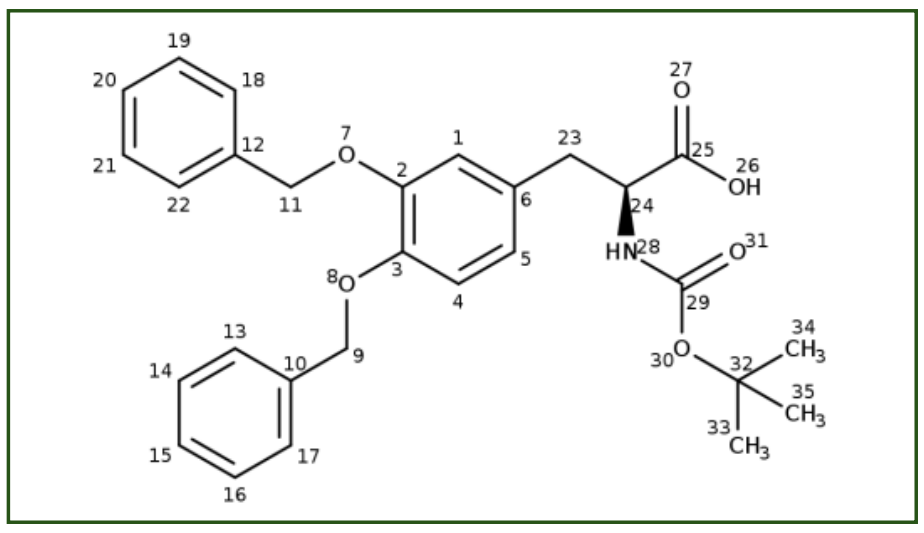

6.580 g (1.0 eq., 11.6 mmol) of **2** were dissolved in a mixture of 126 mL of 1,4-dioxane and 13 mL of water. While cooling with ice, 1.06 g (2.3 eq., 26.5 mmol) of sodium hydroxide were added, and the ice cooling is removed. After 24 hours of reaction time at room temperature, the solution was concentrated under reduced pressure in a rotary evaporator to reduce the 1,4-dioxane content. Under ice cooling, the pH of the solution was adjusted to approximately 2 using concentrated hydrochloric acid. The resulting solution was extracted three times with 20 mL of ethyl acetate. The combined ethyl acetate phases were freed of residual water by a single wash with 15 mL of brine solution and drying with magnesium sulfate, and were concentrated to dryness in a rotary evaporator under reduced pressure. The solid thus obtained was recrystallized from dichloromethane/hexane. Yield: 4.620 g of a colorless solid (9.7 mmol, 83%); m.p. = 111.1 °C; TLC: R_F =

0.67 in eluent IV; ^{1}H NMR (($CD_3)_2SO$): 12.55 (s, 1H, O(26)H), 7.66 – 6.46 (m, 14H, C(1)H, C(4)H, C(5)H, C(13)H, C(14)H, C(15)H, C(16)H, C(17)H, C(18)H, C(19)H, C(20)H, C(21)H, C(22)H, N(28)H), 5.08 (s, 4H, C(9)H, C(11)H), 4.11 – 4.02 (m, 1H, C(24)H), 2.96 – 2.65 (m, 2H, C(23)H), 1.32 (s, 9H, C(33)H, C(34)H, C(35)H); ^{13}C NMR (($CD_3)_2SO$): 155.4 (C25), 148.0 (C29), 146.9 – 114.4 (C1, C2, C3, C4, C5, C6, C10, C12, C13, C14, C15, C16, C17, C18, C19, C20, C21, C22), 78.0 (C32), 70.2 (C9, C11), 55.2 (C23), 36.0 (C24), 28.1 (C33, C34, C35).

3.2 Chloromethyl Esters

Chloromethyl (2*S*)-3-[3,4-bis(benzyloxy)phenyl]-2-[*tert*-butyl(oxycarbonylamino)]propionate (4)

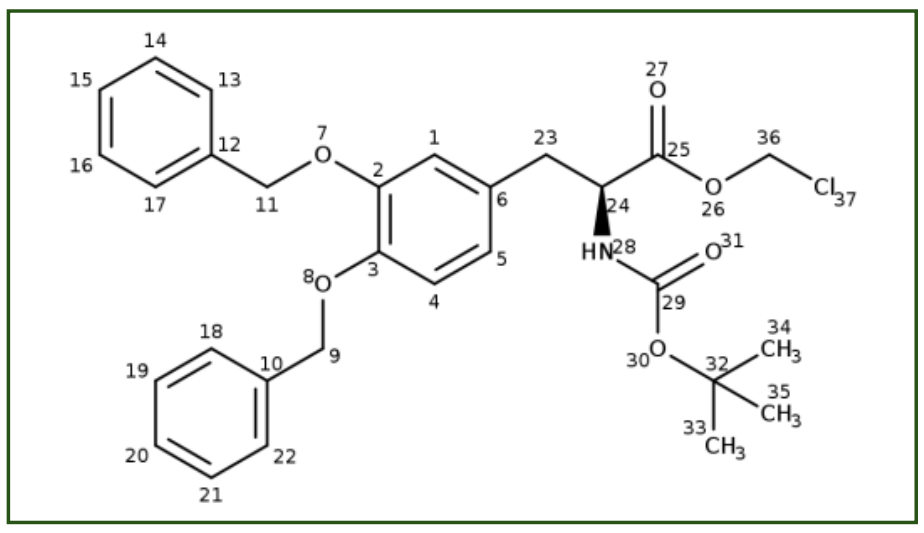

Using general synthetic procedure A, 1.667 g (3.5 mmol) of **3** were used as the reactant. Yield: 1.682 g of a yellowish solid (3.2 mmol, 91%); m.p. = 112.1 °C; TLC: R_F = 0.62 in eluent I; ^{1}H NMR (($CD_3)_2SO$): 7.54 – 6.63 (m, 14H, C(1)H, C(4)H, C(5)H, C(13)H, C(14)H, C(15)H, C(16)H, C(17)H, C(18)H, C(19)H, C(20)H, C(21)H, C(22)H, N(28)H), 5.86 (q, 2H, C(36)H, ^2J = 12.0 Hz), 5.08 (s, 4H, C(9)H, C(11)H), 4.23 (m, 1H, C(24)H), 2.84 (m, 2H, C(23)H), 1.33 (s, 9H, C(33)H, C(34)H, C(35)H); ^{13}C NMR (($CD_3)_2SO$): 155.3 (C25), 148.1 (C29), 137.6 – 114.3 (C1, C2, C3, C4, C5, C6, C10, C12, C13, C14, C15, C16, C17, C18, C19, C20, C21, C22), 78.5 (C32), 70.2 (C9, C11), 69.7 (C36), 55.2 (C24), 35.8 (C23), 28.1 (C33, C34, C35); HRAMS (ESI – QTOF) calculated for $C_{29}H_{32}ClNO_6$ + Na^+: 548.1816, found 548.1816.

Chloromethyl 5-phenylvalerate (5b)

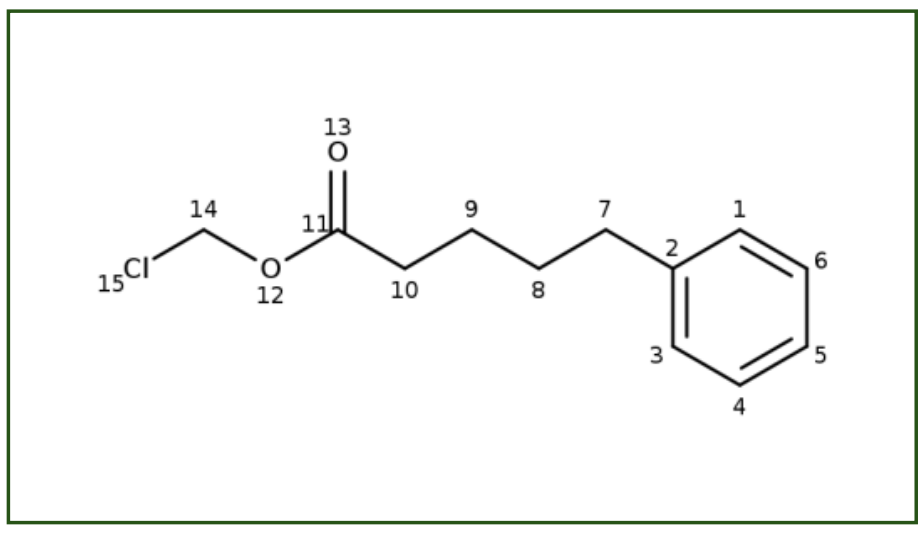

Using general synthetic procedure A, 0.891 g (5 mmol) of 5-phenylvaleric acid were used as an educt. Yield: 1.099 g of a colorless oil (4.85 mmol, 97%); TLC: R_F = 0.70 in eluent V; ^{1}H NMR (($CD_3)_2SO$): 7.33 – 7.10 (m, 5H, C(1)H, C(3)H, C(4)H, C(5)H, C(6)H), 5.84 (s, 2H, C(14)H), 2.58 (t, 2H, C(7)H, ^3J = 8.0 Hz), 2.44 (t, 2H, C(10)H, ^3J = 8.0 Hz), 1.57 (m, 4H, C(8)H, C(9)H); ^{13}C NMR (($CD_3)_2SO$): 171.3 (C11), 141.8 (C2), 128.2 (C1, C3, C4, C6), 125.7 (C5), 69.3 (C14), 34.6 (C7), 32.9 (C10), 30.0 (C9), 23.6 (C8).

Chloromethyl (*E*)-cinnamate (5c)

The synthesis and characterisation of compound **4d** was described in our previously published paper (see reference 30 in the main article).

Chloromethyl 4-phenoxybutyrate (5e)

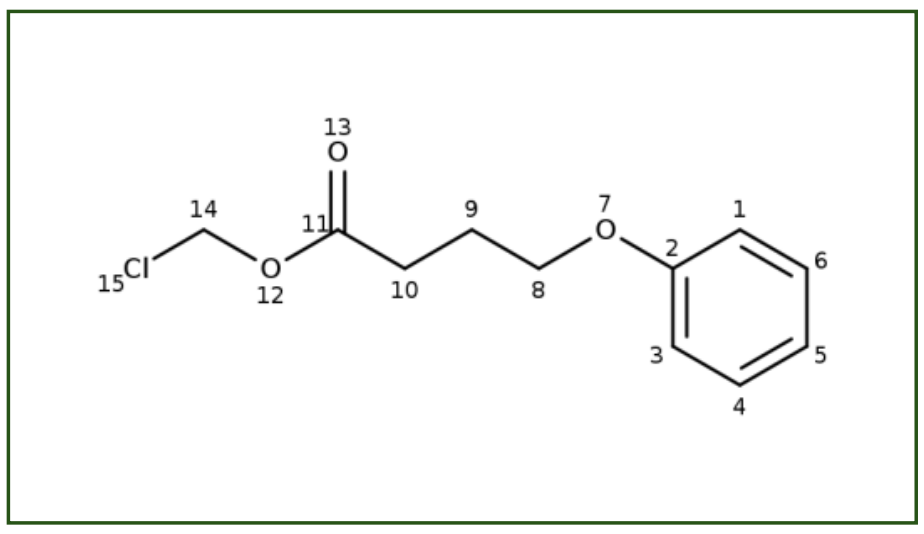

Using general synthetic procedure A, 0.901 g (5 mmol) 4-phenoxybutyric acid were used as an educt. Yield: 1.120 g of a colorless liquid (4.90 mmol, 98%); TLC: R_F = 0.63 in eluent III; ^{1}H NMR ($(CD_3)_2SO$): 7.31 – 7.24 (m, 2H, C(4)H, C(6)H), 6.95 – 6.89 (m, 3H, C(1)H, C(3)H, C(5)H), 5.86 (s, 2H, C(14)H), 3.99 (t, 2H, C(10)H, ^3J = 6.3 Hz), 2.59 (t, 2H, C(8)H, ^3J = 7.3 Hz), 2.00 (m, 2H, C(9)H); ^{13}C NMR ($(CD_3)_2SO$): 171.1 (C11), 158.4 (C2), 129.5 (C4, C6), 120.5 (C5), 114.4 (C1, C3), 69.4 (C14), 66.1 (C10), 29.9 (C8), 23.9 (C9); HRAMS (ESI – QTOF) calculated for $C_{11}H_{13}ClO_3$ + Na^+: 251.0451, found 251.0445.

Chloromethyl 2-phenylbutyrate (5f)

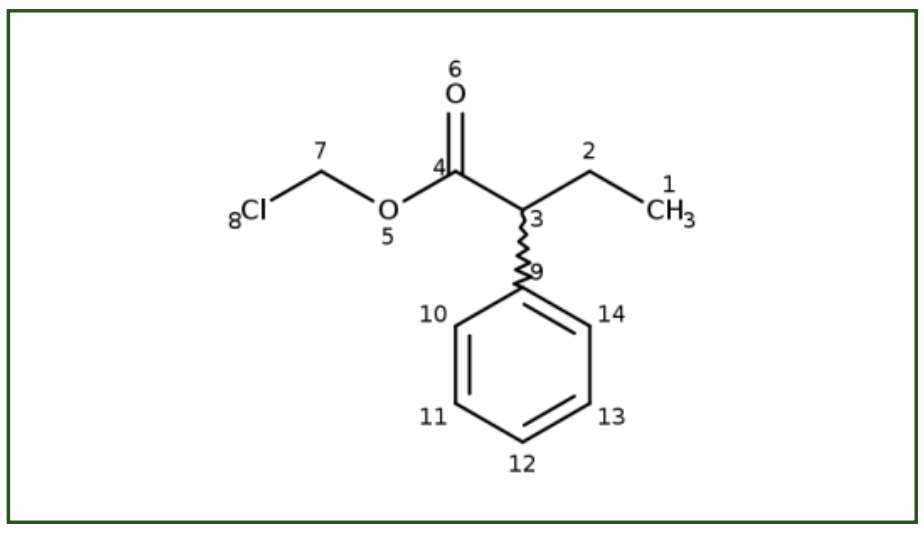

Using general synthetic procedure A, 0.821 g (5 mmol) 2-phenylbutanoic acid were used as educt. The raw product was purified using DCVC. Yield: 0.959 g of a colorless liquid (4.51 mmol, 90%); TLC: R_F = 0.82 in eluent III; ^{1}H NMR ($(CD_3)_2SO$): 7.42 – 7.20 (m, 5H, C(10)H, C(11)H, C(12)H, C(13)H, C(14)H), 5.85 (q, 2H, C(7)H, ^2J = 6.1 Hz), 3.67 (t, 1H, C(3)H, ^3J = 7.6 Hz), 2.01 and 1.73 (m, 2H, C(2)H), 0.83 (t, 3H, C(1)H, ^3J = 7.3 Hz); ^{13}C NMR ($(CD_3)_2SO$): 171.6 (C4), 137.9 (C9), 128.6 (C11, C13), 127.8 (C10, C14), 127.4 (C12), 69.6 (C7), 51.7 (C3), 26.0 (C2), 11.6 (C1).

Chloromethyl 2-furoate (5g)

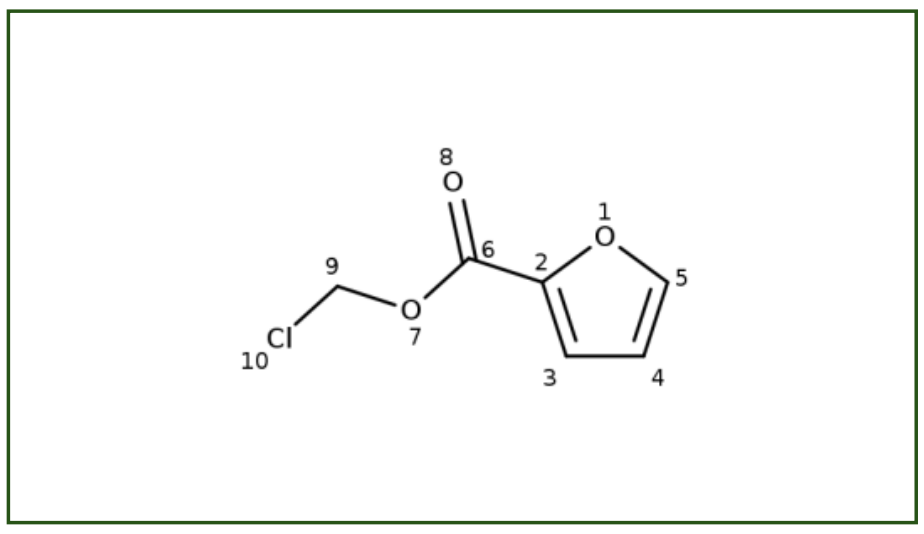

Using general synthetic procedure A, 0.560 g (5 mmol) of furan-2-carboxylic acid were used as educt. The raw product was purified using DCVC. Yield: 0.524 g of a colorless solid (3.25 mmol, 65%); m.p. = 53.4 °C. TLC: R_F = 0.64 in eluent III; ^{1}H NMR ($(CD_3)_2SO$): 8.09 (dd, 1H, C(3)H, ^3J = 0.7 Hz; 1.6 Hz), 7.50 (dd, 1H, C(5)H, ^3J = 0.6 Hz; 3.6 Hz), 6.77 (dd, 1H, C(4)H, ^3J = 1.7 Hz; 3.6 Hz), 6.07 (s, 2H, C(9)H); ^{13}C NMR ($(CD_3)_2SO$): 155.7 (C6), 149.1 (C5), 142.3 (C2), 120.7 (C3), 112.8 (C4), 69.8 (C9).

Chloromethyl 2-phenoxybenzoate (5h)

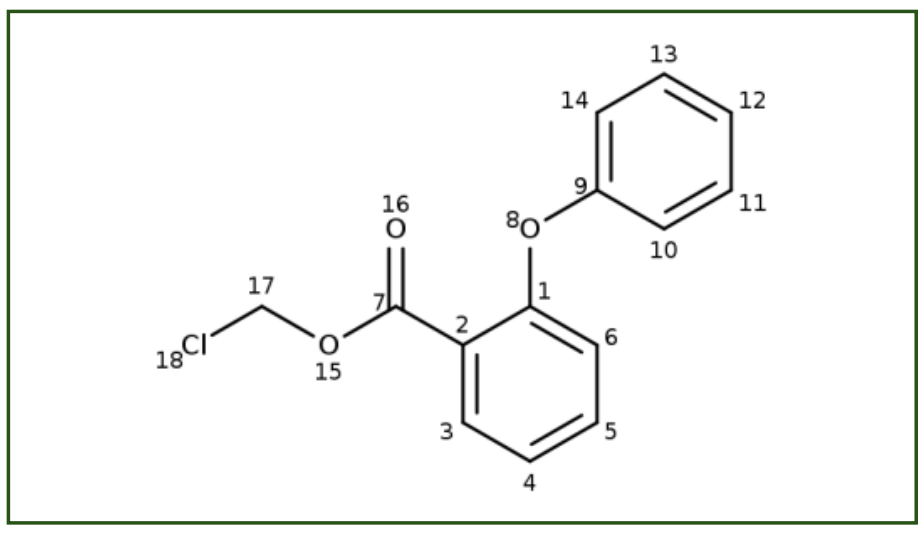

Using general synthetic procedure A, 1.071 g (5 mmol) 2-phenoxybenzoic acid were used as educt. The raw product was purified using DCVC. Yield: 1.213 g of a colorless solid (4.60 mmol, 92%); m.p. = 48.9 °C; TLC: R_F = 0.58 in eluent V; ^{1}H NMR ((CD$_3$)$_2$SO): 7.92 (dd, 1H, C(3)H, ^3J = 7.8 Hz; 1.9 Hz), 7.67 (t, 1H, C(5)H, ^3J = 8.0 Hz), 7.39 (t, 2H, C(11)H, C(13)H, ^3J = 8.0 Hz), 7.32 (t, 1H, C(6)H, ^3J = 8.0 Hz); 7.15 (t, 1H, C(4)H, ^3J = 8.0 Hz), 7.04 (d, 1H, C(12)H, ^3J = 8.0 Hz), 6.97 (m, 2H, C(10), C(14)), 6.01 (s, 2H, C(17)H); ^{13}C NMR ((CD$_3$)$_2$SO): 162.6 (C7), 156.7 (C9), 156.0 (C1), 135.3 (C3), 131.7 (C5), 130.0 (C11, C13), 124.0 (C12), 123.6 (C6), 120.8 (C2), 120.6 (C4), 118.1 (C10, C14), 69.9 (C17).

Chloromethyl 4-phenoxybenzoate (5i)

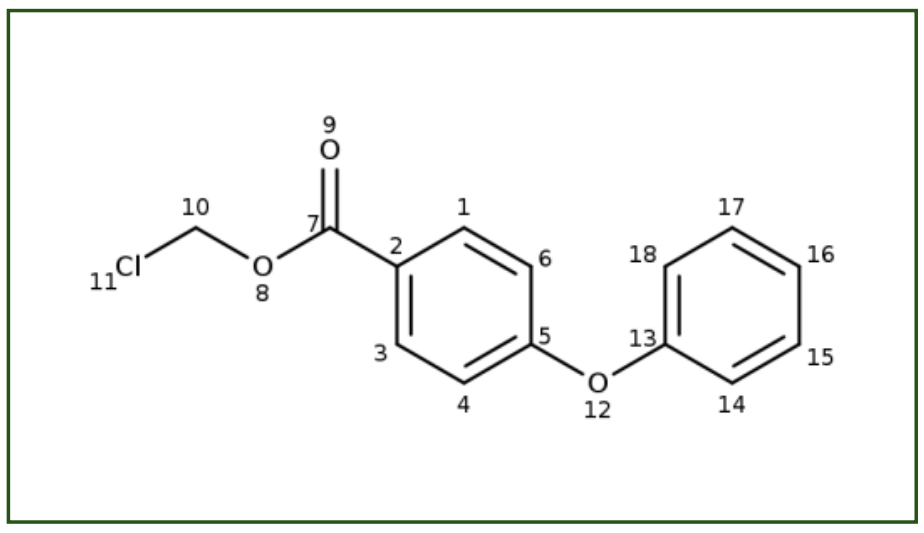

Using general synthetic procedure A, 1.071 g (5 mmol) 4-phenoxybenzoic acid were used as educt. The raw product was purified using DCVC. Yield: 1.156 g of a colorless solid (4.40 mmol, 88%); m.p. = 45.6 °C; TLC: R_F = 0.73 in eluent V; ^{1}H NMR ((CD$_3$)$_2$SO): 8.03 (m, 2H, C(1)H, C(3)H), 7.48 (m, 2H, C(4)H, C(6)H), 7.28 (m, 1H, C(16)H), 7.16 (m, 2H, C(15)H, C(17)H), 7.09 (m, 2H, C(14)H, C(18)H), 6.10 (s, 2H, C(10)H); ^{13}C NMR ((CD$_3$)$_2$SO): 166.7 (C2), 163.4 (C7), 162.3 (C5), 154.8 (C13), 131.9 (C4, C6), 130.3 (C15, C17), 124.9 (C1, C3), 120.1 (C14, C18), 117.2 (C16), 70.1 (C10).

Chloromethyl 2-(4-isobutylphenyl)propionate (5j)

The synthesis and characterisation of compound **5k** was described in our previously published paper (see reference 30 in the main article).

Chloromethyl (2*S*)-2-acetylamino-3-phenylpropionate (5k)

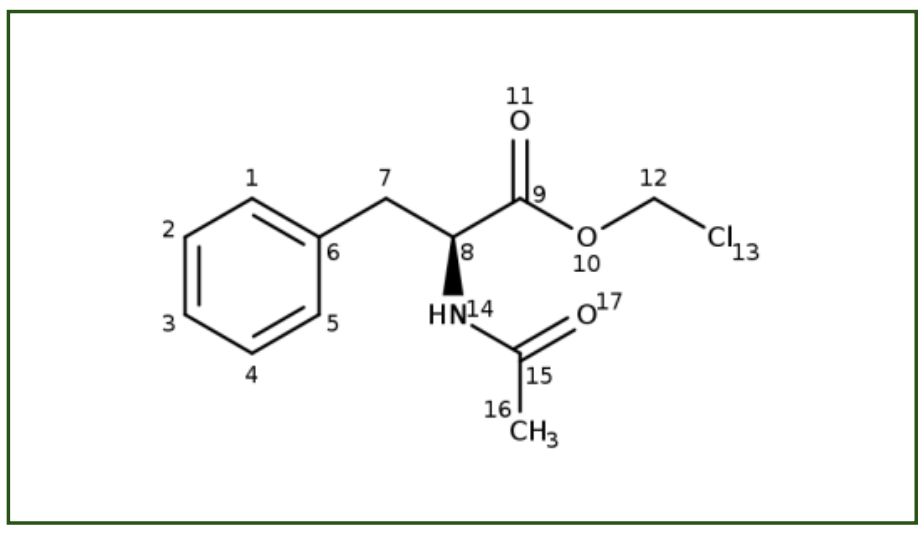

1.652 g (10 mmol, 1 Äq.) of phenylalanine were suspended in 8 mL of water. To this mixture, 9.4 mL (100 mmol, 10 eq, 0.108 g) of acetic anhydride were added. This reaction mixture is kept in a water bath at 45 °C for 60 minutes under ultrasonic agitation. The mixture is then concentrated as much as possible in a rotary evaporator under reduced pressure. The resulting residue is then processed according to general synthetic procedure A without further purification; however, all quantities of substances specified there are used in double the amount. Yield: 1.331 g of a colorless solid (5.20 mmol, 52%); m.p. = 73.7 °C; TLC: R_F = 0.35 in eluent II; ^{1}H NMR ((CD$_3$)$_2$SO):

8.46 (d, 1H, N(14)H, ${}^{3}\mathcal{J}$ = 7.5 Hz), 7.26 (m, 5H, C(1)H, C(2)H, C(3)H, C(4)H, C(5)H), 5.86 (dd, 2H, C(12)H, ${}^{2}\mathcal{J}$ = 2.1 Hz; 1.1 Hz), 4.50 (m, 1H, C(8)H), 2.97 (m, 2H, C(7)H), 1.80 (s, 3H, C(16)H); ^{13}C NMR (($CD_3)_2SO$): 170.2 (C9), 169.5 (C15), 136.8 (C6), 129.1 (C1, C5), 128.3 (C2, C4), 126.7 (C3), 69.7 (C12), 53.5 (C8), 36.0 (C7), 22.1 (C16); HRAMS (ESI − QTOF) calculated for $C_{12}H_{14}ClNO_3$ + H^+: 256.0740, found 256.0738.

Chloromethyl oleate (5l)

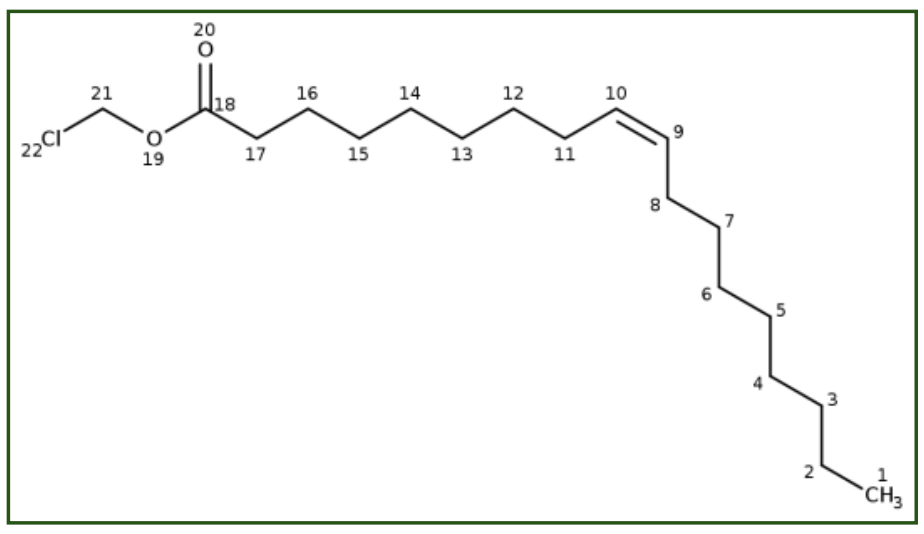

Using general synthetic procedure A, 1.412 g (5 mmol) oleic acid were used as educt. The raw product was purified using DCVC. Yield: 1.473 g of a colorless liquid (4.45 mmol, 89%); TLC: R_F = 0.88 in eluent V; ^{1}H NMR (($CD_3)_2SO$): 5.83 (s, 2H, C(21)H), 5.32 (m, 2H, C(9)H, C(10)H), 2.39 (t, 2H, C(17)H, ${}^{3}\mathcal{J}$ = 8.0Hz), 1.97 (m, 2H, C(8)H, C(11)H), 1.53 (m, 2H, C(16)H), 1.25 (m, 20H, C(2)H, C(3)H, C(4)H, C(5)H, C(6)H, C(7)H, C(12)H, C(13)H, C(14)H, C(15)H), 0.85 (t, 3H, C(1)H, ${}^{3}\mathcal{J}$ = 8.0 Hz); ^{13}C NMR (($CD_3)_2SO$): 171.3 (C18), 129.6 (C9, C10), 69.2 (C21), 33.1 (C17), 31.2 (C3), 29.1 − 28.2 (C4, C5, C6, C12, C13, C14, C15), 26,5 (C8, C11), 24.0 (C16), 22.1 (C2), 13.9 (C1).

Chloromethyl 2-(acetyloxy)benzoate (5m)

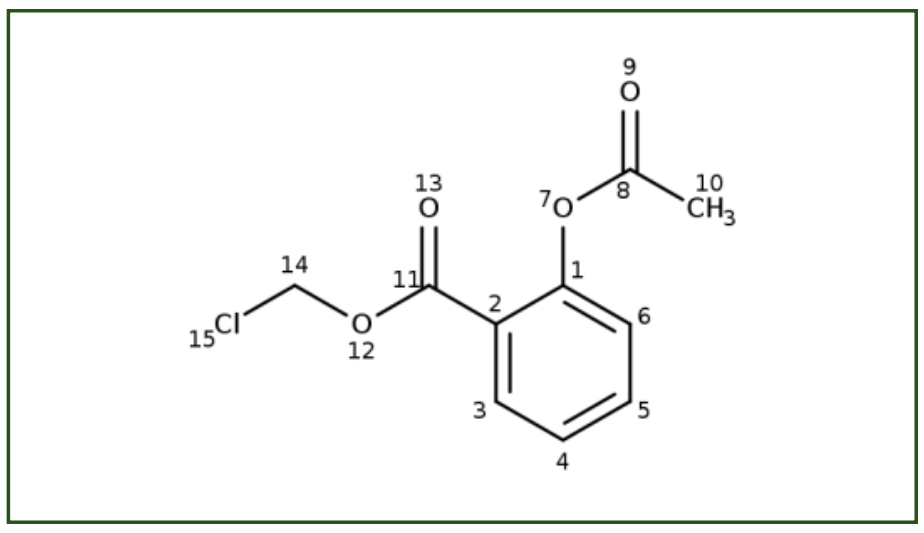

Using general synthetic procedure A, 0.901 g (5 mmol) of acetylsalicylic acid were used as educt. The raw product was purified using DCVC. Yield: 0.943 g of a colorless liquid (4,1 mmol, 82%); TLC: R_F = 0.47 in eluent III; ^{1}H NMR (($CD_3)_2SO$): 7.99 (dd, 1H, C(3)H, ${}^{3}\mathcal{J}$ = 7.9 Hz; 1.1 Hz), 7.76 (ddd, 1H, C(5)H, ${}^{3}\mathcal{J}$ = 8.2 Hz; 7.4 Hz; 1.8 Hz), 7.47 (td, 1H, C(4)H, ${}^{3}\mathcal{J}$ = 7.7 Hz; 1.1 Hz), 7.31 (dd, 1H, C(6)H, ${}^{3}\mathcal{J}$ = 8.2 Hz; 1.1 Hz), 6.07 (s, 2H, C(14)H), 2.32 (s, 3H, C(10)H); ^{13}C NMR (($CD_3)_2SO$): 169.1 (C8), 162.1 (C11), 150.4 (C1), 135.4 (C3), 131.5 (C5), 126.6 (C6), 124.3 (C4), 121.5 (C2), 69.9 (C14), 20.7 (C10).

Chloromethyl 1-adamantanecarboxylate (5n)

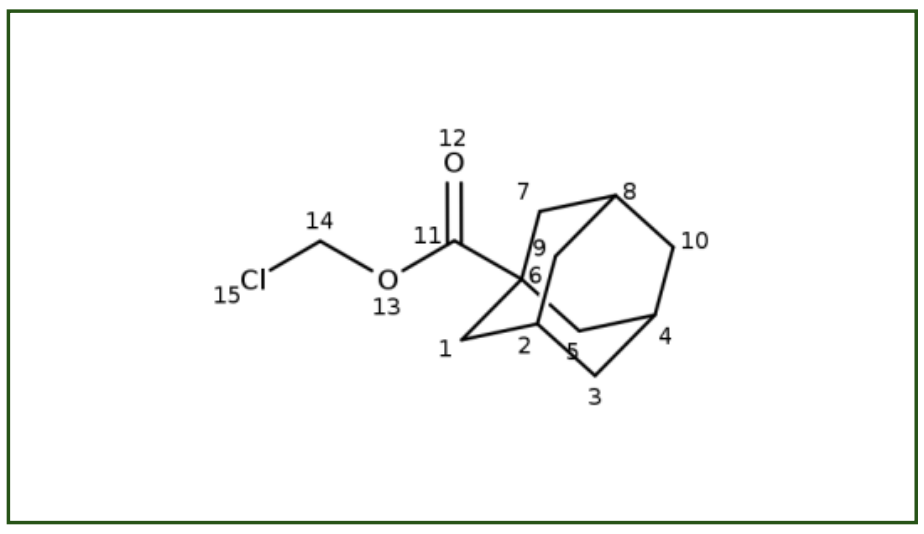

Using general synthetic procedure A, 0.229 g (5 mmol) 1-adamantanecarboxylic acid were used as educt. The raw product was purified using DCVC. Yield:0.500 g of a colorless liquid (3.75 mmol, 75%); TLC: R_F = 0.85 in eluent V; ^{1}H NMR (($CD_3)_2SO$): 5.85 (s, 2H, C(14)H), 1.98 (m, 3H, C(2)H, C(4)H, C(8)H), 1.83 (d, 6H, C(1)H, C(5), H(7), ${}^{3}\mathcal{J}$ = 4.0 Hz), 1.68 (m, 6H, C(3)H, C(9)H, C(10)H); ^{13}C NMR (($CD_3)_2SO$): 174.5 (C11), 69.4 (C14), 40.1 (C6), 37.8 (C1, C5, C7), 35.7 (C3, C9, C10), 27.1 (C2, C4, C8).

3.3 Protected Acylals

Benzoyloxymethyl (2*S*)-3-[3,4-bis(benzyloxy)phenyl]-2-[*tert*-butyl(oxycarbonylamino)]propionate (6a)

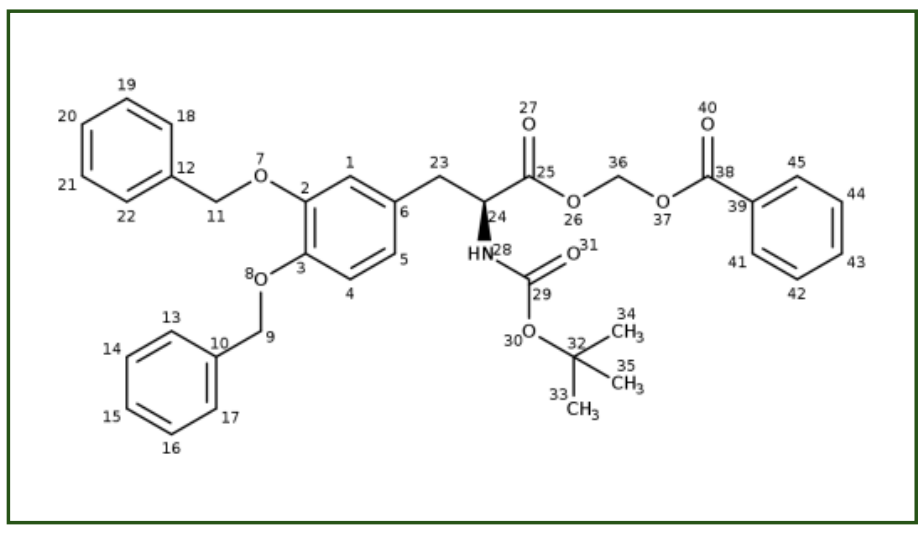

Using general synthetic procedure B, 0.171 g (1 mmol) of **5a** is used as the starting material. Flash chromatography is employed as the purification method. In this case, the product is additionally recrystallized from ethyl acetate/hexane. Yield: 0.281 g of a colorless solid (0.46 mmol, 46%); m.p. = 112.0 °C; TLC: R_F = 0.67 in eluent IV; ^{1}H NMR ($(CD_3)_2SO$): 7.97 (m, 2H, C(41)H, C(45)H), 7.71 (m, 1H, C(43)H), 7.55 (m, 2H, C(42)H, C(44)H), 7.46 − 7.25 (m, 10H, C(13), C(14)H, C(15)H, C(16)H, C(17)H, C(18)H, C(19)H, C(20)H, C(21)H, C(22)H), 7.03 (m, 1H, C(1)H), 6.86 (m, 1H, C(4)H), 6.73 (m, 1H, C(5)H), 5.96 (dd, 2H, C(36)H, ^{2}J = 3.6 Hz; 2.0 Hz), 5.04 (s, 4H, C(9)H, C(11)H), 4.20 (m, 1H, C(24)H), 2.85 (m, 2H, C(23)H), 1.28 (s, 9H, C(33)H, C(34)H, C(35)H); ^{13}C NMR ($(CD_3)_2SO$): 171.1 (C25), 164.4 (C38), 148.0 (C29), 147.0 (C10, C12), 137.3 (C2, C3), 134.1 (C43), 130.1 (C6), 129.5 (C41, C45), 128.9 (C42, C44), 128.5 (C39), 128.3 (C14, C16, C19, C21), 127.7 (C15, C20), 127.5 (C13, C17, C18, C22), 121.8 (C5), 115.6 (C1), 114.3 (C4), 79.9 (C36), 78.4 (C32), 70.1 (C9, C11), 55.3 (C24), 35.6 (C23), 28.0 (C33, C34, C35); HRAMS (ESI – QTOF) calculated for $C_{36}H_{37}NO_8$ + Na^+: 634.2417, found 634.2435.

(5-Phenylvaleroxy)methyl (2*S*)-3-[3,4-bis(benzyloxy)phenyl]-2-[*tert*-butyl(oxycarbonylamino)]-propionate (6b)

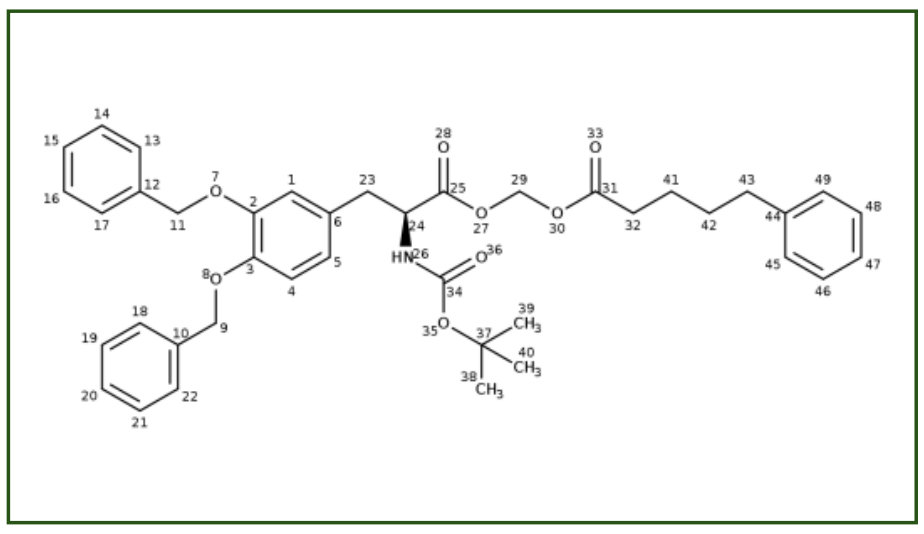

Using general synthetic procedure B, 0.150 g (1 mmol) **5b** were used as educt. Flash chromatography is employed as the purification method. Yield: 0.338 g of a colorless solid (0.58 mmol, 58%); m.p. = 75.3 °C; TLC: R_F = 0.65 in eluent IV; ^{1}H NMR ($(CD_3)_2SO$): 7.47 − 7.12 (m, 16H, C(13)H, C(14)H, C(15)H, C(16)H, C(17)H, C(18)H, C(19)H, C(20)H, C(21)H, C(22)H, N(26)H, C(46)H, C(47)H, C(48)H, C(49)H), 7.02 (m, 1H, C(1)H), 6.95 (m, 1H, C(4)H), 6.75 (m, 1H, C(5)H), 5.69 (dd, 2H, C(29)H, ^{2}J = 4.3 Hz; 3.0 Hz), 5.08 (s, 4H, C(9)H, C(11)H), 4.17 (m, 1H, C(24)H), 2.83 (m, 2H, C(23)H), 2.55 (t, 2H, C(43)H, ^{3}J = 8.0 Hz), 2.36 (t, 2H, C(32)H, ^{3}J = 8.0 Hz), 1.54 (m, 4H, C(41)H, C(42)H), 1.32 (s, 9H, C(38)H, C(39)H, C(40)H); ^{13}C NMR ($(CD_3)_2SO$): 171.6 (C31), 171.1 (C25), 184.1 (C34), 147.0 (C10, C12), 141.8 (C44), 137.3 (C2, C3), 130.1 (C6), 128.3 (C14, C16, C19, C21), 128.2 (C45, C46, C48, C49), 127.7 (C15, C20), 127.5 (C13, C17, C18, C22), 125.6 (C47), 121.8 (C5), 115.6 (C1), 114.4 (C4), 79.1 (C29), 78.4 (C37), 70.2 (C9, C11), 55.2 (C24), 35.6 (C23), 34.7 (C43), 32.9 (C32), 30.1 (C41), 28.1 (C38, C39, C40), 23.7 (C42); HRAMS (ESI – QTOF) calculated for $C_{40}H_{45}NO_8$ + Na^+: 690.3043, found 690.3050.

[(*E*)-2-Phenylethenylcarbonyloxy]methyl (2*S*)-3-[3,4-bis(benzyloxy)phenyl]-2-[*tert*-butyl(oxy-carbonylamino)]propionate (6c)

Using general synthetic procedure B, 0.144 g (1 mmol) **5c** were used as educt. Flash chromatography is employed as the purification method. Yield: 0.478 g of a colorless solid (0,75 mmol, 75%); m.p. = 110.3 °C;

TLC: R_F = 0.56 in eluent I; ^{1}H NMR ($(CD_3)_2SO$): 7.74 (m, 2H, C(43)H, C(47)H), 7.71 (m, 1H, C(43)H), 7.55 – 7.27 (m, 14H, C(13)H, C(14)H, C(15)H, C(16)H, C(17)H, C(18)H, C(19)H, C(20)H, C(21)H, C(22)H, N(26)H, C(44)H, C(45)H, C(46)H), 7.03 (s, 1H, C(1)H), 6.91 (d, 1H, C(4)H, ^3J = 8.0 Hz), 6.70 (m, 3H, C(5)H, C(32)H, C(41)H), 5.82 (dd, 2H, C(29)H, ^2J = 20.0 Hz; 4.0 Hz), 5.04 (s, 4H, C(9)H, C(11)H), 4.18 (m, 1H, C(24)H), 2.85 (m, 2H, C(23)H), 1.31 (s, 9H, C(33)H, C(34)H, C(35)H); ^{13}C NMR ($(CD_3)_2SO$): 164.8 (C25), 155.3 (C31), 148.1 (C29), 147.0 (C10, C12), 137.3 (C2, C3), 133.7 (C41), 130.9 (C6), 129.0 – 127.6 (C14, C15, C16, C19, C20, C21, C42, C43, C44, C45, C46, C47), 127.4 (C13, C17, C18, C22), 121.8 (C5), 116.7 (C32), 115.6 (C1), 114.4 (C4), 79.4 (C36), 78.4 (C32), 70.2 (C9, C11), 55.3 (C24), 35.6 (C23), 28.1 (C33, C34, C35); HRAMS (ESI – QTOF) calculated for $C_{38}H_{39}NO_8$ + Na^+: 660.2573, found 660.2572.

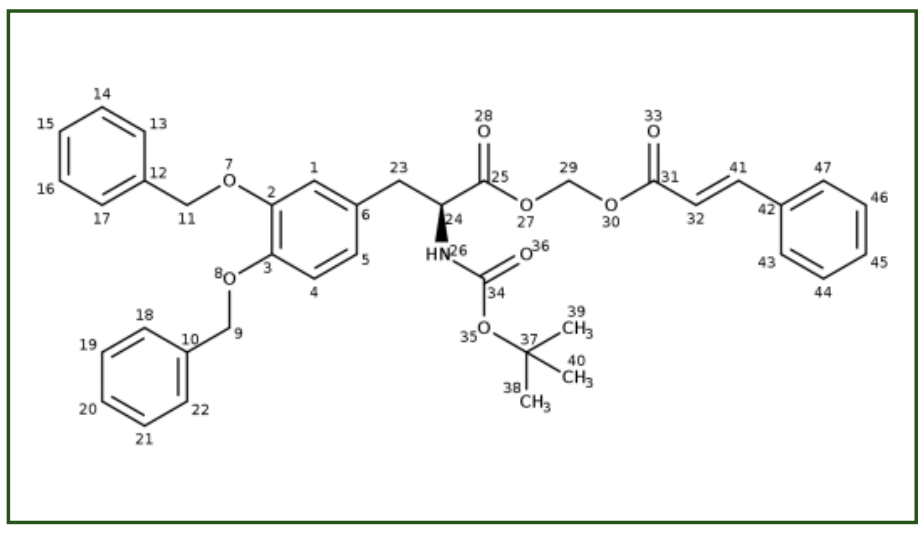

{(2*S*)-3-[3,4-Di(benzyloxy)phenyl]-2-[*tert*-butyl(oxycarbonylamino)]propanoyl}oxymethyl 2,2-dimethylpropionate (6d)

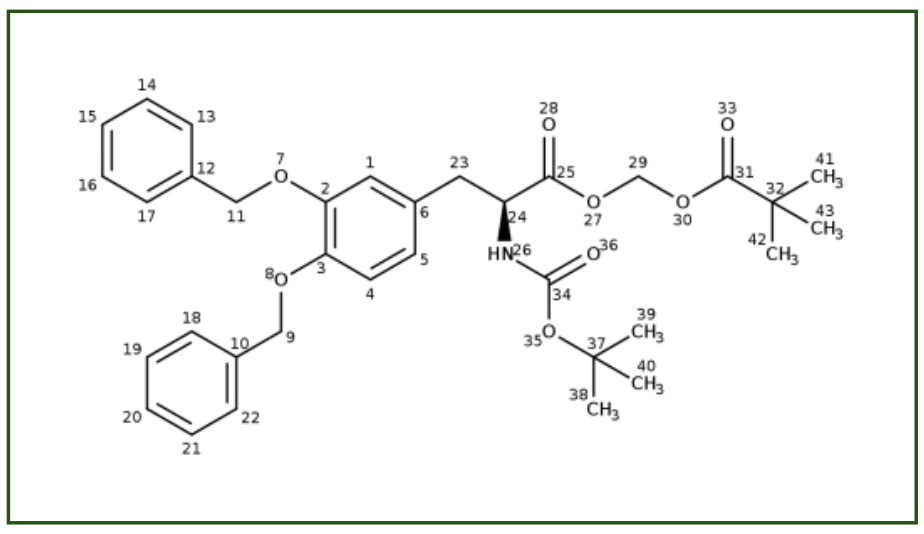

Using general synthetic procedure B, 0.144 mL (1 mmol, 0.150 g) **5d** were used as educt. Flash chromatography is employed as the purification method. Yield: 0.400 g of a colorless solid (0.68 mmol, 68%); m.p. = 107.5 °C; TLC: R_F = 0.53 in eluent III; ^{1}H NMR ($(CD_3)_2SO$): 7.47 – 7.28 (m, 11H, C(13)H, C(14)H, C(15)H, C(16)H, C(17)H, C(18)H, C(19)H, C(20)H, C(21)H, C(22)H, N(26)H), 7.04 (m, 1H, C(1)H), 6.95 (m, 1H, C(4)H), 6.77 (m, 1H, C(5)H), 5.71 (dd, 2H, C(29)H, ^2J = 2.6 Hz; 1.3 Hz), 5.08 (s, 4H, C(9)H, C(11)H), 4.17 (m, 1H, C(24)H), 2.83 (m, 2H, C(23)H), 1.33 (s, 9H, C(38)H, C(39)H, C(40)H), 1.14 (s, 9H, C(41)H, C(42)H, C(43)H); ^{13}C NMR ($(CD_3)_2SO$): 176.1 (C31), 171.2 (C25), 148.1 (C34), 147.0 (C10, C12), 137.3 (C2, C3), 130.1 (C6), 128.3 (C14, C16, C19, C21), 127.7 (C15, C20), 127.5 (C13, C17, C18, C22), 121.8 (C5), 115.6 (C1), 114.4 (C4), 79.4 (C29), 78.4 (C37), 70.1 (C9, C11), 55.2 (C24), 38.2 (C32), 35.5 (C23), 28.1 (C38, C39, C40), 26.5 (C41, C42, C43); HRAMS (ESI – QTOF) calculated for $C_{40}H_{45}NO_8$ + Na^+: 690.3043, found 690.3050.

(4-Phenoxybutyroxy)methyl (2*S*)-3-[3,4-bis(benzyloxy)phenyl]-2-[*tert*-butyl(oxycarbonylamino)]-propionate (6e)

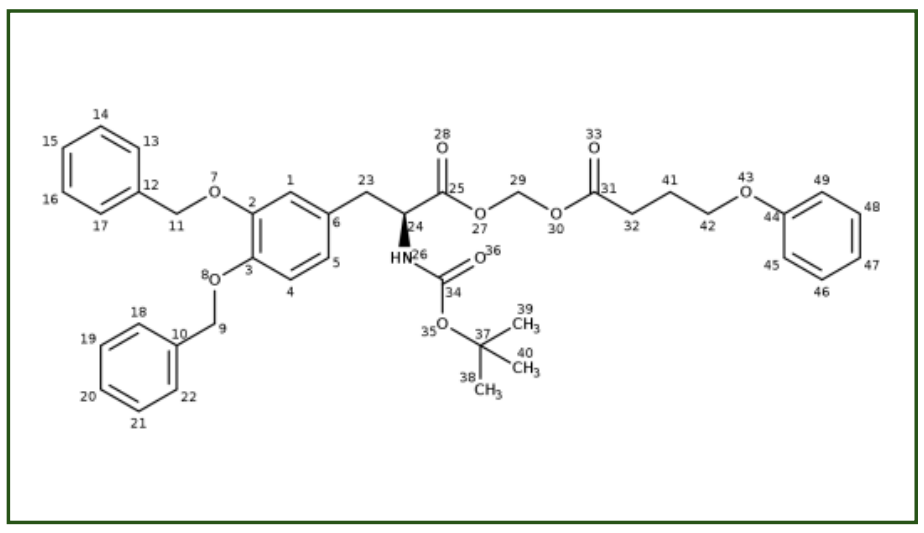

Using general synthetic procedure B, 0.229 g (1 mmol) **5e** were used as educt. Flash chromatography is employed as the purification method. Yield: 0.248 g of a colorless solid (0.37 mmol, 37%); m.p. = 87.1 °C; TLC: R_F = 0.54 in eluent III; ^{1}H NMR ($(CD_3)_2SO$): 7.46 – 7.23 (m, 13H, C(13)H, C(14)H, C(15)H, C(16)H, C(17)H, C(18)H, C(19)H, C(20)H, C(21)H, C(22)H, C(46)H, C(48)H), 7.02 (d, 1H, C(1)H, ^3J = 1.7 Hz), 6.96 – 6.88 (m, 4H, C(4)H, C(45)H, C(47)H, C(49)H), 6.75 (dd, 1H, C(5)H, ^3J = 8.4 Hz; 1.6 Hz), 5.71 (dd, 2H, C(29)H, ^2J = 3.6 Hz; 2.5 Hz), 5.08 (s, 4H, C(9)H, C(11)H), 4.17 (m, 1H, C(24)H), 3.96

(t, 2H, C(32)H, ^3J = 6.3 Hz), 2.83 (m, 2H, C(23)H), 1.96 (qui, 2H, C(41)H, ^3J = 6.8 Hz), 1.32 (s, 9H, C(38)H, C(39)H, C(40)H); ^{13}C NMR ($(CD_3)_2SO$): 171.2 (C25, C31), 158.3 (C44), 148.0 (C34), 147.0 (C10, C12), 137.3 (C2, C3), 130.1 (C6), 129.4 (C46, C48), 128.3 (C14, C16, C19, C21), 127.7 (C15, C20), 127.5 (C13, C17, C18, C22), 121.8 (C5), 120.5 (C47), 115.6 (C1), 114.4 (C4, C45, C49), 79.3 (C29), 78.4 (C37), 70.2 (C9, C11), 66.0 (C32), 55.2 (C24), 35.6 (C23), 29.9 (C42), 28.1 (C38, C39, C40), 23.9 (C41); HRAMS (ESI − QTOF) calculated for $C_{39}H_{43}NO_9 + Na^+$: 692.2836, found 692.2833.

[(2*S*)-2-(*tert*-Butoxycarbonylamino)-3-(3,4-dibenzyloxyphenyl)propanoyl]oxymethyl 2-phenylbutanoate (6f)

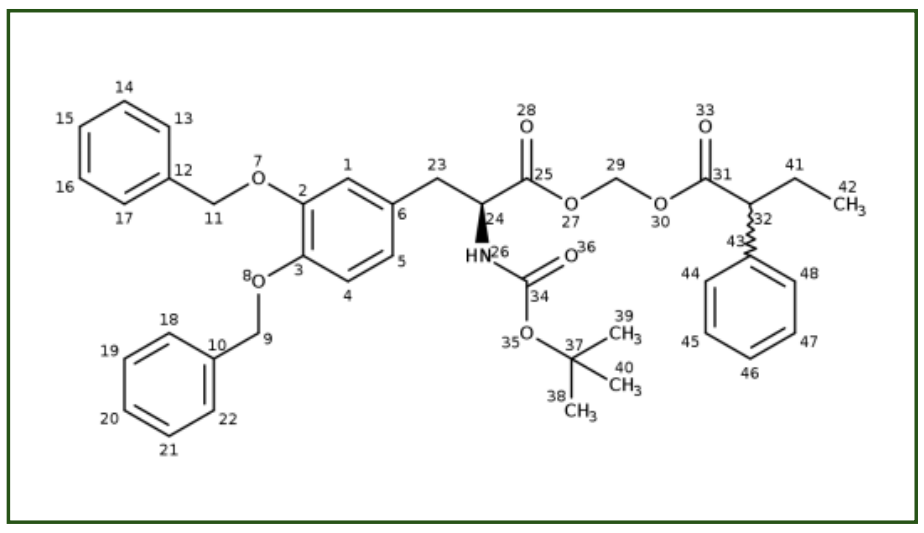

Using general synthetic procedure B, 0.213 g (1 mmol) of **5f** were used as educt. Flash chromatography is employed as the purification method. Yield: 0.534 g of a colorless solid (0.82 mmol, 82%); m.p. = 90.2 °C; TLC: R_F = 0.70 in eluent IV; ^{1}H NMR ($(CD_3)_2SO$): 7.97 − 7.20 (m, 16H, C(13)H, C(14)H, C(15)H, C(16)H, C(17)H, C(18)H, C(19)H, C(20)H, C(21)H, C(22)H, N(26)H, C(44)H, C(45)H, C(46)H, C(47)H, C(48)H), 6.99 (s, 1H, C(1)H), 6.94 (d, 1H, C(4)H, ^3J = 8.2 Hz), 6.71 (d, 1H, C(5)H, ^3J = 8.0 Hz), 5.72 (m, 2H, C(29)H), 5.09 (s, 4H, C(9)H, C(11)H), 4.12 (m, 1H, C(24)H), 3.58 (t, 1H, C(32)H, ^3J = 7.1 Hz), 2.68 (m, 2H, C(23)H), 2.02 − 1.70 (m, 2H, C(41)H), 1.32 (s, 9H, C(33)H, C(34)H, C(35)H), 0.81 (t, 3H, C(42)H, ^3J = 7.0 Hz); ^{13}C NMR ($(CD_3)_2SO$): 172.0 (C25), 170.1 (C31), 148.1 (C34), 147.1 (C10, C12), 137.4 (C2, C3), 128.6 (C43), 128.3 (C14, C16, C19, C21, C44, C45, C46, C47, C48), 127.7 (C15, C20), 127.5 (C13, C17, C18, C22), 121.7 (C5), 115.5 (C1), 114.4 (C4), 78.4 (C32), 70.1 (C9, C11), 51.9 (C24), 28.1 (C38, C39, C40), 26.0 (C41), 11.7 (C42); HRAMS (ESI − QTOF) calculated for $C_{39}H_{43}NO_8 + Na^+$: 676.2886, found 676.2885.

[(*S*)-3-[3,4-Di(benzyloxy)phenyl]-2-[*tert*-butyl(oxycarbonylamino)]propionoxy]methyl 2-furoate (6g)

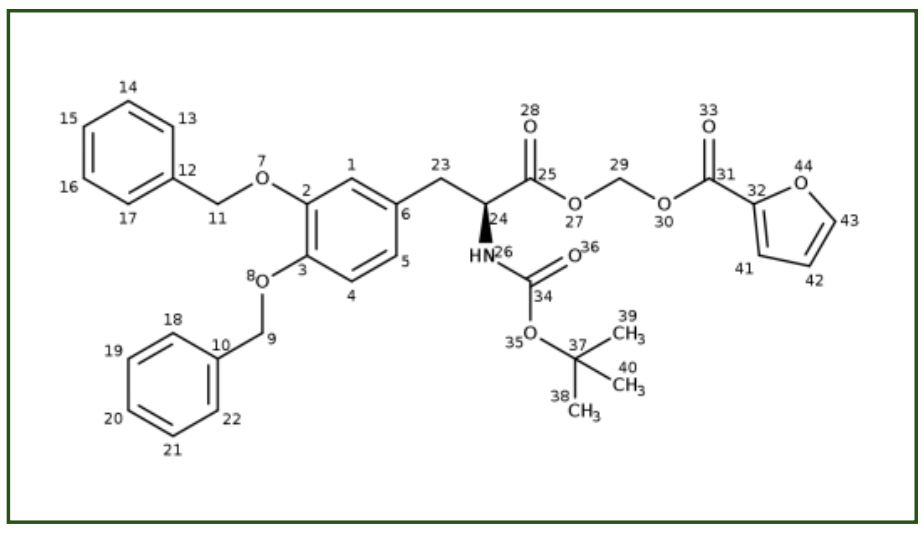

Using general synthetic procedure B, 0.161 g (1 mmol) of **5g** were used as educt. Flash chromatography is employed as the purification method. In this case, the product is additionally recrystallized from ethyl acetate/hexane. Yield: 0.251 g of a colorless solid (0.42 mmol, 42%); m.p. =90.1 °C; TLC: R_F = 0.34 in eluent I; ^{1}H NMR ($(CD_3)_2SO$): 8.05 (d, 1H, C(41)H, ^3J = 1.7 Hz), 7.45 − 7.28 (m, 12H, C13, C14, C15, C16, C17, C18, C19, C20, C21, C22, N(26)H, C(43)H), 7.03 (m, 1H, C(1)H), 6.88 (m, 1H, C(4)H), 6.74 (m, 2H, C(5)H, C(42)H), 5.90 (dd, 2H, C(29)H, ^2J = 3.6 Hz; 1.8 Hz), 5.06 (s, 4H, C(9)H, C(11)H), 4.19 (m, 1H, C(24)H), 2.83 (m, 2H, C(23)H), 1.29 (s, 9H, C(38)H, C(39)H, C(40)H); ^{13}C NMR ($(CD_3)_2SO$): 171.0 (C25), 156.2 (C31), 148.7 (C43), 148.1 (C34), 147.0 (C10, C12), 142.6 (C32), 137.3 (C2, C3), 130.1 (C6), 128.3 (C14, C16, C19, C21), 127.7 (C15, C20), 127.5 (C13, C17, C18, C22), 121.8 (C5), 120.1 (C41), 115.6 (C1), 114.3 (C4), 112.6 (C42), 79.4 (C29), 78.4 (C37), 70.1 (C9, C11), 55.2 (C24), 33.5 (C23), 28.0 (C38, C39, C40); HRAMS (ESI − QTOF) calculated for $C_{34}H_{35}NO_9 + Na^+$: 624.2210, found 624.2208

(2-Phenoxybenzoyloxy)methyl (2*S*)-3-[3,4-di(benzyloxy)phenyl]-2-[*tert*-butyl(oxycarbonylamino)]-propionate (6h)

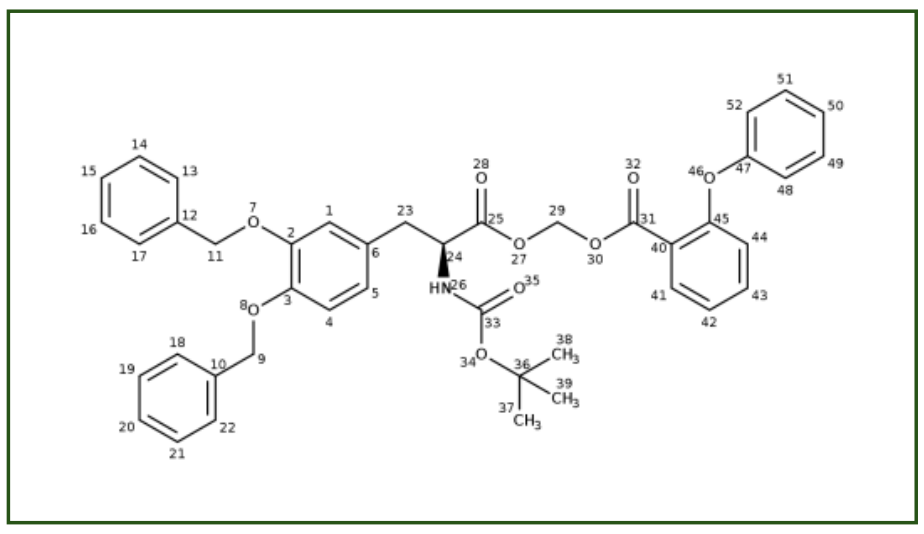

Using general synthetic procedure B, 0.263 g (1 mmol) of **5h** were used as educt. Flash chromatography is employed as the purification method. In this case, the product is additionally recrystallized from ethyl acetate/hexane. Yield: 0.508 g of a colorless solid (0.72 mmol, 72%); m.p. = 98.1 °C; TLC: R_F = 0.49 in eluent III; ^{1}H NMR ($(CD_3)_2SO$): 7.86 (d, 1H, C(41)H, ^3J = 7.3 Hz), 7.63 (t, 1H, C(43)H, ^3J = 7.7), 7.45 − 7.27 (m, 14H, C(13)H, C(14)H, C(15)H, C(16)H, C(17)H, C(18)H, C(19)H, C(20)H, C(21)H, C(22)H, N(26)H, C(44)H, C(49)H, C(51)H), 7.10 (t, 1H, C(42)H, ^3J = 7.4 Hz), 7.01 (m, 2H, C(1)H, C(50)H), 6.93 (d, 2H, C(48)H, C(52)H, ^3J = 7.9 Hz), 6,89 (d, 1H, C(4)H, ^3J = 7.2 Hz), 6.72 (d, 1H, C(5)H, ^3J = 7.6 Hz), 5.86 (dd, 2H, C(29)H, ^2J = 4.5 Hz; 3.1 Hz), 5.06 (s, 4H, C(9)H, C(11)H), 4.17 (m, 1H, C(24)H), 2.79 (m, 2H, C(23)H), 1.30 (s, 9H, C(37)H, C(38)H, C(39)H); ^{13}C NMR ($(CD_3)_2SO$): 171.1 (C25), 162.9 (C31), 156.8 (C47), 155.9 (C45), 148.0 (C33), 147.0 (C10, C12), 137.3 (C2, C3), 134.9 (C41), 131.7 (C43), 130.1 (C6), 130.0 (C49, C51), 128.3 (C14, C16, C19, C21), 127.7 (C15, C20), 127.5 (C13, C17, C18, C22), 123.8 (C50), 123.4 (C44), 121.8 (C5), 121.3 (C40), 120.5 (C42), 118.1 (C48, C52), 115.6 (C1), 114.3 (C4), 79.7 (C29), 78.4 (C36), 70.1 (C9, C11), 55.2 (C24), 28.1 (C37, C38, C39); HRAMS (ESI − QTOF) calculated for $C_{42}H_{41}NO_9 + Na^+$: 726.2679, found 726.2687.

(4-Phenoxybenzoyloxy)methyl (2*S*)-3-[3,4-bis(benzyloxy)phenyl]-2-[*tert*-butyl(oxycarbonyl-amino)]propionate (6i)

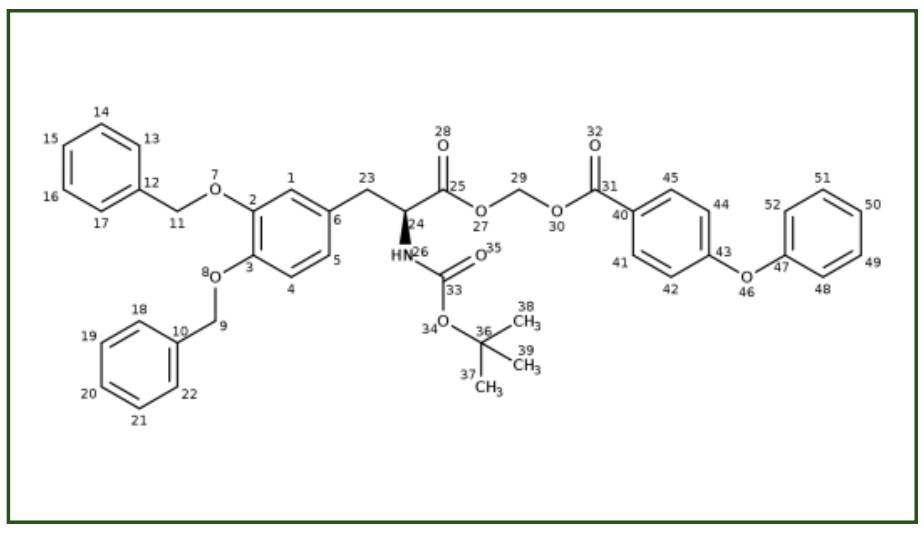

Using general synthetic procedure B, 0.263 g (1 mmol) of **5i** were used as educt. Flash chromatography is employed as the purification method. In this case, the product is additionally recrystallized from ethyl acetate/hexane. Yield: 0.497 g of a colorless solid (0.71 mmol, 71%); m.p. = 80.8 °C; TLC: R_F = 0.70 in eluent III; ^{1}H NMR ($(CD_3)_2SO$): 7.97 (m, 2H, C(41)H, C(45)H), 7.48 − 7.24 (m, 14H, C(13)H, C(14)H, C(15)H, C(16)H, C(17)H, C(18)H, C(19)H, C(20)H, C(21)H, C(22)H, N(26)H, C(41)H, C(42)H, C(44)H, C(45)H, C(48)H, C(49)H, C(50)H, C(51)H, C(52)H), 7.12 (m, 2H, C(49)H, C(51)H), 7.06 (m, 2H, C(48)H, C(52)H), 7.03 (m, 1H, C(1)H), 6.86 (m, 1H, C(4)H), 6.72 (m, 1H, C(5)H), 5.94 (dd, 2H, C(29)H, ^2J = 4.0 Hz; 2.8 Hz), 5.04 (s, 4H, C(9)H, C(11)H), 4.20 (m, 1H, C(24)H), 2.85 (m, 2H, C(23)H), 1.29 (s, 9H, C(37)H, C(38)H, C(39)H); ^{13}C NMR ($(CD_3)_2SO$): 171.1 (C25), 163.4 (C31), 162.0 (C43), 154.7 (C47), 148.1 (C33), 147.0 (C10, C12), 137.3 (C2, C3), 132.1 (C41, C45), 130.4 (C42, C44), 130.1 (C6), 128.3 (C14, C16, C19, C21), 127.7 (C15, C20), 127.5 (C13, C17, C18, C22), 124.9 (C49, C51), 122.7 (C40), 121.8 (C5), 120.1 (C50), 117.3 (C48, C52), 115.6 (C1), 114.3 (C4), 79.8 (C29), 78.4 (C36), 70.1 (C9, C11), 55.3 (C24), 35.6 (C23), 28.0 (C37, C38, C39); HRAMS (ESI − QTOF) calculated for $C_{42}H_{41}NO_9 + Na^+$: 726.2679, found 726.2687.

[2-(4-Isobutylphenyl)propionoxy]methyl (2*S*)-3-[3,4-bis(benzyloxy)phenyl]-2-[*tert*-butyl(oxycarbonylamino)]propionate (6j)

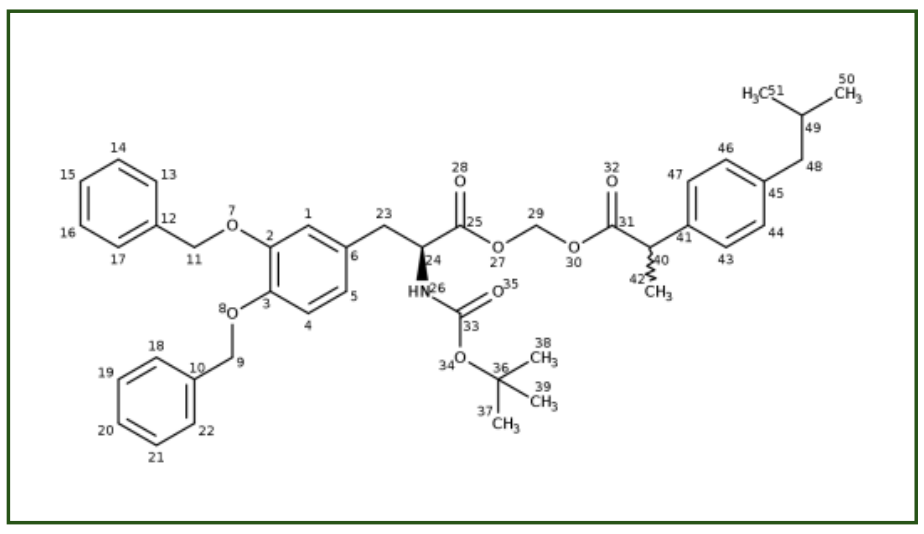

Using general synthetic procedure B, 0.255 g (1 mmol) of **5j** were used as educt. Flash chromatography is employed as the purification method. Yield: 0.477 g of a colorless solid (0.69 mmol, 69%); m.p. = 106.5 °C; TLC: R_F = 0.28 in eluent II; ^{1}H NMR ($(CD_3)_2SO$): 7.47 – 7.29 (m, 11H, C(13)H, C(14)H, C(15)H, C(16)H, C(17)H, C(18)H, C(19)H, C(20)H, C(21)H, C(22)H, N(26)H), 7.16 (m, 2H, C(43)H, C(47)H), 7.07 (t, 2H, C(44)H, C(46)H, ^3J = 7.8 Hz), 6.99 (m, 1H, C(1)H), 6.94 (d, 1H, C(4)H, ^3J = 8.2 Hz), 6.71 (d, 1H, C(5)H, ^3J = 8.2 Hz), 5.71 (m, 2H, C(29)H), 5.08 (s, 4H, C(9)H, C(11)H), 4.11 (m, 1H, C(24)H), 3.80 (m, 1H, C(40)H), 2.68 (m, 2H, C(23)H), 2.33 (m, 2H, C(48)H), 1.73 (m, 1H, C(49)H), 1.37 (m, 3H, C(42)H), 1.32 (s, 9H, C(37)H, C(38)H, C(39)H), 0.80 (m, 6H, C(50)H, C(51)H); ^{13}C NMR ($(CD_3)_2SO$): 172.7 (C25, C31), 148.0 (C33), 147.0 (C10, C12), 140.0 (C45), 137.3 (C2, C3), 137.0 (C41), 130.1 (C6), 129.1 (C43, C47), 128.3 (C14, C16, C19, C21), 127.7 (C15, C20), 127.5 (C13, C17, C18, C22), 127.1 (C44, C46), 121.8 (C5), 115.6 (C1), 114.4 (C4), 100.5 (C29), 78.4 (C36), 72.7 (C24), 70.2 (C9, C11), 44.1 (C48), 43.8 (C40), 30.8 (C23), 29.4 (C49), 28.1 (C38, C38, C39), 22.1 (C50, C51), 18.3 (C42); HRAMS (ESI – QTOF) calculated for $C_{42}H_{41}NO_9 + Na^+$: 718.3356, found 718.3355.

[(2*S*)-2-Acetylamino-3-phenylpropionoxy]methyl (2*S*)-3-[3,4-di(benzyloxy)phenyl]-2-[*tert*-butyl-(oxycarbonylamino)]propionate (6k)

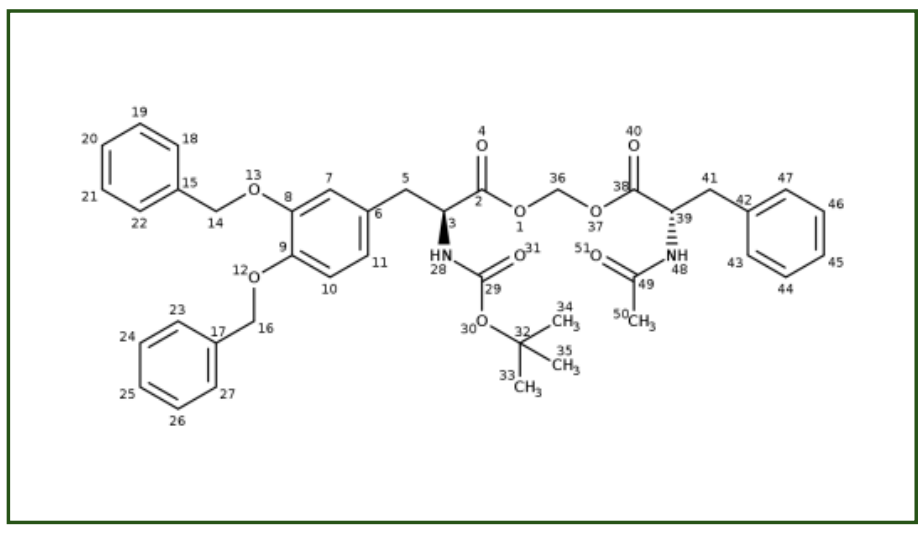

Using general synthetic procedure B, 0.256 g (1 mmol) of **5k** were used as educt. DCVC is employed as the purification method. Yield: 0.136 g of a colorless resin (0.20 mmol, 20%); TLC: R_F = 0.20 in eluent IV; ^{1}H NMR ($(CD_3)_2SO$): 7.46 – 7.18 (m, 17H, C(18)H, C(19)H, C(20)H, C(21)H, C(22)H, C(23)H, C(24)H, C(25)H, C(26)H, C(27)H, N(28)H, C(43)H, C(44)H, C(45)H, C(46)H, C(47)H, N(48)H), 7.04 (s, 1H, C(1)H), 6.96 (d, 1H, C(4)H, ^3J = 8.3), 6.77 (d, 1H, C(5)H, ^3J = 8.2 Hz), 5.75 (m, 2H, C(36)H), 4.45 (m, 1H, C(39)H), 4.18 (m, 1H, C(3)H), 2.88 (m, 4H, C(5)H, C(41)H), 1.78 (s, 3H, C(50)H), 1.31 (s, 9H, C(33)H, C(34)H, C(35)H); ^{13}C NMR ($(CD_3)_2SO$): 170.5 (C2, C38), 169.5 (C49), 137.3 (C8, C9), 137.0 (C42), 130.2 (C6), 129.0 (C43, C47), 128.4 (C19, C21, C24, C26), 128.3 (C44, C46), 127.7 (C20, C25), 127.5 (C18, C22, C23, C27), 126.6 (C45), 121.8 (C11), 115.6 (C7), 114.4 (C10), 80.6 (C36), 78.4 (C32), 70.2 (C14, C16), 55.1 (C3), 53.5 (C39), 36.2 (C5), 36.0 (C41), 28.1 (C33, C34, C35), 22.1 (C50); HRAMS (ESI – QTOF) calculated for $C_{40}H_{44}N_2O_9 + Na^+$: 719.2945, found 719.2952.

[(9*Z*)-8-Heptadecenylcarbonyloxy]methyl (2*S*)-3-[3,4-di(benzyloxy)phenyl]-2-[*tert*-butyl(oxycarbonylamino)]propionate (6l)

Using general synthetic procedure B, 0.331 g (1 mmol) of **5l** were used as educt. Flash chromatography is employed as the purification method. In this case, the product is additionally recrystallized from toluene/hexane. Yield: 0.426 g of a colorless resin (0.55 mmol, 55%); m.p. = 84.3 °C; TLC: R_F = 0.30 in eluent I; ^{1}H NMR ($(CD_3)_2SO$): 7.47 – 7.29 (m, 11H, C(13)H, C(14)H, C(15)H, C(16)H, C(17)H, C(18)H, C(19)H,

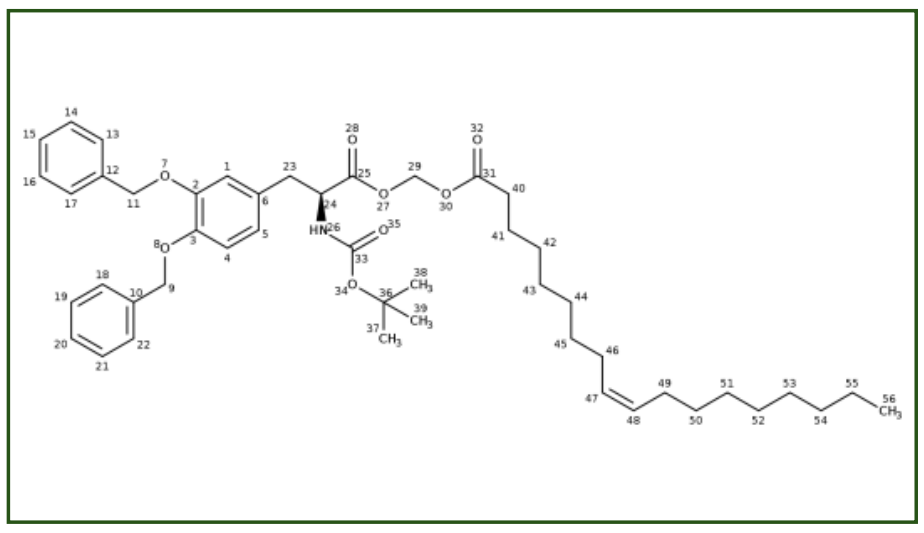

C(20)H, C(21)H, C(22)H, N(26)H), 7.03 (s, 1H, C(1)H), 6.95 (m, 1H, C(4)H), 6.76 (m, 1H, C(5)H), 5.70 (dd, 2H, C(29)H, ^2J = 22.5 Hz; 5.9 Hz), 5.31 (m, 2H, C(47)H, C(48)H), 5.08 (s, 4H, C(9)H, C(11)H), 4.17 (m, 1H, C(24)H), 2.83 (m, 2H, C(23)H), 2.32 (t, 2H, C(40)H, ^3J = 7.1 Hz), 1.96 (m, 4H, C(46)H, C(49)H), 1.50 (m, 2H, C(41)H), 1.34 (s, 9H, C(37)H, C(38)H, C(39)H), 1.23 (m, 20H, C(42)H, C(43)H, C(44)H, C(45)H, C(50)H, C(51)H, C(52)H, C(53)H, C(54)H, C(55)H), 0.85 (t, 3H, C(56)H, ^3J = 6.1 Hz); ^{13}C NMR ($(CD_3)_2SO$): 171.6 (C31), 171.1 (C25), 148.1 (C33), 147.0 (C10, C12), 137.3 (C2, C3), 130.1 (C6), 129.6 (C47, C48), 128.3 (C14, C16, C19, C21), 127.7 (C15, C20), 127.4 (C13, C17, C18, C22), 121.8 (C5), 115.6 (C1), 114.4 (C4), 79.1 (C29), 78.3 (C36), 70.2 (C9, C11), 55.2 (C24), 35.6 (C23), 33.1 (C40), 31.2 (C54), 29.0 – 28.1 (C37, C38, C39, C42, C43, C44, C45, C50, C51, C52, C53), 26.5 (C47, C48), 24.1 (C41), 22.0 (C55), 13.9 (C56); HRAMS (ESI – QTOF) calculated for $C_{47}H_{65}NO_8$ + Na^+: 794.4608, found 794.4598.

[(2-Acetyloxybenzoyl)oxy]methyl (2*S*)-3-[3,4-di(benzyloxy)phenyl]-2-[*tert*-butyl(oxycarbonyl-amino)]propionate (6m)

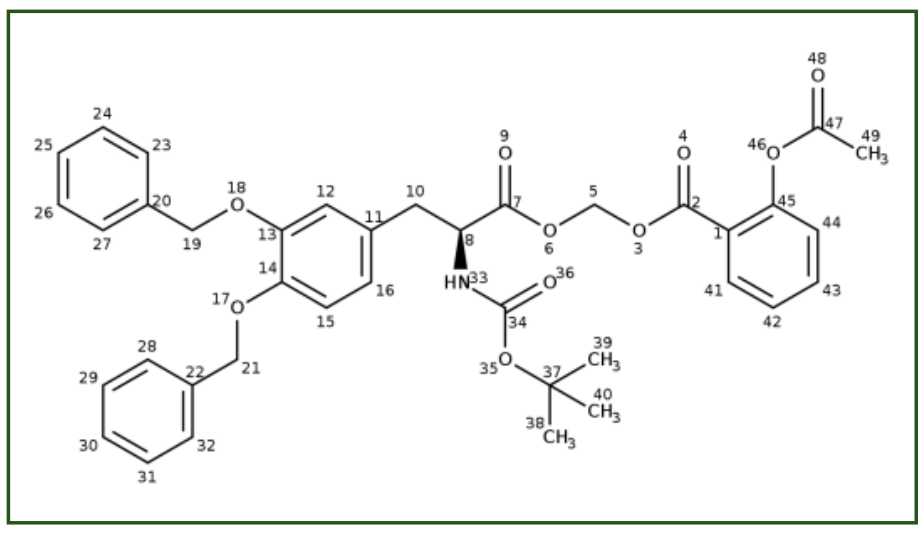

Using general synthetic procedure B, 0.229 g (1 mmol) of **5m** were used as educt. Flash chromatography is employed as the purification method. Yield: 0.380 g of a colorless solid (0.57 mmol, 57%); m.p. = 89.4 °C; TLC: R_F = 0.31 in eluent I; ^{1}H NMR ($(CD_3)_2SO$): 7.94 (dd, 1H, C(41)H, ^3J = 7.8 Hz; 1.7 Hz), 7.73 (m, 1H, C(43)H), 7.45 – 7.26 (m, 13H, C(23)H, C(24)H, C(25)H, C(26)H, C(27)H, C(28)H, C(29)H, C(30)H, C(31)H, C(32)H, N(33)H, C(42)H, C(44)H), 7.04 (m, 1H, C(12)H), 6.89 (m, 1H, C(15)H), 6.75 (m, 1H, C(16)H), 5.91 (dd, 2H, C(5)H, ^2J = 3.9 Hz; 2.6 Hz), 5.06 (s, 4H, C(19)H, C(21)H), 4.21 (m, 1H, C(8)H), 2.85 (m, 2H, C(10)H), 2.28 (s, 3H, C(49)H), 1.30 (s, 3H, C(49)H); ^{13}C NMR ($(CD_3)_2SO$): 171.2 (C7), 169.1 (C47), 162.5 (C2), 150.4 (C45), 148.0 (C34), 147.0 (C20, C22), 137.3 (C13, C14), 135.1 (C41), 131.5 (C43), 130.1 (C11), 128.3 (C24, C26, C29, C31), 127.7 (C25, C30), 127.5 (C23, C27, C28, C32), 126.4 (C44), 124.2 (C42), 121.8 (C1, C16), 115.6 (C12), 114.3 (C15), 79.8 (C5), 78.4 (C37), 70.1 (C19, C21), 55.2 (C8), 35.5 (C10), 28.0 (C38, C39, C40), 20.7 (C49); HRAMS (ESI – QTOF) calculated for $C_{38}H_{39}NO_{10}$ + Na^+: 692.2472, found 692.2483.

[1-Adamantanylcarbonyloxy]methyl (2*S*)-3-[3,4-di(benzyloxy)phenyl]-2-[*tert*-butyl(oxycarbonyl-amino)]propionate (6n)

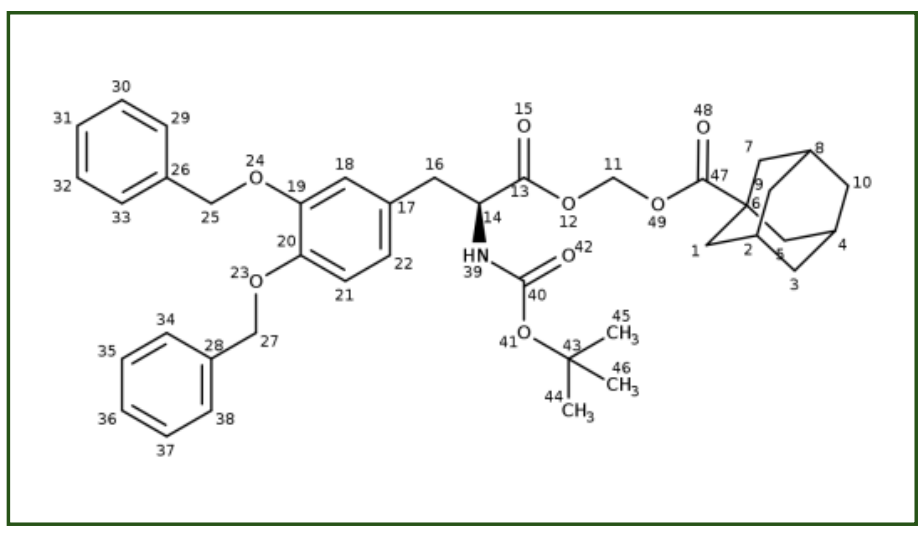

Using general synthetic procedure B, 0.229 g (1 mmol) of **5n** were used as educt. Flash chromatography is employed as the purification method. Yield: 0.500 g of a colorless resin (0.75 mmol, 75%); TLC: R_F = 0.78 in eluent IV. ^{1}H NMR ($(CD_3)_2SO$): 7.47 – 7.18 (m, 11H, C(29)H, C(30)H, C(31)H, C(32)H, C(32)H, C(33)H, C(34)H, C(35)H, C(36)H, C(37)H, C(38)H, N(39)H), 7.03 (m, 1H, C(18)H), 6.95 (m, 1H, C(21)H), 6.76 (m, 1H, C(22)H), 5.69 (dd, 2H, C(11)H, ^2J = 3.6 Hz; 2.0 Hz), 5.08 (s, 4H, C(25)H, C(27)H), 4.16 (m, 1H, C(14)H), 2.81 (m, 2H,

C(16)H), 1.95 (m, 3H, C(2)H, C(4)H, C(8)), 1.79 (m, 6H, C(1)H, C(5)H, C(7)H), 1.65 (m, 6H, C(3)H, C(9)H, C(19)H), 1.33 (s, 9H, C(44)H, C(45)H, C(46)H); ^{13}C NMR (($CD_3)_2SO$): 175.0 (C47), 171.2 (C13), 148.1 (C40), 147.0 (C26, C28), 137.3 (C19, C20), 130.1 (C17), 128.3 (C30, C32, C35, C37), 127.7 (C31, C36), 127.5 (C29, C33, C34, C38), 121.8 (C22), 115.5 (C18), 114.3 (C21), 79.2 (C11), 78.4 (C43), 70.1 (C25, C27), 55.2 (C14), 40.0 (C6), 37.9 (C1, C5, C7), 35.8 (C3, C9, C10), 28.1 (C44, C45, C46), 27.1 (C2, C4, C8); HRAMS (ESI – QTOF) calculated for $C_{40}H_{47}NO_8$ + Na^+: 692.3199, found 692.3192.

Acetoxymethyl (2*S*)-3-[3,4-bis(benzyloxy)phenyl]-2-[*tert*-butyl(oxycarbonylamino)]propionate (6o)

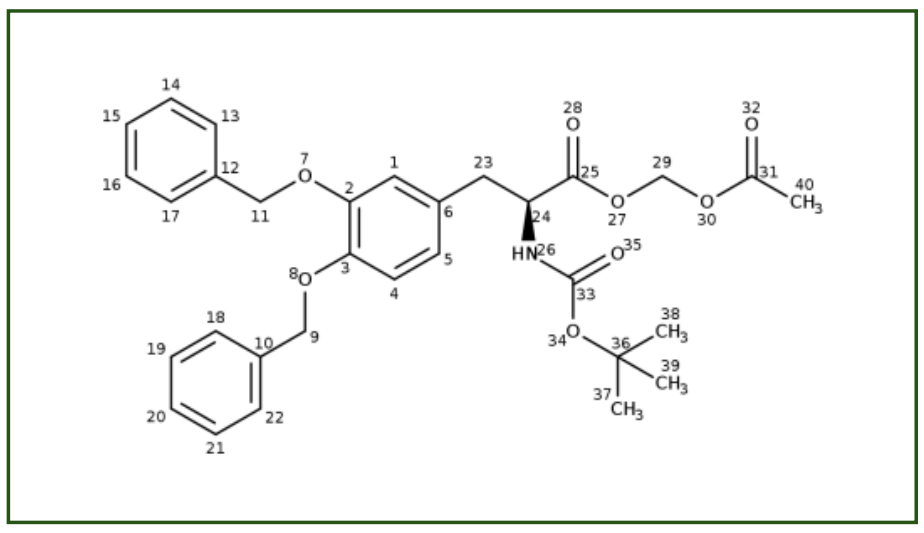

Using general synthetic procedure B, 0.098 mL (1 mmol) of **5o** were used as educt. Flash chromatography is employed as the purification method. Yield: 0.349 g of a colorless resin (0.63 mmol, 63%); m.p. = 106.5 °C; TLC: R_F = 0.65 in eluent III; ^{1}H NMR (($CD_3)_2SO$): 7.48 – 6.72 (m, 14H, C(1)H, C(4)H, C(5)H, C(13)H, C(14)H, C(15)H, C(16)H, C(17)H, C(18)H, C(19)H, C(20)H, C(21)H, C(22)H, N(26)H), 5.67 (dd, 2H, C(29)H, ^2J = 8.0 Hz; 12.0 Hz), 5.08 (s, 4H, C(9)H, C(11)H), 4.17 (m, 1H, C(24)H), 2.83 (m, 2H, C(23)H), 2.05 (s, 3H, C(40)H), 1.33 (s, 9H, C(37)H, C(38)H, C(39)H); ^{13}C NMR (($CD_3)_2SO$): 171.0 (C25), 169.1 (C31), 155.3 (C33), 148.2 – 114.2 (C1, C2, C3, C4, C5, C6, C10, C12, C13, C14, C15, C16, C17, C18, C19, C20, C21, C22), 78.8 (C36), 70.2 (C9, C11), 55.2 (C24), 35.6 (C23), 28.1 (C37, C38, C39), 20.4 (C40); HRAMS (ESI – QTOF) calculated for $C_{31}H_{35}NO_8$ + Na^+: 572.2260, found 572.2264.

3.4 Deproteced Acylals

Benzoyloxymethyl (2*S*)-2-amino-3-(3,4-dihydroxyphenyl)propionate hydrochloride (7a)

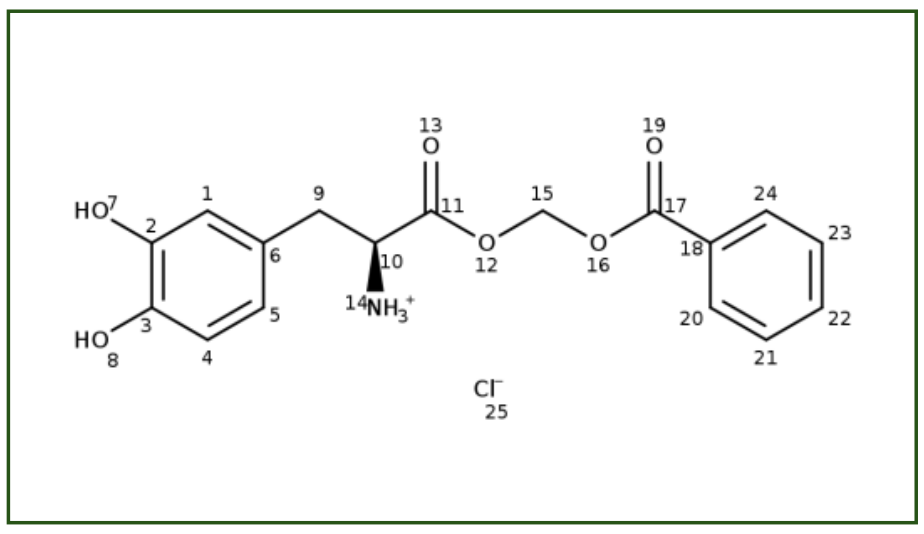

Using general synthetic procedure C, 0.100 g (0,16 mmol) of **6a** were used as educt. Yield: 0.053 g of a grayish solid (0,15 mmol, 90%); ^{1}H NMR (($CD_3)_2SO$): 8.93 (s, 2H, O(7)H, O(8)H), 8.58 (s, 3H, N(14)H), 8.00 (m, 2H, C(20)H, C(24)H), 7.74 (m, 1H, C(22)H), 7.59 (m, 2H, C(21)H, C(23)H), 6.66 (d, 1H, C(1)H, ^3J = 2.1 Hz), 6.54 (d, 1H, C(4)H, ^3J = 8.0 Hz), 6.42 (dd, 1H, C(5)H, ^3J = 8.0 Hz; 2.1 Hz), 6.04 (d, 2H, C(15)H, ^2J = 2.0 Hz), 4.28 (t, 1H, C(10)H, ^3J = 8.0 Hz), 2.97 (m, 2H, C(9)H); ^{13}C NMR (($CD_3)_2SO$): 168.2 (C11), 164.2 (C17), 145.3 (C2), 144.7 (C3), 134.2 (C22), 129.6 (C20, C24), 129.0 (C21, C23), 128.2 (C18), 124.5 (C6), 120.1 (C5), 116.9 (C1), 115.7 (C4), 80.5 (C15), 53.2 (C10), 35.0 (C9); HRAMS (ESI – QTOF) calculated for $C_{17}H_{17}NO_6$ + H^+: 332.1134, found 332.1134

(5-Phenylvaleroxy)methyl (2*S*)-2-amino-3-(3,4-dihydroxyphenyl)propionate hydrochloride (7b)

Using general synthetic procedure C, 0.447 g (0.67 mmol) of **6b** were used as educt. Unlike usual, however, the product of the final reaction step precipitated out of the reaction mixture as a pale gray solid. Instead

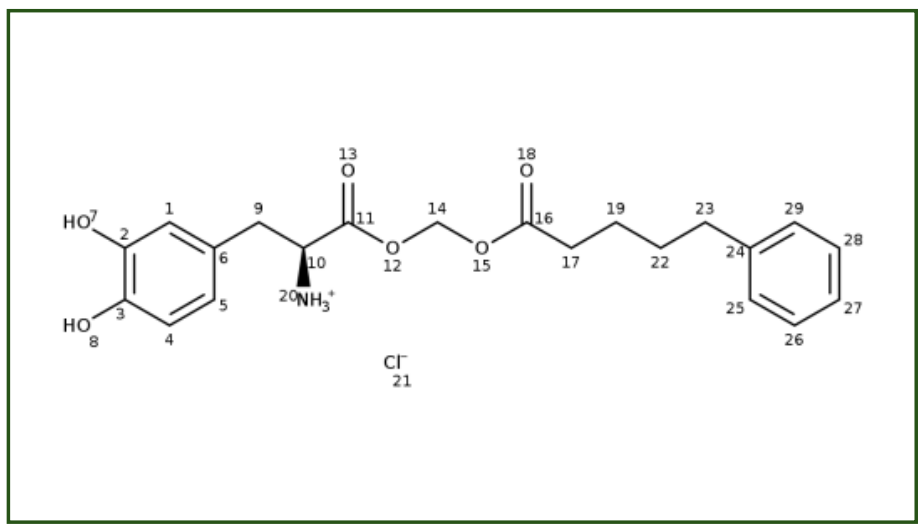

of evaporating the solvent under reduced pressure, this solid is filtered off and then washed with ethyl acetate. Yield: 0.105 g of a grayish solid (0.25 mmol, 37%); ^{1}H NMR ($(CD_3)_2SO$): 8,99 (s, 1H, O(8)H), 8,93 (s, 1H, O(7)H) 8,63 (s, 3H, N(20)H), 7,22 (m, 5H, C(25)H, C(26), C(27)H, C(28)H, C(29)H), 6,67 (d, 1H, C(1)H, ^{3}J = 8,0Hz), 6,62 (d, 1H, C(4)H, ^{3}J = 1,6Hz), 6,45 (d, 1H, C(5)H, ^{3}J = 7,8Hz), 5,76 (q, 2H, C(14)H, ^{2}J = 7,9Hz), 4,20 (t, 1H, C(10)H, ^{3}J = 6,2Hz), 2,96 (m, 2H, C(9)H), 2,50 (m, 2H, C(23)H), 2,41 (t, 2H, C(17)H, ^{3}J = 6,4Hz), 1,57 (m, 4H, C(19)H, C(22)H). ^{13}C NMR ($(CD_3)_2SO$): 171.5 (C16), 168.2 (C11), 145.3 (C2), 144.7 (C3), 141.9 (C24), 128.2 (C25, C26, C28, C29), 125.7 (C27), 124.5 (C6), 120.2 (C5), 116.9 (C1), 115.7 (C4), 79.6 (C14), 53.2 (C10), 35.0 (C9), 34.7 (C23), 32.8 (C17), 30.1 (C19), 23.6 (C22); HRAMS (ESI − QTOF) calculated for $C_{21}H_{25}NO_6 + H^+$: 388.1760, found 388.1765.

(3-Phenylpropionoxy)methyl (2*S*)-2-amino-3-(3,4-dihydroxyphenyl)propionate hydrochloride (7c)

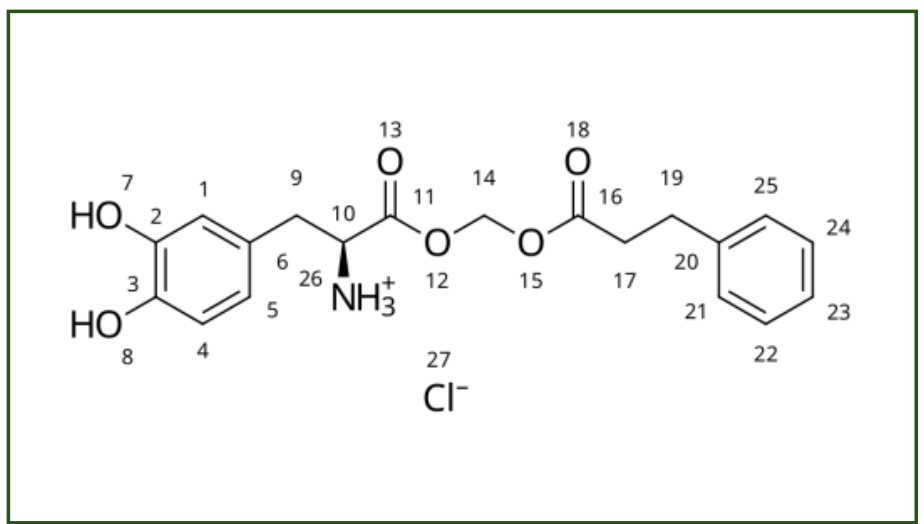

Using general synthetic procedure C, 0.48 g (0.75 mmol) of **6c** were used as educt. Yield: 0.308 g of a light grey solid (0.78 mmol, quantitative); ^{1}H NMR ($(CD_3)_2SO$): 8.96 (s, 2H, O(7)H, O(8)H), 8.63 (s, 3H, N(14)H), 8.00 (m, 2H, C(20)H, C(24)H), 7.74 (m, 1H, C(22)H), 7.59 (m, 2H, C(21)H, C(23)H), 7.30 − 7.17 (m, 5H, C(21)H, C(22)H, C(23)H, C(24)H, C(25)H), 6.62 (d, 1H, C(1)H, ^{3}J = 2.0 Hz), 6.46 (d, 1H, C(4)H, ^{3}J = 7.9 Hz), 6.46 (dd, 1H, C(5)H, ^{3}J = 8.0 Hz; 2.1 Hz), 5.77 (q, 2H, C(14)H, ^{2}J = 5.8 Hz), 4.20 (t, 1H, C(10)H, 6.44 Hz), 2.95 (m, 2H, C(9)H), 2.87 (t, 2H, C(17)H, ^{3}J = 7.4 Hz), 2.72 (t, 2H, C(19)H, ^{3}J = 7.7 Hz); ^{13}C NMR ($(CD_3)_2SO$): 170.9 (C11), 168.2 (C16), 145.3 (C2), 144.7 (C3), 140.1 (C23), 128.64 (C21, C25), 129.0 (C22, C24), 126.1 (C20), 124.5 (C6), 120.2 (C5), 116.9 (C1), 115.7 (C4), 79.6 (C14), 53.2 (C10), 35.0 (C9), 34.5 (C17), 29.7 (C19); HRAMS (ESI − QTOF) calculated for $C_{19}H_{21}NO_6 + Na^+$: 382.1267, found 382.1272.

[(2*S*)-2-Amino-3-(3,4-dihydroxyphenyl)propanoyl]oxymethyl 2,2-dimethylpropionate hydrochloride (7d)

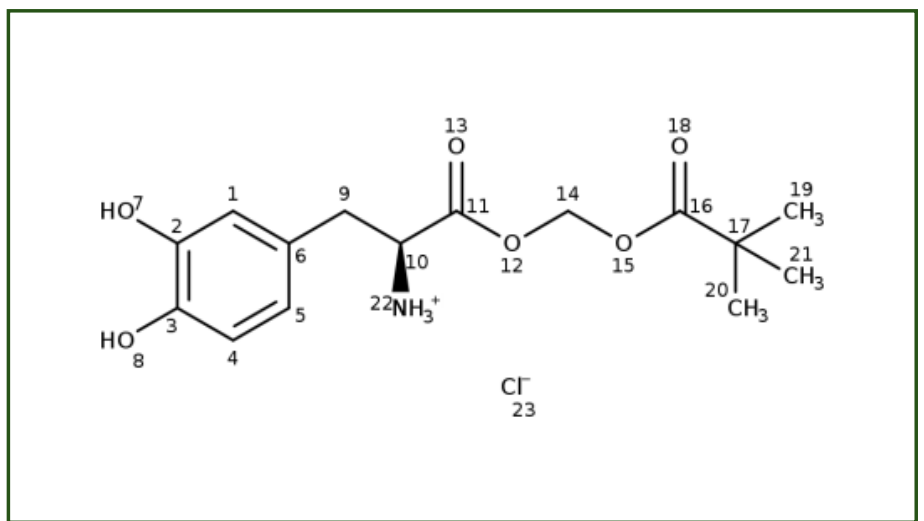

Using general synthetic procedure C, 0.327 g (0.55 mmol) **6d** were used as educt. Yield: 0.177 g of a yellowish solid (0.51 mmol, 92%); ^{1}H NMR ($(CD_3)_2SO$): 8.97 (s, 1H, O(8)H), 8.91 (s, 1H, O(7)H), 8.61 (s, 3H, N(22)H), 6.66 (m, 1H, C(1)H), 6.64 (m, 1H, C(4)H), 6.46 (m, 1H, C(5)H), 5.78 (m, 2H, C(14)H), 4.24 (t, 1H, C(10)H, ^{3}J = 8.0 Hz), 2.95 (m, 2H, C(9)H), 1.16 (s, 9H, C(19)H, C(20)H, C(21)H); ^{13}C NMR ($(CD_3)_2SO$): 176.0 (C16), 168.2 (C11), 145.3 (C2), 144.7 (C3), 124.5 (C6), 120.1 (C5), 116.9 (C1), 115.7 (C4), 80.2 (C14), 53.1 (C10), 38.2 (C17), 35.0 (C9), 26.5 (C19, C20, C21); HRAMS (ESI − QTOF) calculated for $C_{15}H_{21}NO_6 + H^+$: 312.1447, found 312.1442.

[(2*S*)-2-Amino-3-(3,4-dihydroxyphenyl)propanoyl]oxymethyl 4-phenoxybutanoate hydrochloride (7e)

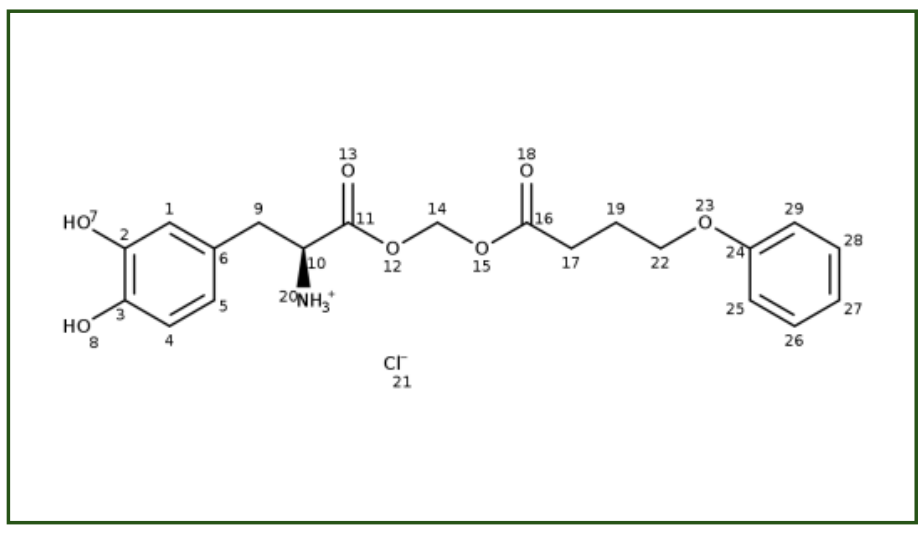

Using general synthetic procedure C, 0.318 g (0.47 mmol) **6e** were used as educt. Unlike usual, however, the product of the final reaction step precipitated out of the reaction mixture as a pale gray solid after adding a few drops of hexane. Instead of evaporating the solvent under reduced pressure, this solid is filtered off and then washed with ethyl acetate. Yield: 0.148 g of a grayish solid (0.35 mmol, 73%); ^{1}H NMR ((CD_3)$_2$SO): 8.99 (s, 1H, O(8)H), 8.94 (s, 1H, O(7)H), 8.61 (s, 3H, N(20)H), 7.28 (t, 2H, C(26)H, C(28), ^3J = 8.0 Hz), 6.92 (m, 3H, C(25)H, C(27)H, C(29)H), 6.68 (d, 1H, C(1)H, ^3J = 8.0 Hz), 6.62 (m, 1H, C(4)H), 6.46 (m, 1H, C(5)H), 5.77 (dd, 2H, C(14)H, ^2J = 3.0 Hz; 1.6 Hz), 4.21 (m, 1H, C(10)H), 4.00 (t, 2H, C(17)H, ^3J = 6.4 Hz), 2.97 (m, 2H, C(9)H), 2.55 (t, 2H, C(22)H, ^3J = 4.0 Hz), 1.98 (m, 2H, C(19)H); ^{13}C NMR ((CD_3)$_2$SO): 171.3 (C16), 168.2 (C11), 158.3 (C24), 145.3 (C2), 144.7 (C3), 129.5 (C26, C28), 124.5 (C6), 120.5 (C27), 120.2 (C5), 116.9 (C1), 115.7 (C4), 114.4 (C25, C29), 79.6 (C14), 66.1 (C17), 53.2 (C10), 35.0 (C9), 29.8 (C22), 23.8 (C19); HRAMS (ESI – QTOF) calculated for $C_{20}H_{23}NO_7$ + H^+: 390.1553, found 390.1554.

[(2*S*)-2-Amino-3-(3,4-dihydroxyphenyl)propanoyl]oxymethyl 2-phenylbutanoate hydrochloride (7f)

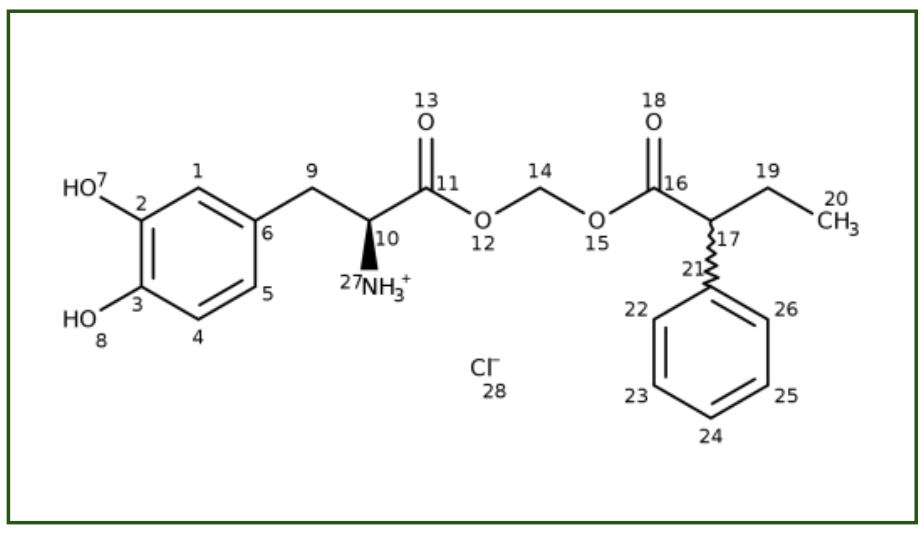

Using general synthetic procedure C, 0.248 g (0.38 mmol) **6f** were used as educt. Yield: 0.157 g of a brownish solid (0.38 mmol, 98%); ^{1}H NMR ((CD_3)$_2$SO): 8.98 (s, 1H, O(8)H), 8.92 (s, 1H, O(7)H), 8.51 (s, 3H, N(22)H), 7.39 – 7.21 (m, 5H, C(22)H, C(23)H, C(24)H, C(25)H, C(26)H), 6.67 (m, 1H, C(1)H), 6.61 (m, 1H, C(4)H), 6.41 (m, 1H, C(5)H), 5.79 (m, 2H, C(14)H), 4.20 (m, 1H, C(10)H), 3.62 (t, 1H, C(17)H, ^3J = 8.0 Hz), 2.87 (m, 2H, C(9)H), 1.99 (m, 2H, C(19)H), 0.82 (t, 3H, C(20)H, ^3J = 8.0 Hz); ^{13}C NMR ((CD_3)$_2$SO): 172.0 (C16), 168.1 (C11), 145.3 (C2), 144.7 (C3), 138.0 (C21), 128.6 (C22, C26), 127.9 (C23, C25), 127.3 (C24), 124.4 (C6), 120.2 (C5), 116.8 (C1), 115.7 (C4), 80.0 (C14), 53.1 (C10), 51.7 (C17), 35.0 (C9), 26.0 (C19), 11.7 (C20); HRAMS (ESI – QTOF) calculated for $C_{20}H_{23}NO_6$ + H^+: 374.1604, found 374.1598.

[(2*S*)-2-Amino-3-(3,4-dihydroxyphenyl)propanoyl]oxymethyl 2-furoate hydrochloride (7g)

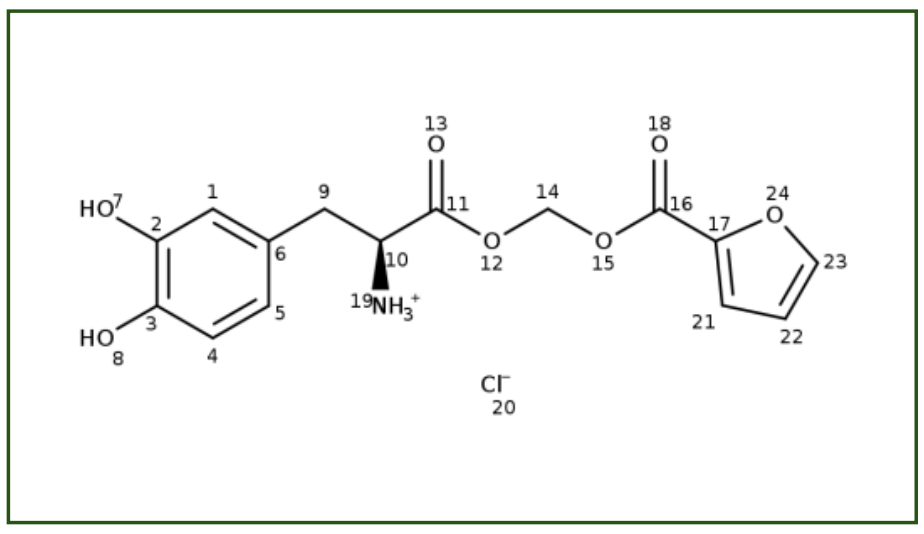

Using general synthetic procedure C, 0.200 g (0.33 mmol) of **6g** were used as educt. Yield: 0.114 g of a brown resin (0.32 mmol, 96%); ^{1}H NMR ((CD_3)$_2$SO): 8.91 (m, 2H, O(7)H, O(8)H), 8.54 (s, 3H, N(19)H), 8.08 (m, 1H, C(21)H), 7.45 (m, 1H, C(23)H), 6.77 (m, 1H, C(22)H), 6.63 (m, 1H, C(1)H), 6.55 (m, 1H, C(4)H), 6.44 (m, 1H, C(5)H), 5.81 (m, 2H, C(14)H), 4.28 (m, 1H, C(10)H), 2.94 (m, 2H, C(9)H); ^{13}C NMR ((CD_3)$_2$SO): 168.9 (C11), 156.3 (C16), 149.4 (C23), 145.3 (C2), 144.7 (C3), 142.4 (C17),

124.5 (C6), 120.4 (C17), 124.5 (C6), 120.4 (C21), 120.2 (C5), 116.9 (C1), 115.7 (C4), 113.1 (C22), 79.9 (C14), 53.4 (C10), 35.2 (C9); HRAMS (ESI – QTOF) calculated for $C_{15}H_{15}NO_7$ + Na^+: 344.0746, found 344.0741.

(2-Phenoxybenzoyloxy)methyl (2*S*)-2-amino-3-(3,4-dihydroxyphenyl)propionate hydrochloride (7h)

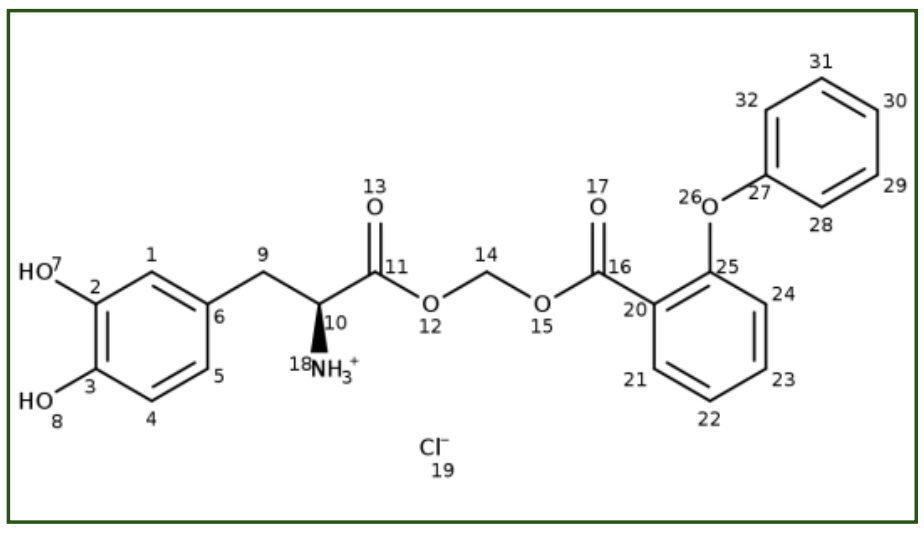

Using general synthetic procedure C, 0.484 g (0.69 mmol) **6h** were used as educt. Yield: 0.307 g of a colorless solid (0,67 mmol, 97%); ^{1}H NMR ($(CD_3)_2SO$): 8.94 (s, 2H, O(7)H, O(8)H), 8.58 (s, 3H, N(18)H), 7.90 (m, 1H, C(21)H), 7.67 (m, 1H, C(23)H), 7.38 (m, 2H, C(29)H, C(31)H), 7.33 (m, 1H, C(24)H), 7.13 (m, 1H, C(22)H), 7.04 (m, 1H, C(30)H), 6.95 (m, 2H, C(28)H, C(32)H), 6.65 (m, 1H, C(1)H), 6.58 (m, 1H, C(4)H), 6.43 (m, 1H, C(5)H), 5.95 (dd, 2H, C(14)H, ^{2}J = 6.0 Hz; 2.0 Hz), 4.24 (t, 1H, C(10)H, ^{3}J = 6.2 Hz), 2.94 (m, 2H, C(9)H); ^{13}C NMR ($(CD_3)_2SO$): 168.1 (C11), 162.6 (C16), 156.8 (C27), 156.1 (C25), 145.3 (C2), 144.7 (C3), 135.2 (C21), 131.9 (C23), 130.1 (C29, C31), 124.5 (C6), 123.9 (C30), 123.4 (C24), 120.8 (C20), 120.6 (C22), 120.2 (C5), 118.1 (C28, C32), 116.9 (C1), 115.7 (C4), 80.2 (C14), 53.2 (C10), 34.9 (C9); HRAMS (ESI – QTOF) calculated for $C_{23}H_{21}NO_7$ + H^+: 424.1396, found 424.1397.

(4-Phenoxybenzoyloxy)methyl (2*S*)-2-amino-3-(3,4-dihydroxyphenyl)propionate hydrochloride (7i)

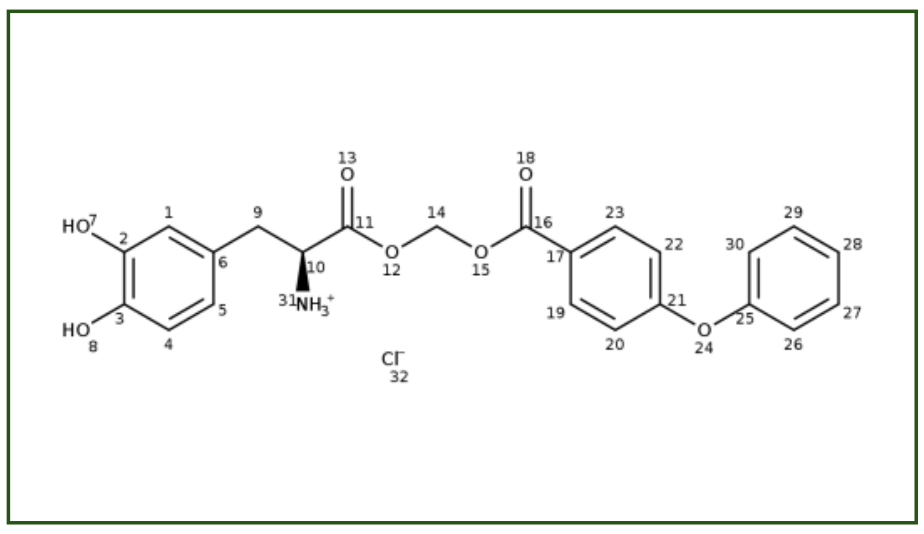

Using general synthetic procedure C, 0.284 g (0.40 mmol) of **6i** were used as product. Yield: 0.176 g of a brownish solid (0.38 mmol, 95%); ^{1}H NMR ($(CD_3)_2SO$): 8.93 (s, 2H, O(7)H, O(H)8), 8.60 (s, 3H, N(31)H), 8.00 (m, 2H, C(19)H, C(23)H), 7.48 (m, 2H, C(20)H, C(22)H), 7.27 (m, 1H, C(28)H), 7.15 (m, 2H, C(27)H, C(29)H), 7.11 (m, 2H, C(26)H, C(30)H), 6.65 (m, 1H, C(1)H), 6.55 (m, 1H, C(4)H), 6.42 (m, 1H, C(5)H), 6.02 (dd, 2H, C(14)H, ^{2}J = 3.6 Hz; 2.9 Hz), 4.27 (m, 1H, C(10)H), 2.98 (m, 2H, C(9)H); ^{13}C NMR ($(CD_3)_2SO$): 168.2 (C11), 163.6 (C16), 162.1 (C21), 154.7 (C25), 145.3 (C2), 144.7 (C3), 132.2 (C19, C23), 130.4 (C20, C22), 125.0 (C27, C29), 124.4 (C6), 122.4 (C17), 120.1 (C28), 120,1 (C5), 117.4 (C26, C30), 116.9 (C1), 115.7 (C4), 80.3 (C14), 53.2 (C10), 35.0 (C9); HRAMS (ESI – QTOF) calculated for $C_{23}H_{21}NO_7$ + H^+: 424.1396, found 424.1391.

[2-(4-Isobutylphenyl)propionoxy]methyl (2*S*)-2-amino-3-(3,4-dihydroxyphenyl)propionate hydrochloride (7j)

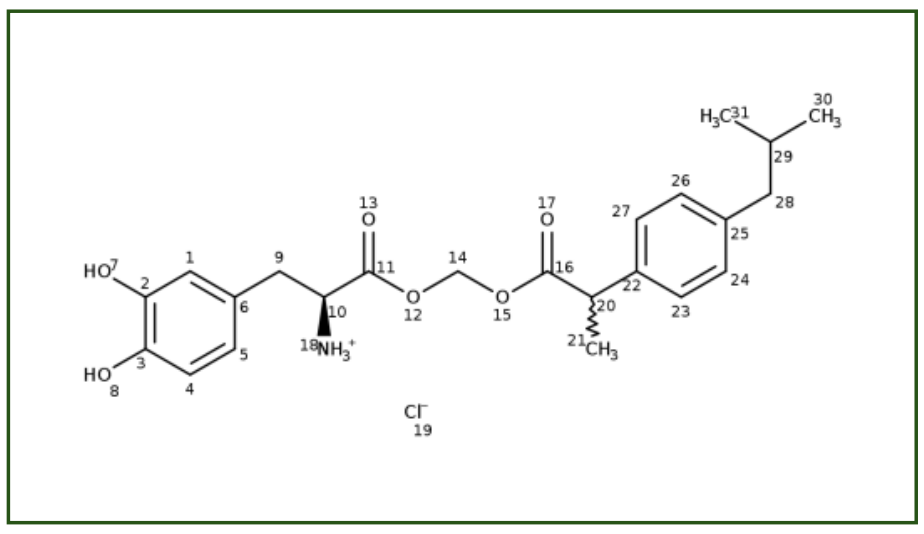

Using general synthetic procedure C, 0.450 g (0.65 mmol) of **6j** were used as educt. Yield: 0.289 g of a grayish solid (0.64 mmol, 98%); ^{1}H NMR ($(CD_3)_2SO$): 8.95 (s, 2H, O(7)H, O(8)H), 8.55 (s, 3H, N(18)H), 6.68 (d, 1H, C(1)H, ^3J = 4.0 Hz), 6.66 (d, 1H, C(4)H, ^3J = 4.0 Hz), 6.42 (m, 1H, C(5)H), 5.77 (m, 2H, C(14)H), 4.18 (m, 1H, C(10)H), 3.83 (m, 1H, C(20)H), 2.90 (m, 2H, C(9)H), 2.24 (m, 2H, C(28)H), 1.39 (m, 3H, C(21)H), 1.77 (m, 1H, C(29)H), 0.83 (m, 6H, C(30)H, C(31)H); ^{13}C NMR ($(CD_3)_2SO$): 172.6 (C16), 168.1 (C11), 145.3 (C2), 144.7 (C3), 140.1 (C25), 136.9 (C22), 129.2 (C23, C27), 127.1 (C24, C26), 124.5 (C6), 120.2 (C5), 116.8 (C1), (C4), 80.0 (C14), 53.1 (C10), 44.1 (C28), 43.8 (C20), 30.8 (C9), 29.5 (C29), 22.1 (C30, C31), 18.3 (C21).; HRAMS (ESI − QTOF) calculated for $C_{23}H_{29}NO_6$ + H^+: 416.2073, found 416.2071.

[(2*S*)-2-Amino-3-(3,4-dihydroxyphenyl)propanoyl]oxymethyl (2*S*)-2-acetylamino-3-phenylpropionate hydrochloride (7k)

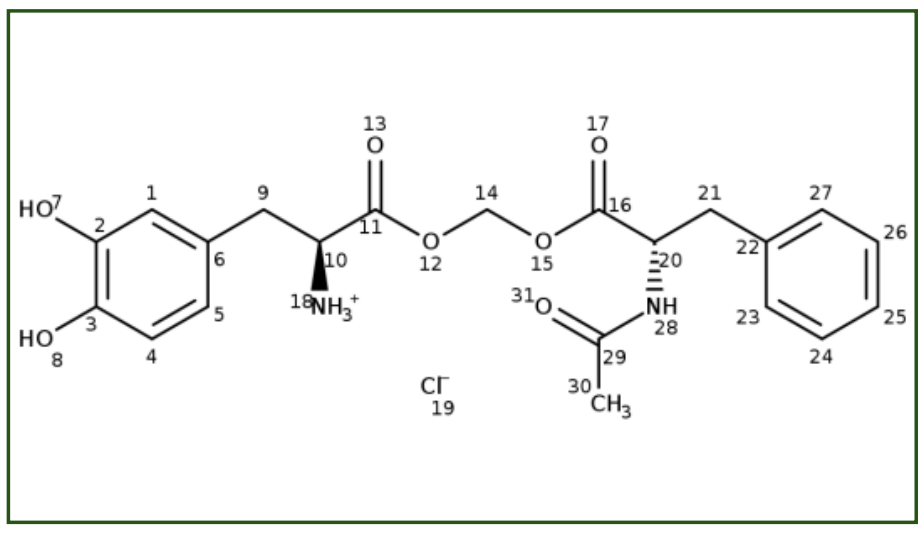

Using general synthetic procedure C, 0.224 g (0.32 mmol) of **6k** were used as educt. Yield: 0.154 g of a brown oil (0.37 mmol, quantitative); ^{1}H NMR ($(CD_3)_2SO$): 8.95 (s, 2H, O(7)H, O(8)H), 8.56 (s, 3H, N(18)H), 7.31 − 7.13 (m, 6H, C(23)H, C(24)H, C(25)H, C(26)H, C(27)H, N(28)H), 6.69 (d, 1H, C(1)H, ^3J = 8.0 Hz), 6.65 (d, 1H, C(4)H, ^3J = 2.0 Hz), 6.49 (dd, 1H, C(5), ^3J = 8.2 Hz; 2.0 Hz), 5.81 (q, 2H, C(14)H, ^2J = 5.9 Hz), 4.48 (m, 1H, C(20)H), 4.23 (m, 1H, C(10)H), 2.97 (m 4H, C(9)H, C(21)H), 1.80 (s, 3H, C(30)H); ^{13}C NMR ($(CD_3)_2SO$): 170.4 (C16), 169.7 (C29), 168.1 (C11), 145.3 (C2), 144.8 (C3), 137.0 (C22), 129.1 (C23, C27), 128.3 (C24, C26), 126.6 (C25), 125.5 (C6), 120.3 (C5), 116.9 (C1), 115.8 (C4), 80.2 (C14), 53.5 (C20), 53.2 (C10), 36.1 (C21), 35.0 (C9), 22.1 (C30); HRAMS (ESI − QTOF) calculated for $C_{21}H_{24}N_2O_7$ + Na^+: 439.1481, found 439.1476.

[(2*S*)-2-Amino-3-(3,4-dihydroxyphenyl)propanoyl]oxymethyl octadecanoate hydrochloride (7l)

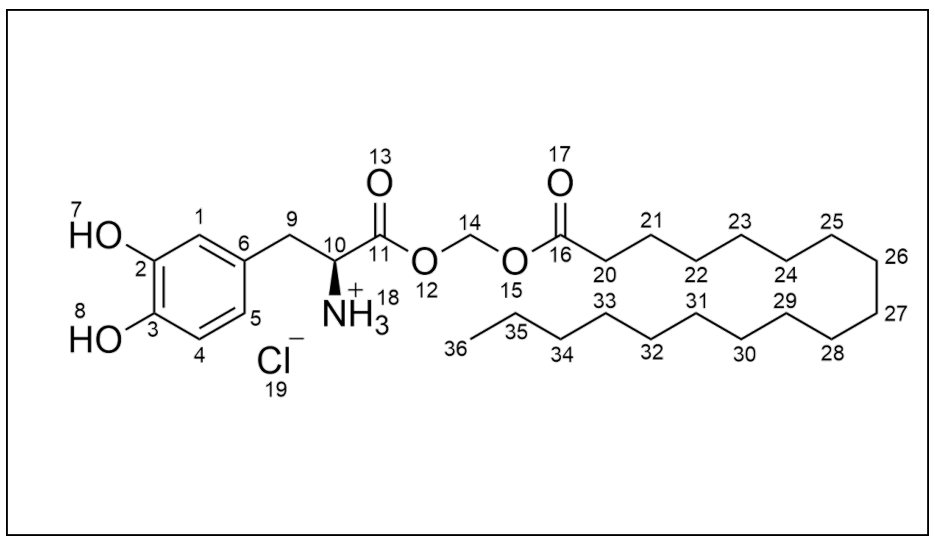

Using general synthetic procedure C, 0.334 g (0.41 mmol) of **6l** were used as educt. Yield: 0.192 g of a grayish solid (0.36 mmol, 88%); ^{1}H NMR ($(CD_3)_2SO$): 8.93 (s, 2H, O(7)H, O(8)H), 8.56 (s, 3H, N(18)H), 6.66 (m, 1H, C(1)H), 6.61 (m, 1H, C(4)H), 6.45 (m, 1H, C(5)H), 5.77 (q, 2H, C(14)H, ^2J = 6.1 Hz), 4.22 (t, 1H, C(10)H, ^3J = 6.2 Hz), 2.94 (m, 1H, C(10)H), 2.36 (t, 2H, C(20)H, ^3J = 7.3 Hz), 1.52 (m, 2H, C(21)H), 1.23 (m, 28H, C(22)H, C(23)H, C(24)H, C(25)H, C(26)H, C(27)H, C(28)H, C(29)H, C(30)H, C(31)H, C(32)H, C(33)H, C(34)H, C(35)H), 0.85 (t, 3H, C(36)H, ^3J = 6.5 Hz); ^{13}C NMR ($(CD_3)_2SO$): 171.6 (C16), 168.2 (C11), 145.3 (C2), 144.7 (C3), 124.4 (C6), 120.2 (C5), 116.8 (C1), 115.7 (C4), 79.6 (C14), 53.1 (C10), 35.0

(C9), 33.0 (C20), 31.3 (C34), 29.0 – 28.3 (C22, C23, C24, C25, C26, C27, C28, C29, C30, C31, C32, C33), 24.0 (C21), 22.1 (C35), 13.9 (C36); HRAMS (ESI – QTOF) calculated for $C_{28}H_{47}NO_6$ + H^+: 494.3482, found 494.3484.

[(2-Acetyloxybenzoyl)oxy]methyl (*S*)-2-amino-3-(3,4-dihydroxyphenyl)propionate hydrochloride (7m)

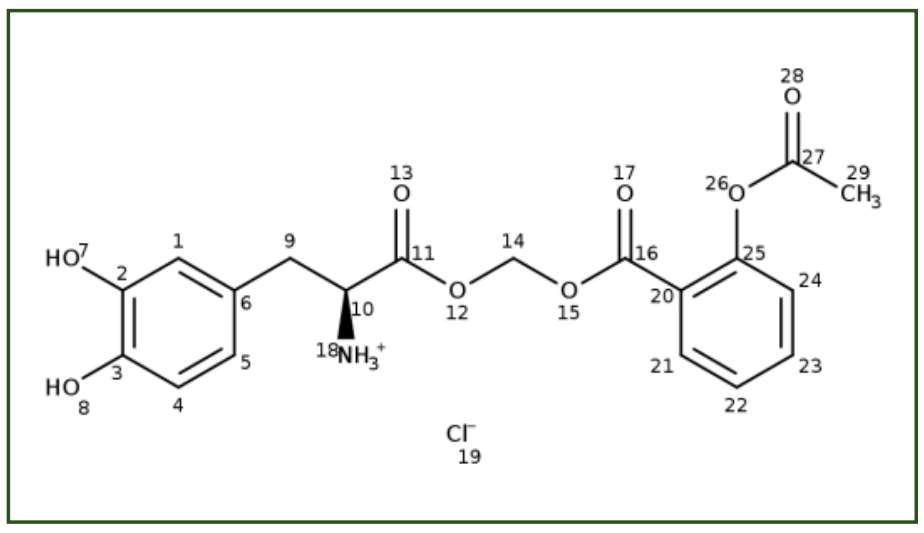

Using general synthetic procedure C, 0.380 g (0.57 mmol) of **6m** were used as educt. Yield: 0.230 g of a grayish solid (0.54 mmol, 95%); ^{1}H NMR ($(CD_3)_2SO$): 8.93 (s, 2H, O(7)H, O(8)H), 8.58 (s, 3H, N(18)H), 7.97 (dd, 1H, C(21)H, ^3J = 7.9 Hz), 7.77 (m, 1H, C(23)H), 7.48 (m, 1H, C(22)H), 7.30 (dd, 1H, C(24)H, ^3J = 8.1 Hz; 1.0 Hz), 6.66 (d, 1H, C(1)H, ^3J = 2.1 Hz), 6.59 (d, 1H, C(4)H, ^3J = 8.1 Hz), 6.44 (dd, 1H, C(5)H, ^3J = 8.1 Hz; 2.0 Hz), 5.99 (q, 2H, C(14)H, ^2J = 6.2 Hz), 4.28 (t, 1H, C(10)H, ^3J = 6.2 Hz), 2.98 (m, 2H, C(9)H), 2.30 (s, 3H, C(29)H); ^{13}C NMR ($(CD_3)_2SO$): 169.1 (C27), 168.2 (C11), 162.2 (C16), 150.6 (C25), 145.3 (C2), 144.7 (C3), 135.3 (C21), 131.6 (C23), 126.5 (C24), 124.5 (C6), 124.3 (C22), 121.4 (C20), 120.2 (C5), 116.9 (C1), 115.7 (C4), 80.3 (C14), 53.2 (C10), 35.0 (C9), 20.7 (C29); HRAMS (ESI – QTOF) calculated for $C_{19}H_{19}NO_8$ + H^+: 390.1189, found 390.1197.

[(2*S*)-2-Amino-3-(3,4-dihydroxyphenyl)propanoyl]oxymethyl 1-adamantanecarboxylate hydrochloride (7n)

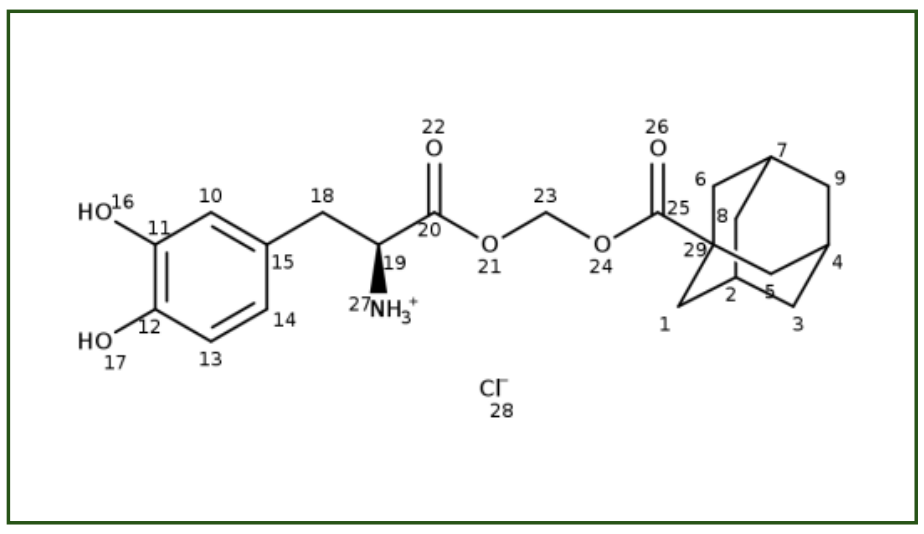

Using general synthetic procedure C, 0.262 g (0.39 mmol) of **6n** were used as educt. Yield: 0.174 g of a colorless solid (0.37 mmol, 96%); ^{1}H NMR ($(CD_3)_2SO$): 8.97 (s, 1H, O(17)H), 8.90 (s, 1H, O(16)H), 8.56 (s, 3H, N(27)H), 6.66 (m, 1H, C(10)H), 6.64 (m, 1H, C(13)H), 6.45 (m, 1H, C(14)H), 5.78 (m, 2H, C(23)H), 4.25 (t, 1H, C(19)H, ^3J = 8.0 Hz), 2,94 (m, 2H, C(18)H), 1.99 (m, 3H, C(2)H, C(4)H, C(7)H), 1.81 (d, 6H, C(1)H, C(5)H, C(6)H, ^3J = 2.8 Hz), 1.67 (m, 6H, C(3)H, C(8)H, C(9)H); ^{13}C NMR ($(CD_3)_2SO$): 174.9 (C25), 168.2 (C20), 145.3 (C11), 144.7 (C12), 124.4 (C15), 120.1 (C14), 116.9 (C10), 115.7 (C13), 80.0 (C23), 53.1 (C19), 40.0 (C29), 37.9 (C1, C5, C6), 35.7 (C3, C8, C9), 27.1 (C2, C4, C7); HRAMS (ESI – QTOF) calculated for $C_{21}H_{27}NO_6$ + H^+: 390.1917, found 390.1908.

3.4.1 Acetyloxymethyl (2*S*)-2-amino-3-(3,4-dihydroxyphenyl)propionate hydrochloride (7o)

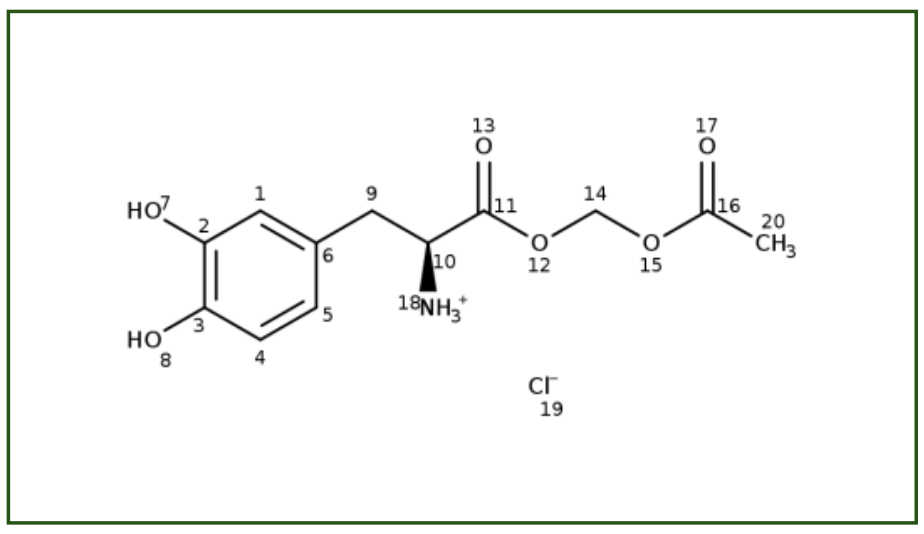

Using general synthetic procedure C, 0.400 g (0.73 mmol) of **6o** were used as educt. Yield: 0.192 g of a brownish resin (0.71 mmol, 97%); ^{1}H NMR ($(CD_3)_2SO$): 8.97 (s, 1H, O(8)H), 8.91 (s, 1H, O(7)H), 8.58 (s, 3H, N(22)H), 6.66 (d, 1H, C(1)H, ^{3}J = 8.0 Hz), 6.64 (d, 1H, C(4)H, ^{3}J = 2.1 Hz), 6.46 (dd, 1H, C(5)H, ^{3}J = 8.0 Hz), 5.79 (q, 2H, C(14)H, ^{3}J = 5.8 Hz), 4.25 (t, 1H, C(10)H, ^{3}J = 6.3 Hz), 2.95 (m, 2H, C(9)H), 1.16 (s, 3H, C(20)H); ^{13}C NMR ($(CD_3)_2SO$): 176.0 (C16), 168.2 (C11), 145.3 (C2), 144.7 (C3), 124.5 (C6), 120.1 (C5), 116.9 (C1), 115.7 (C4), 80.3 (C14), 53.2 (C10), 38.2 (C20), 35.0 (C9); HRAMS (ESI − QTOF) calculated for $C_{12}H_{15}NO_6 + H^+$: 270.0978, found 270.0973.

4 ^{1}H and ^{13}C NMR spectra

Compound 2

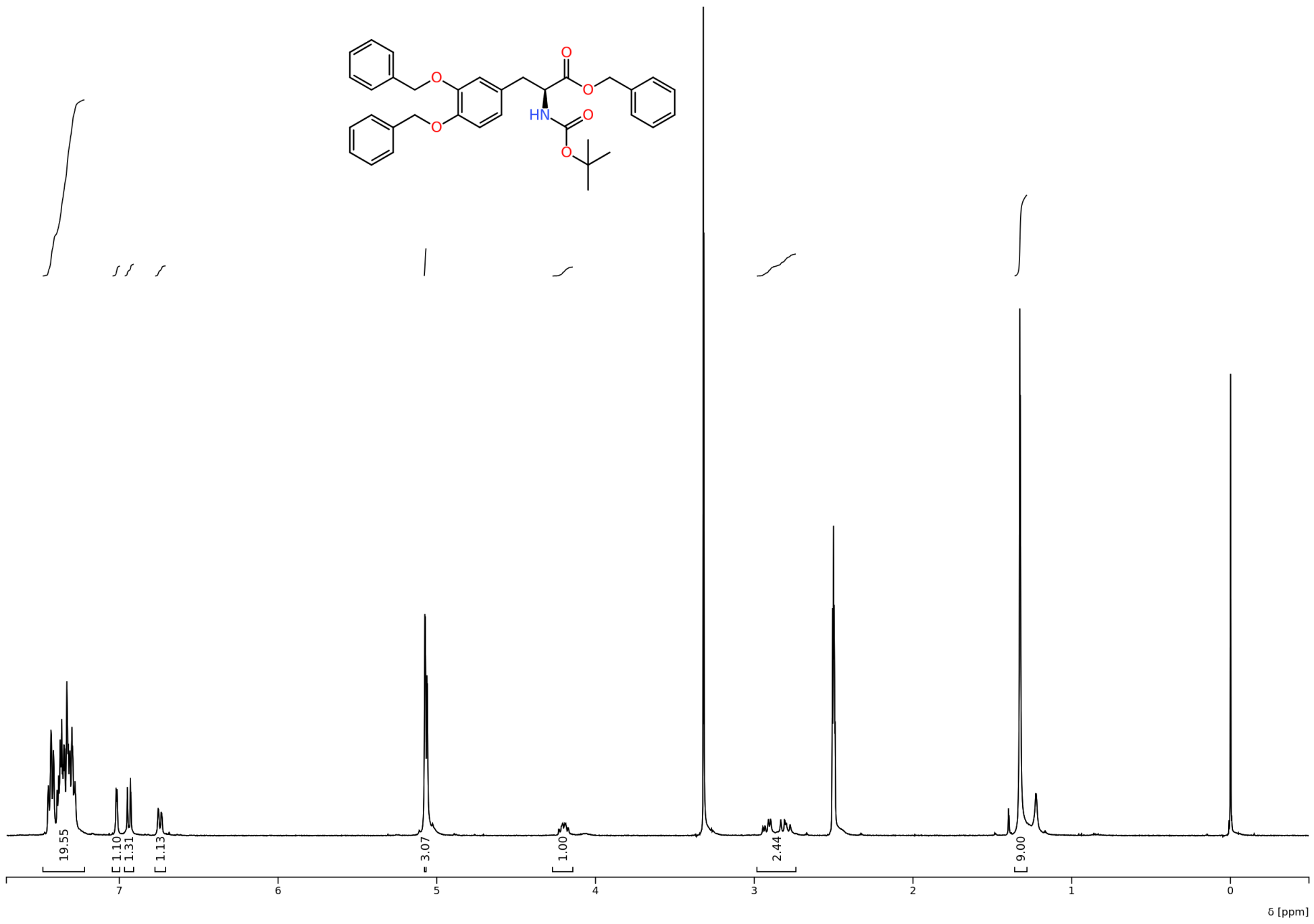

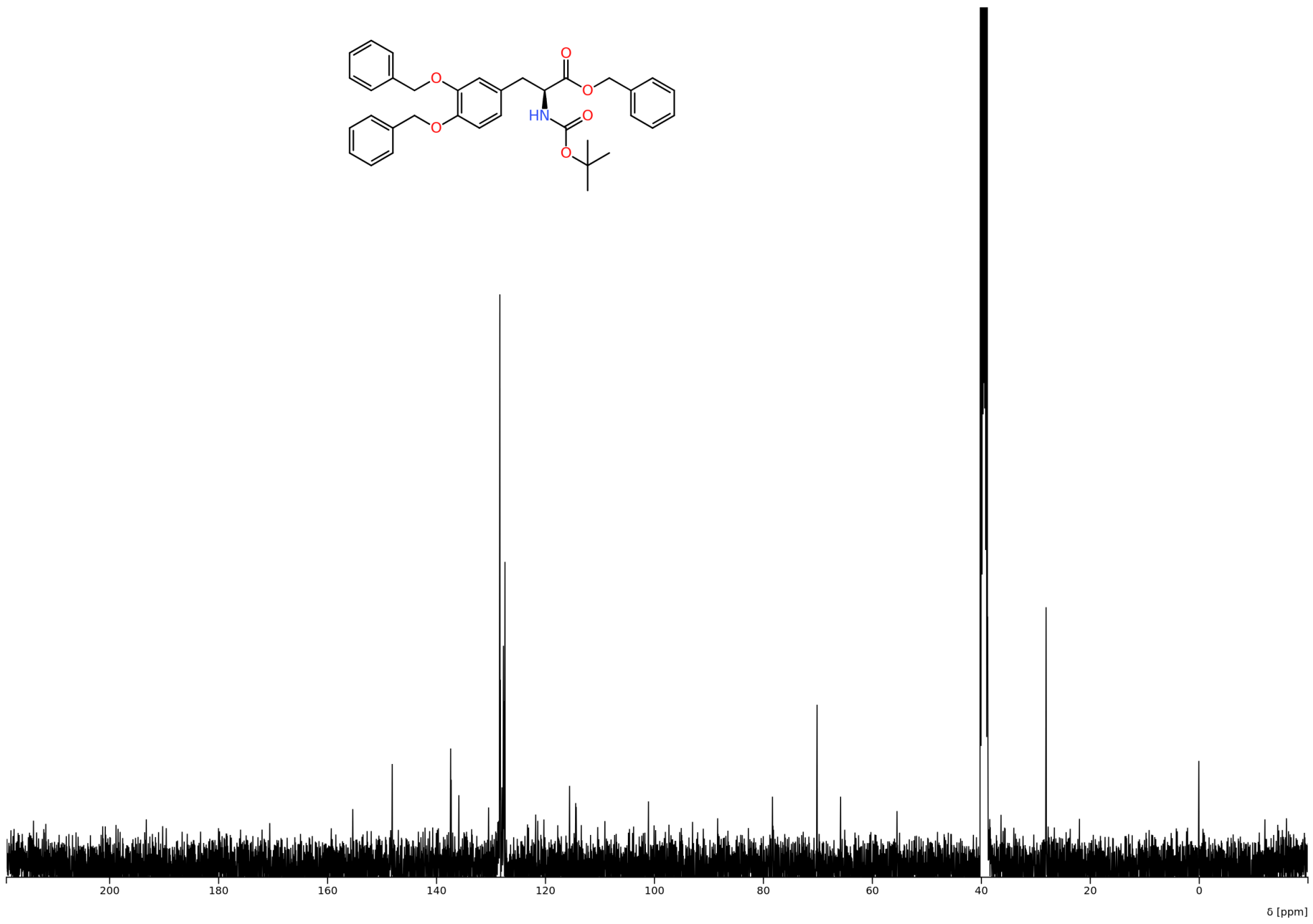

Compound 3

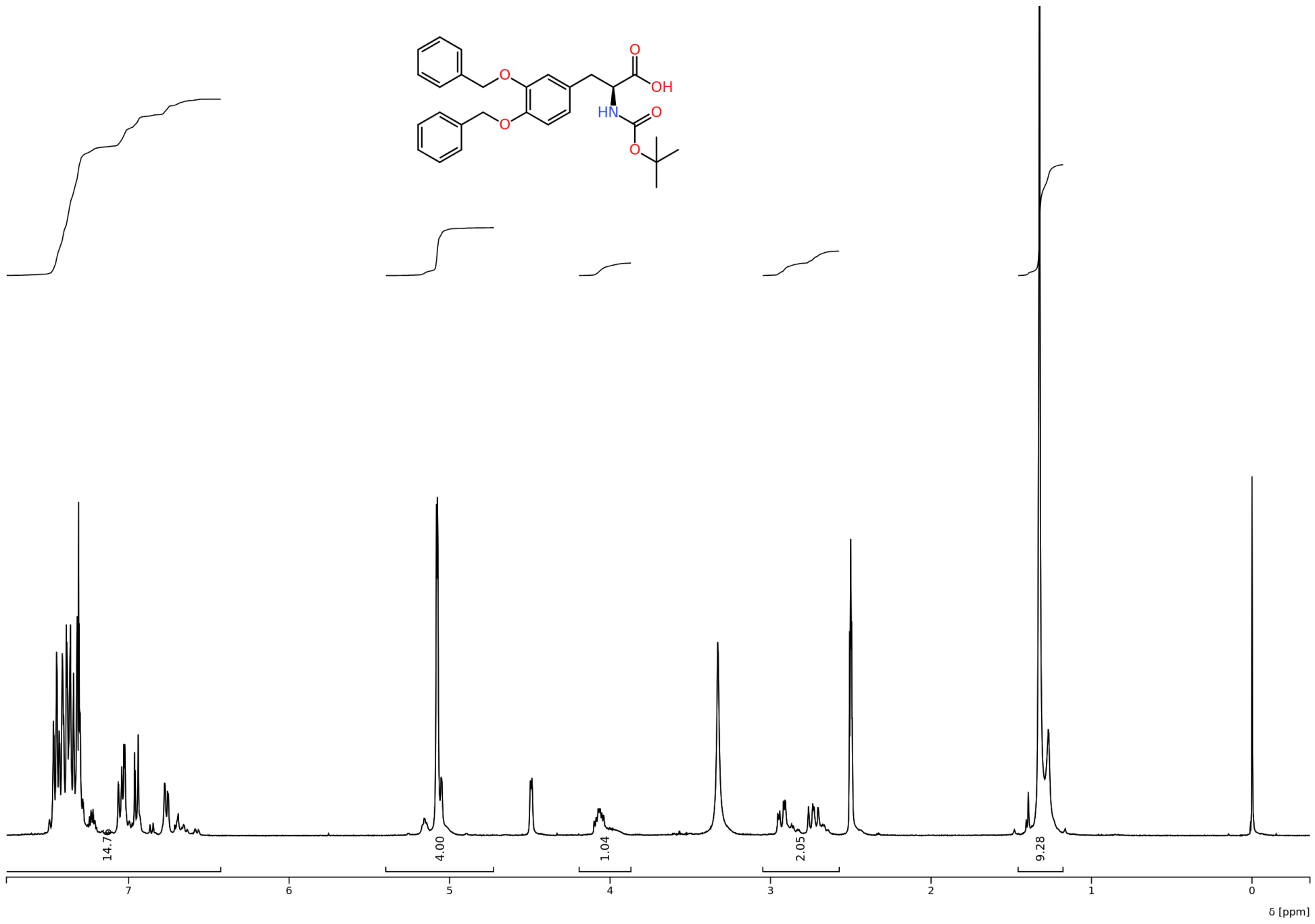
O
OH
HN
O
O
O
O
4.00
1.04
2.05
9.28
7
6
5
4
3
2
1
0
δ [ppm]

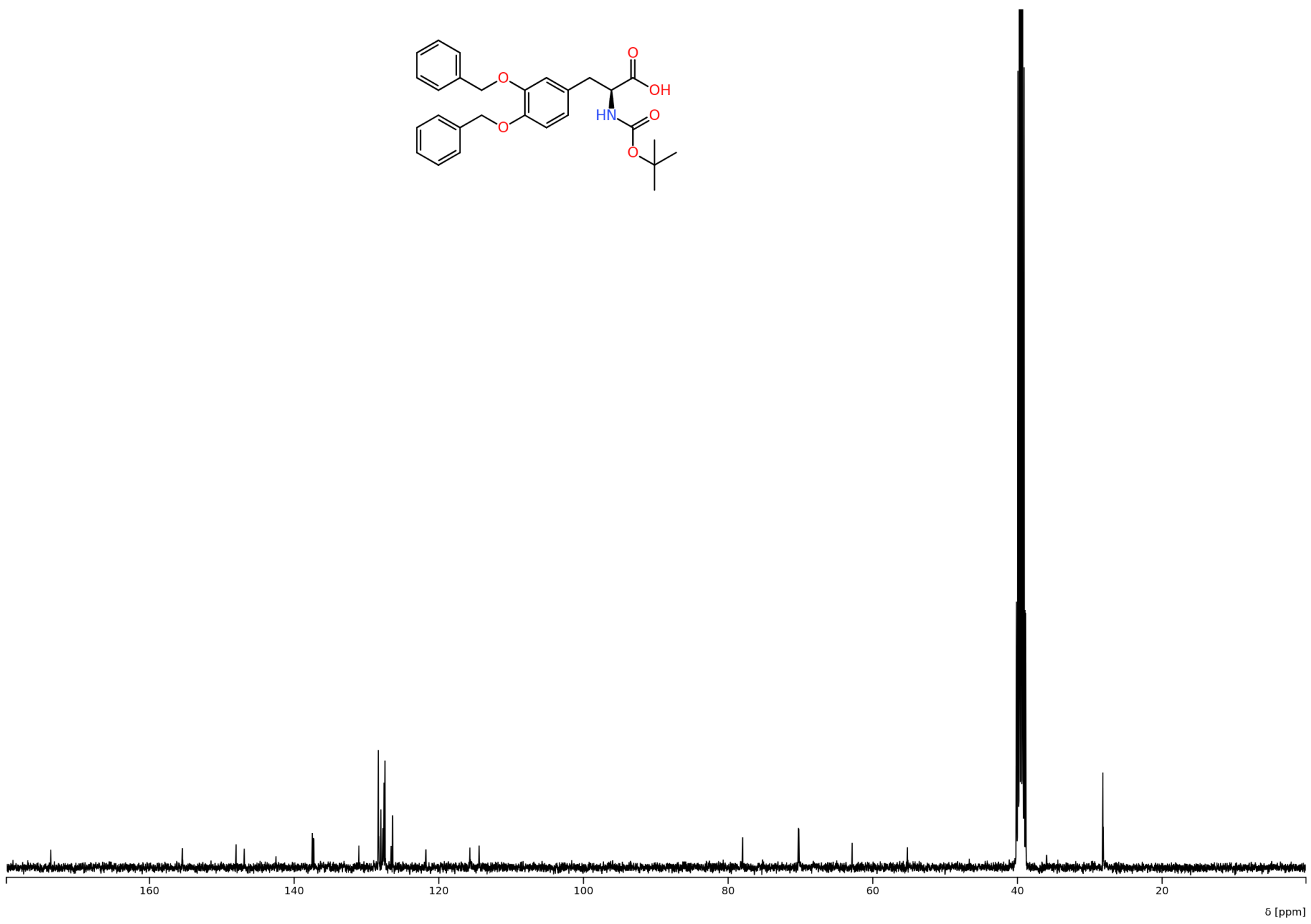
O
OH
HN
O
O
O
O
160
140
120
100
80
60
40
20
δ [ppm]

Compound 4

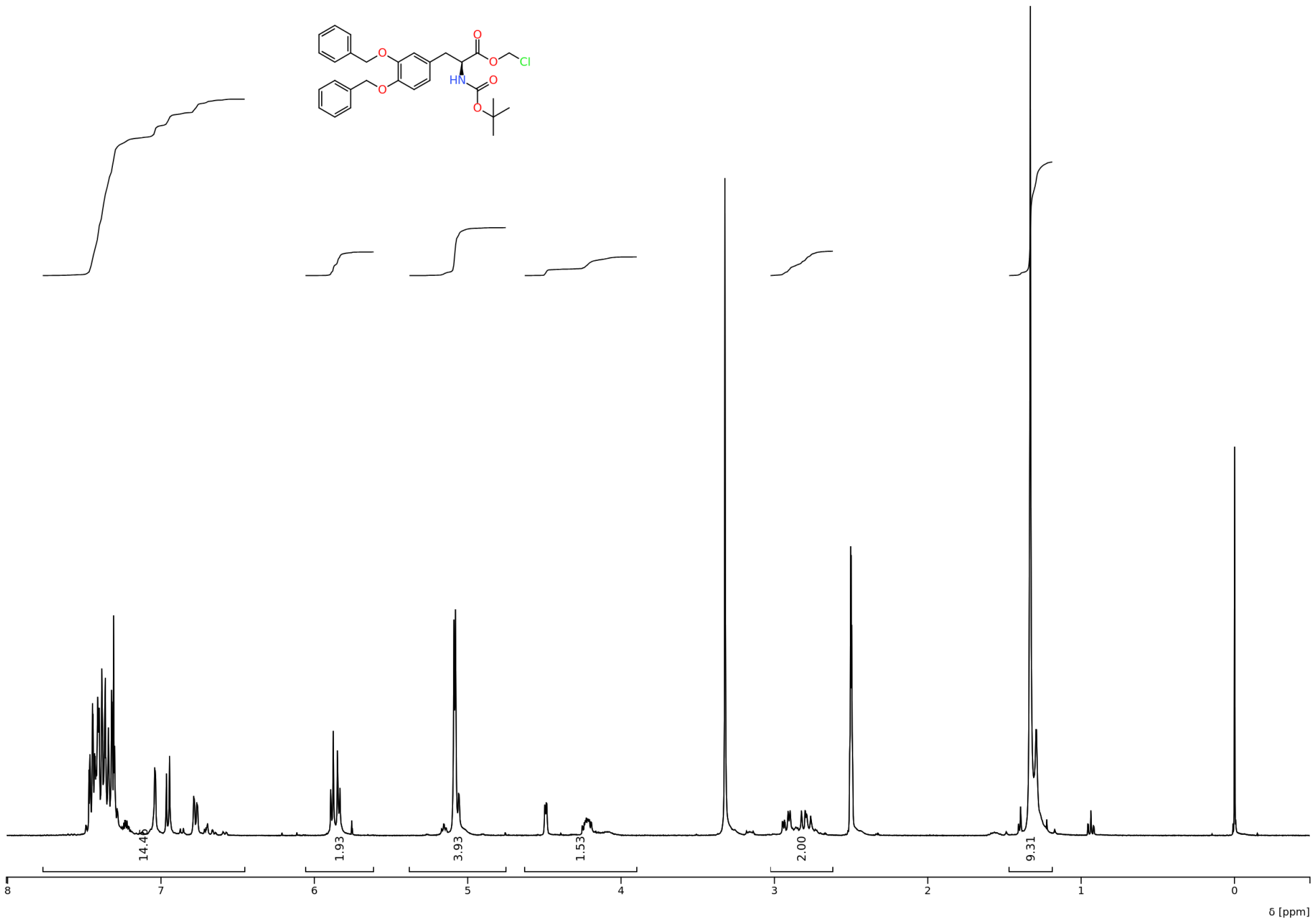

O
O
O
O
HN
O
O
Cl
14.46
1.93
3.93
1.53
2.00
9.31
8
7
6
5
4
3
2
1
0
δ [ppm]

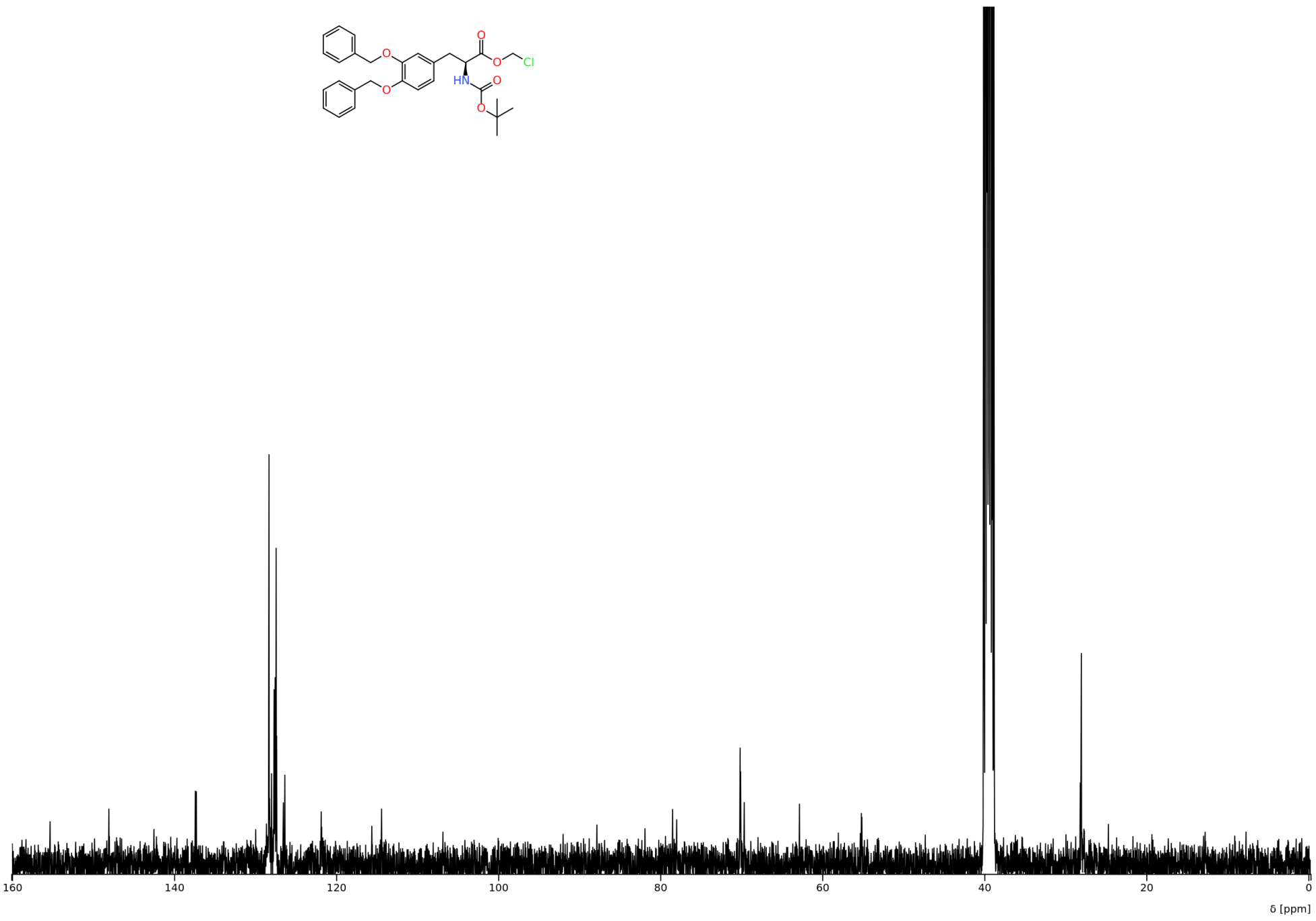

O
O
O
O
HN
O
O
Cl
160
140
120
100
80
60
40
20
0
δ [ppm]

Compound 5b

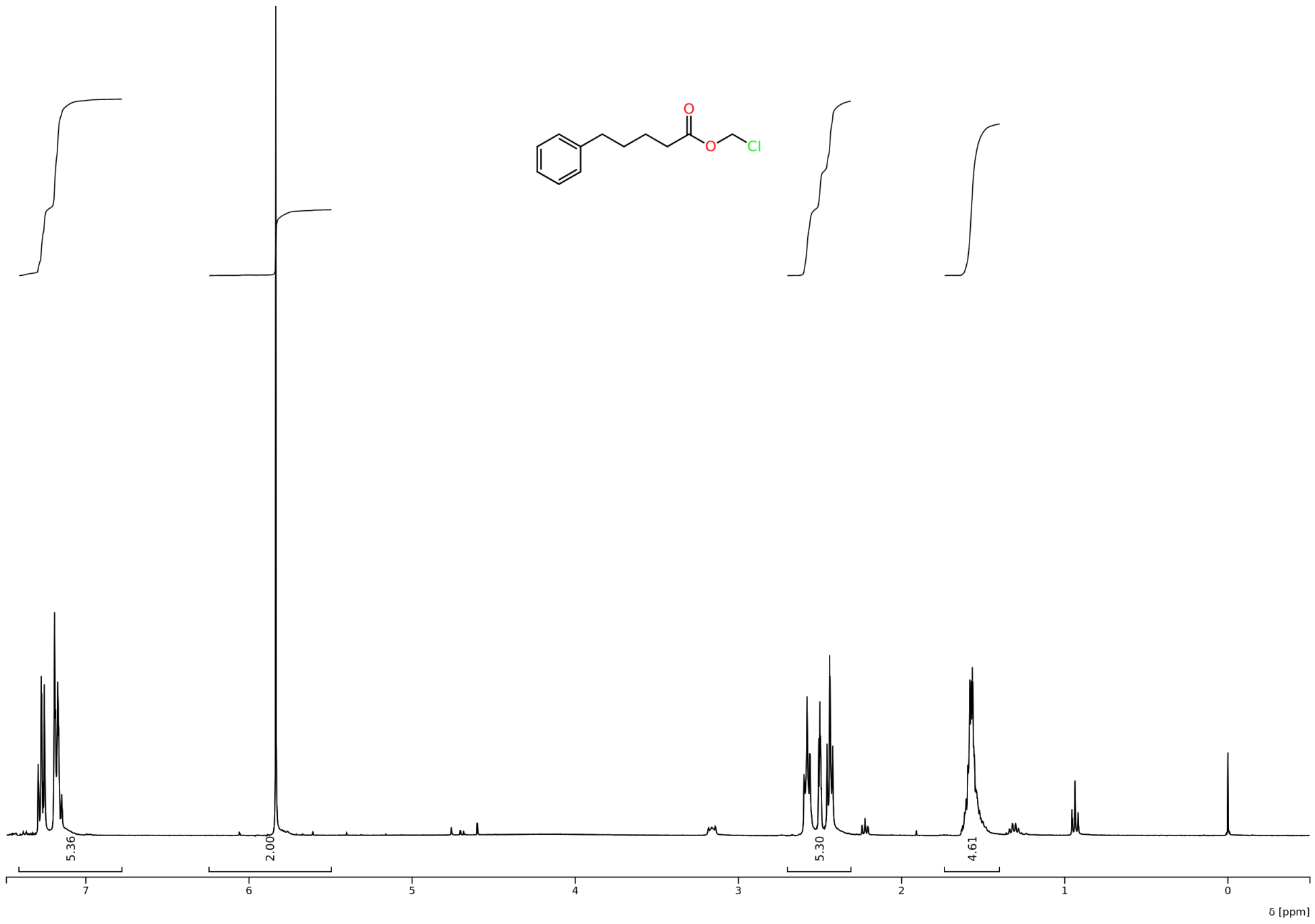
O
O
Cl
5.36
2.00
5.30
4.61
7
6
5
4
3
2
1
0
δ [ppm]

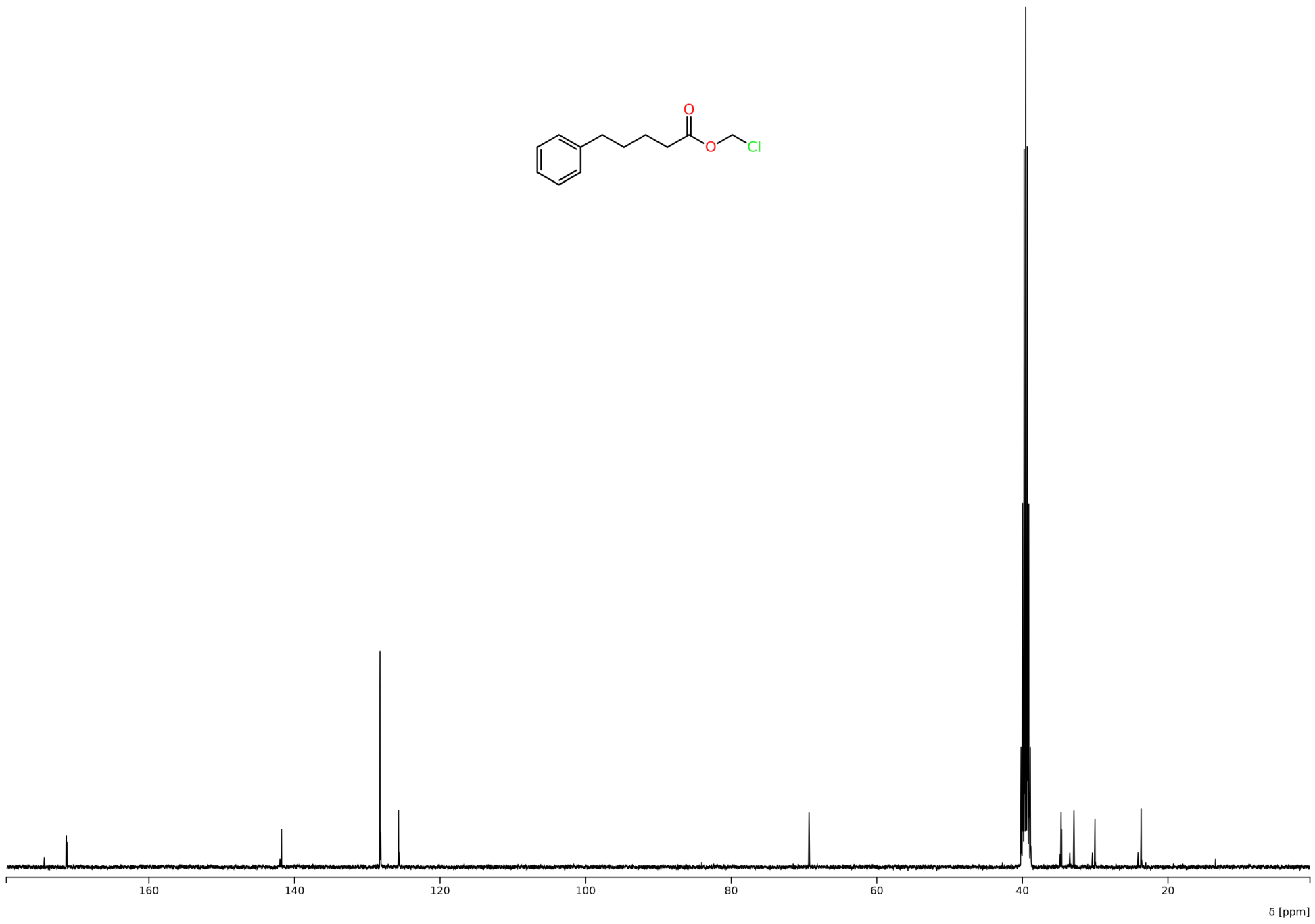
O
O
Cl
160
140
120
100
80
60
40
20
δ [ppm]

Compound 5e

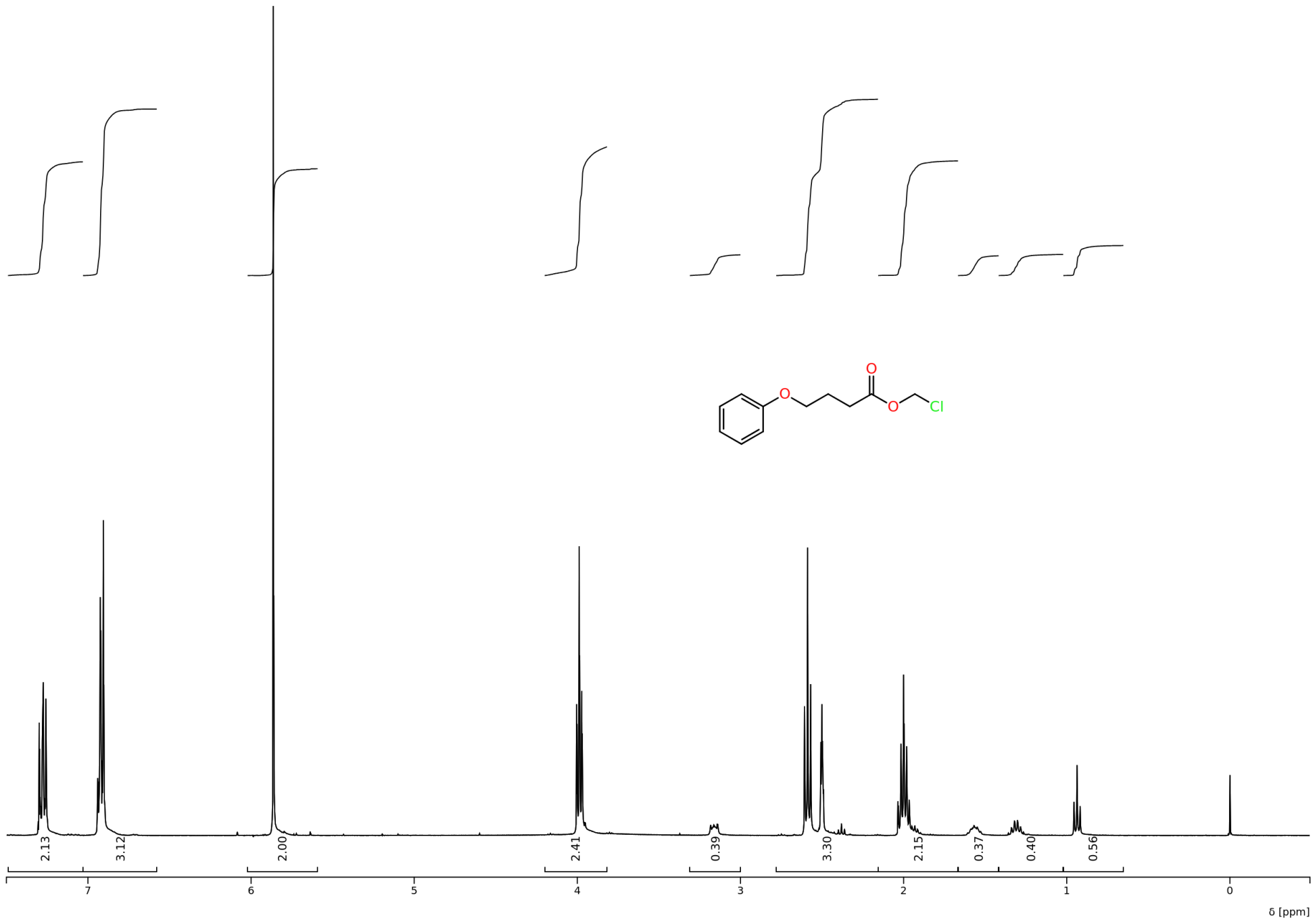

O
O
O
Cl
2.13
3.12
2.00
2.41
0.39
3.30
2.15
0.37
0.40
0.56
7
6
5
4
3
2
1
0
δ [ppm]

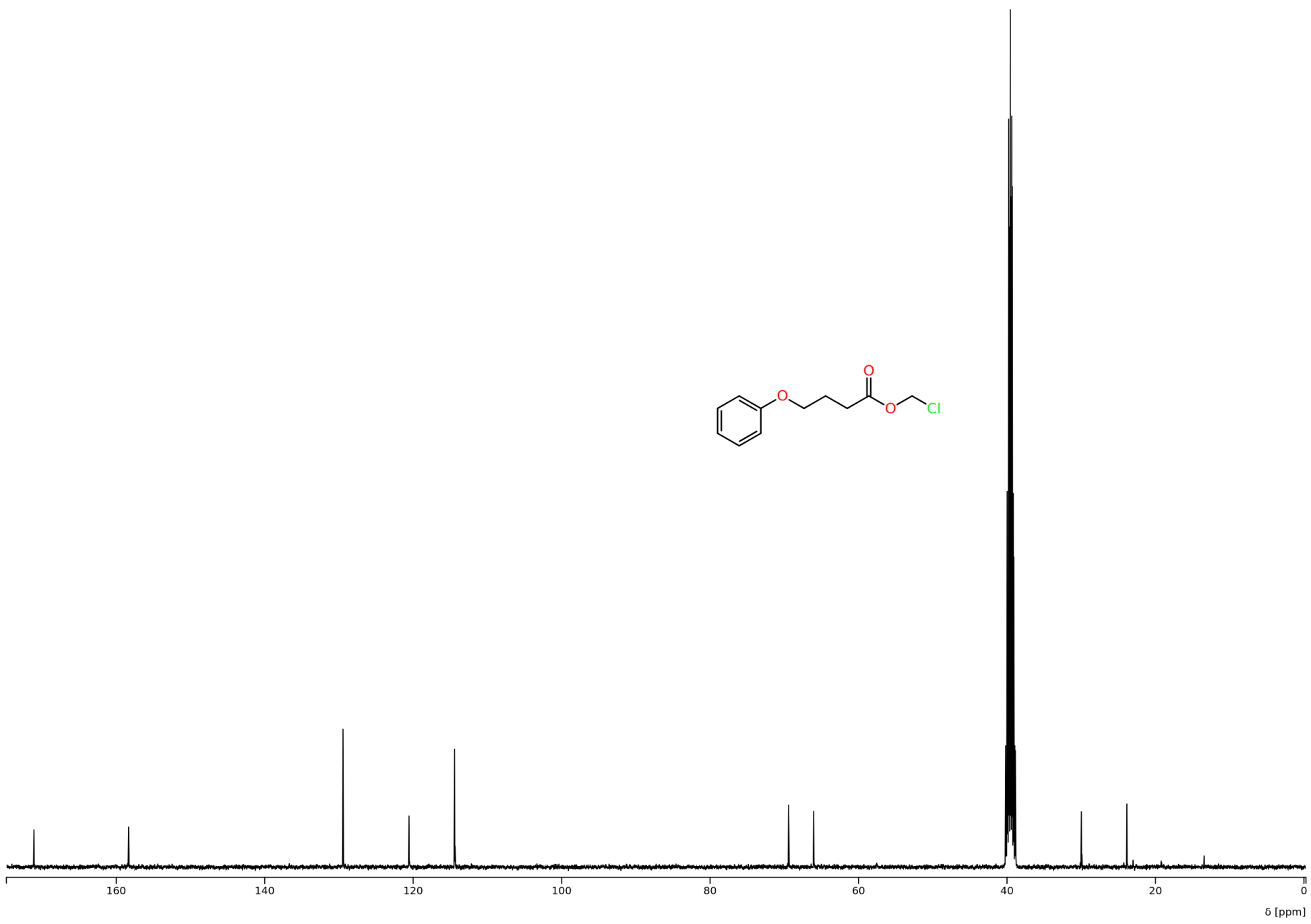

O
O
O
Cl
160
140
120
100
80
60
40
20
0
δ [ppm]

Compound 5f

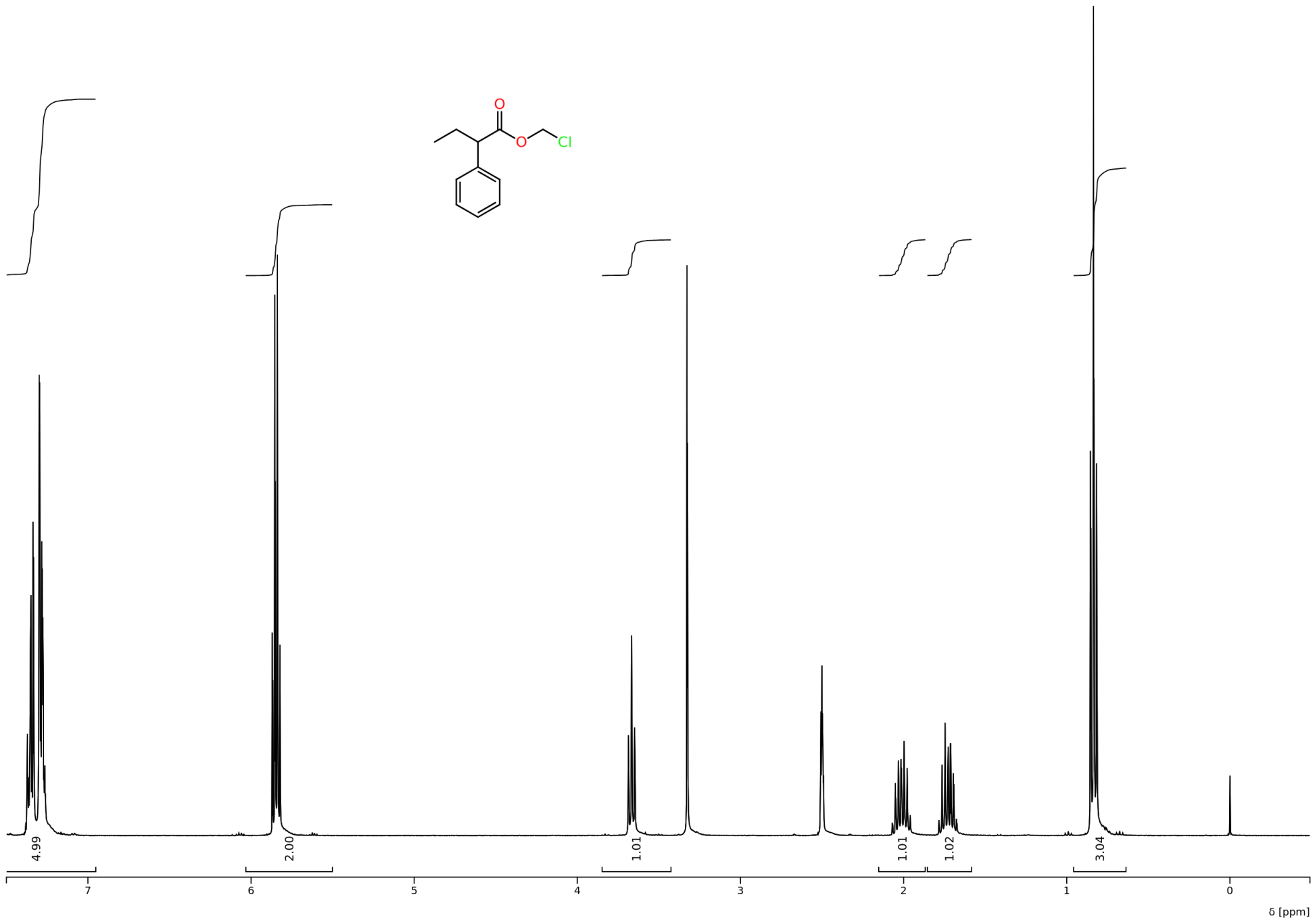

O
O
Cl
4.99
2.00
1.01
1.01
1.02
3.04
7
6
5
4
3
2
1
0
δ [ppm]

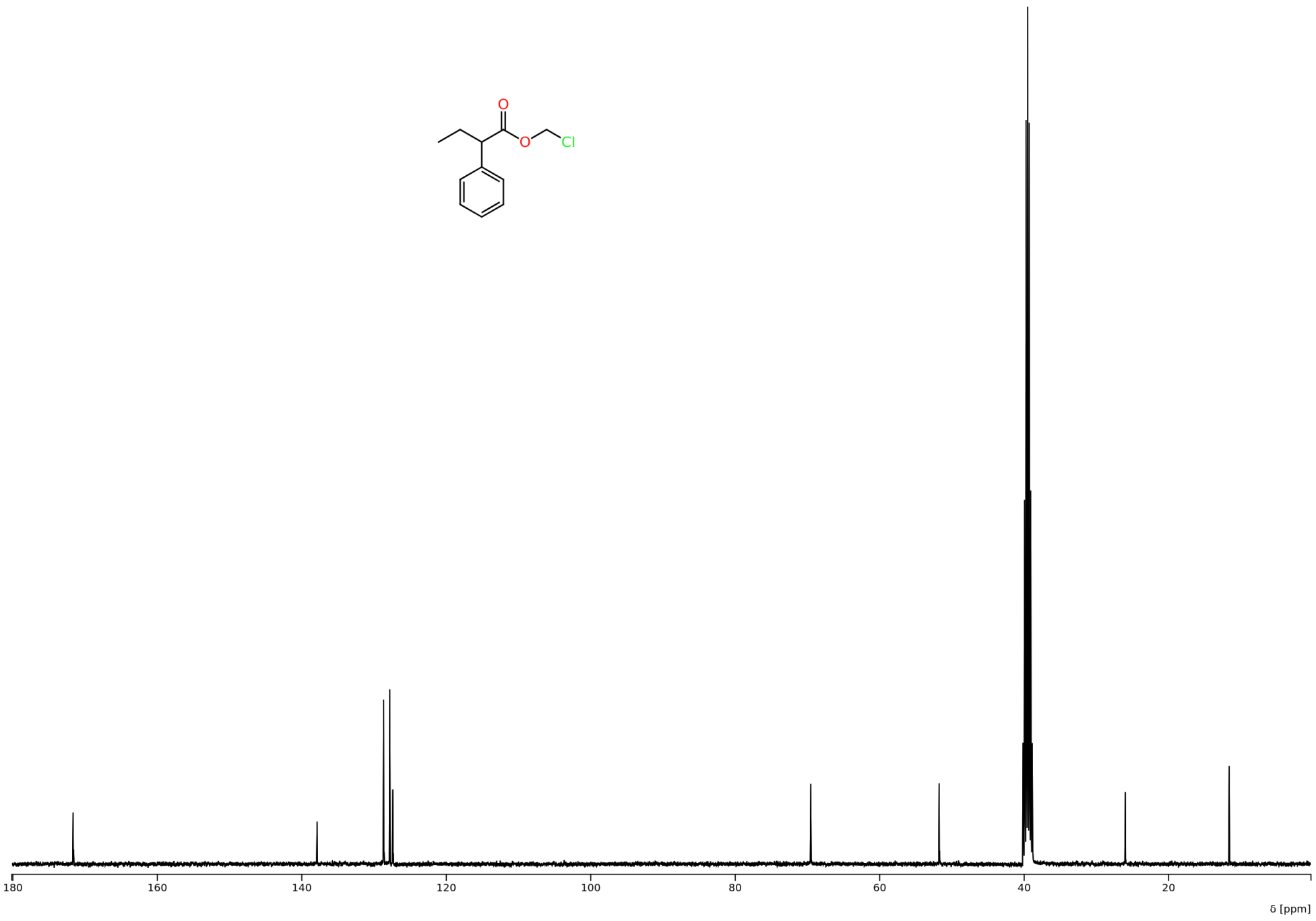

O
O
Cl
180
160
140
120
100
80
60
40
20
δ [ppm]

Compound 5g

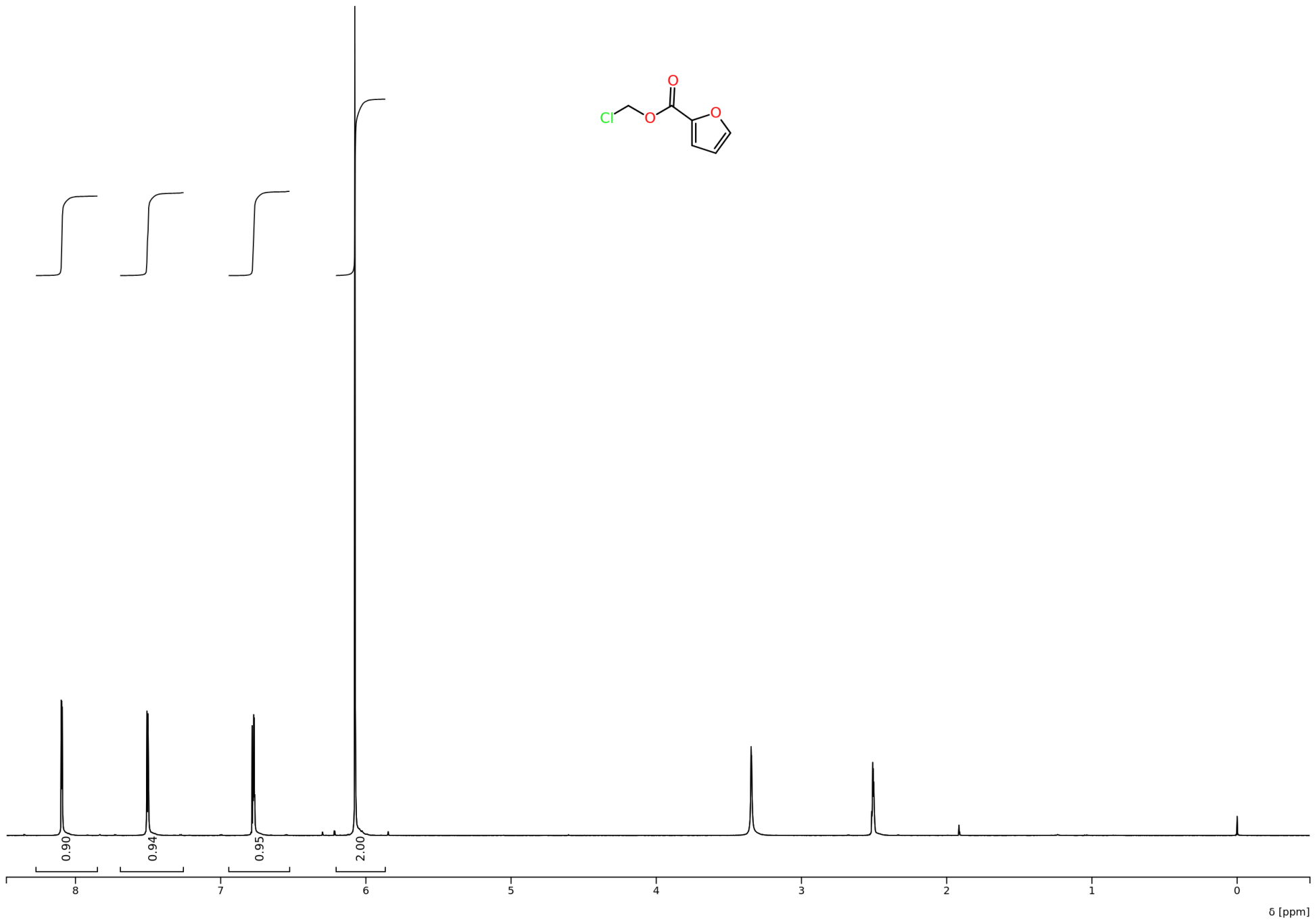

Cl
O
O
O
0.90
0.94
0.95
2.00
8
7
6
5
4
3
2
1
0
δ [ppm]

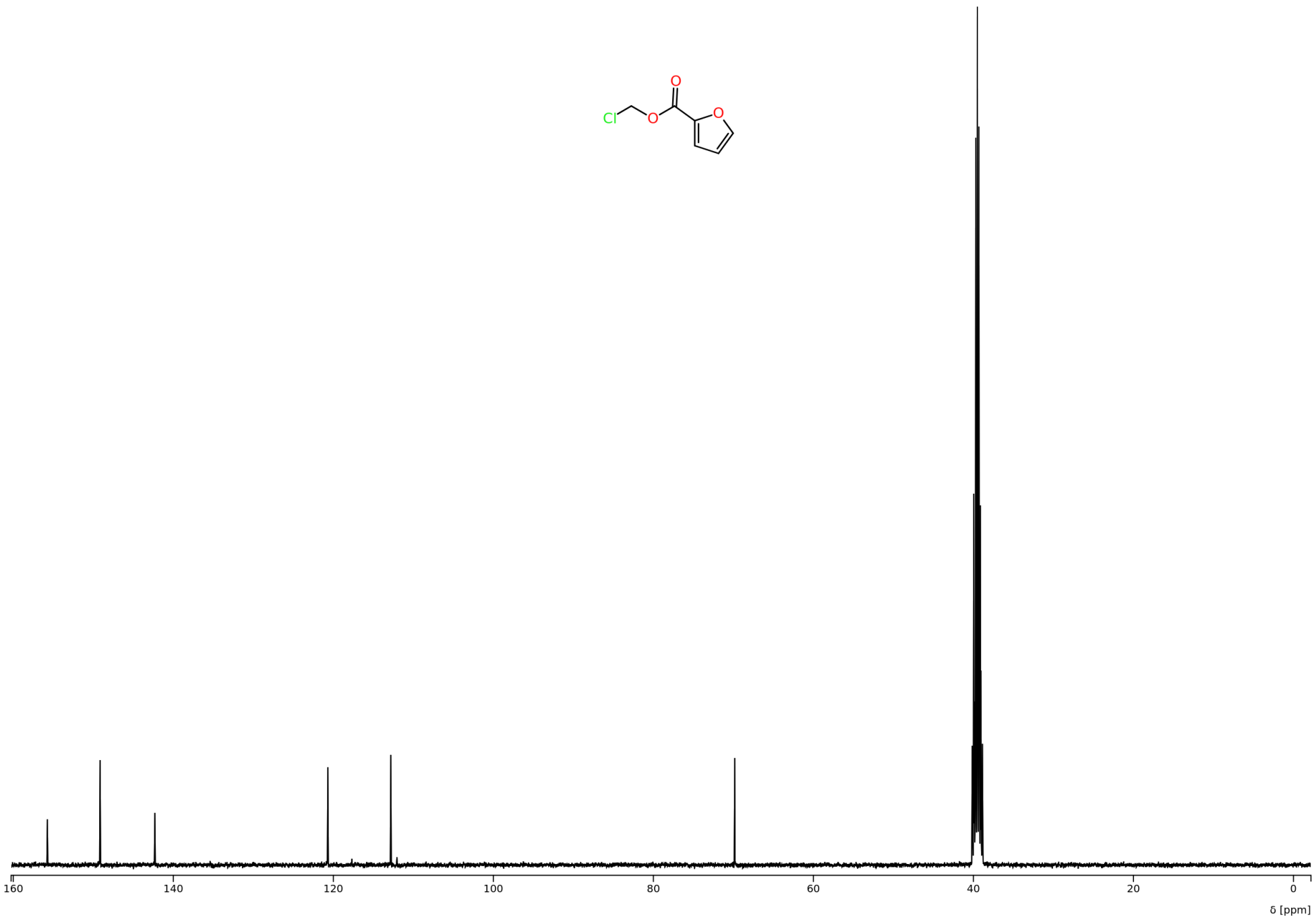

Cl
O
O
O
160
140
120
100
80
60
40
20
0
δ [ppm]

Compound 5h

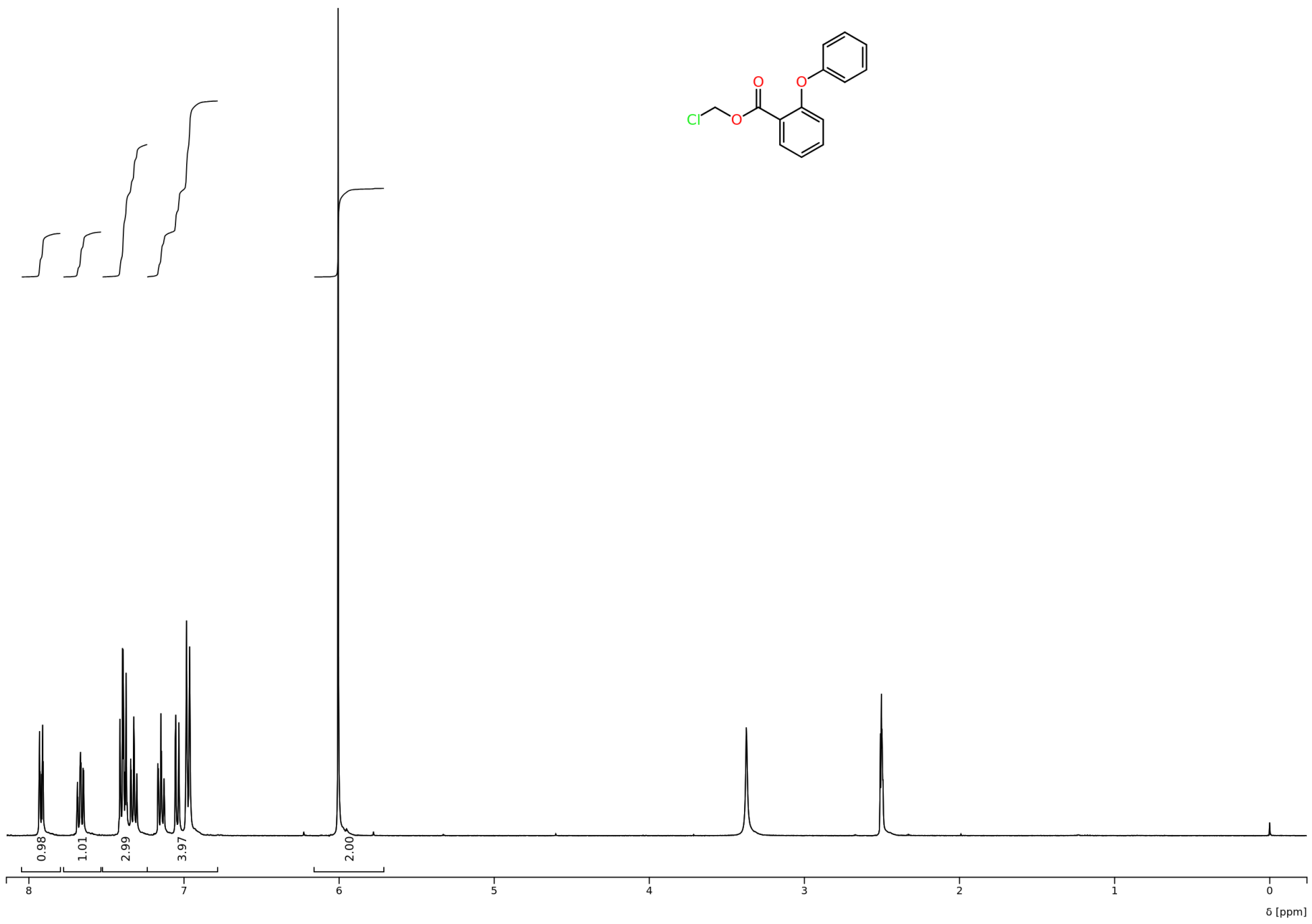

Cl
O
O
O
0.98
1.01
2.99
3.97
2.00
8
7
6
5
4
3
2
1
0
δ [ppm]

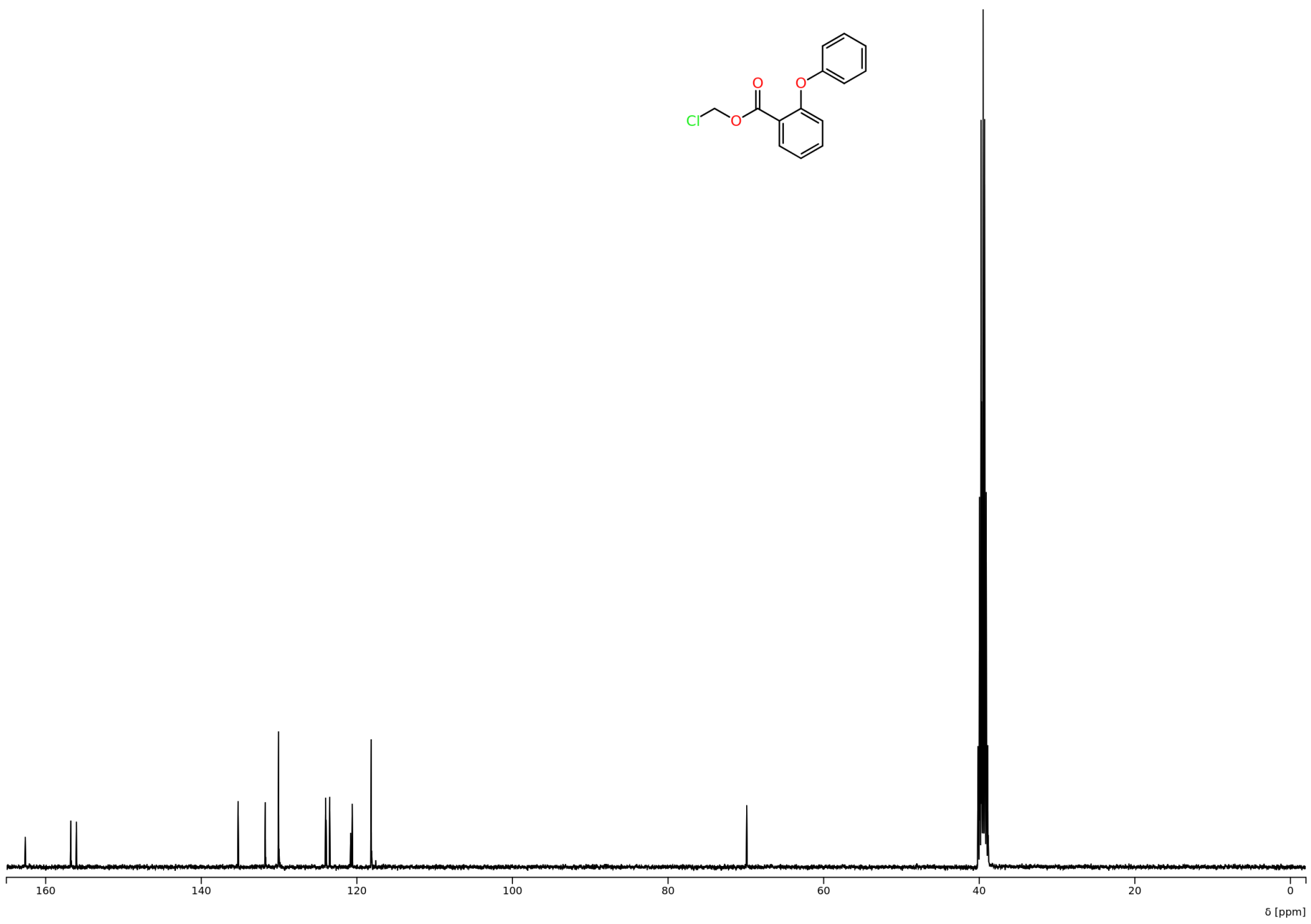

Cl
O
O
O
160
140
120
100
80
60
40
20
0
δ [ppm]

Compound 5i

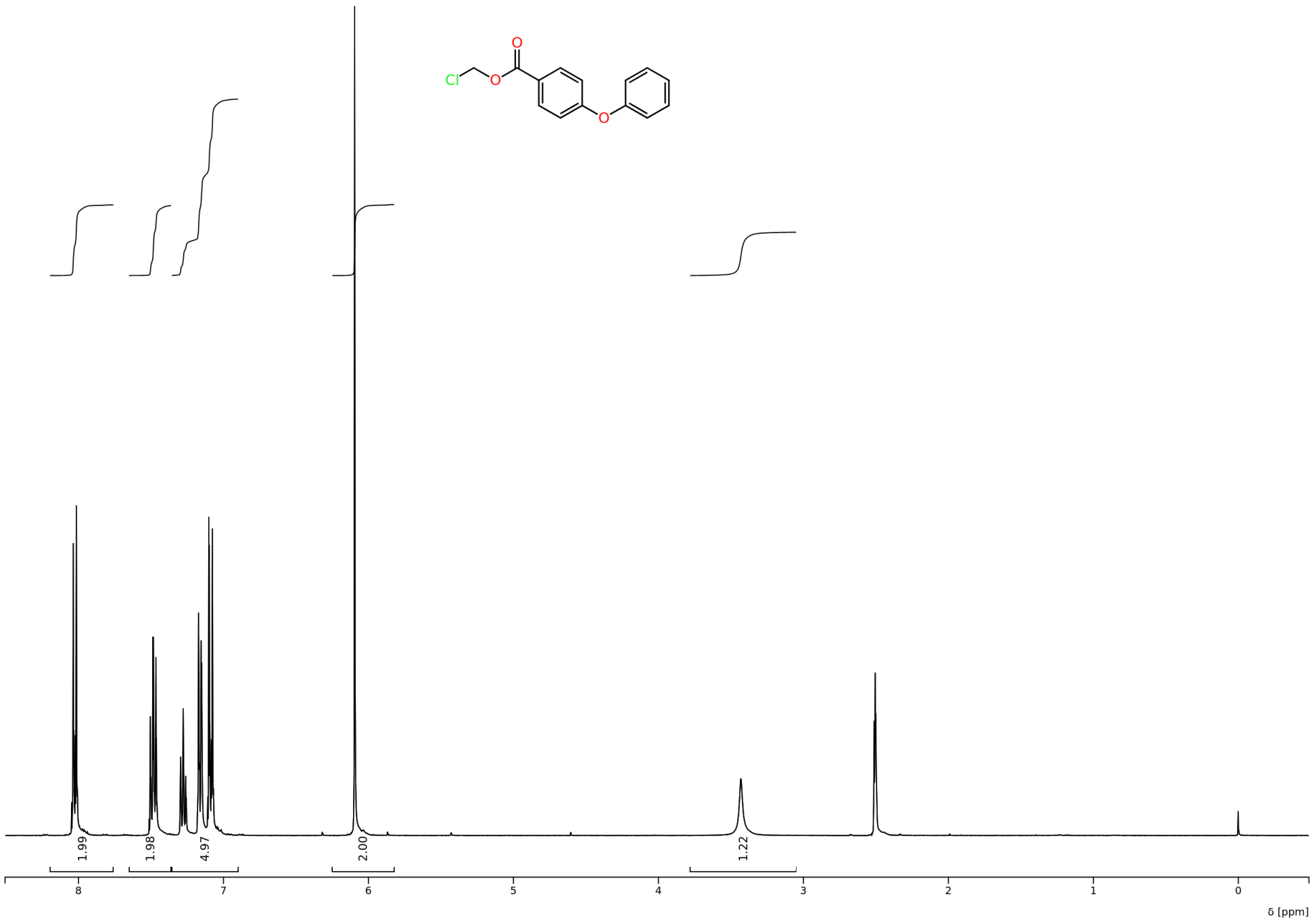
Cl
O
O
O
1.99
1.98
4.97
2.00
1.22
8
7
6
5
4
3
2
1
0
δ [ppm]

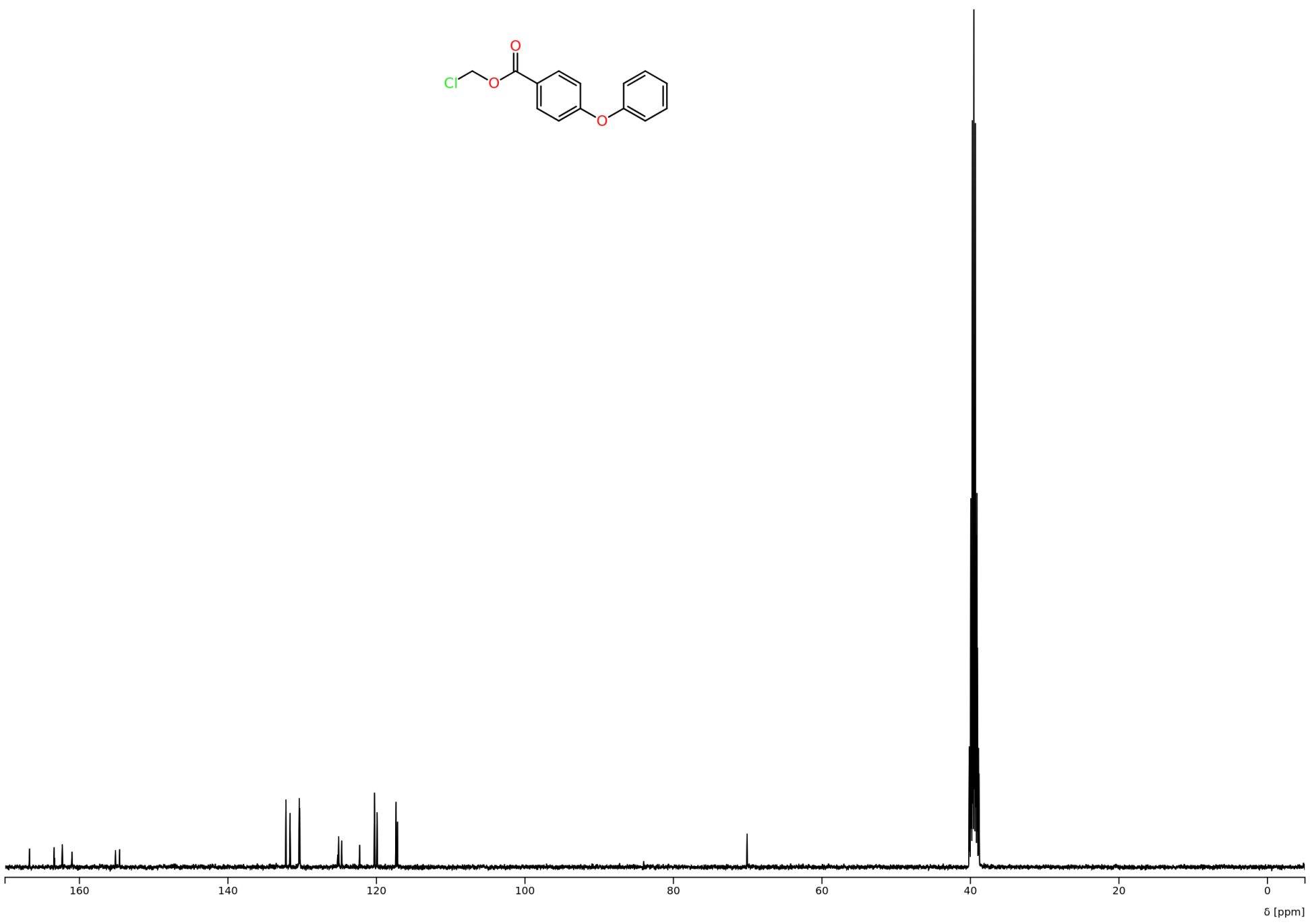
Cl
O
O
O
160
140
120
100
80
60
40
20
0
δ [ppm]

Compound 5k

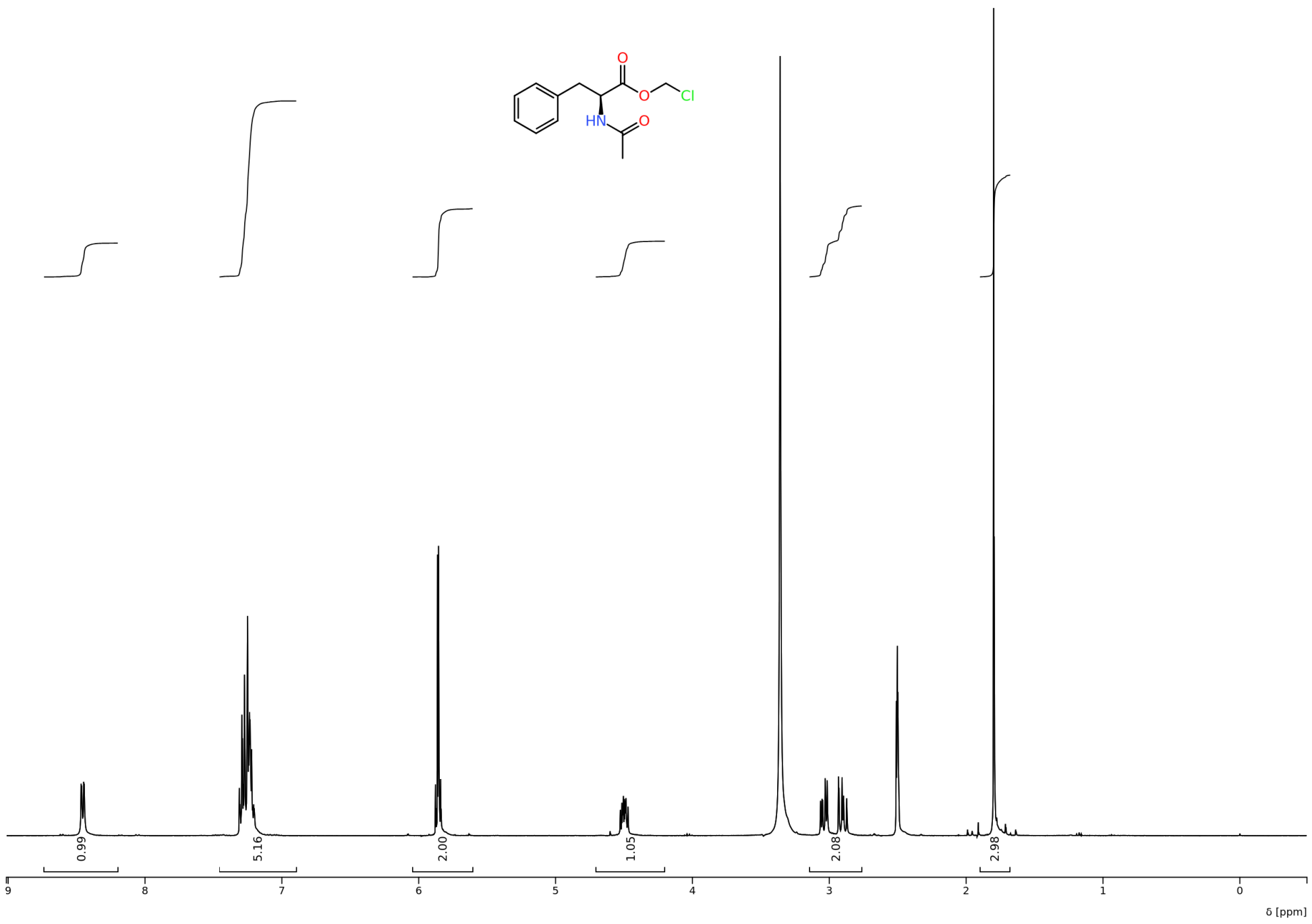

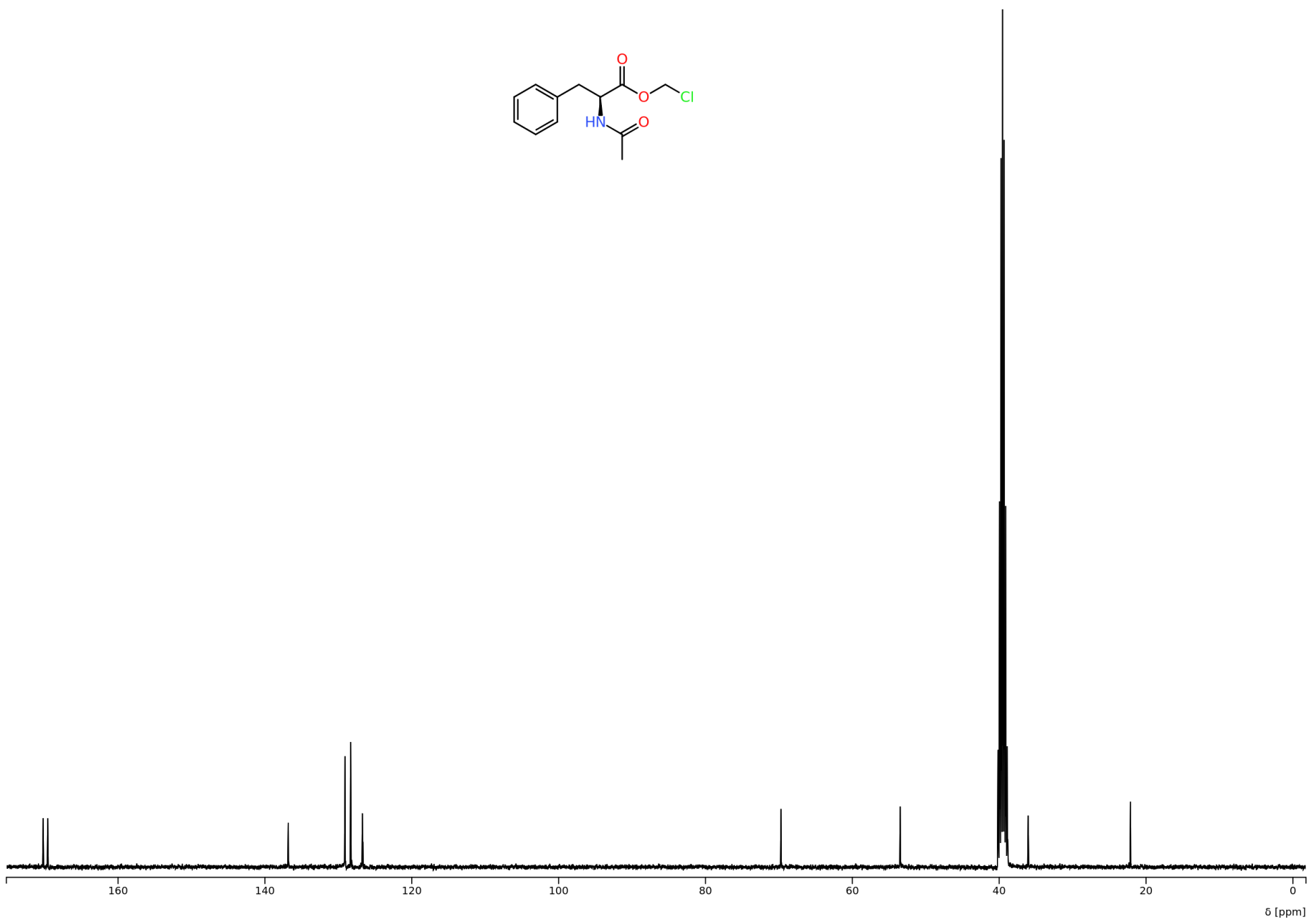

Compound 5l

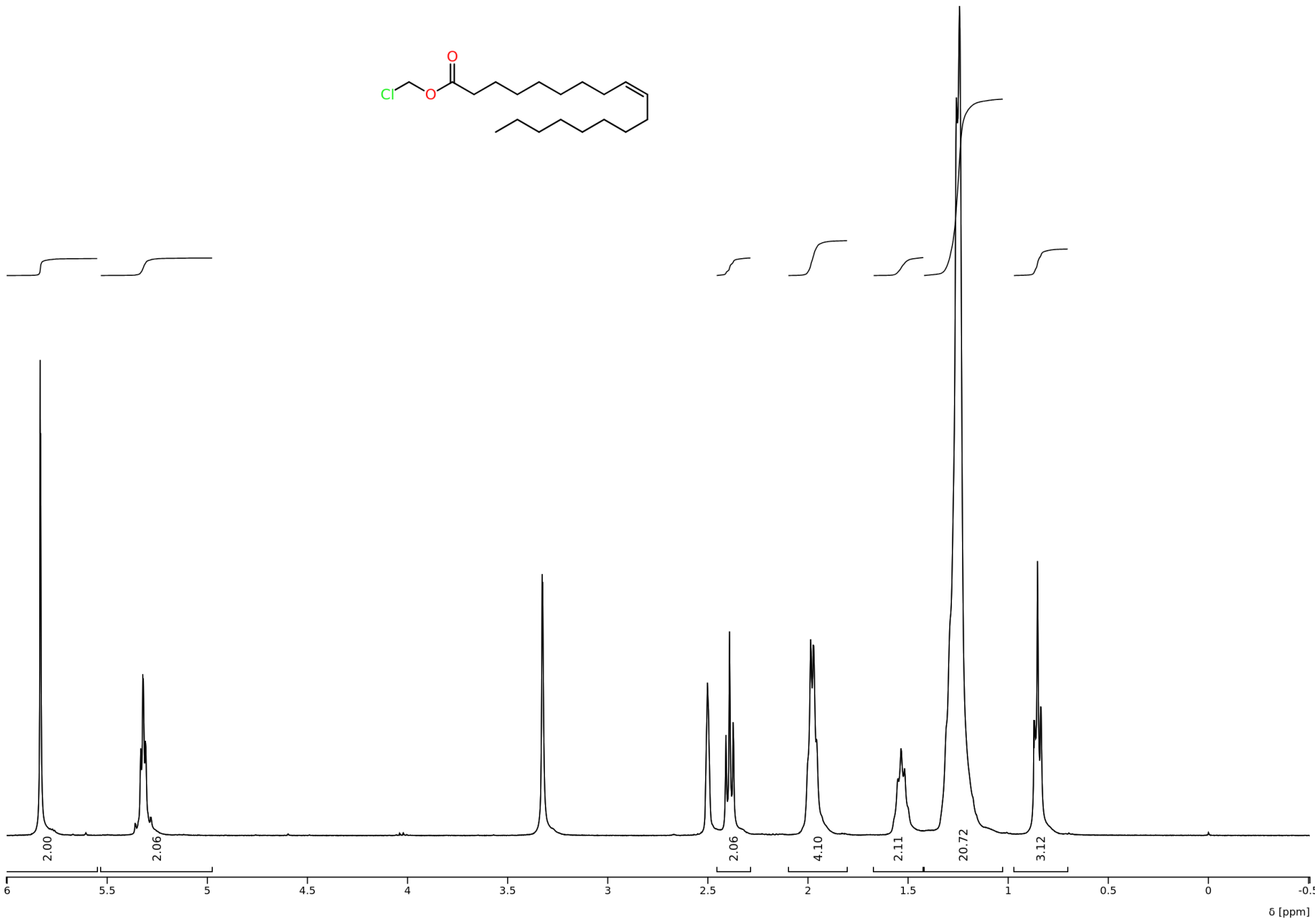
Cl
O
O
2.00
2.06
2.06
4.10
2.11
20.72
3.12
6
5.5
5
4.5
4
3.5
3
2.5
2
1.5
1
0.5
0
-0.5
δ [ppm]

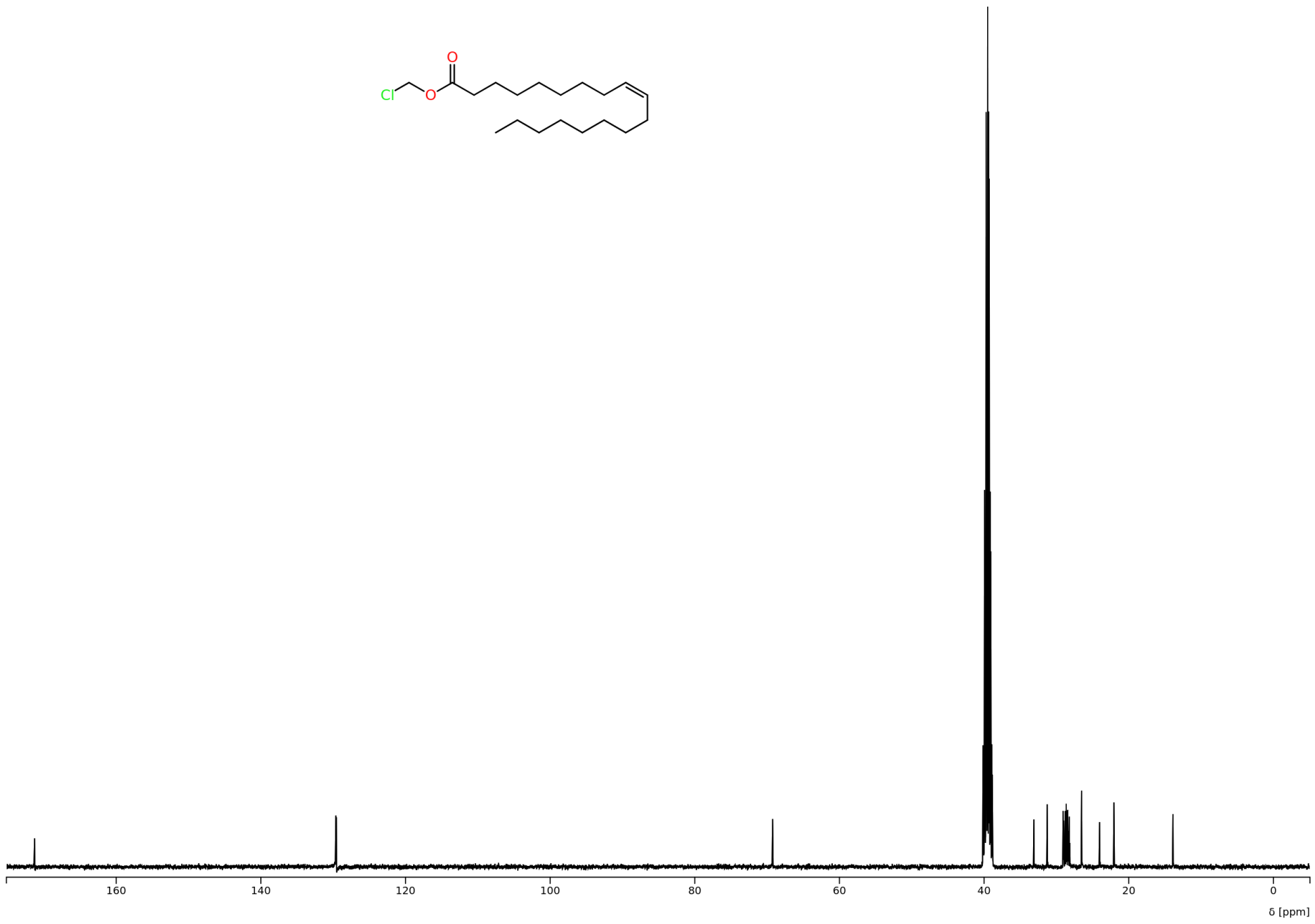
Cl
O
O
160
140
120
100
80
60
40
20
0
δ [ppm]

Compound 5m

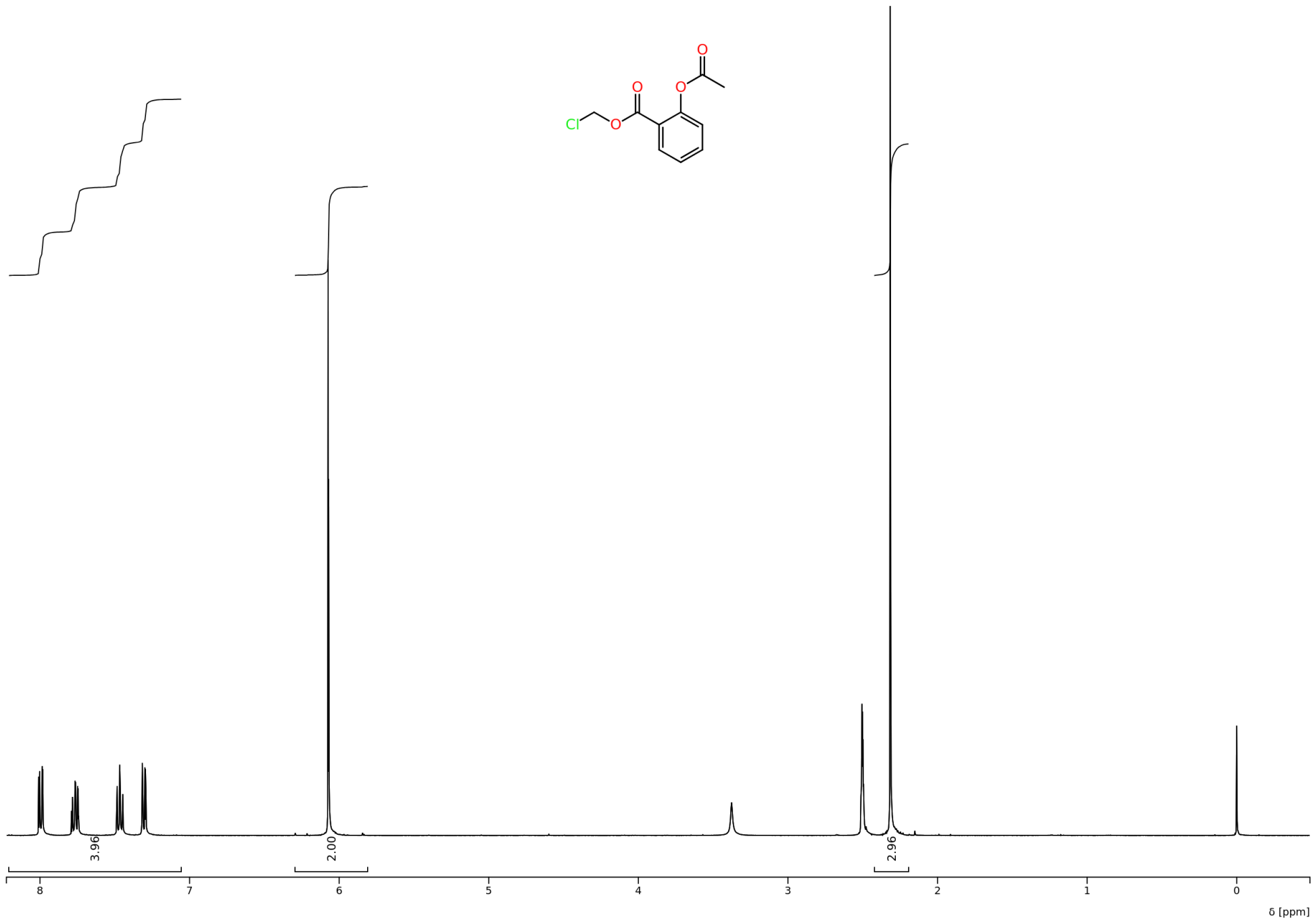
O
O
O
O
Cl
3.96
2.00
2.96
8
7
6
5
4
3
2
1
0
δ [ppm]

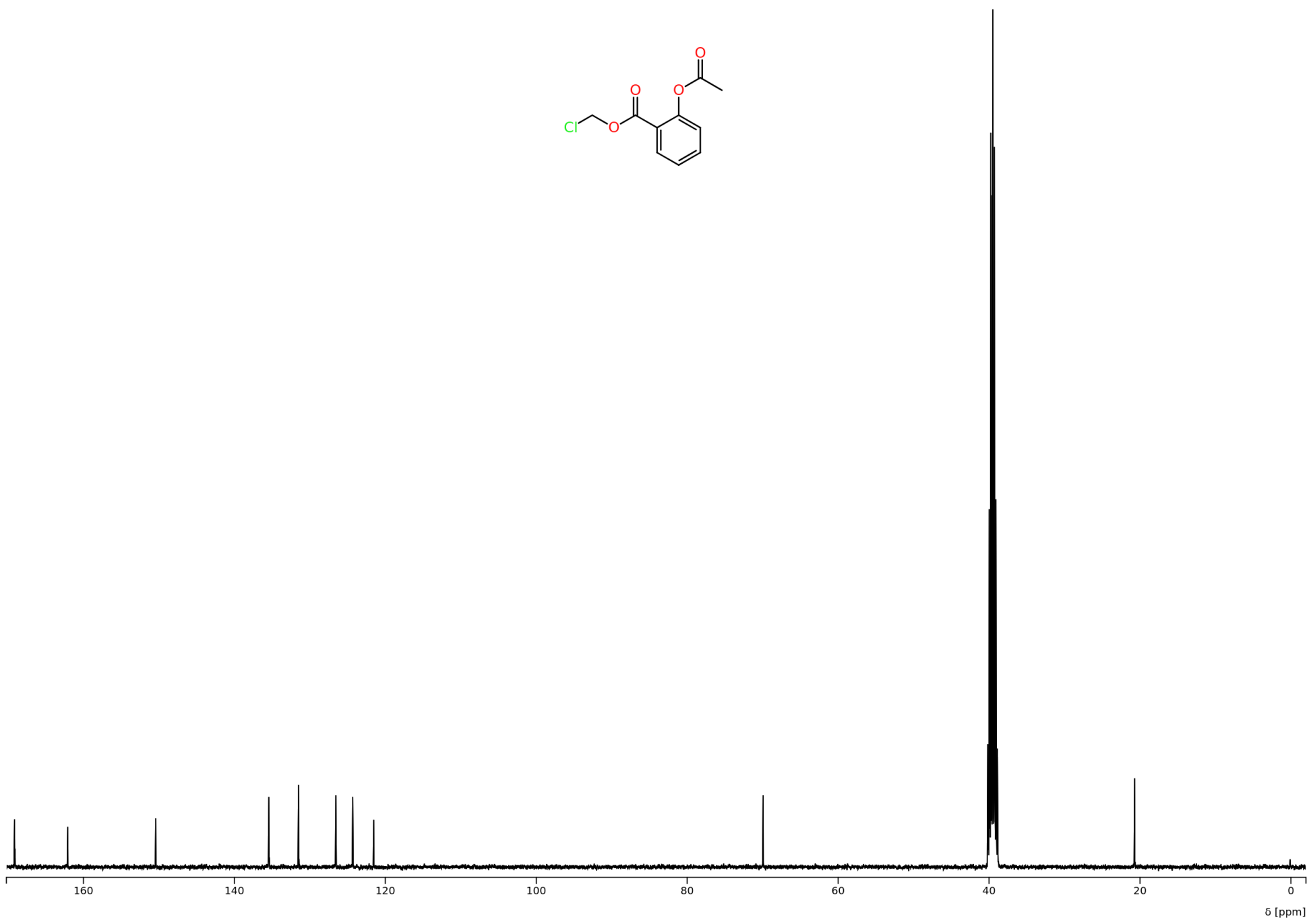
O
O
O
O
Cl
160
140
120
100
80
60
40
20
0
δ [ppm]

Compound 5n

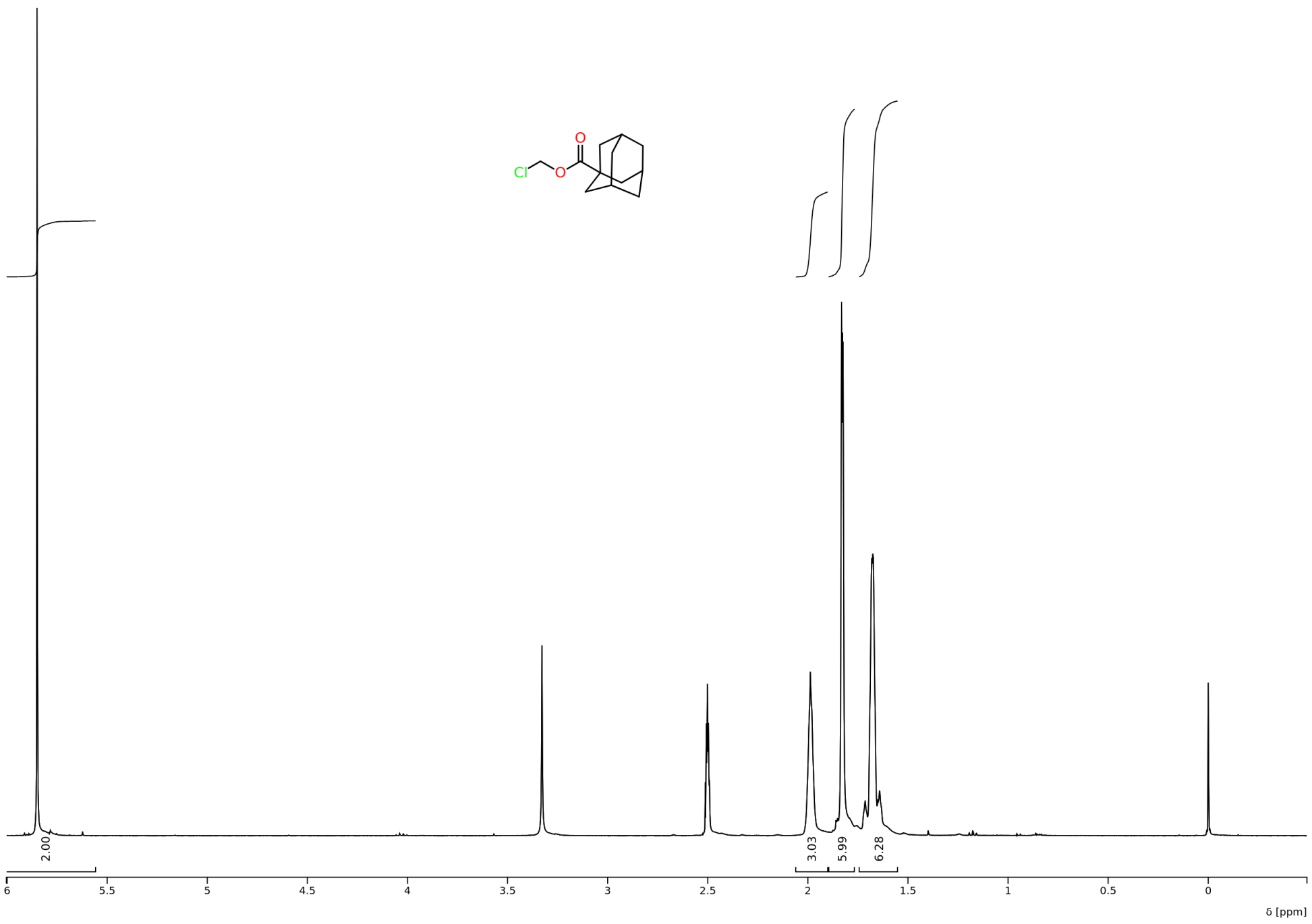

Cl
O
O
2.00
3.03
5.99
6.28
6
5.5
5
4.5
4
3.5
3
2.5
2
1.5
1
0.5
0
δ [ppm]

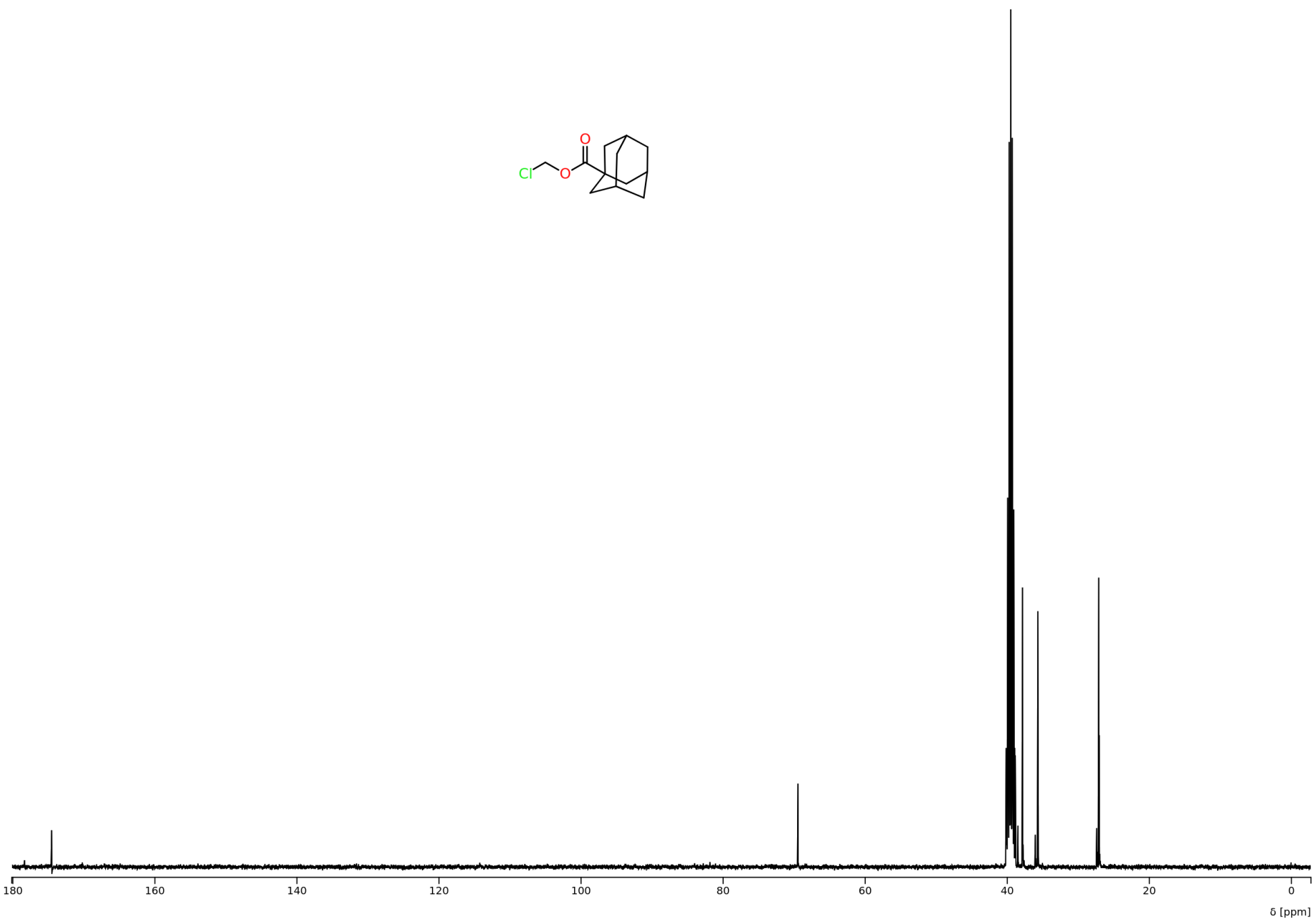

Cl
O
O
180
160
140
120
100
80
60
40
20
0
δ [ppm]

Compound 6a

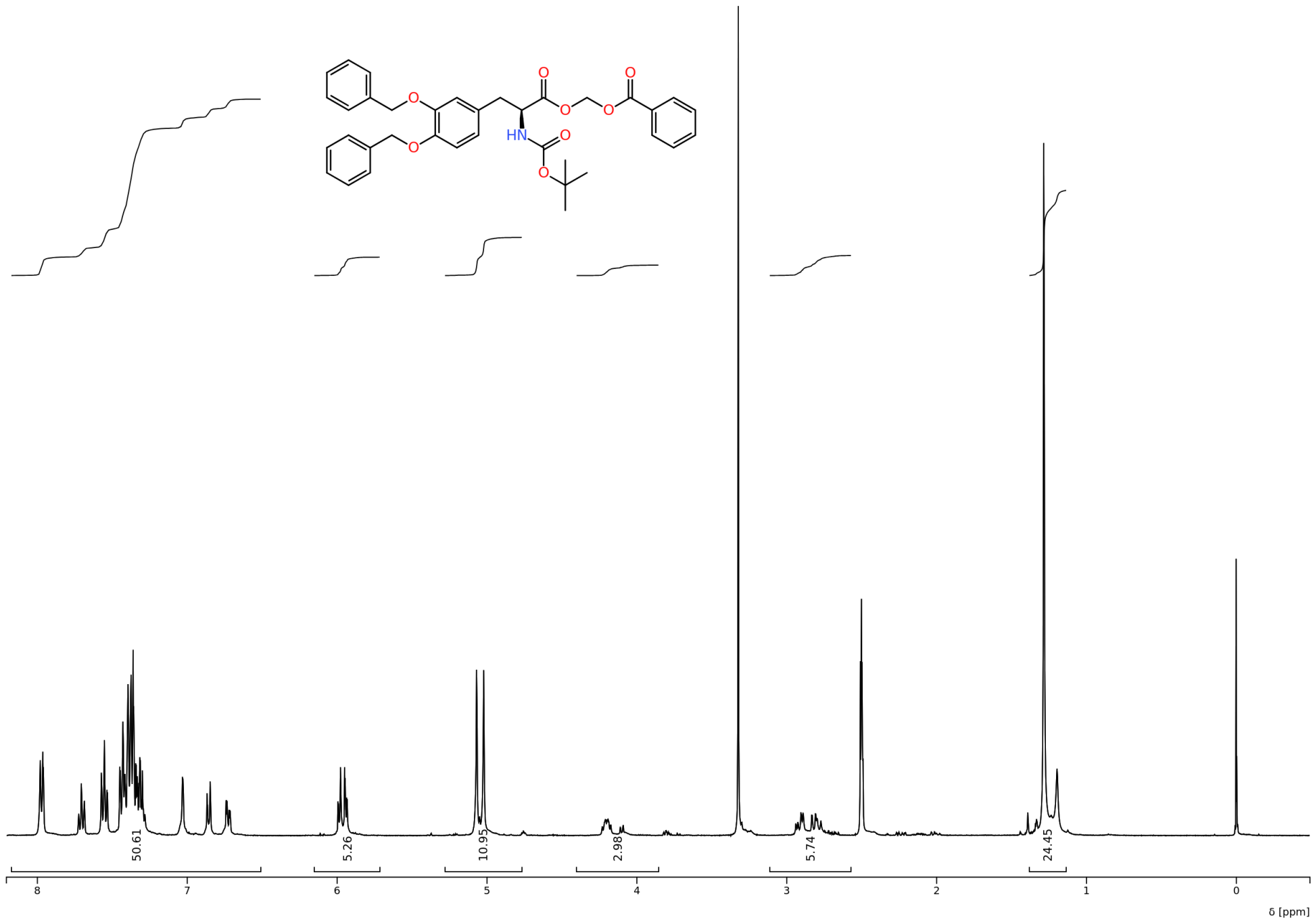

50.61
5.26
10.95
2.98
5.74
24.45
8
7
6
5
4
3
2
1
0
δ [ppm]

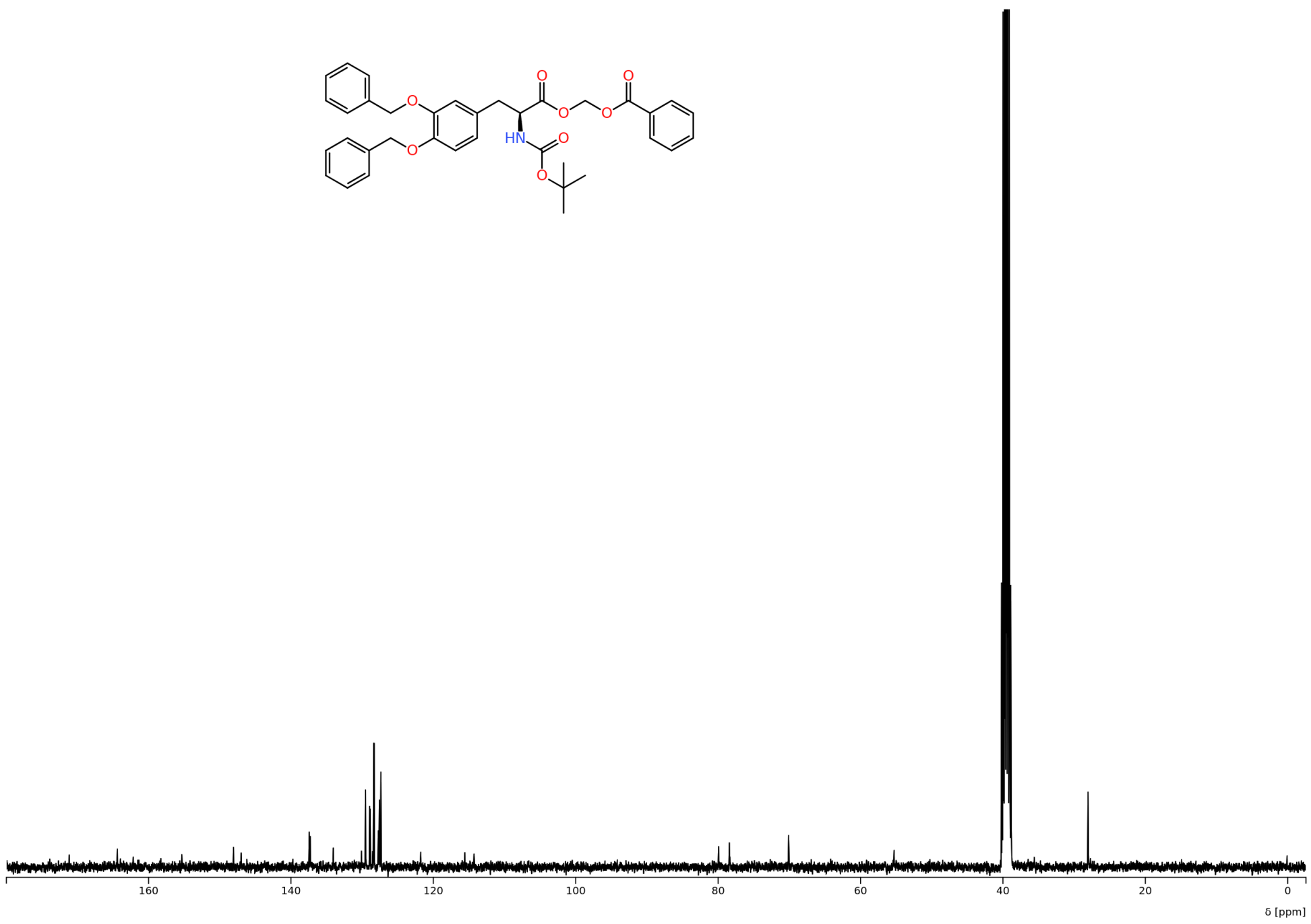

160
140
120
100
80
60
40
20
0
δ [ppm]

Compound 6b

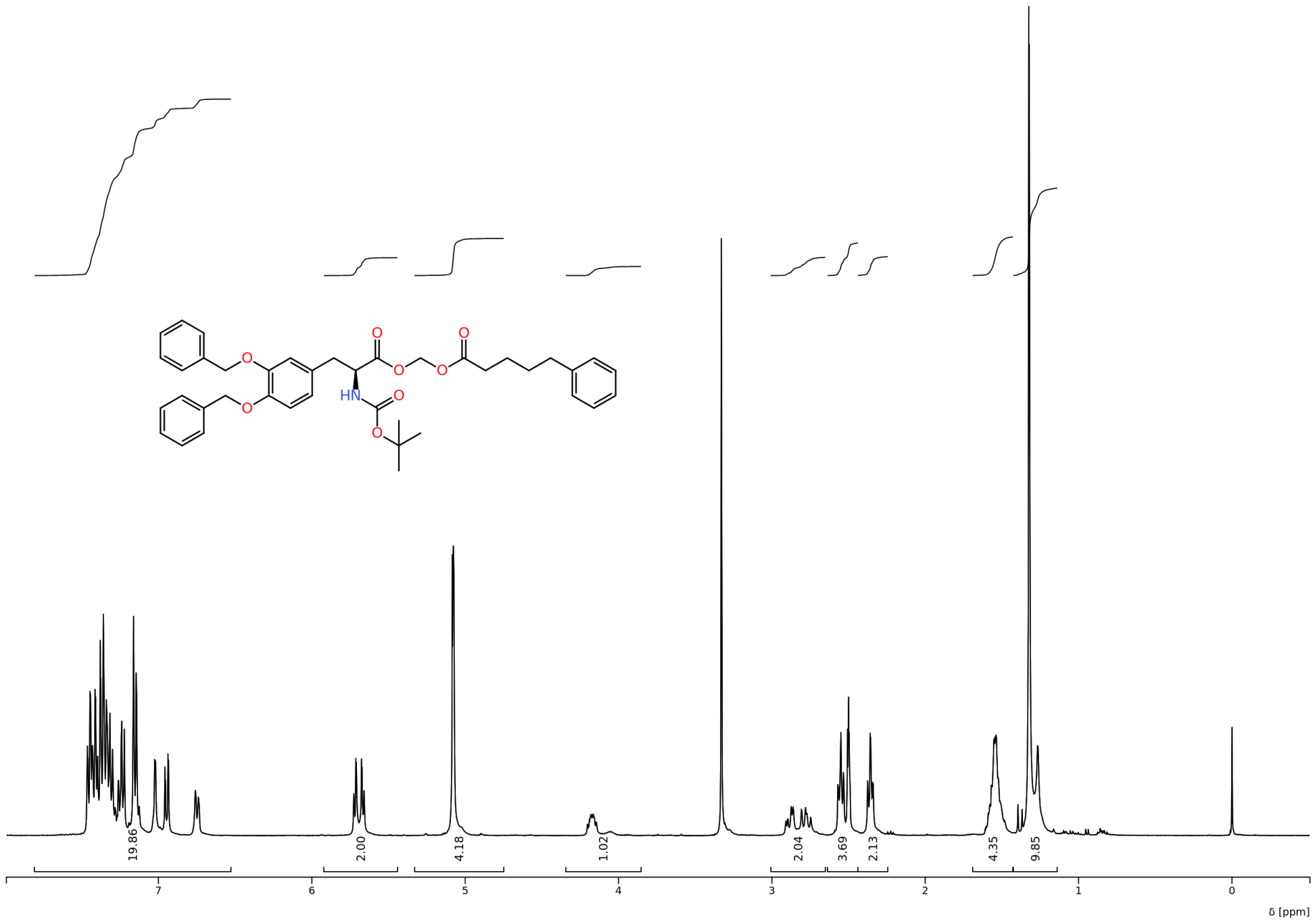

19.86
2.00
4.18
1.02
2.04
3.69
2.13
4.35
9.85
7
6
5
4
3
2
1
0
δ [ppm]

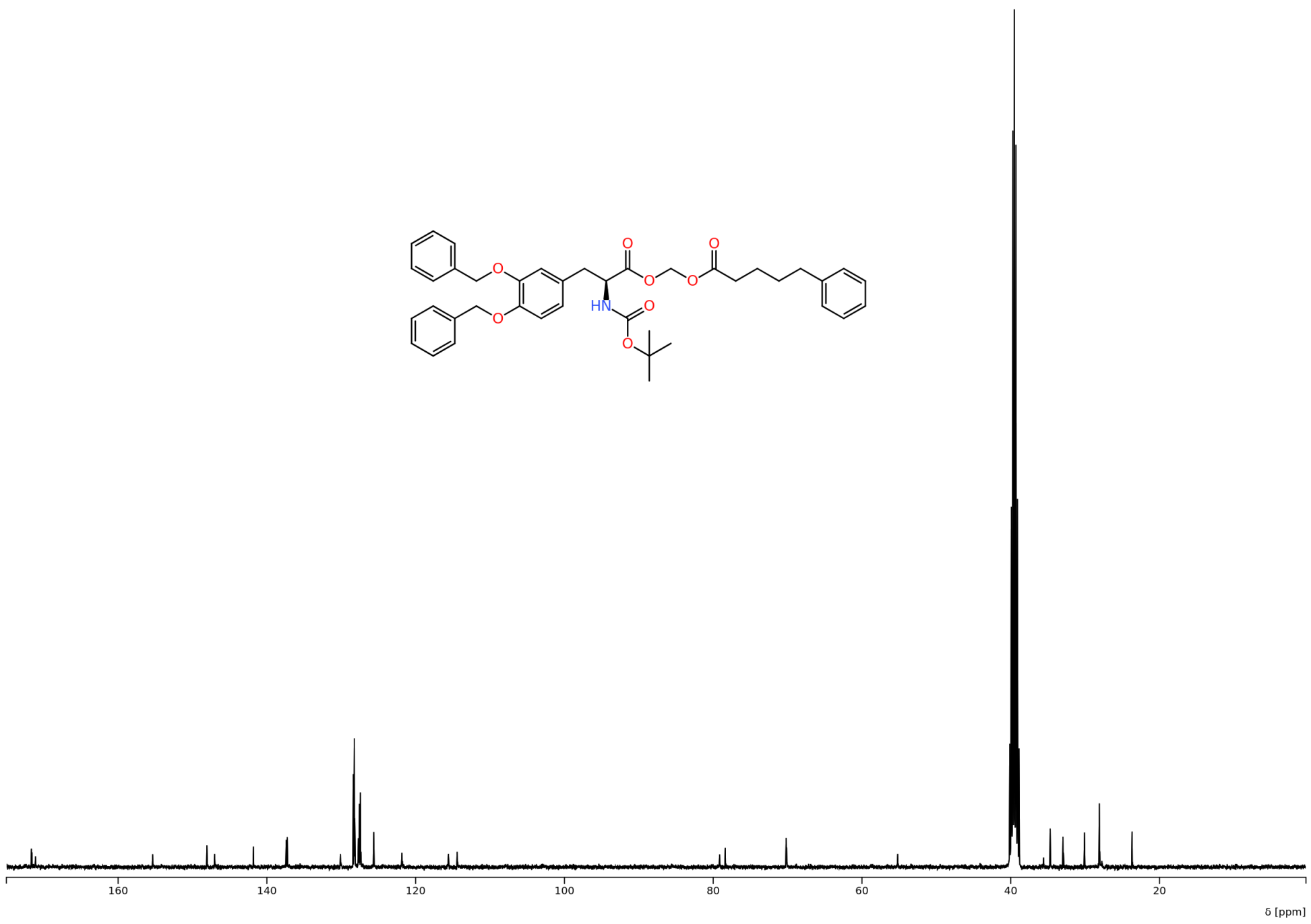

160
140
120
100
80
60
40
20
δ [ppm]

Compound 6c

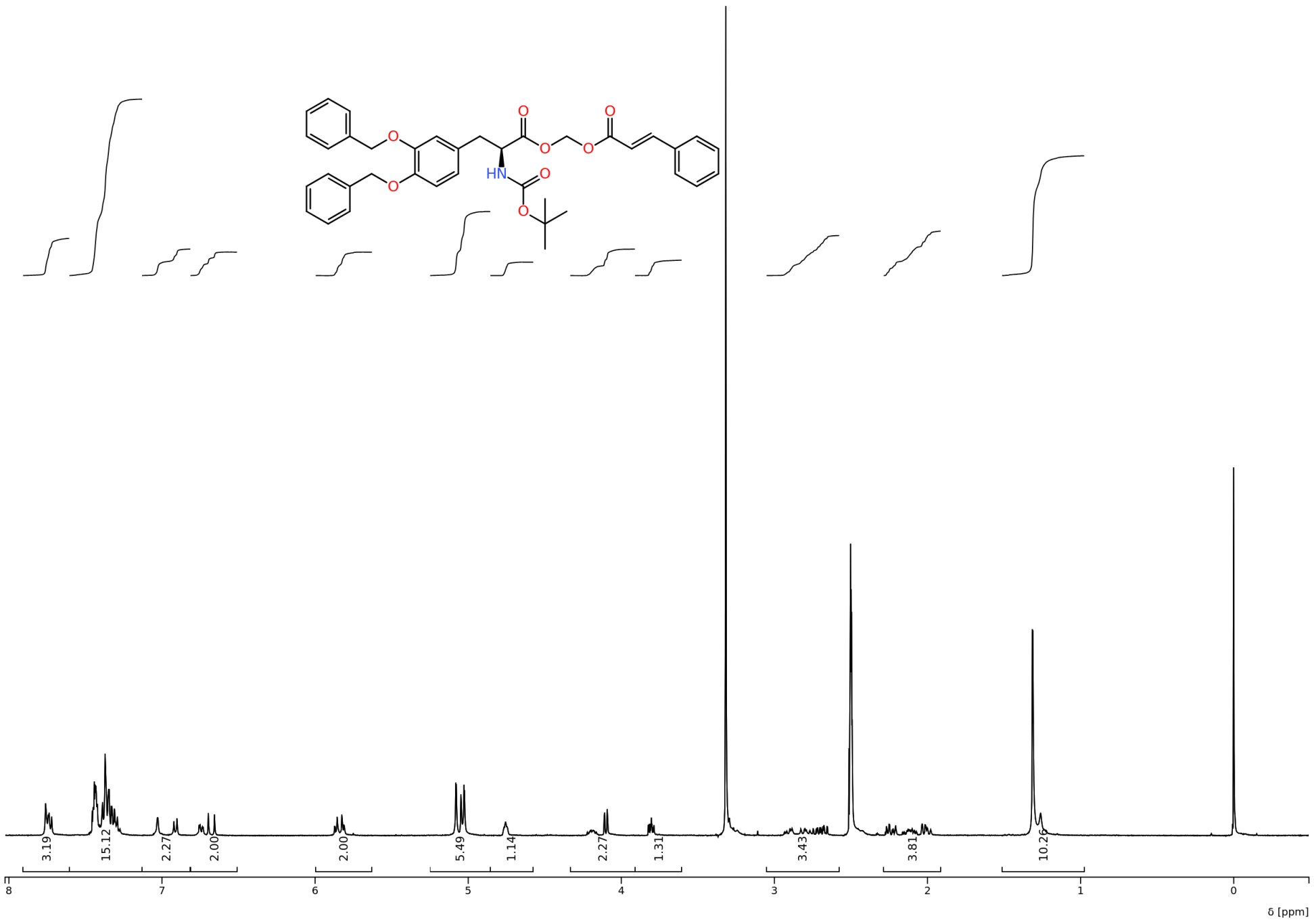

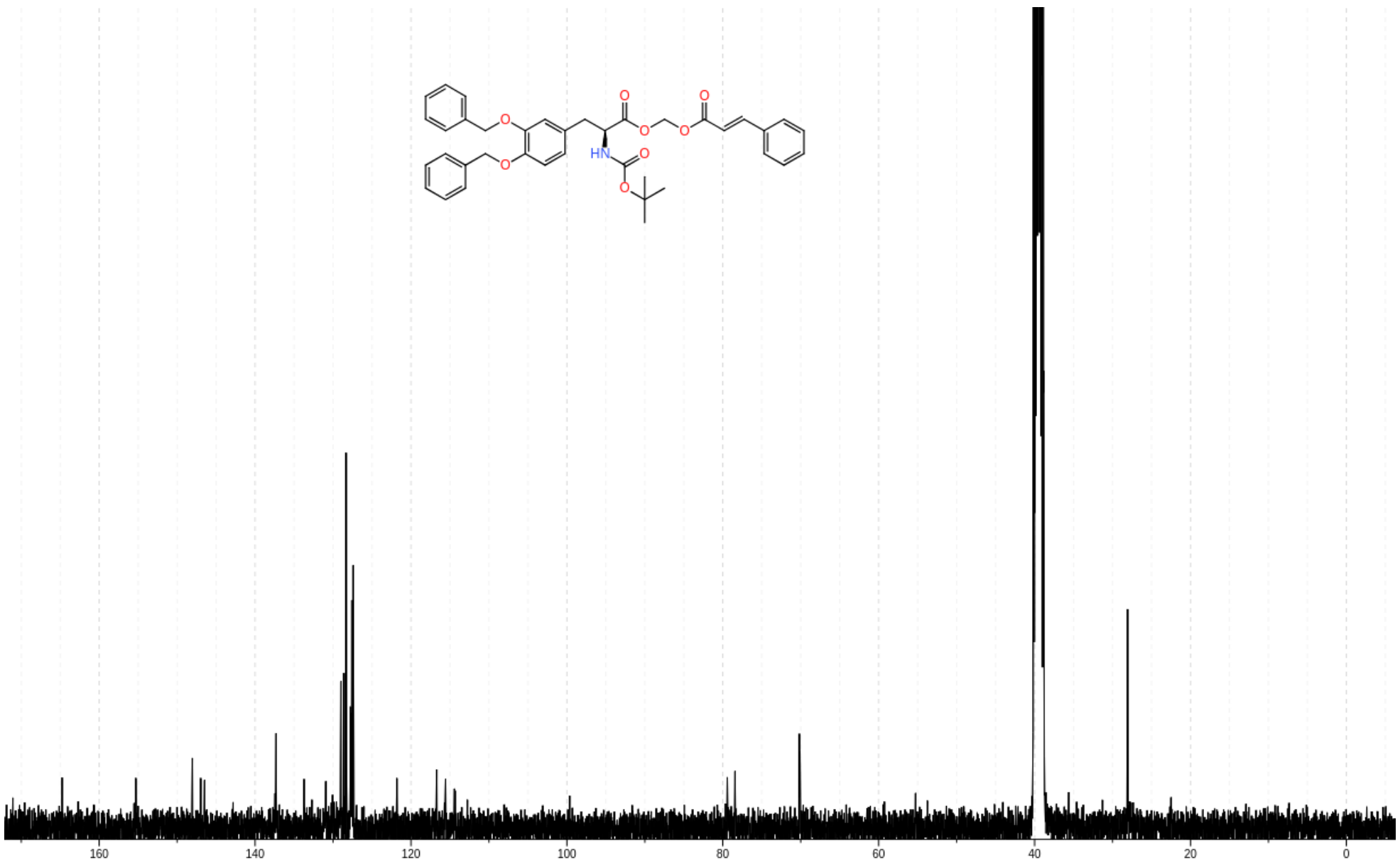

Compound 6d

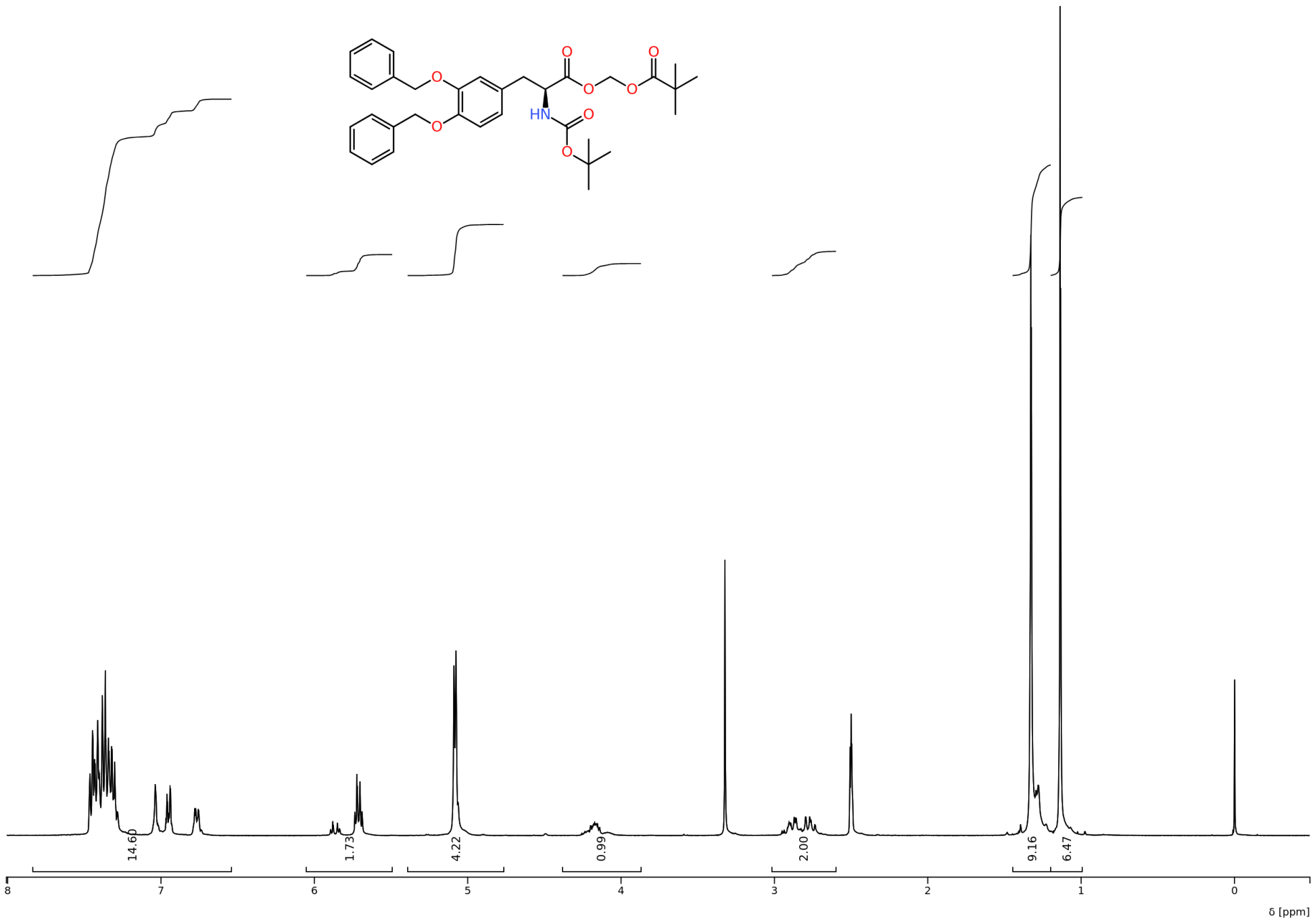

14.60
1.73
4.22
0.99
2.00
9.16
6.47
8
7
6
5
4
3
2
1
0
δ [ppm]

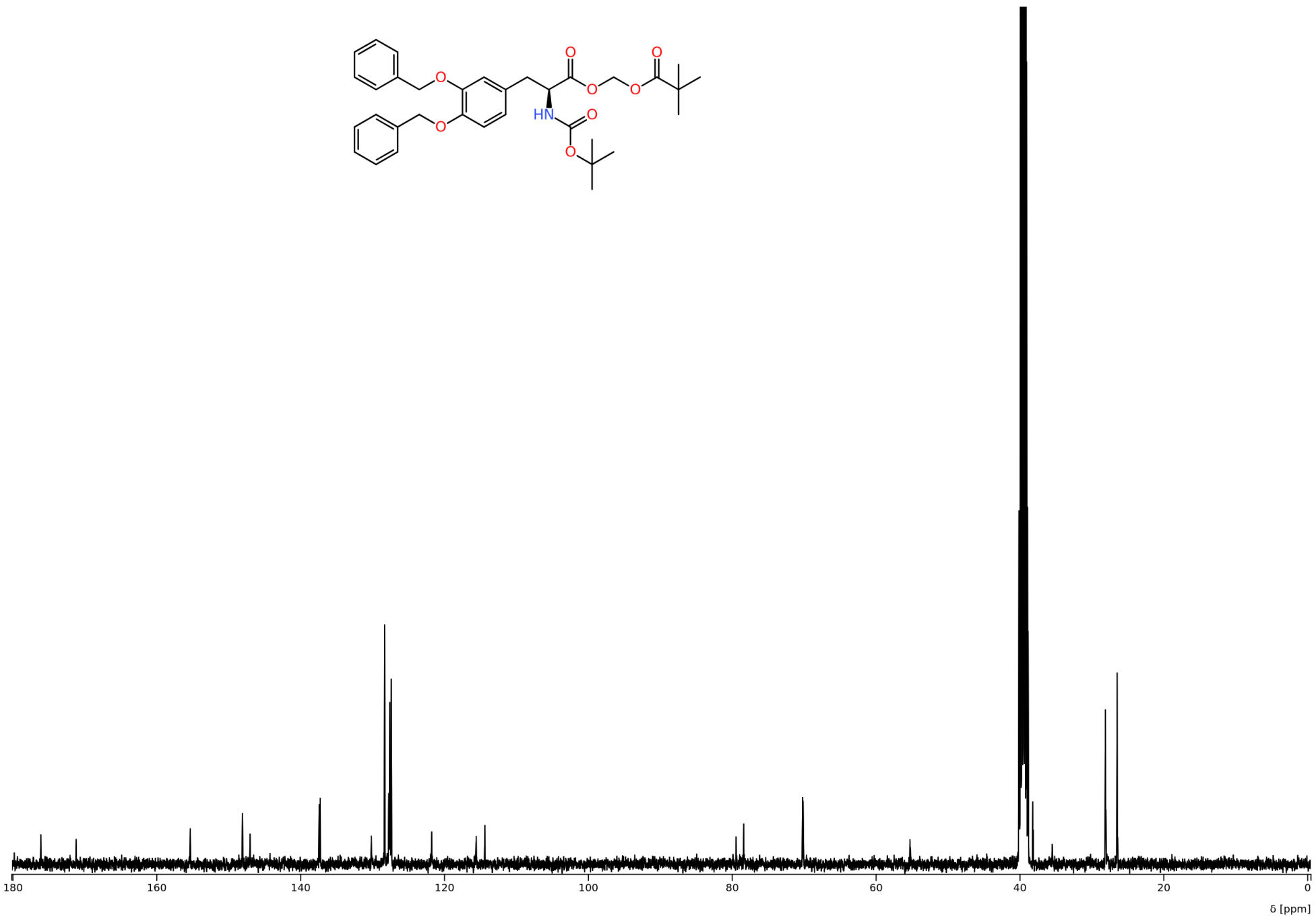

180
160
140
120
100
80
60
40
20
0
δ [ppm]

Compound 6e

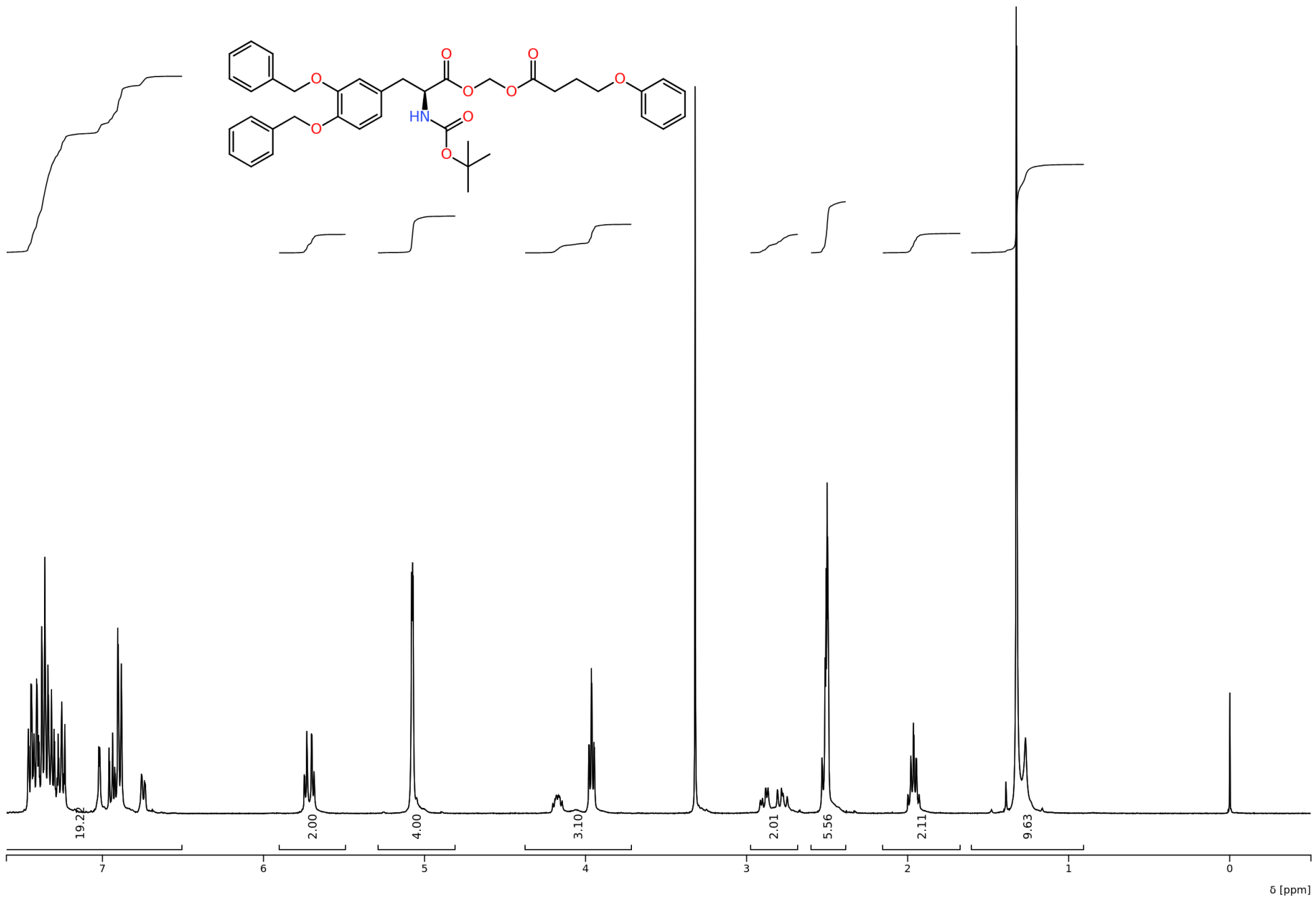

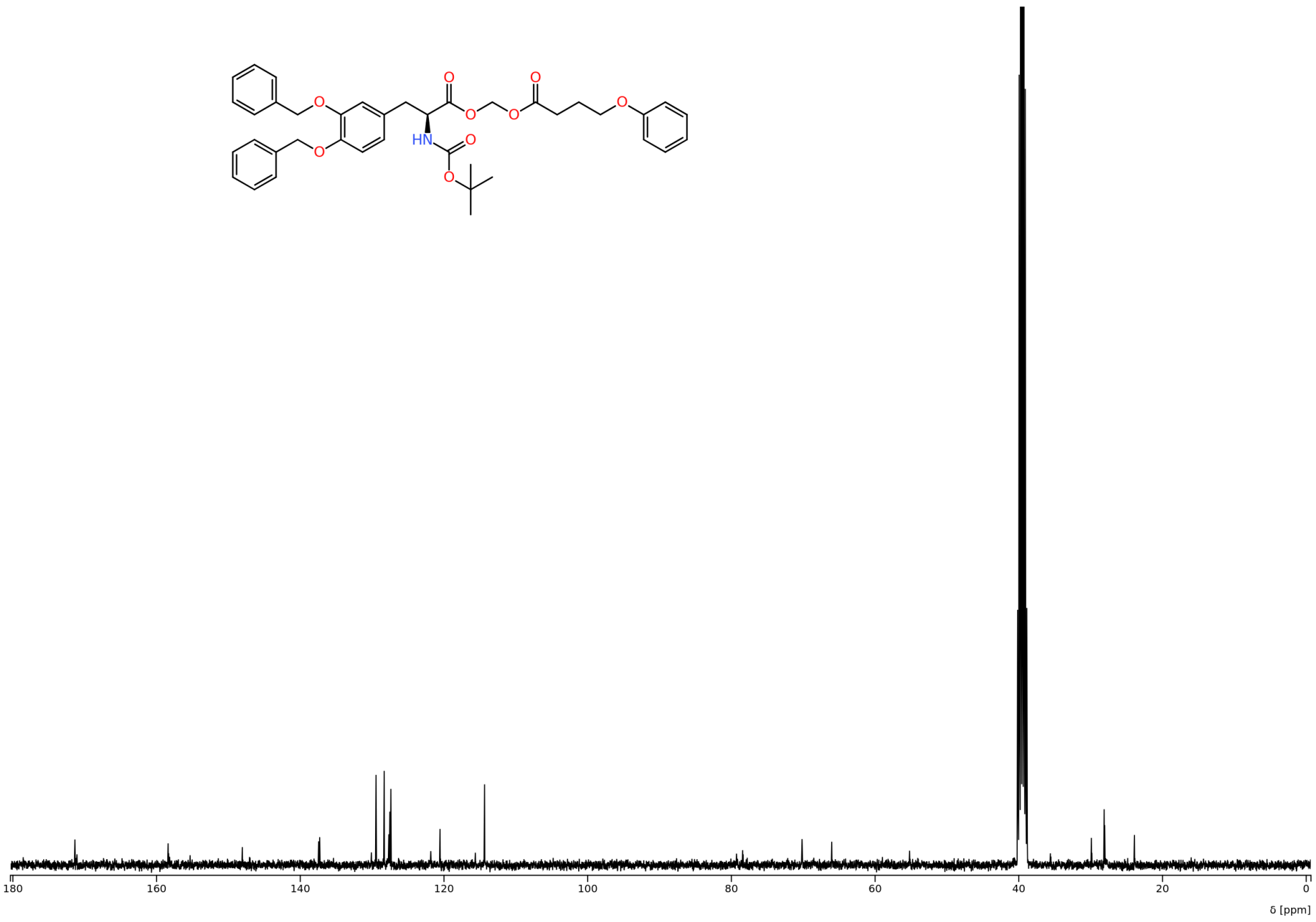

Compound 6f

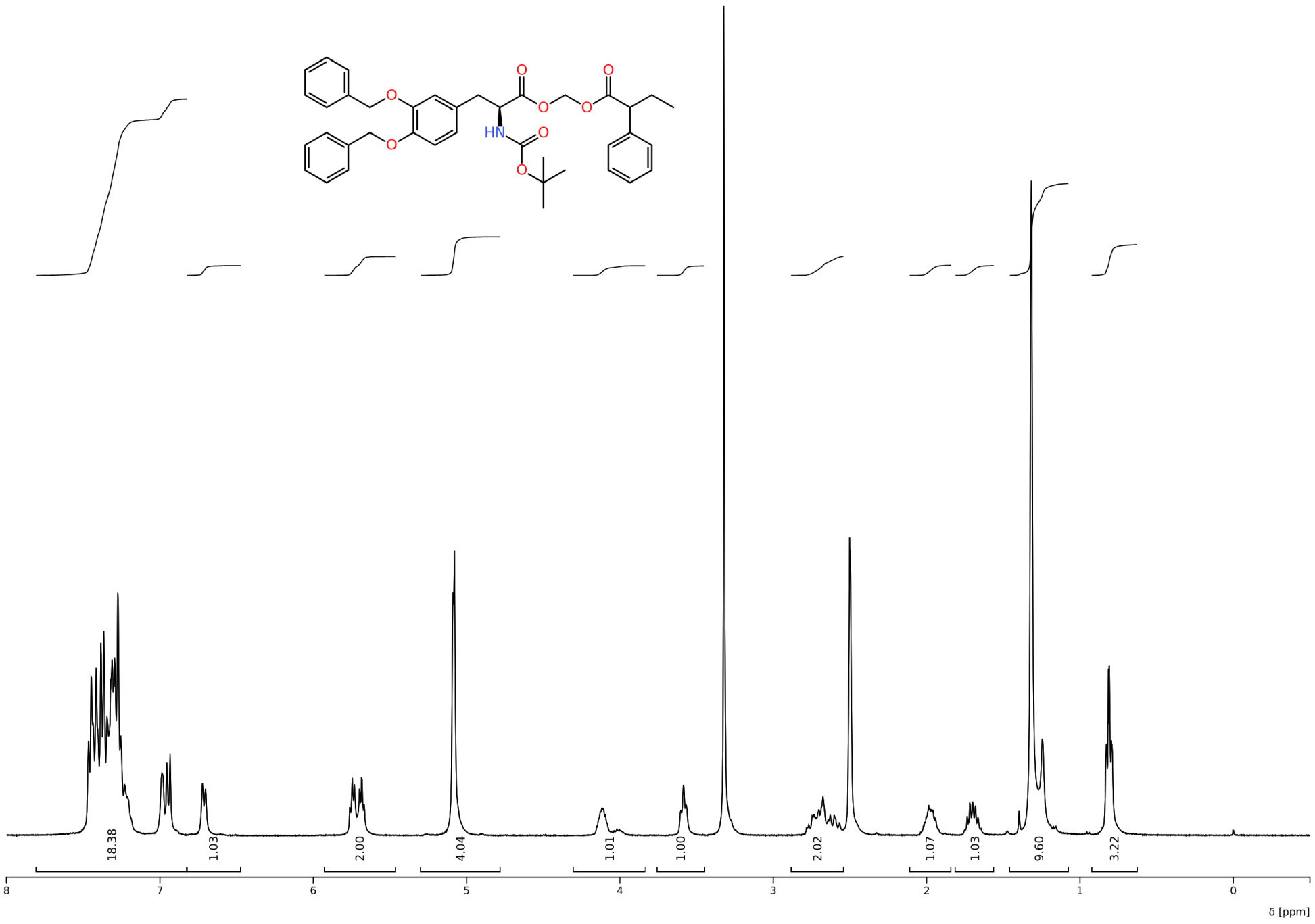

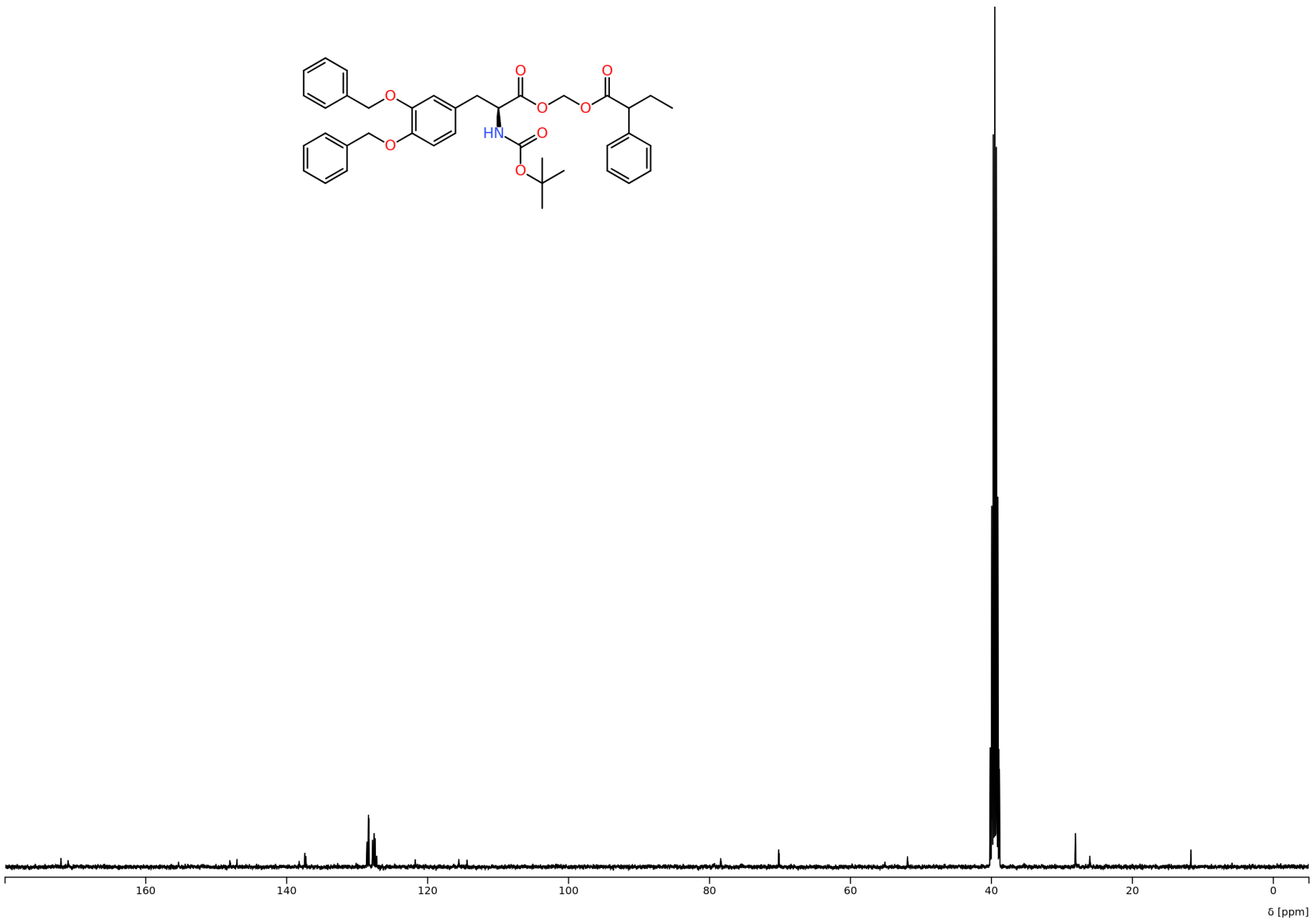

Compound 6g

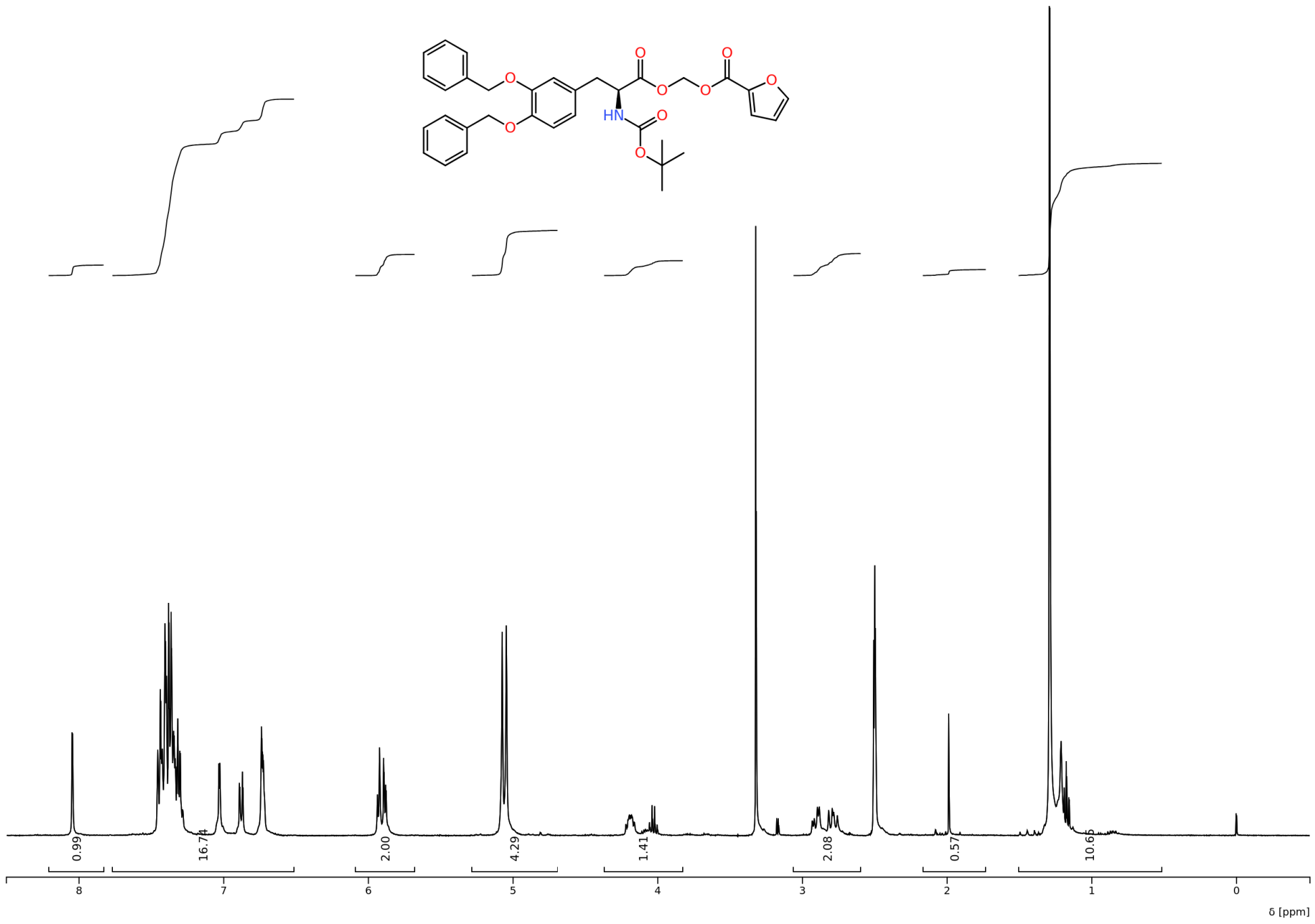

O
O
O
O
O
O
HN
O
O
0.99
16.74
2.00
4.29
1.41
2.08
0.57
10.65
8
7
6
5
4
3
2
1
0
δ [ppm]

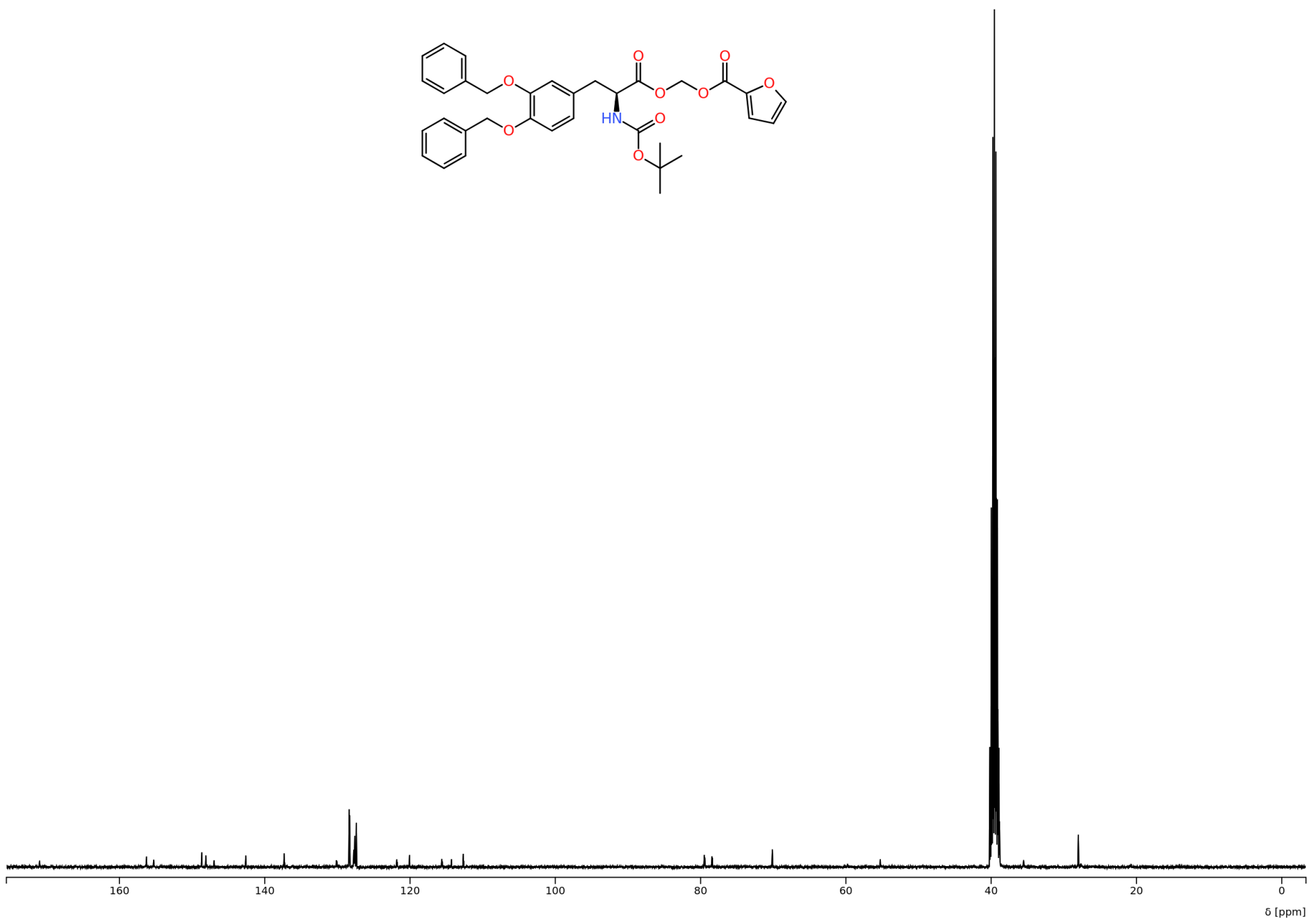

O
O
O
O
O
O
HN
O
O
160
140
120
100
80
60
40
20
0
δ [ppm]

Compound 6h

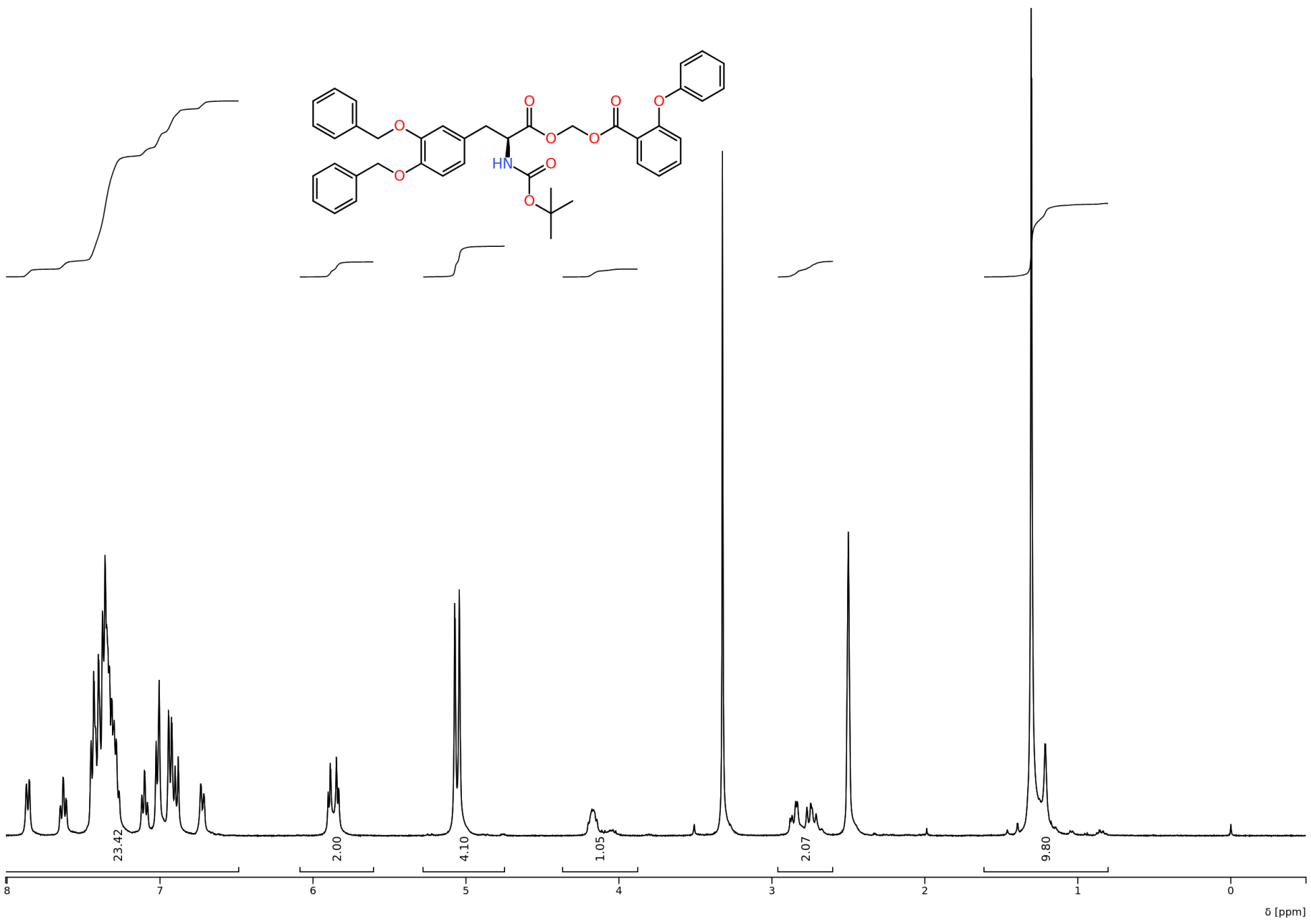

HN
23.42
2.00
4.10
1.05
2.07
9.80
8
7
6
5
4
3
2
1
0
δ [ppm]

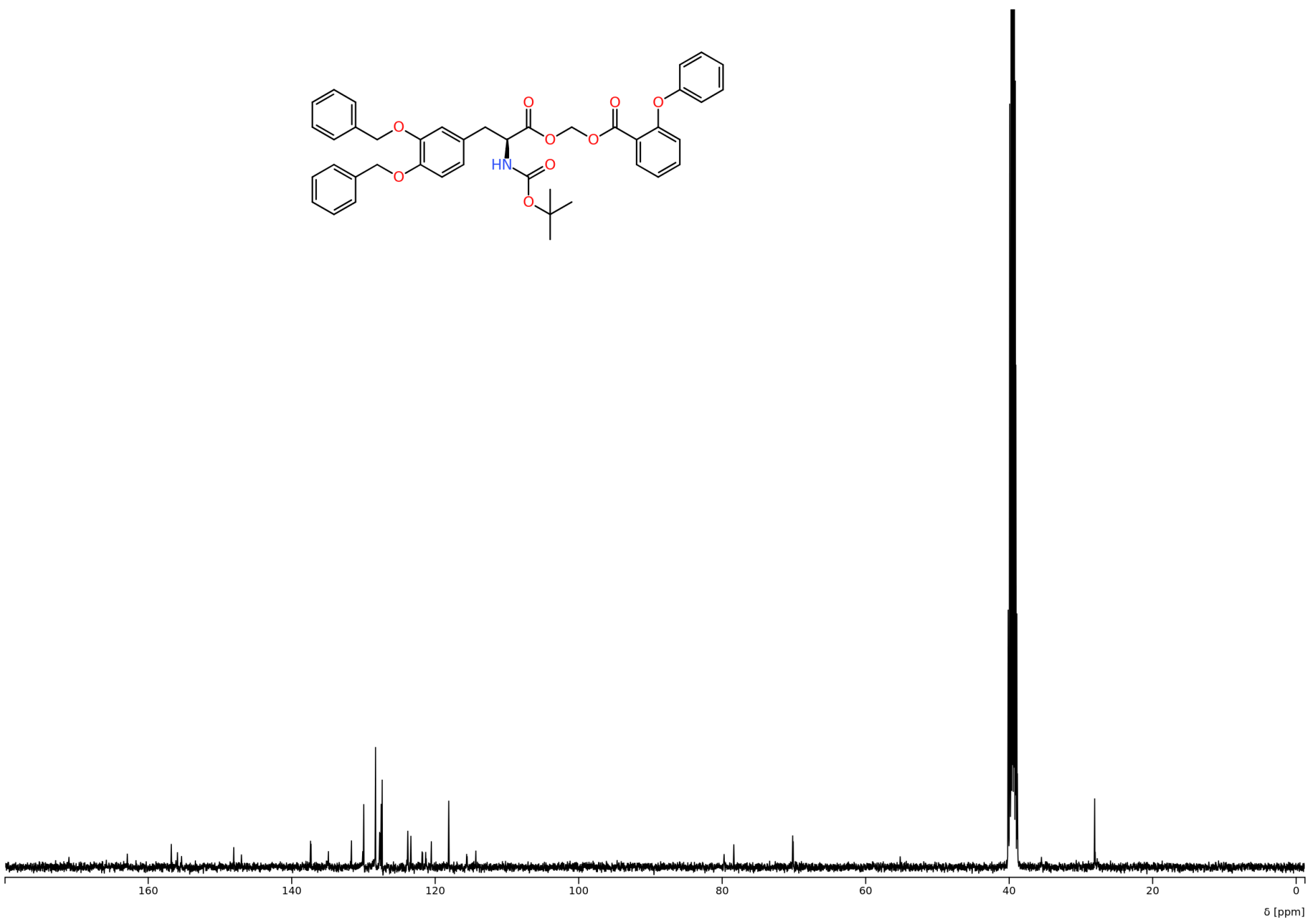

HN
160
140
120
100
80
60
40
20
0
δ [ppm]

Compound 6i

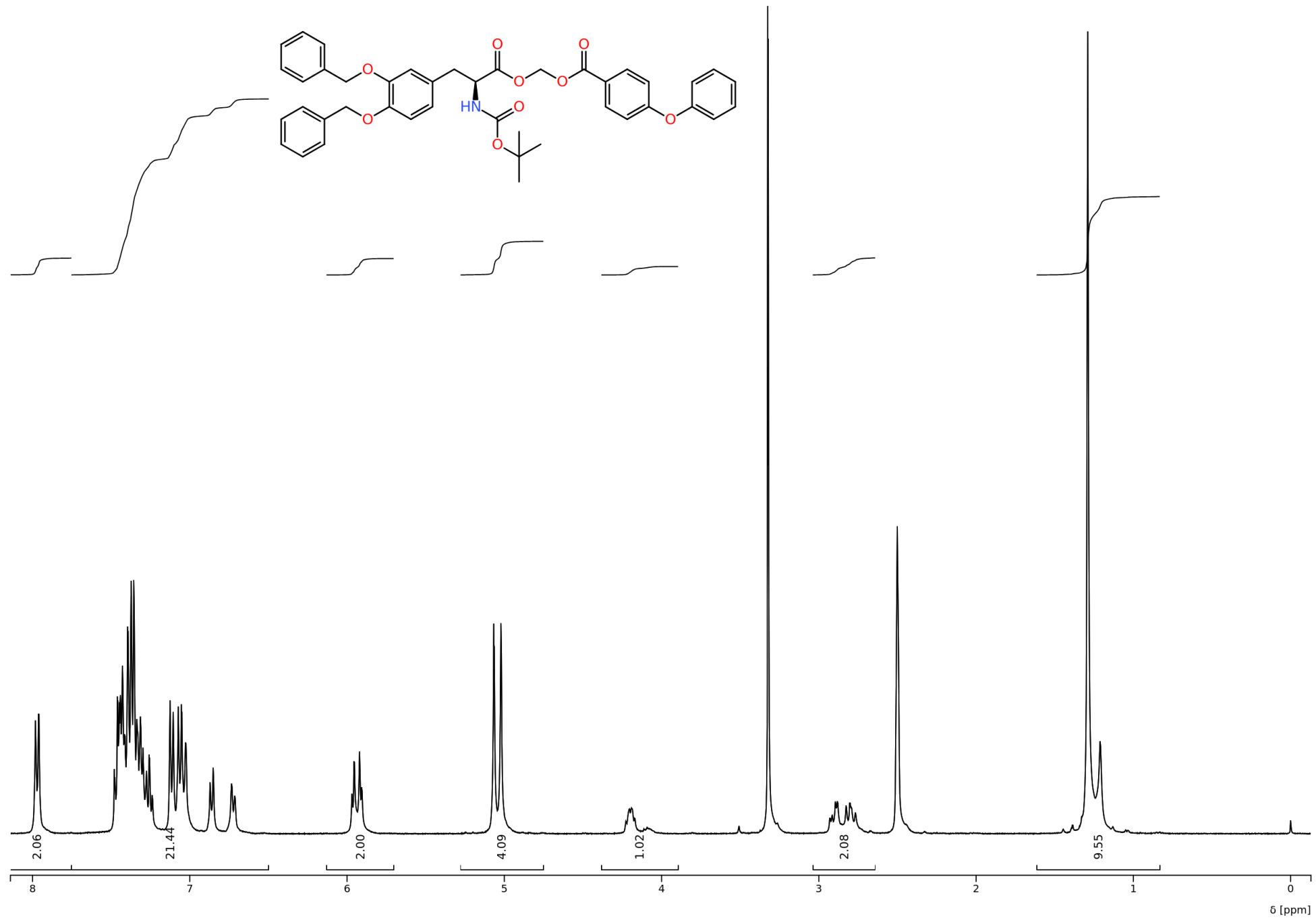
O
O
O
O
HN
O
O
O
O
2.06
21.44
2.00
4.09
1.02
2.08
9.55
8
7
6
5
4
3
2
1
0
δ [ppm]

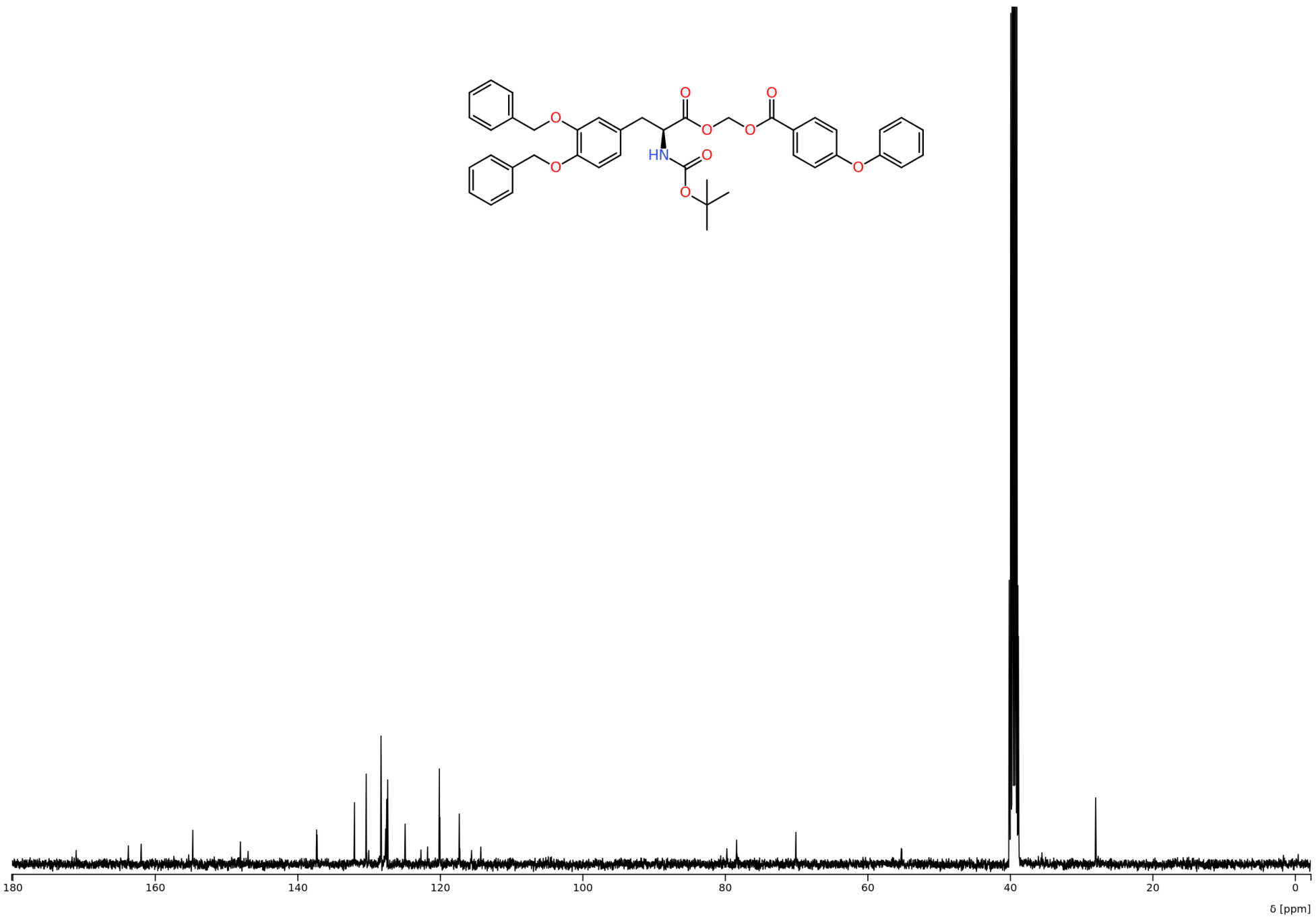
O
O
O
O
HN
O
O
O
O
180
160
140
120
100
80
60
40
20
0
δ [ppm]

Compound 6j

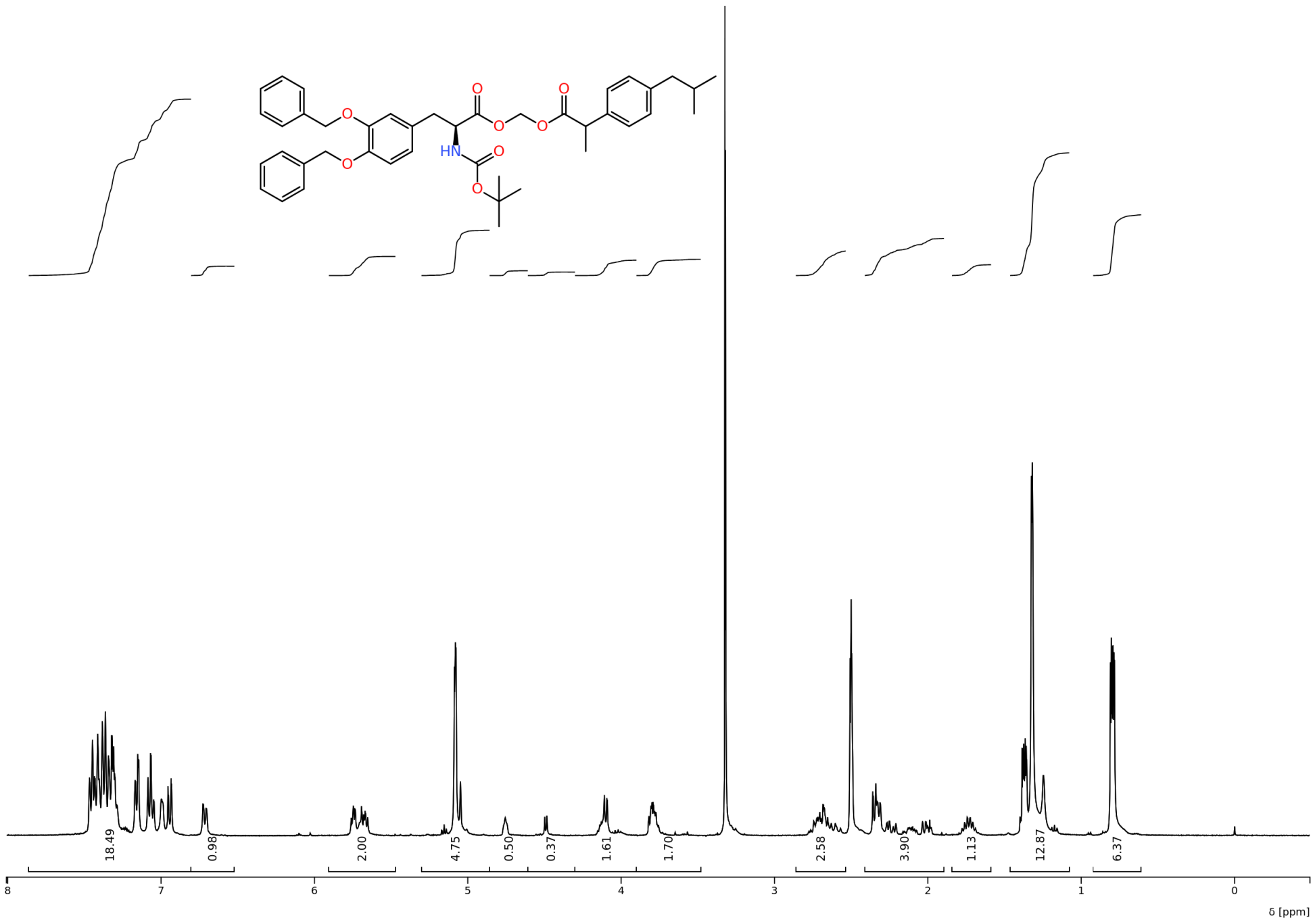

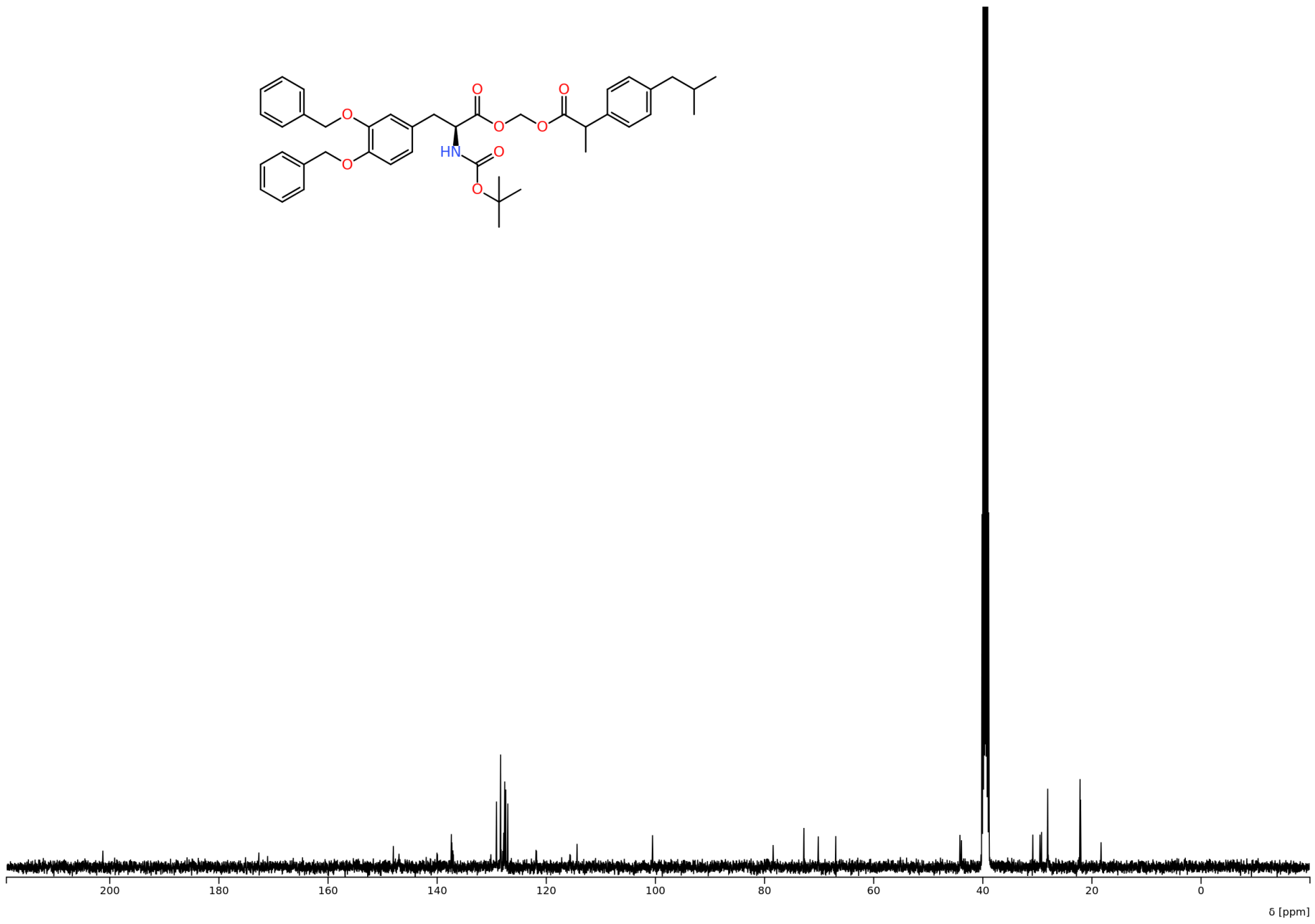

Compound 6k

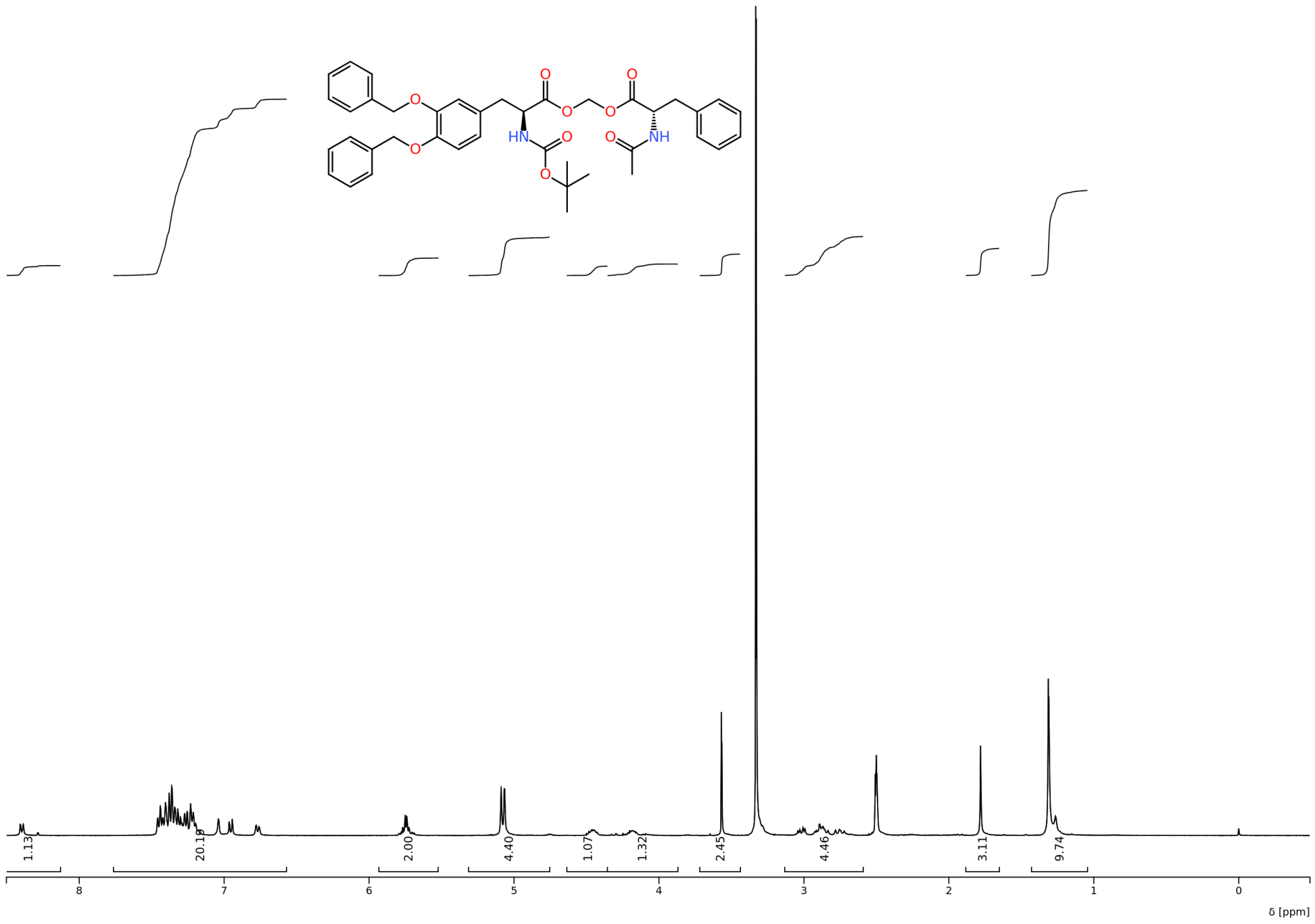
1.13
20.18
2.00
4.40
1.07
1.32
2.45
4.46
3.11
9.74
8
7
6
5
4
3
2
1
0
δ [ppm]

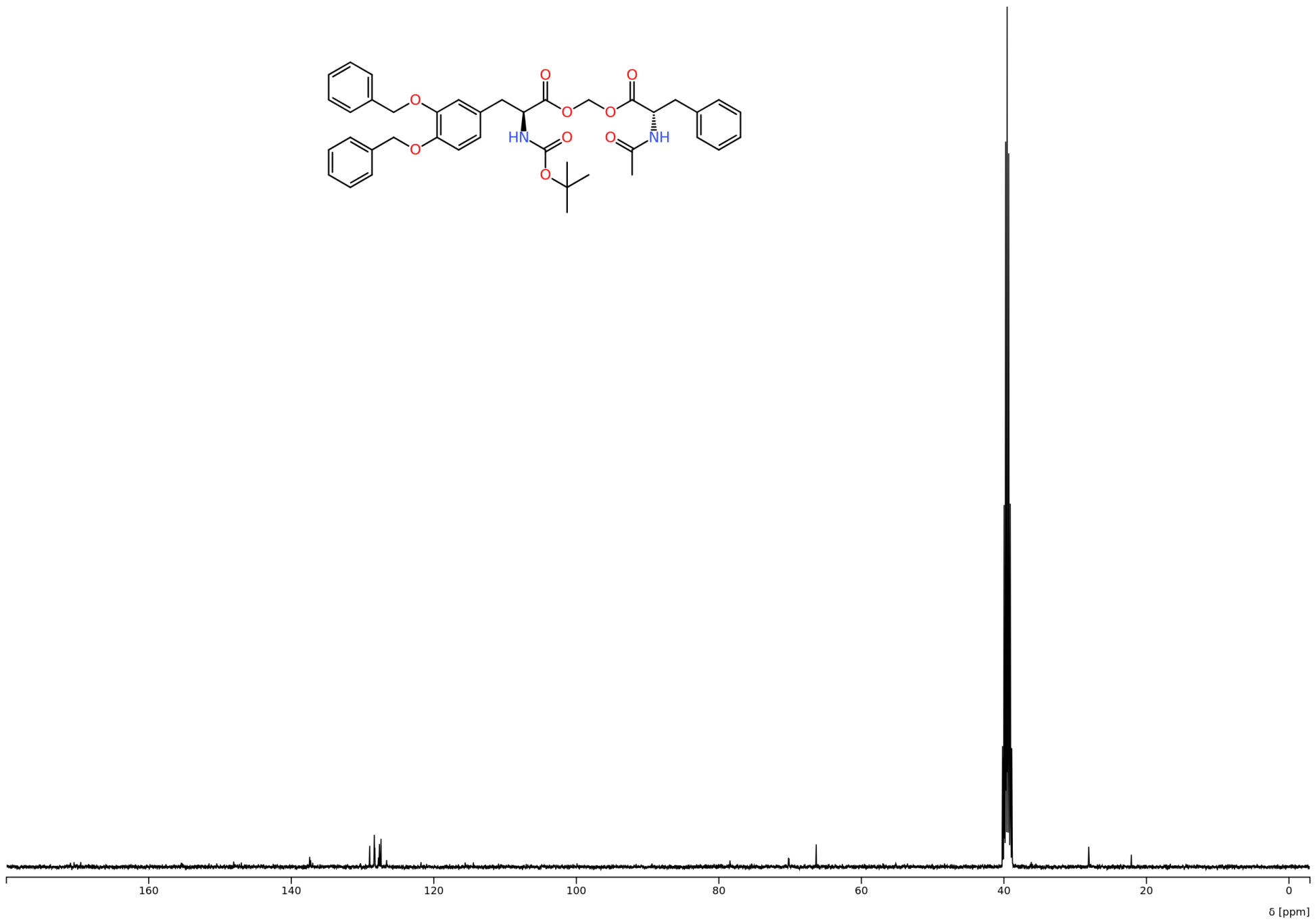
160
140
120
100
80
60
40
20
0
δ [ppm]

Compound 6l

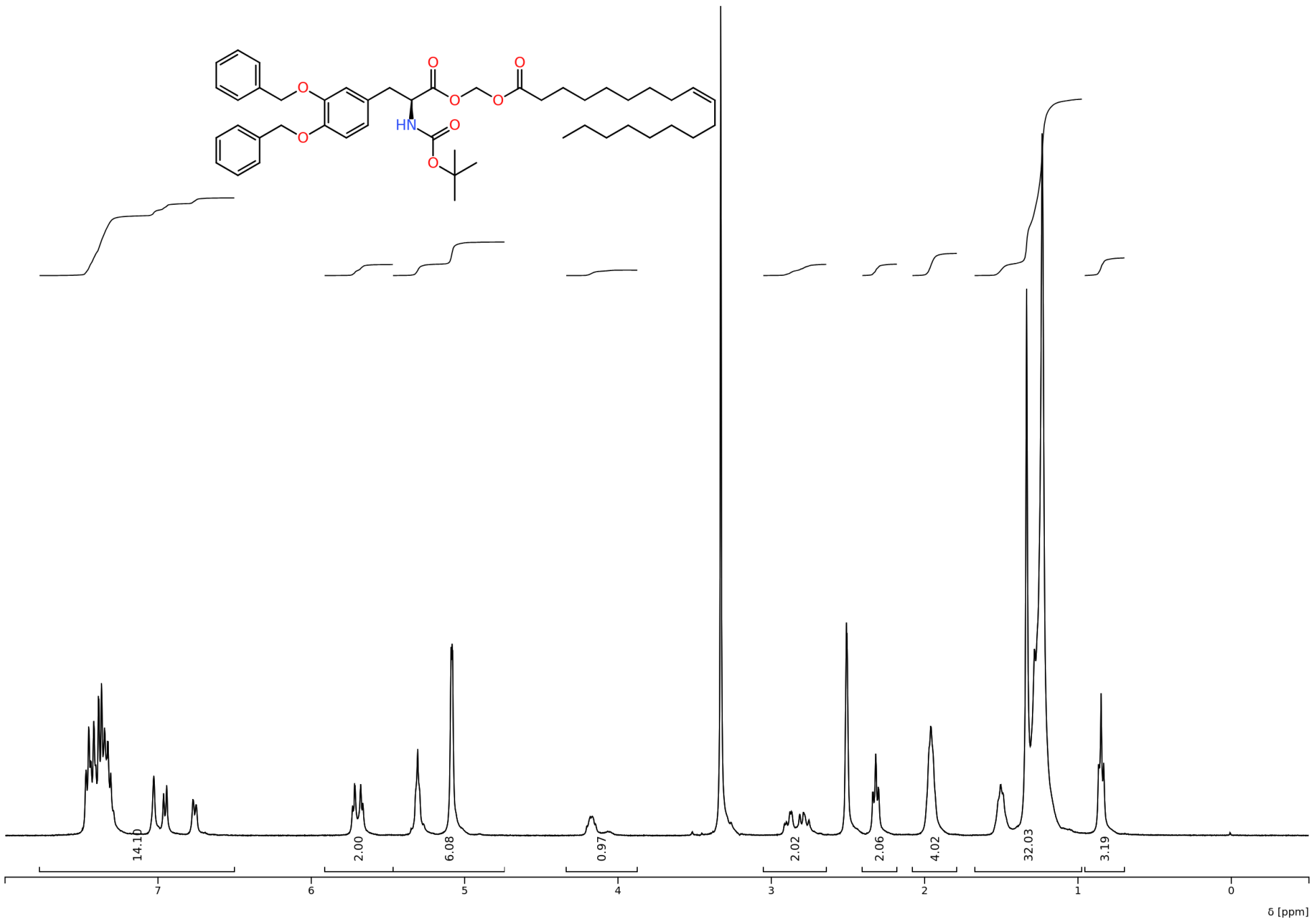

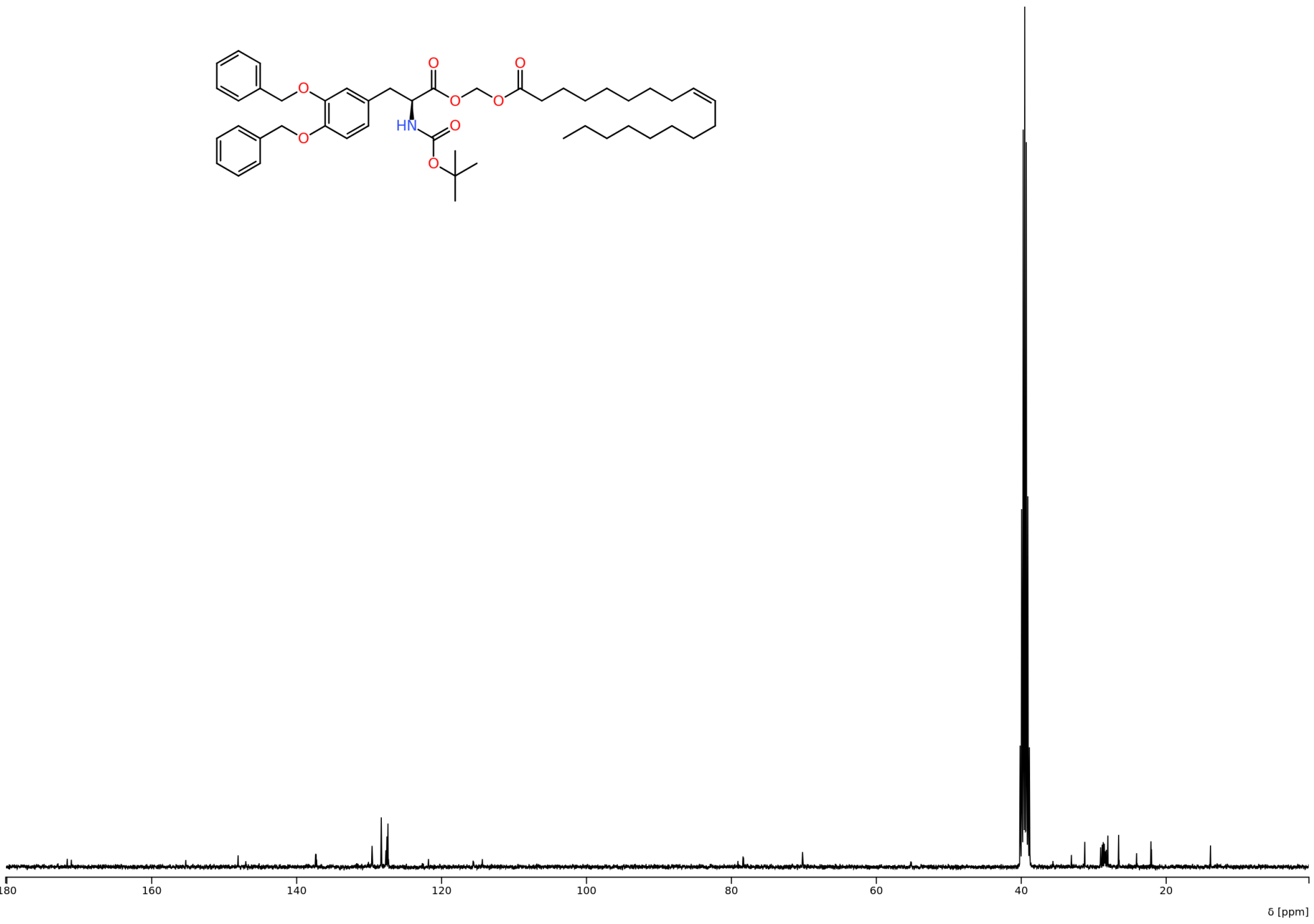

Compound 6m

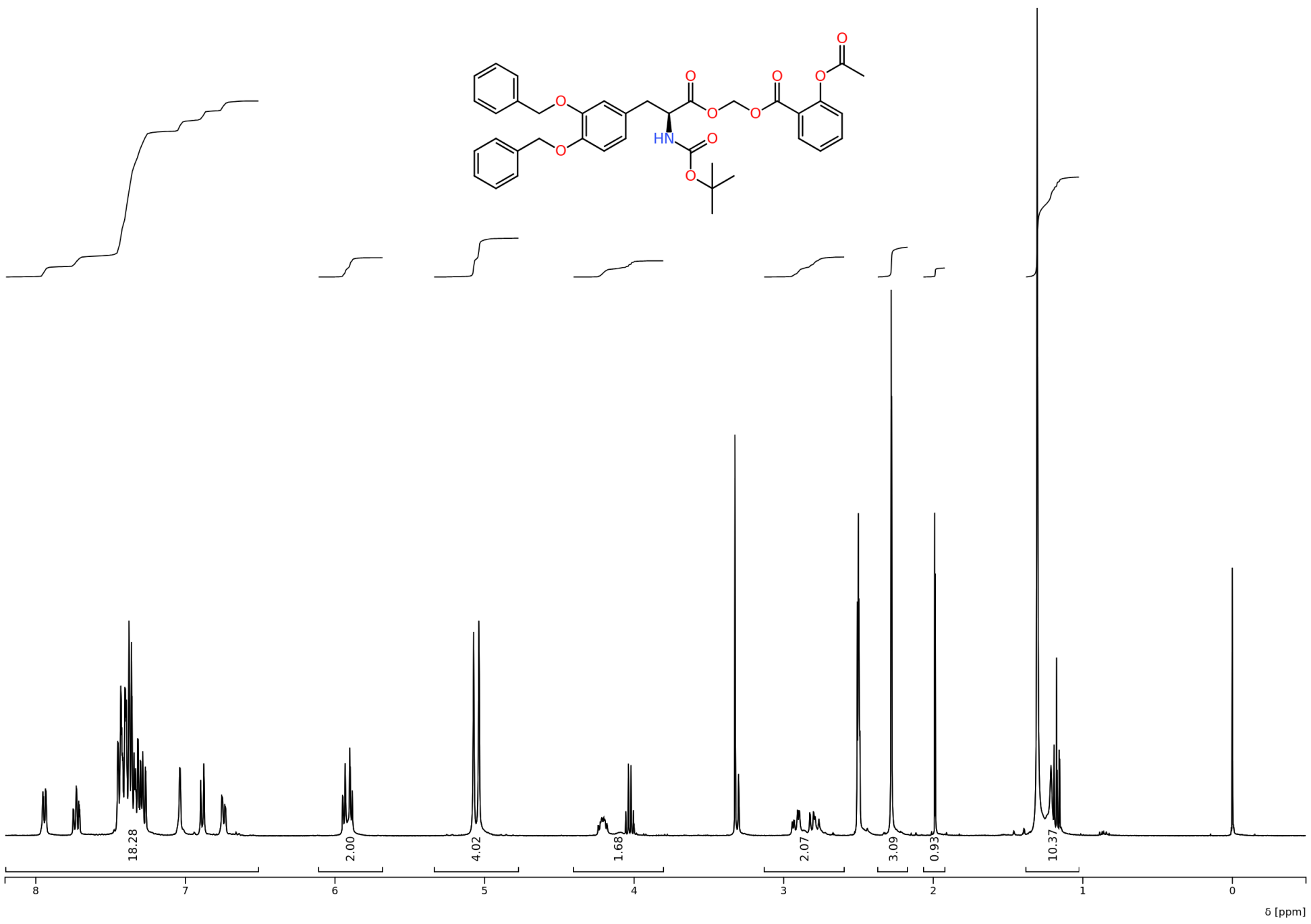
HN
18.28
2.00
4.02
1.68
2.07
3.09
0.93
10.37
8
7
6
5
4
3
2
1
0
δ [ppm]

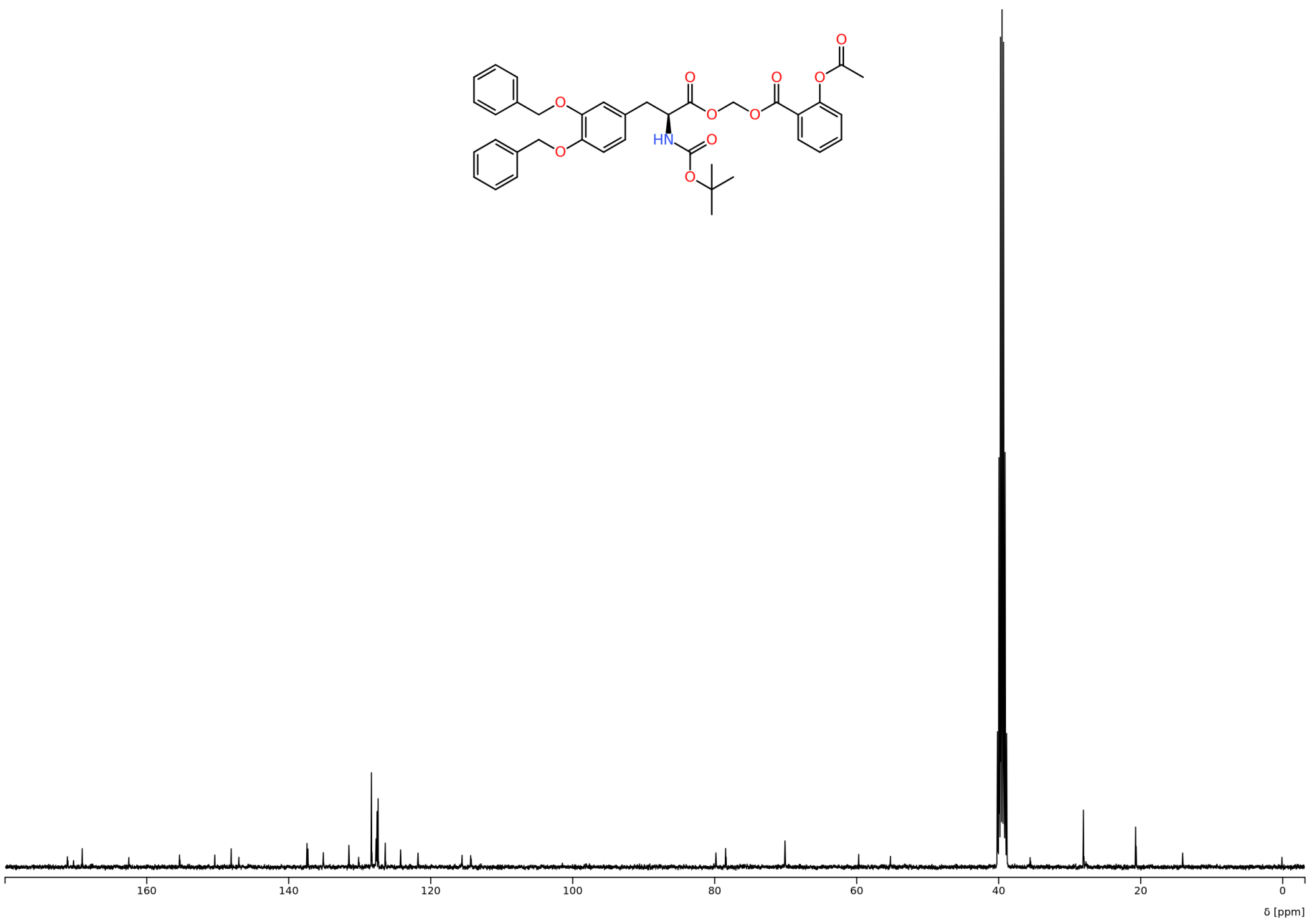
HN
160
140
120
100
80
60
40
20
0
δ [ppm]

Compound 6n

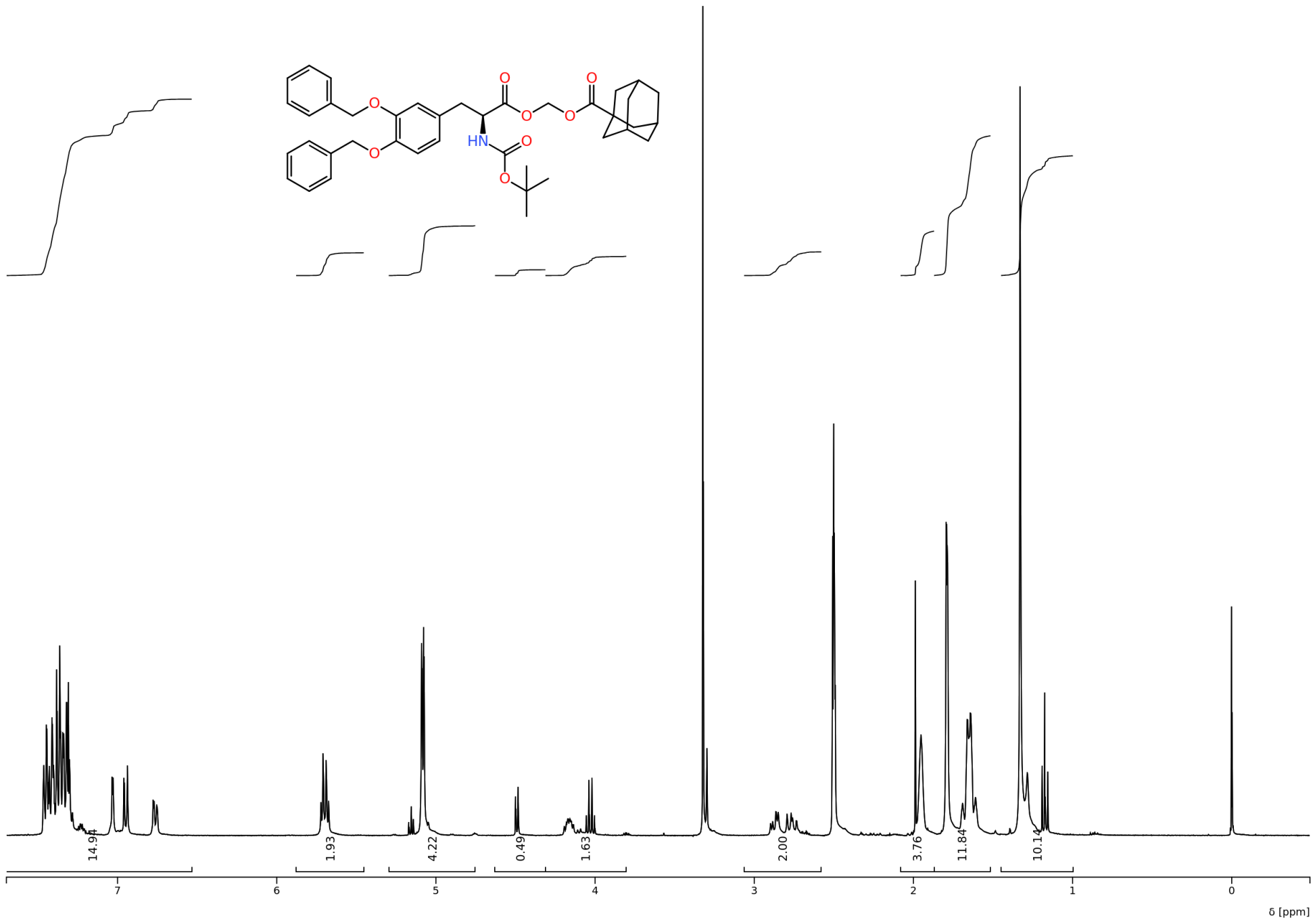

O
O
O
O
O
HN
O
O
14.94
1.93
4.22
0.49
1.63
2.00
3.76
11.84
10.14
7
6
5
4
3
2
1
0
δ [ppm]

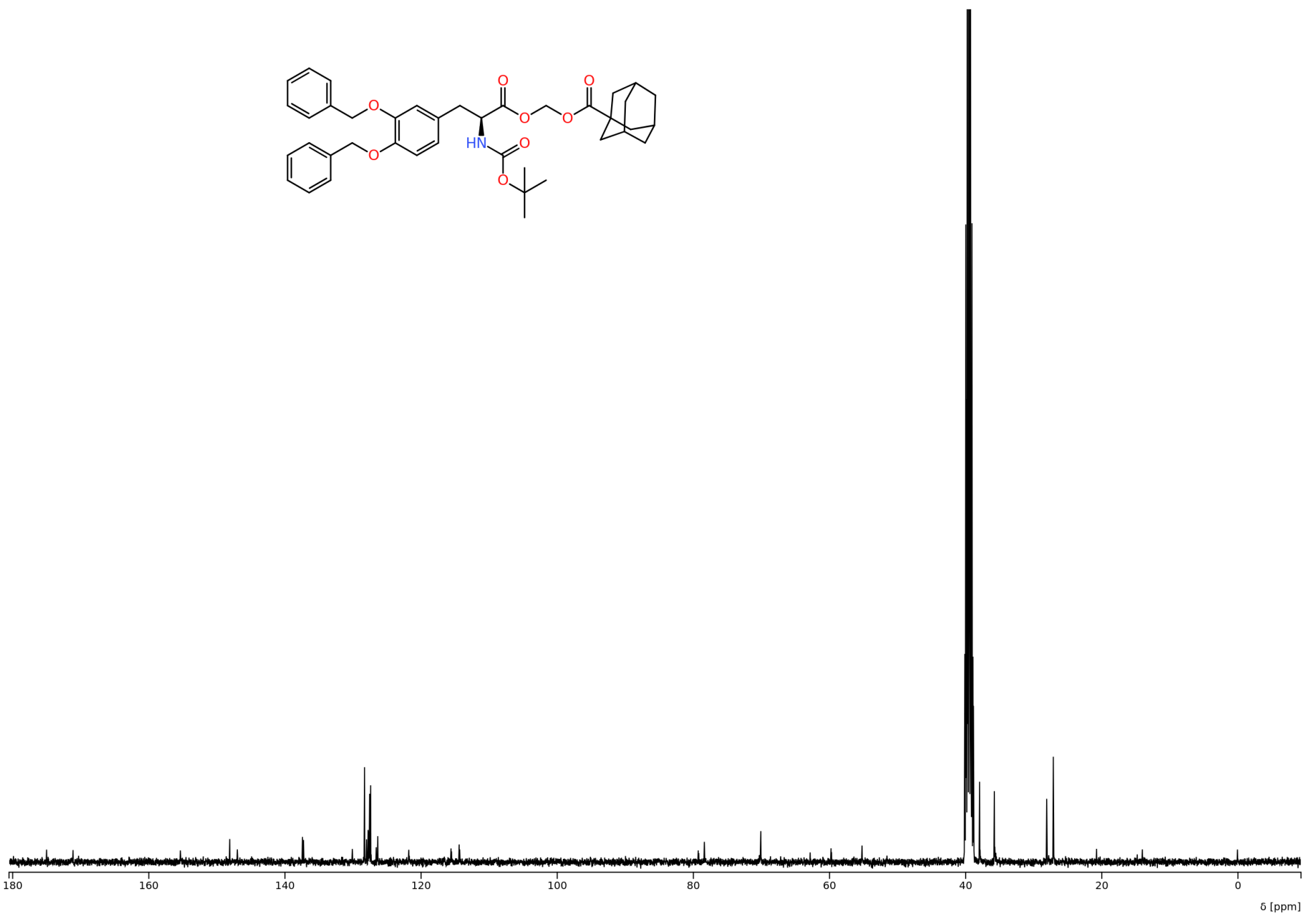

O
O
O
O
O
HN
O
O
180
160
140
120
100
80
60
40
20
0
δ [ppm]

Compound 6o

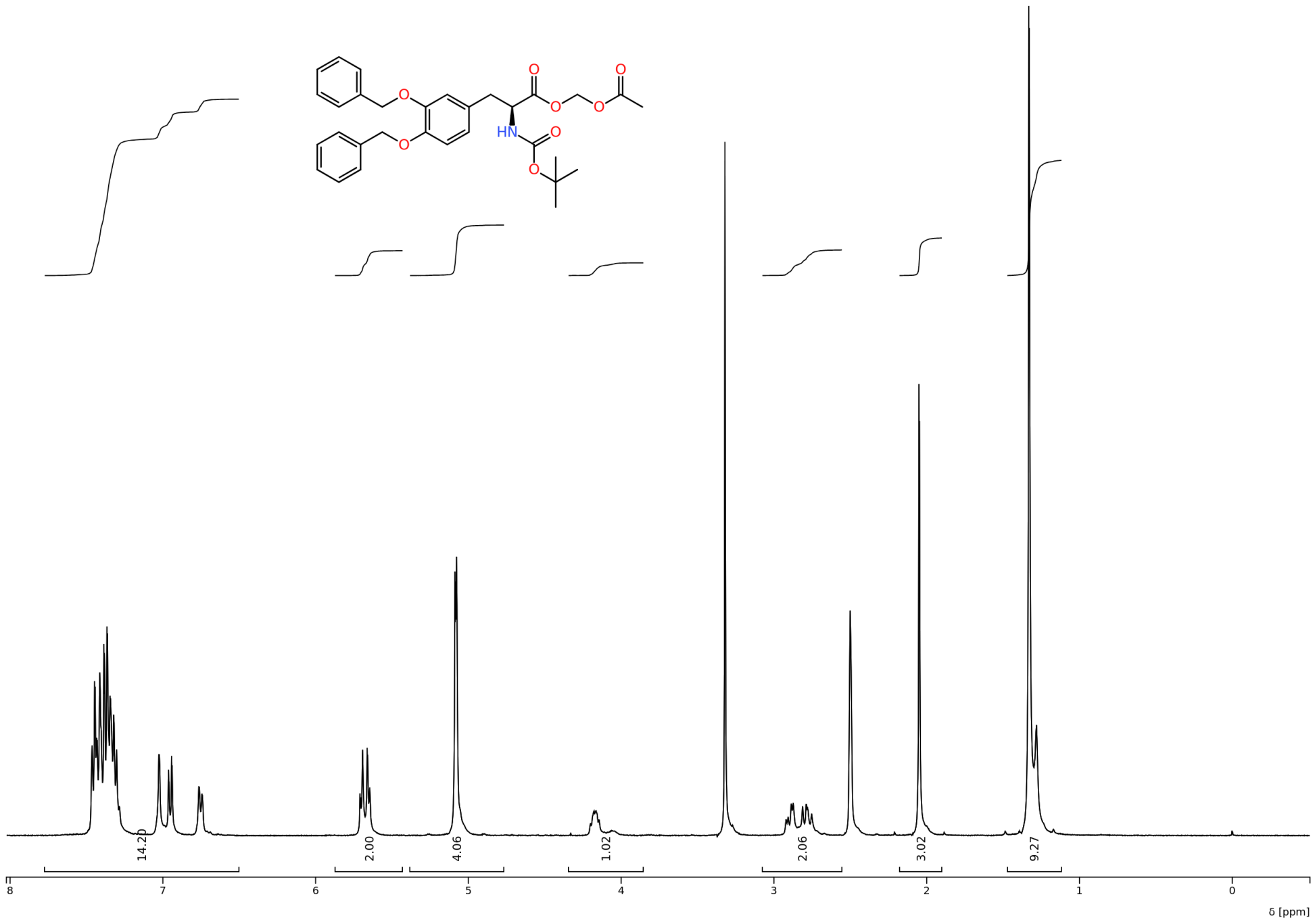

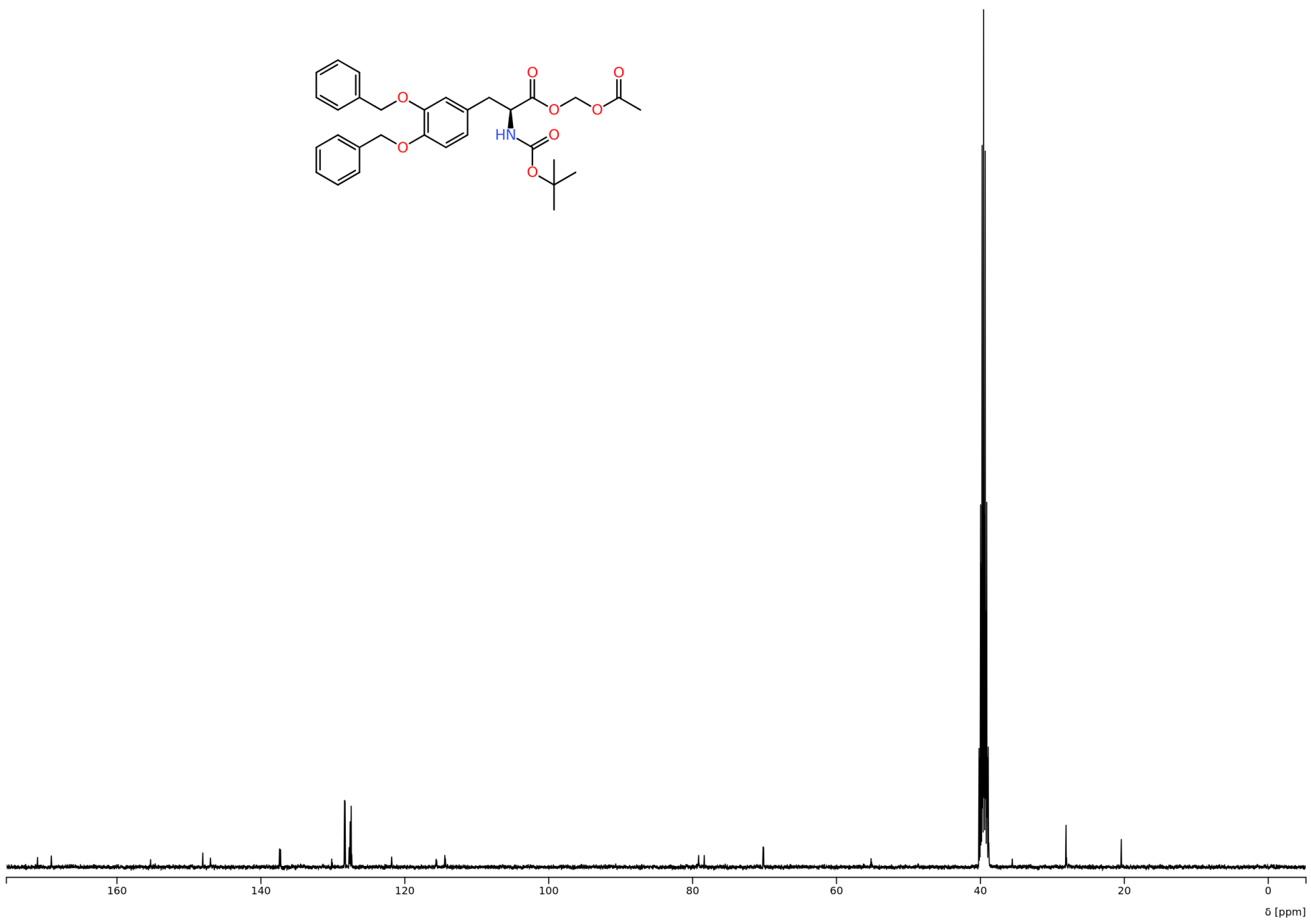

Compound 7a

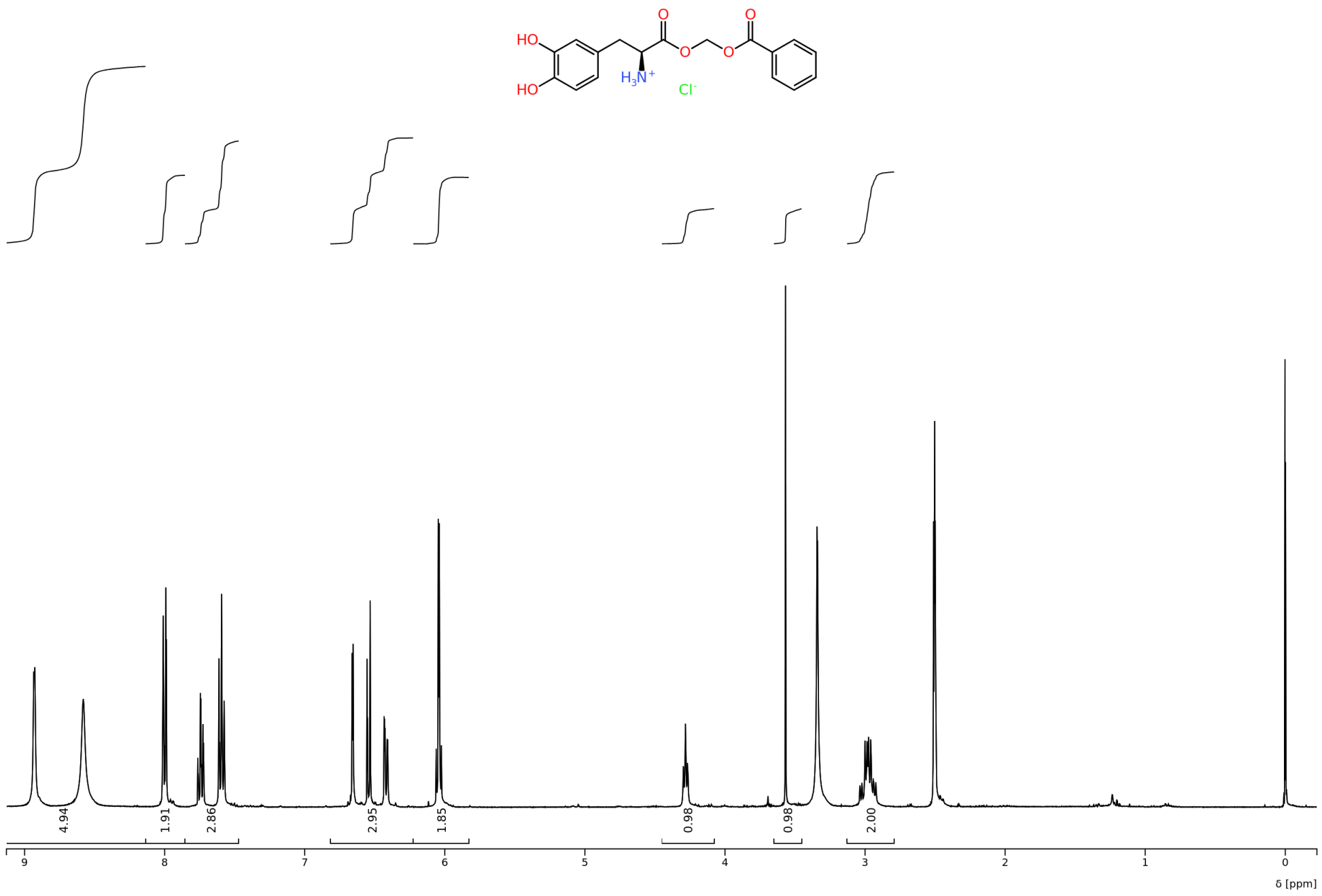

HO
HO
O
O
O
O
H3N+
Cl-
4.94
1.91
2.86
2.95
1.85
0.98
0.98
2.00
δ [ppm]

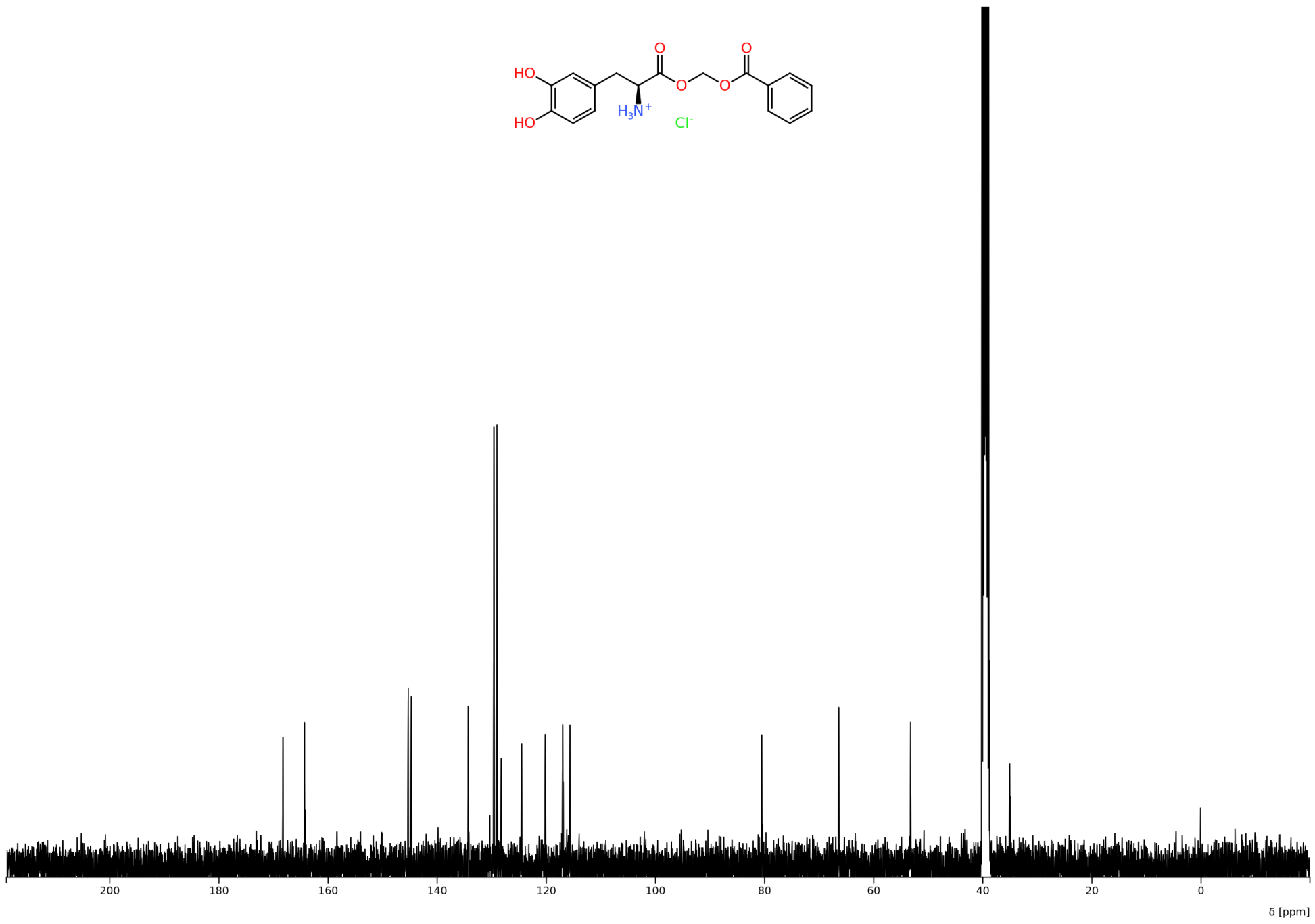

HO
HO
O
O
O
O
H3N+
Cl-
δ [ppm]

Compound 7b

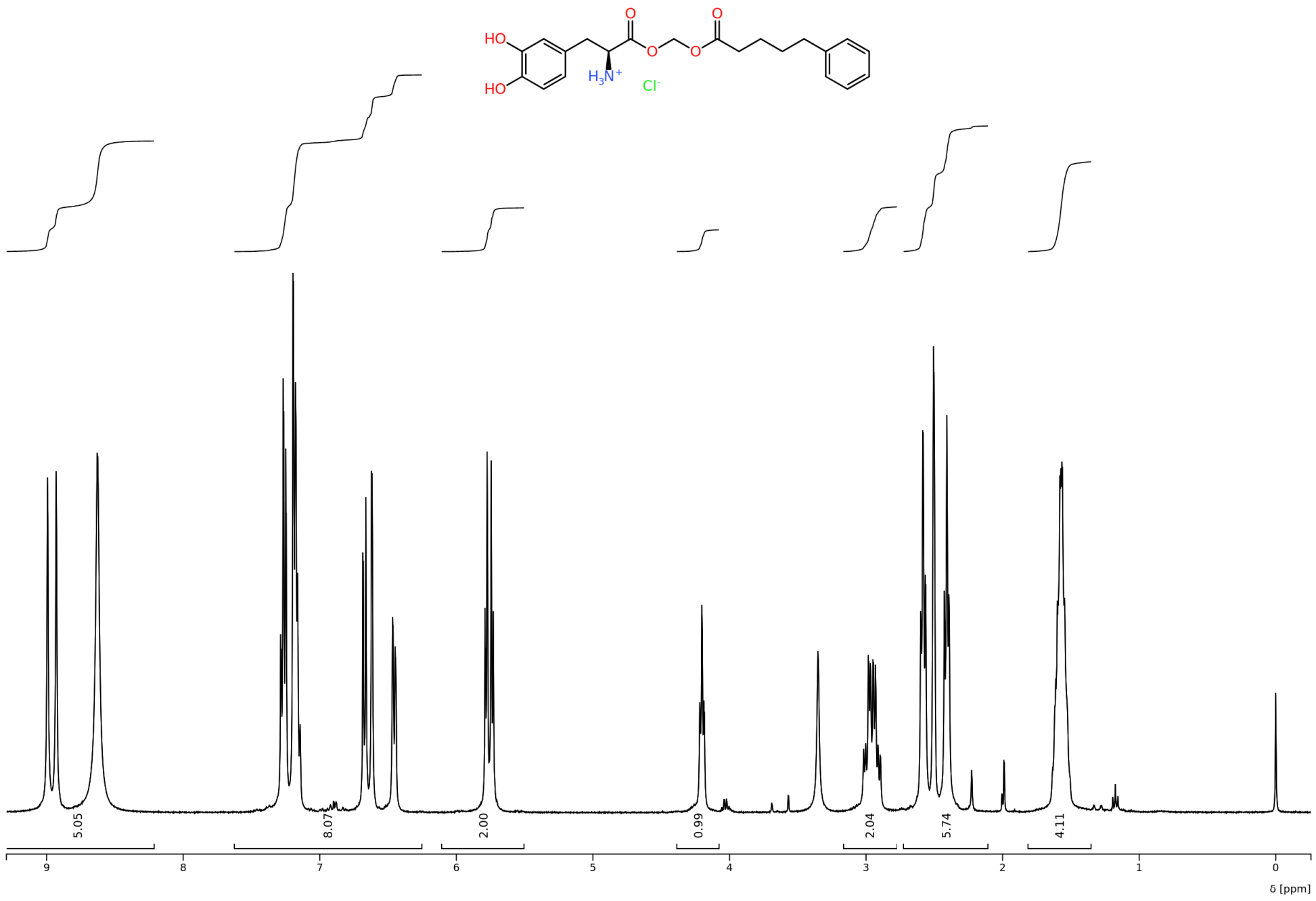
HO
HO
O
O
H3N+
Cl-
5.05
8.07
2.00
0.99
2.04
5.74
4.11
9
8
7
6
5
4
3
2
1
0
δ [ppm]

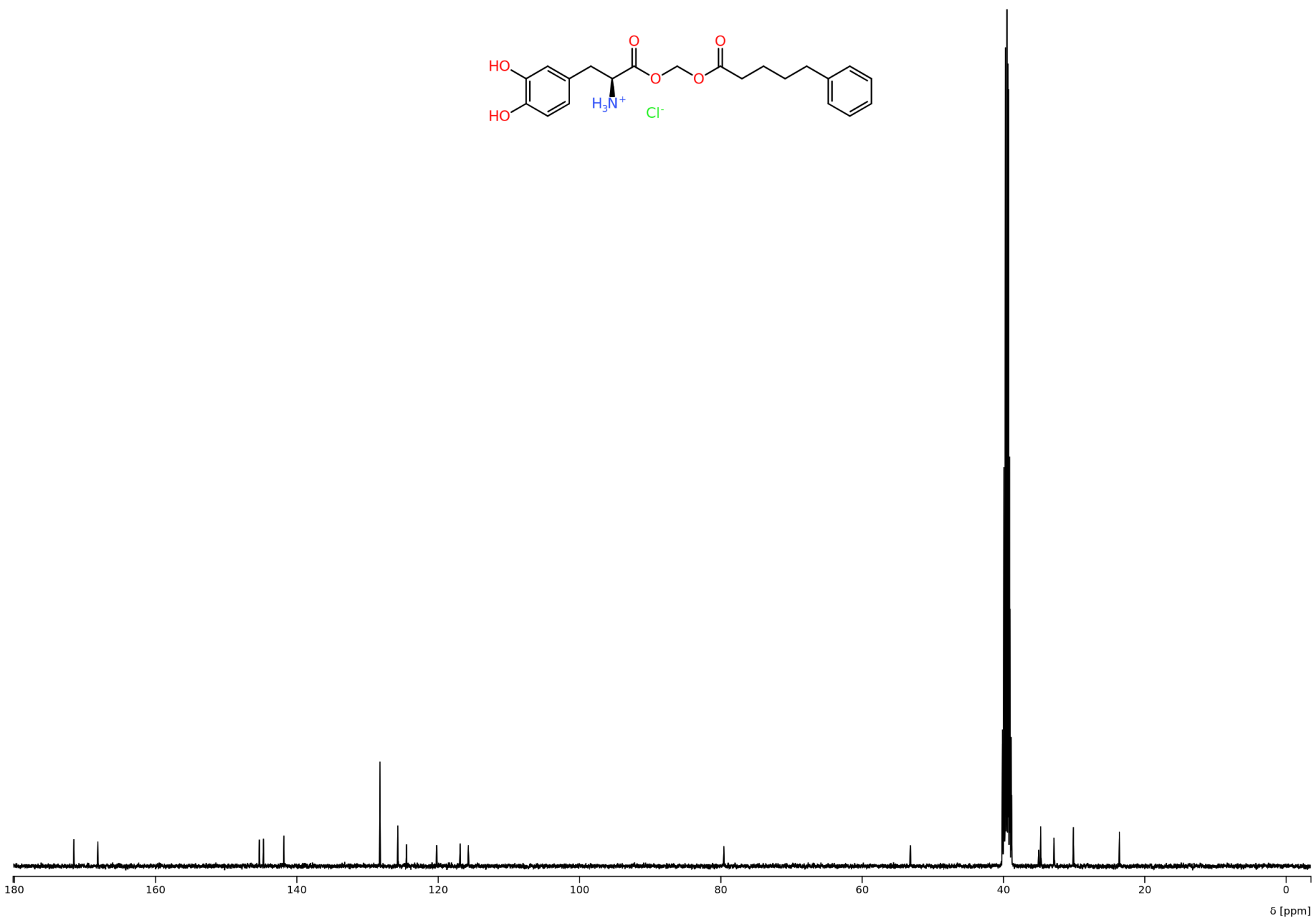
HO
HO
O
O
H3N+
Cl-
180
160
140
120
100
80
60
40
20
0
δ [ppm]

Compound 7c

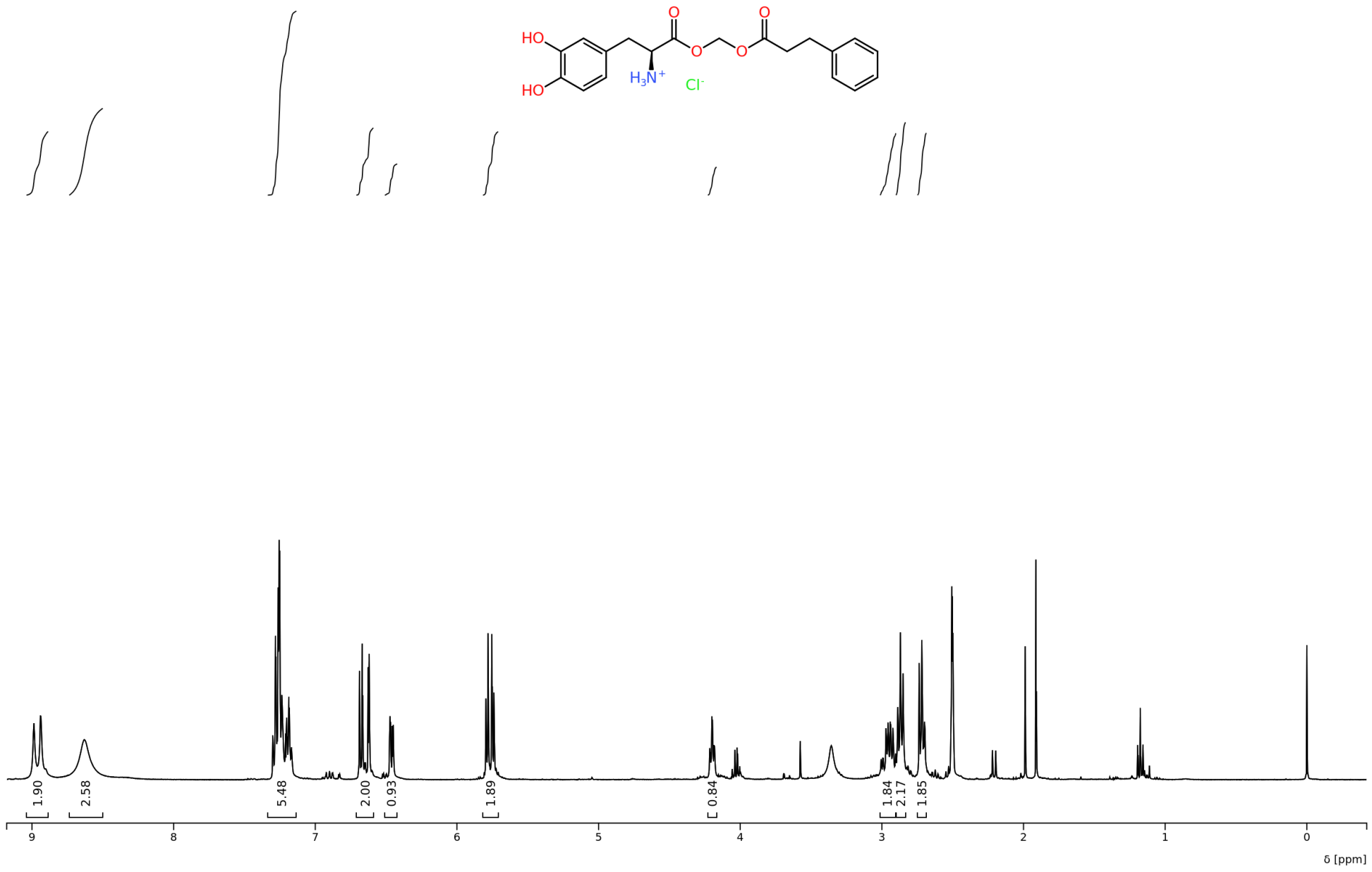

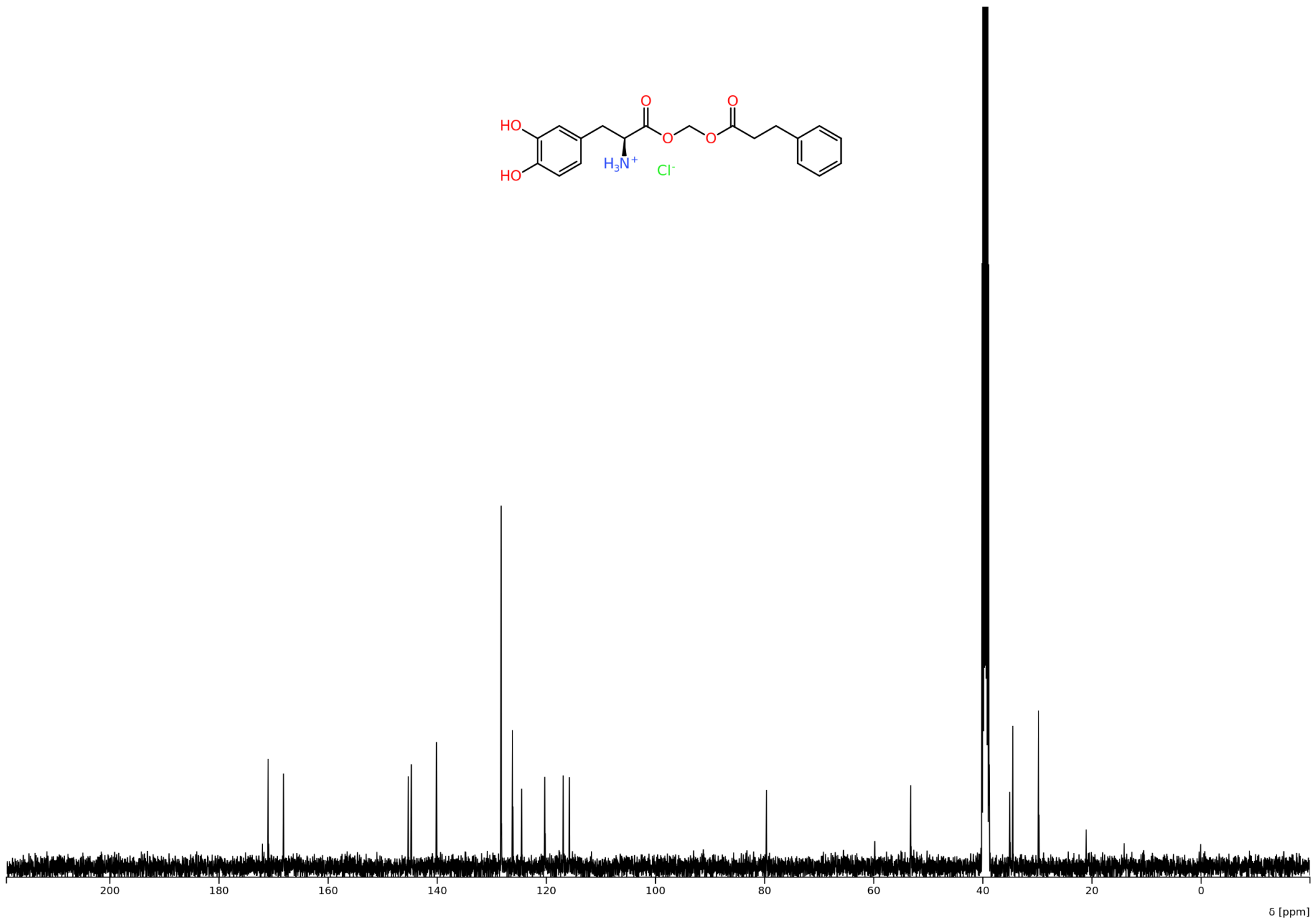

Compound 7d

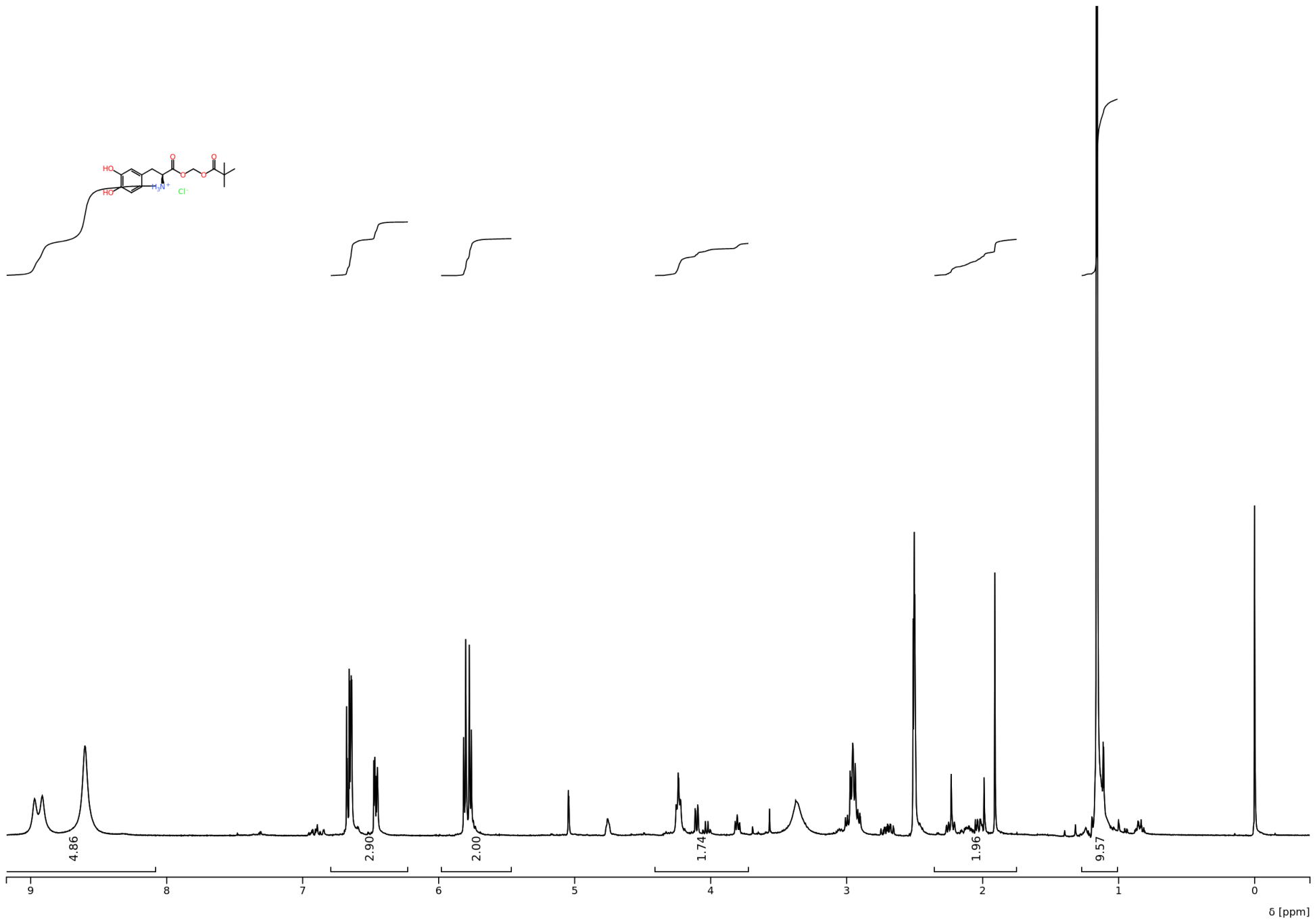

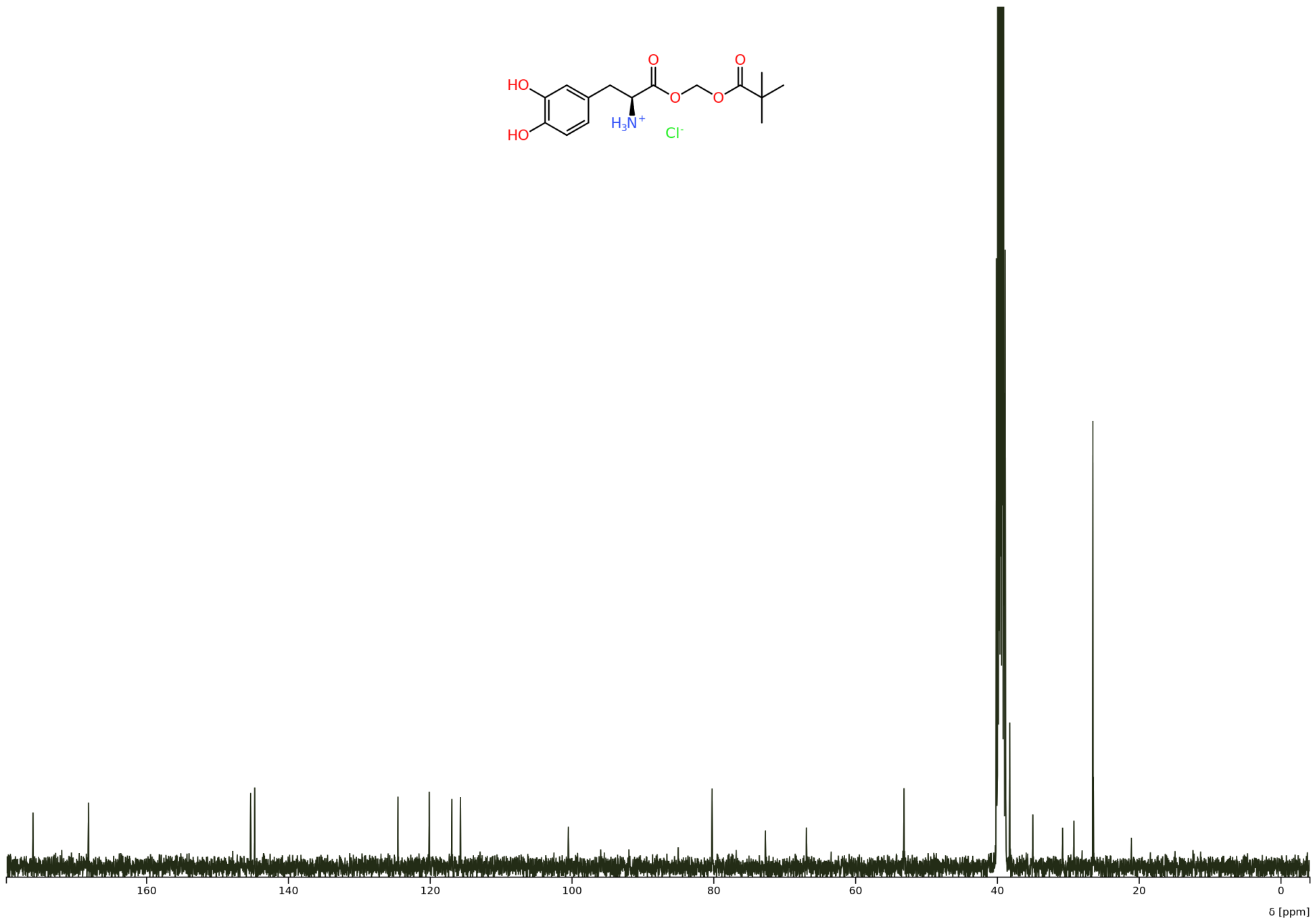

Compound 7e

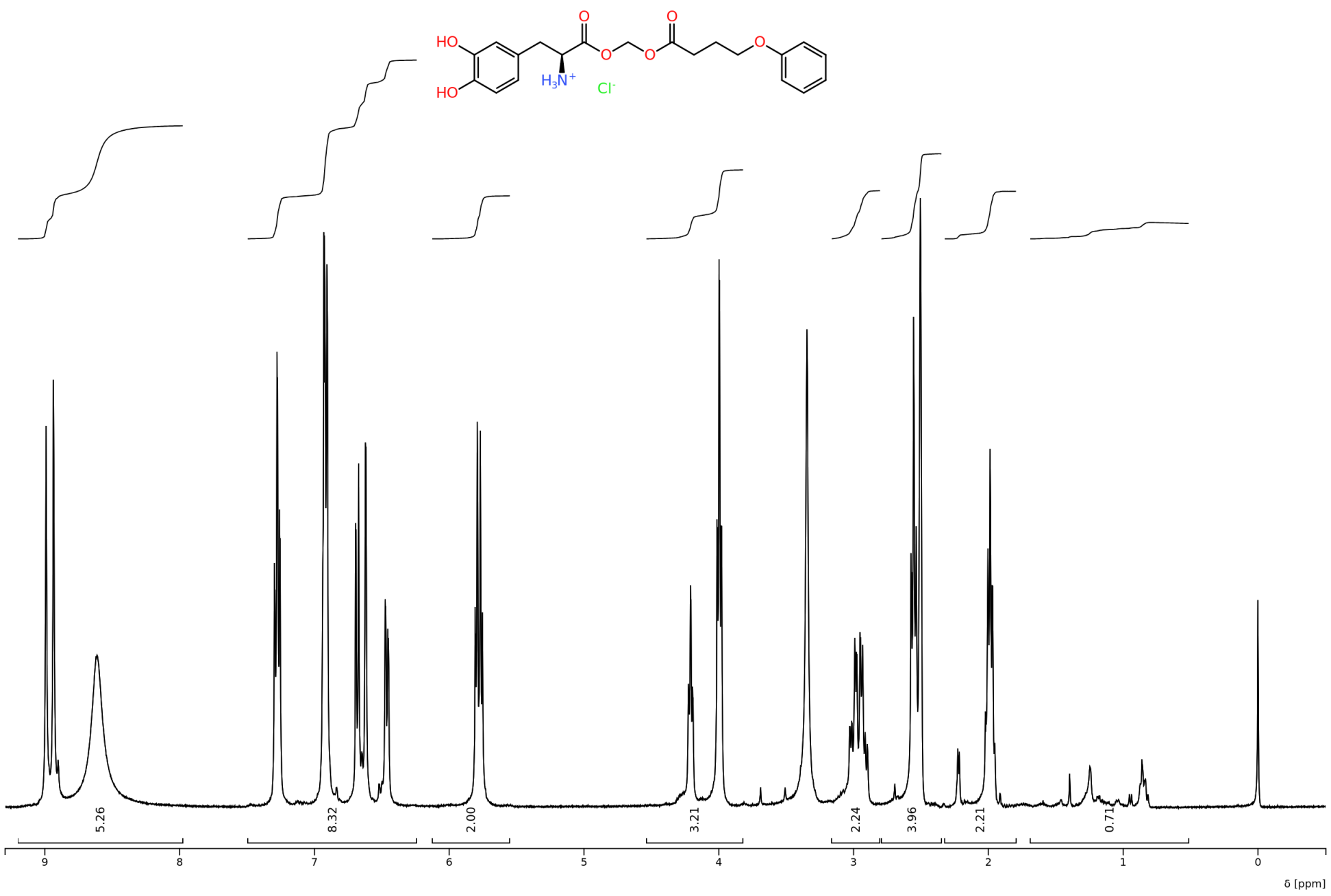
HO
HO
O
O
O
O
O
H_3N^+
Cl^-
5.26
8.32
2.00
3.21
2.24
3.96
2.21
0.71
9
8
7
6
5
4
3
2
1
0
δ [ppm]

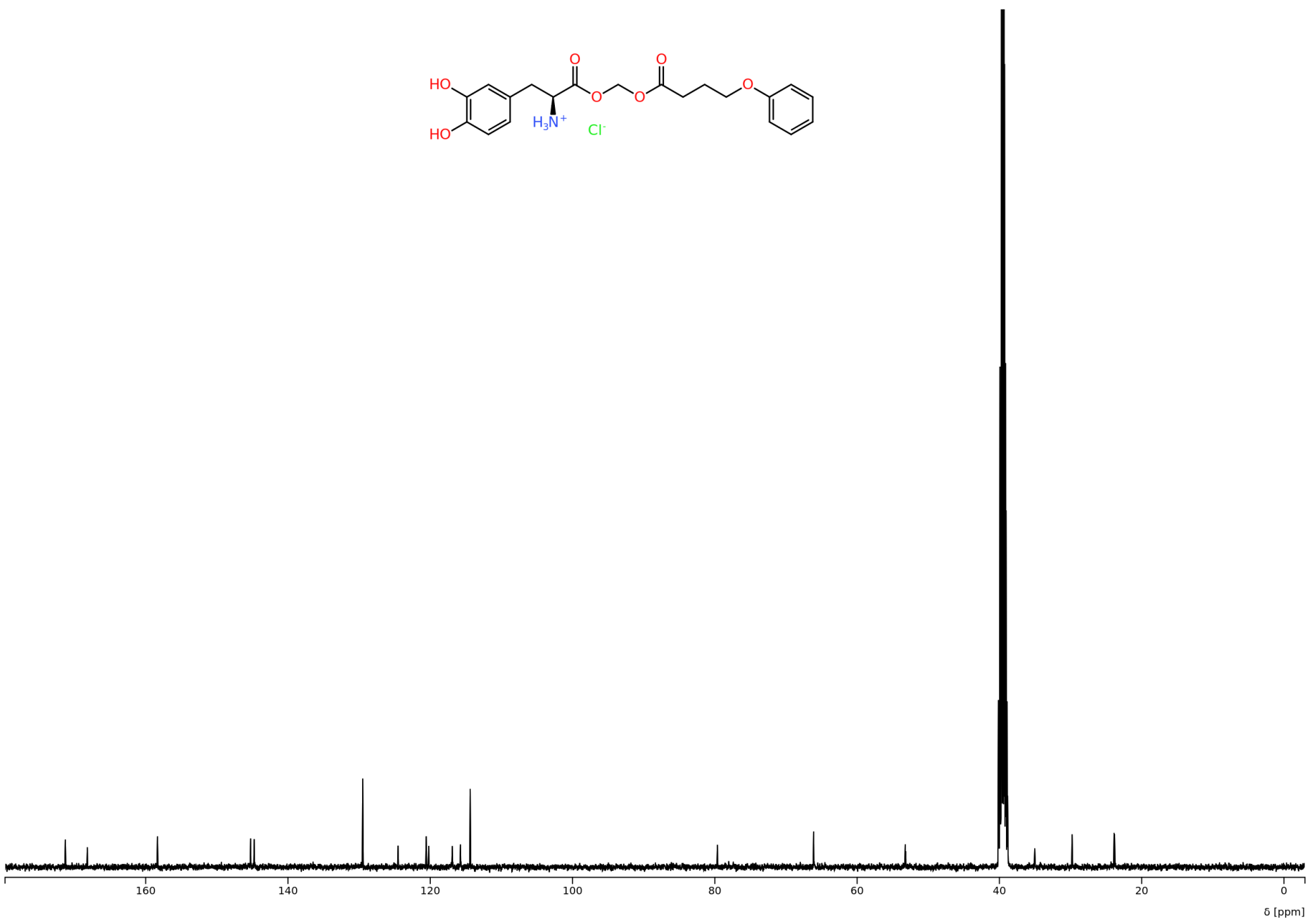
HO
HO
O
O
O
O
O
H_3N^+
Cl^-
160
140
120
100
80
60
40
20
0
δ [ppm]

Compound 7f

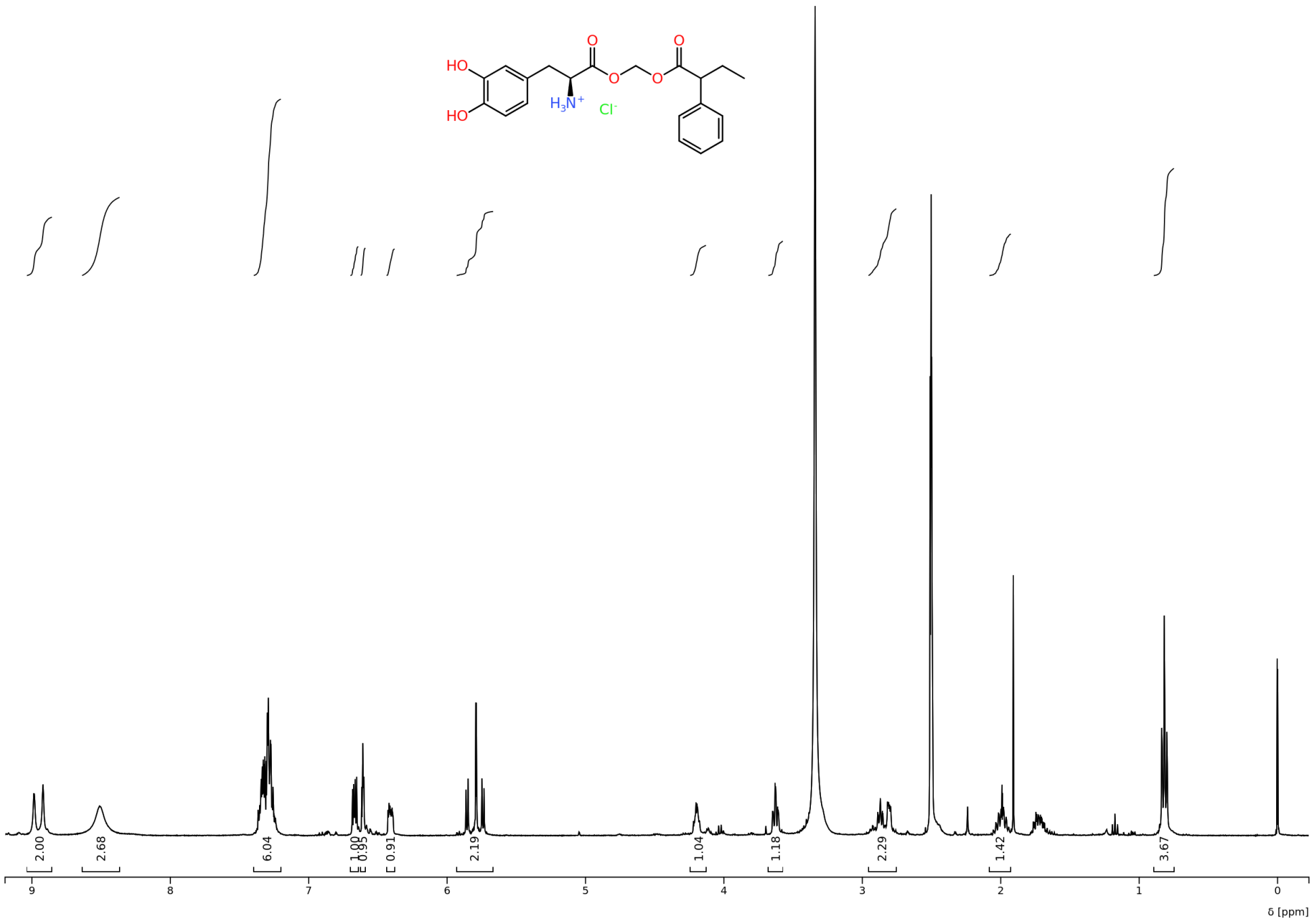
HO
HO
O
O
O
O
H_3N^+
Cl^-
2.00
2.68
6.04
1.00
0.95
0.91
2.19
1.04
1.18
2.29
1.42
3.67
9
8
7
6
5
4
3
2
1
0
δ [ppm]

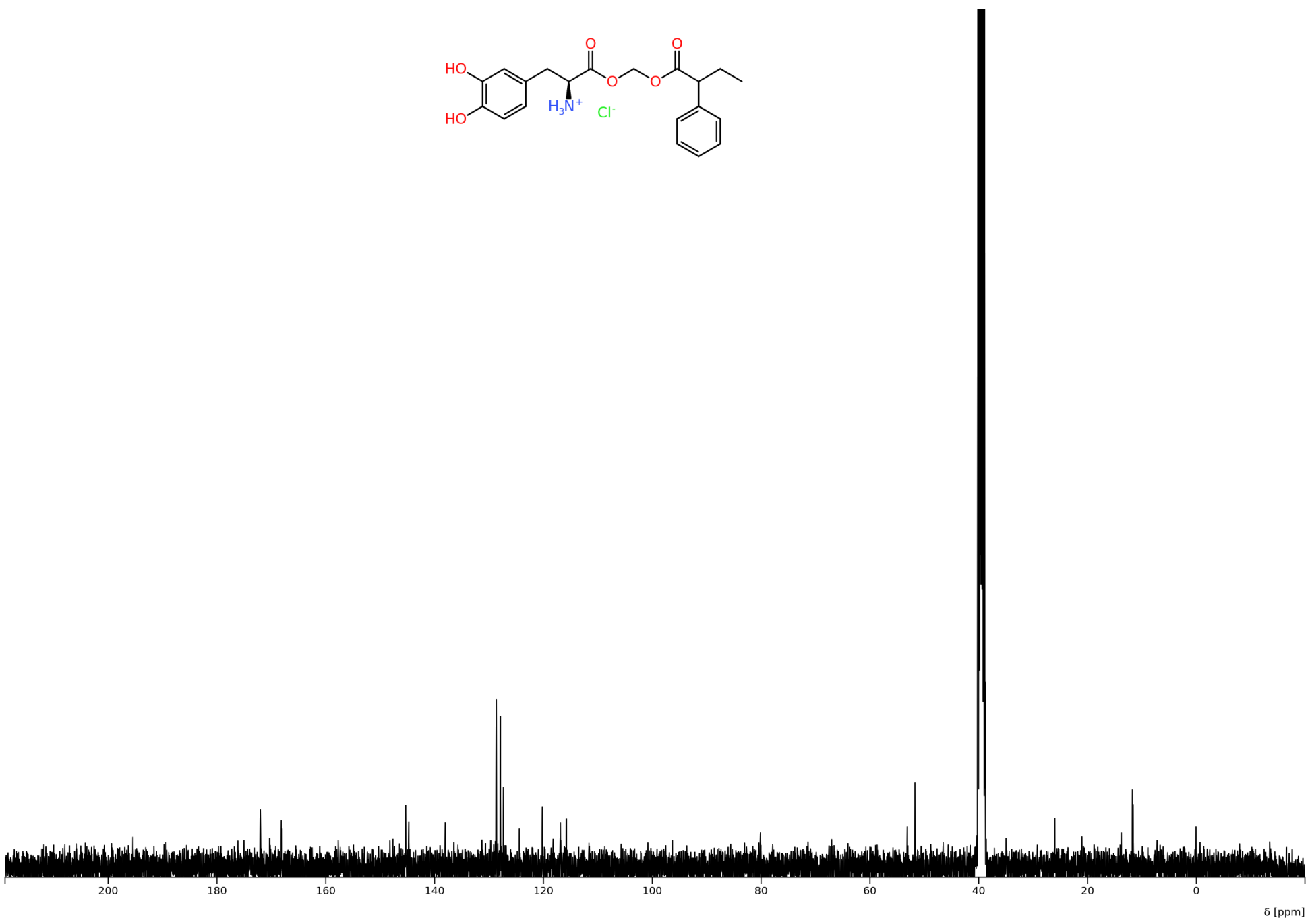
HO
HO
O
O
O
O
H_3N^+
Cl^-
200
180
160
140
120
100
80
60
40
20
0
δ [ppm]

Compound 7g

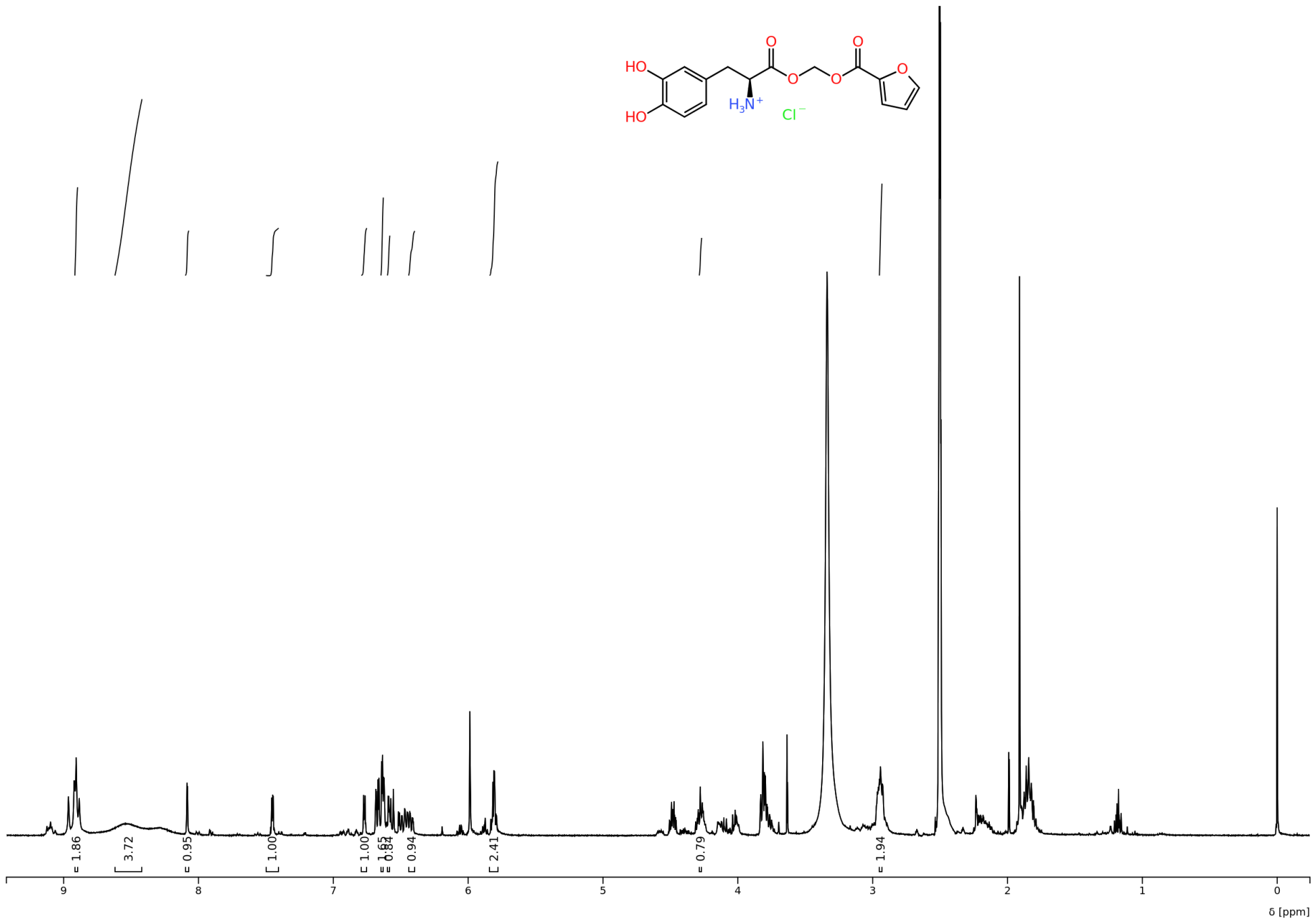

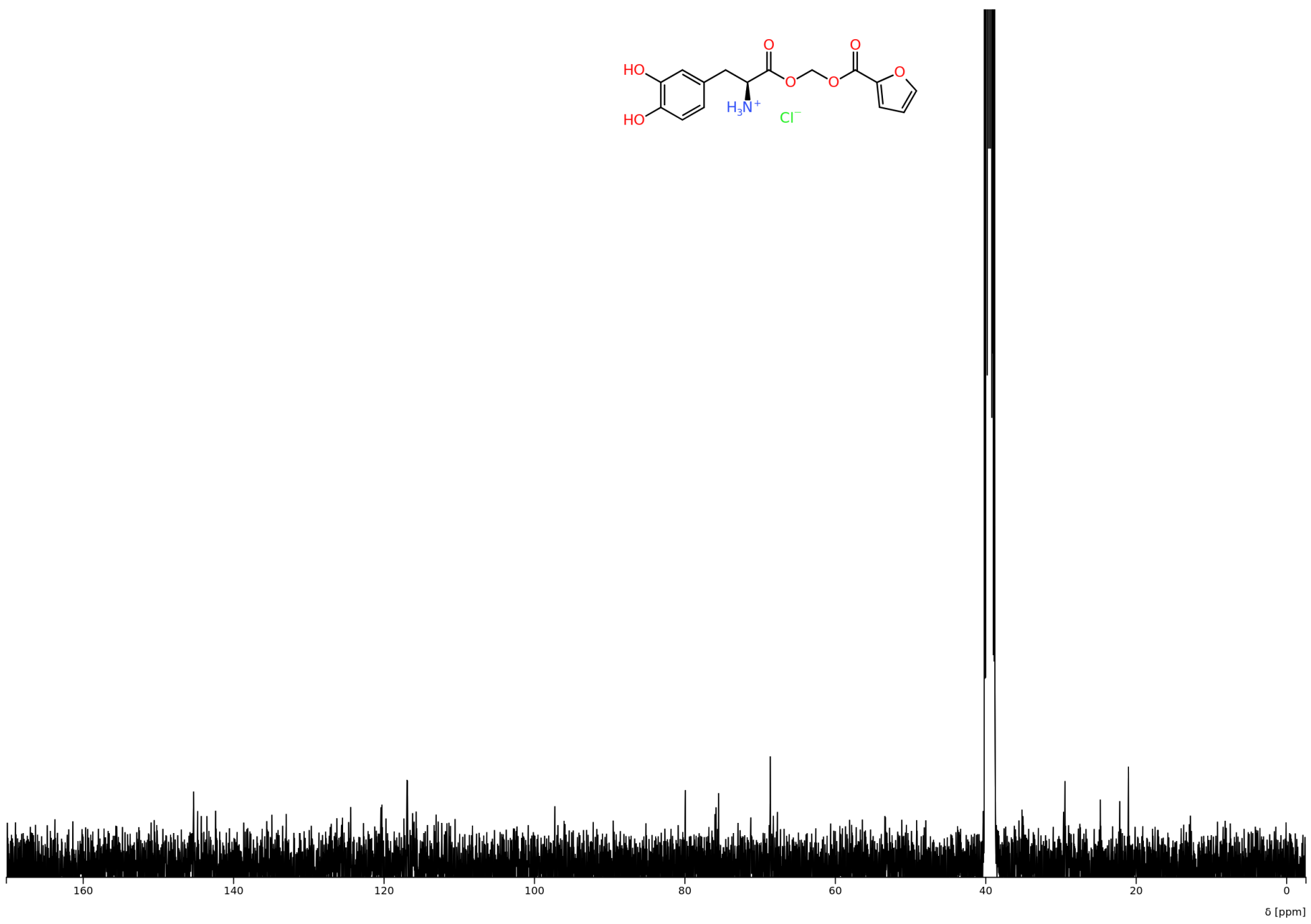

Compound 7h

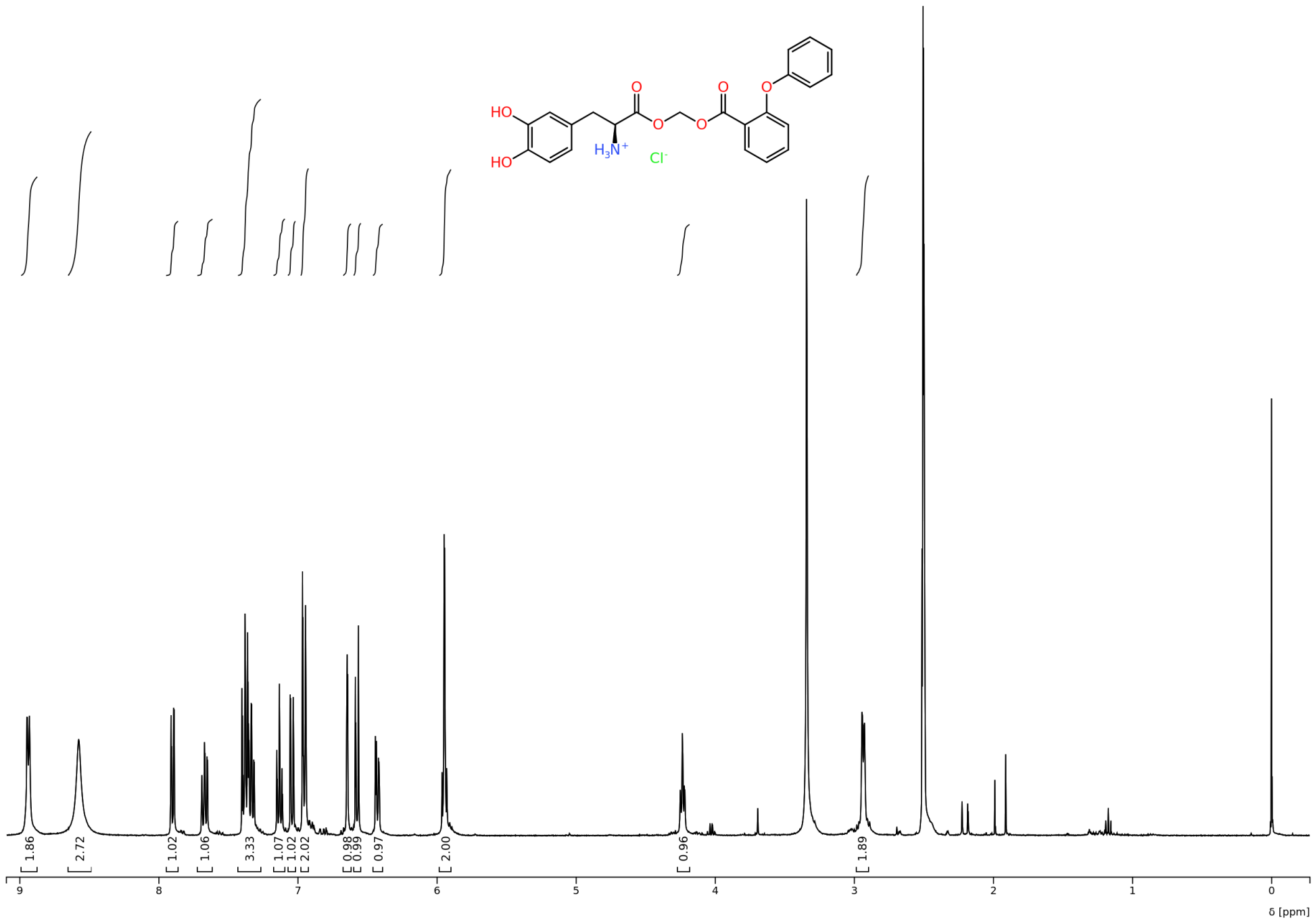

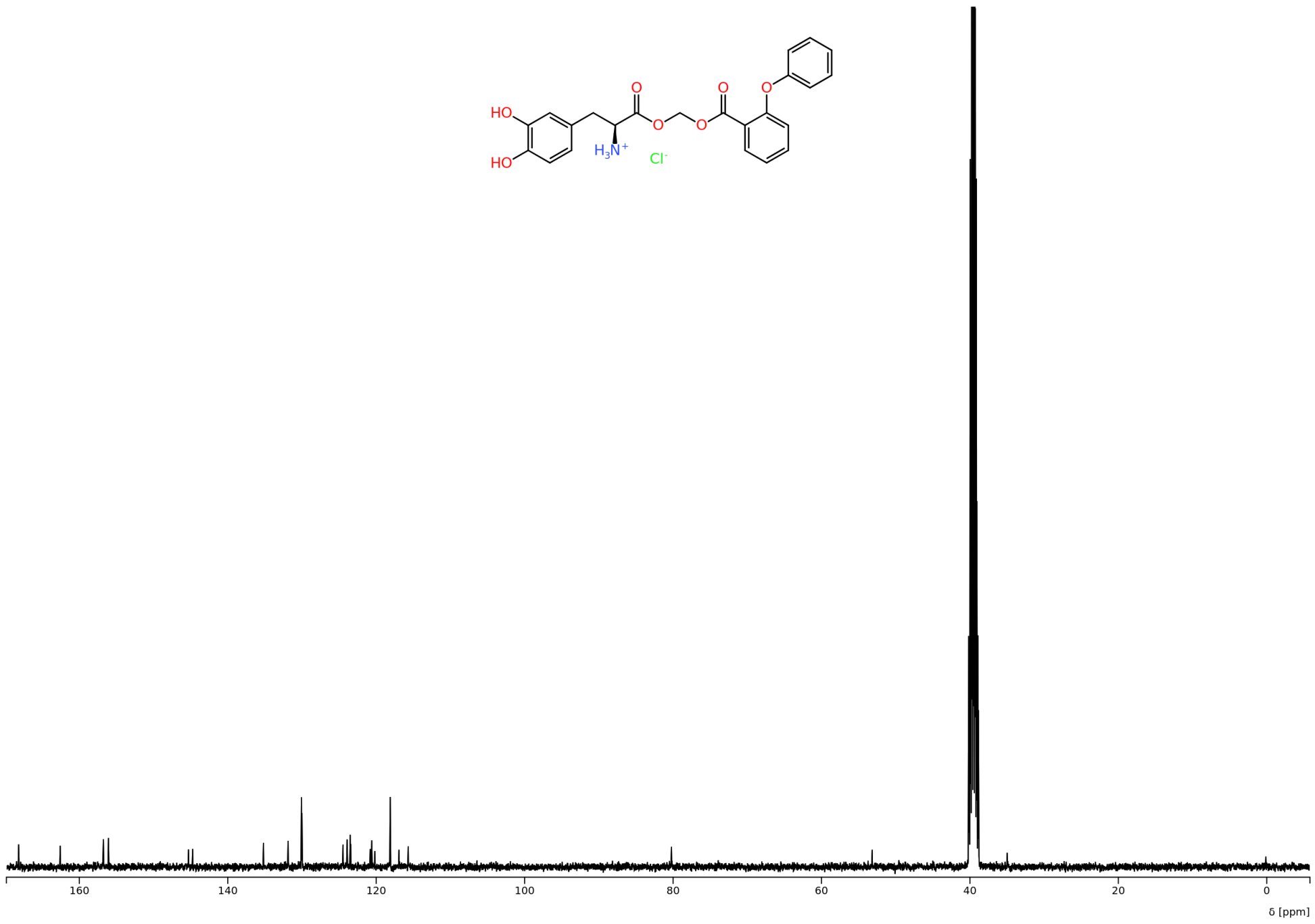

Compound 7i

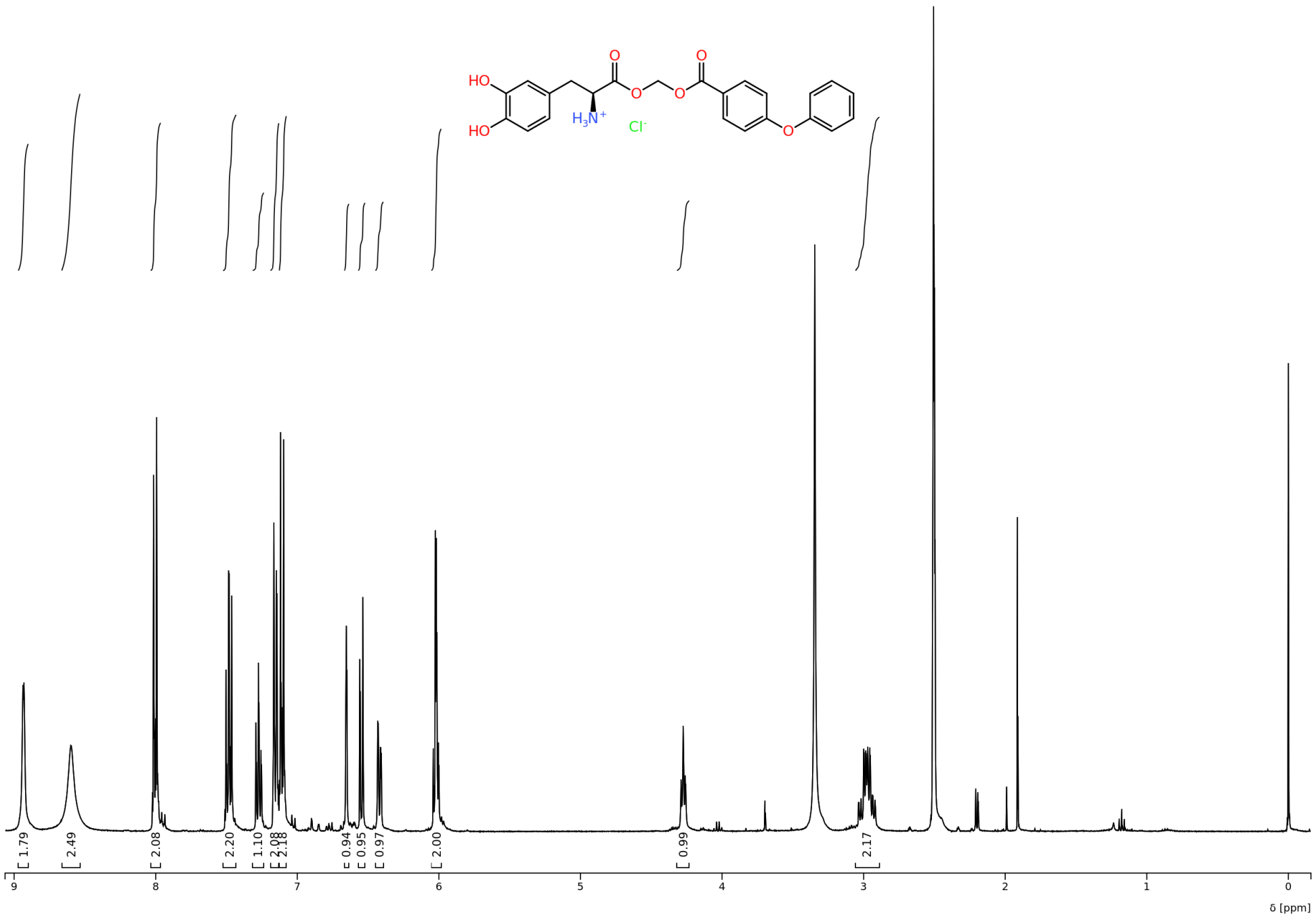
HO
HO
H3N+
Cl-
1.79
2.49
2.08
2.20
1.10
2.08
2.18
0.94
0.95
0.97
2.00
0.99
2.17
9
8
7
6
5
4
3
2
1
0
δ [ppm]

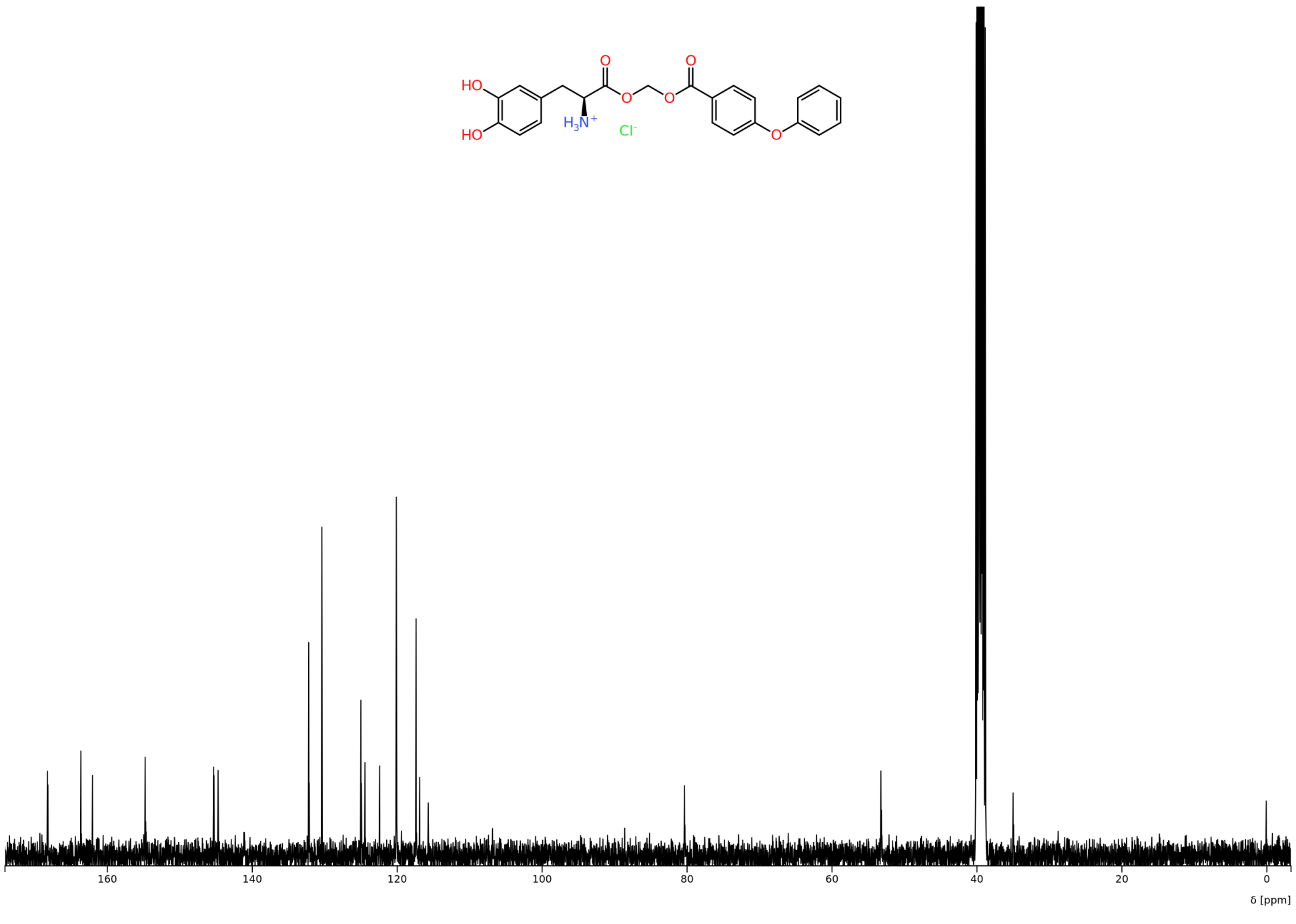
HO
HO
H3N+
Cl-
160
140
120
100
80
60
40
20
0
δ [ppm]

Compound 7j

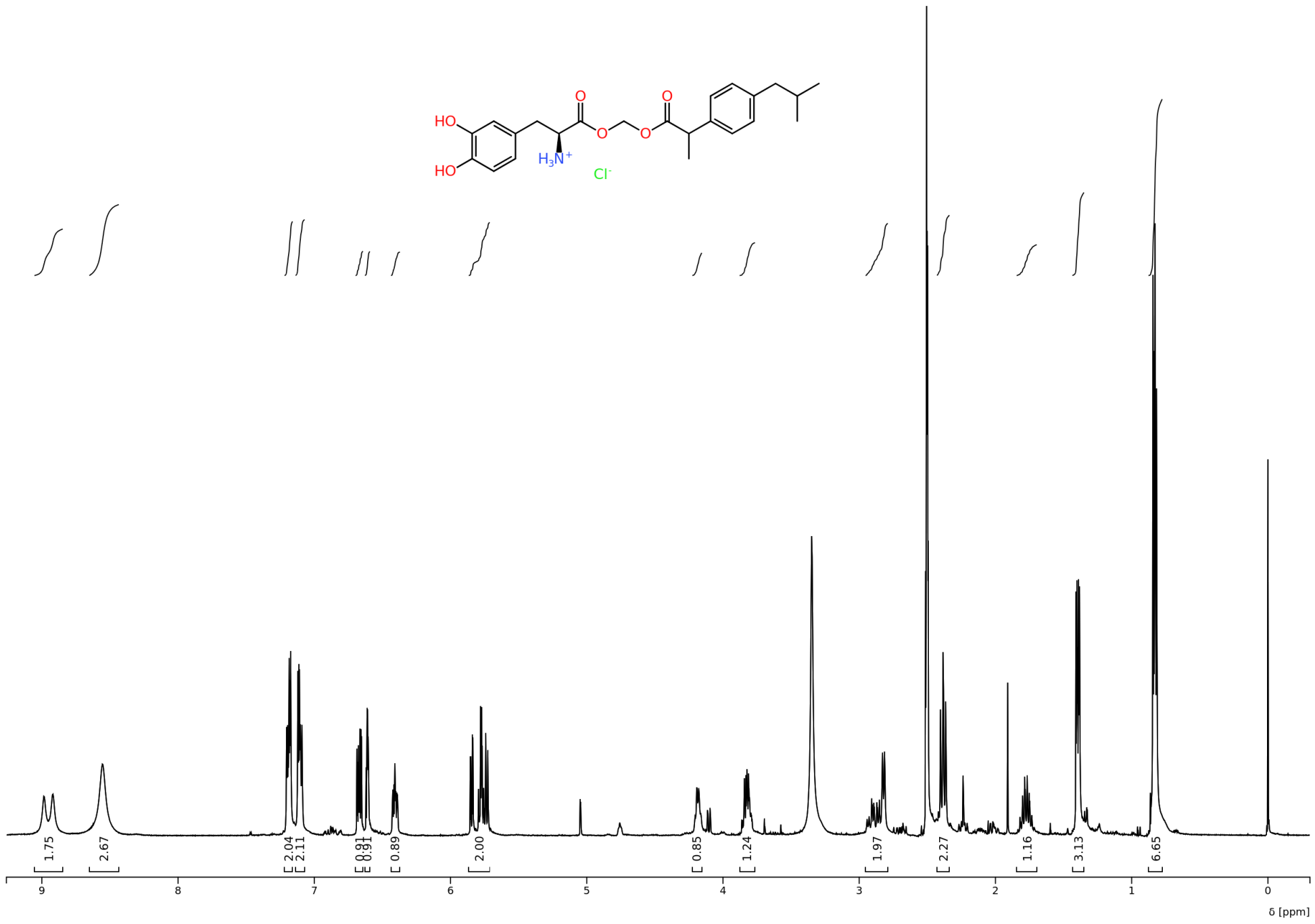
HO
HO
O
O
O
O
H3N+
Cl-
1.75
2.67
2.04
2.11
0.91
0.91
0.89
2.00
0.85
1.24
1.97
2.27
1.16
3.13
6.65
9 8 7 6 5 4 3 2 1 0
δ [ppm]

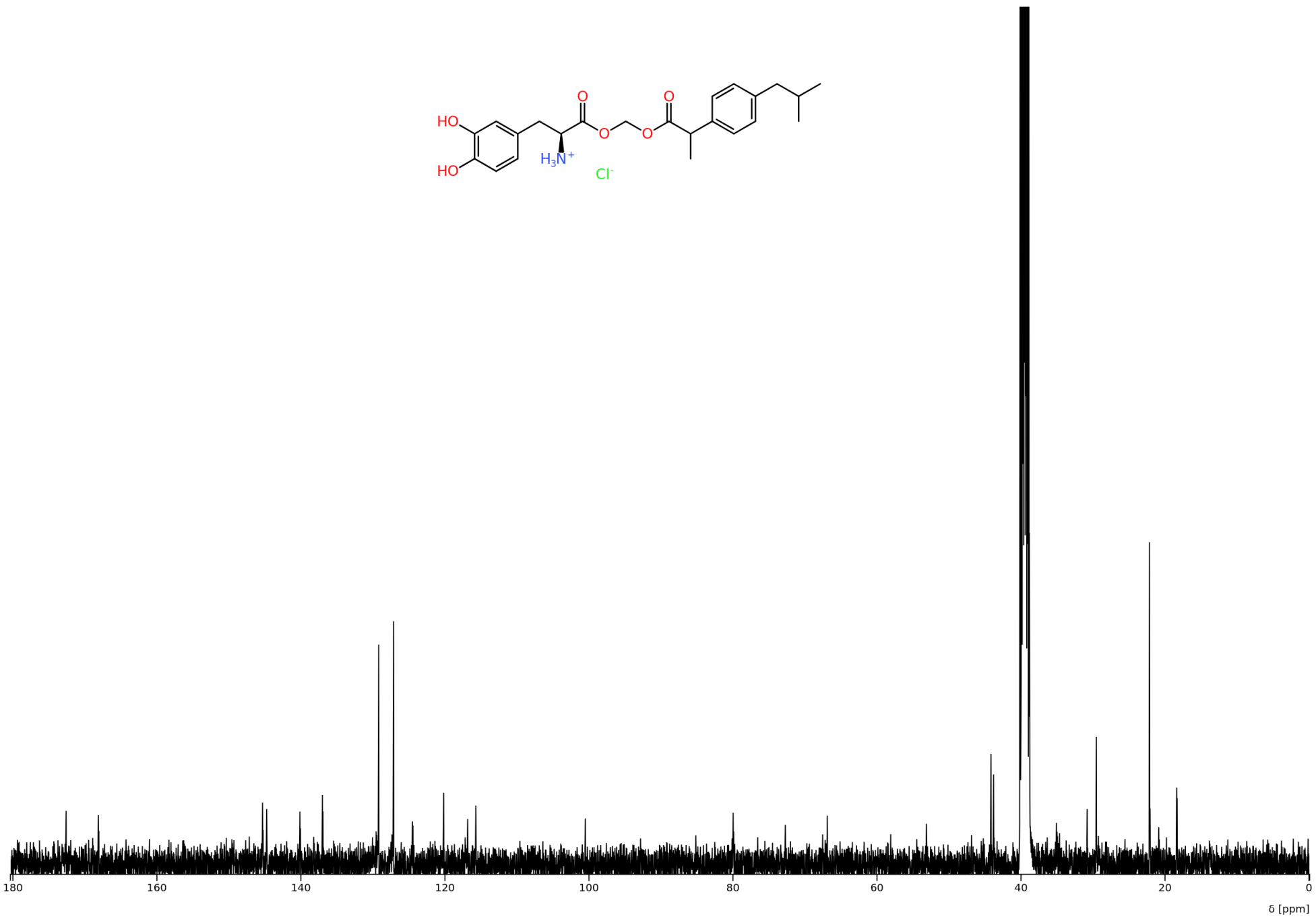
HO
HO
O
O
O
O
H3N+
Cl-
180 160 140 120 100 80 60 40 20 0
δ [ppm]

Compound 7k

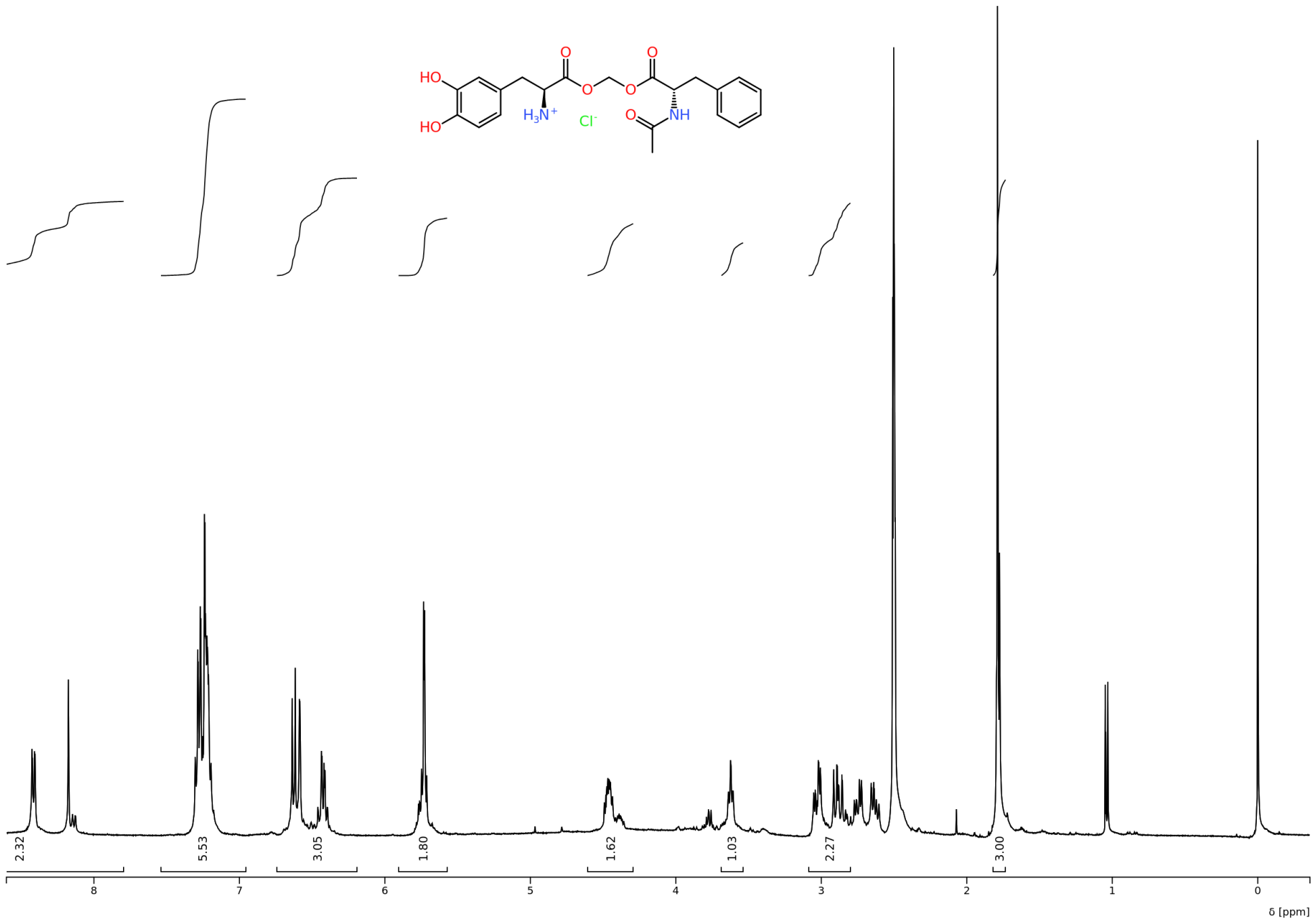

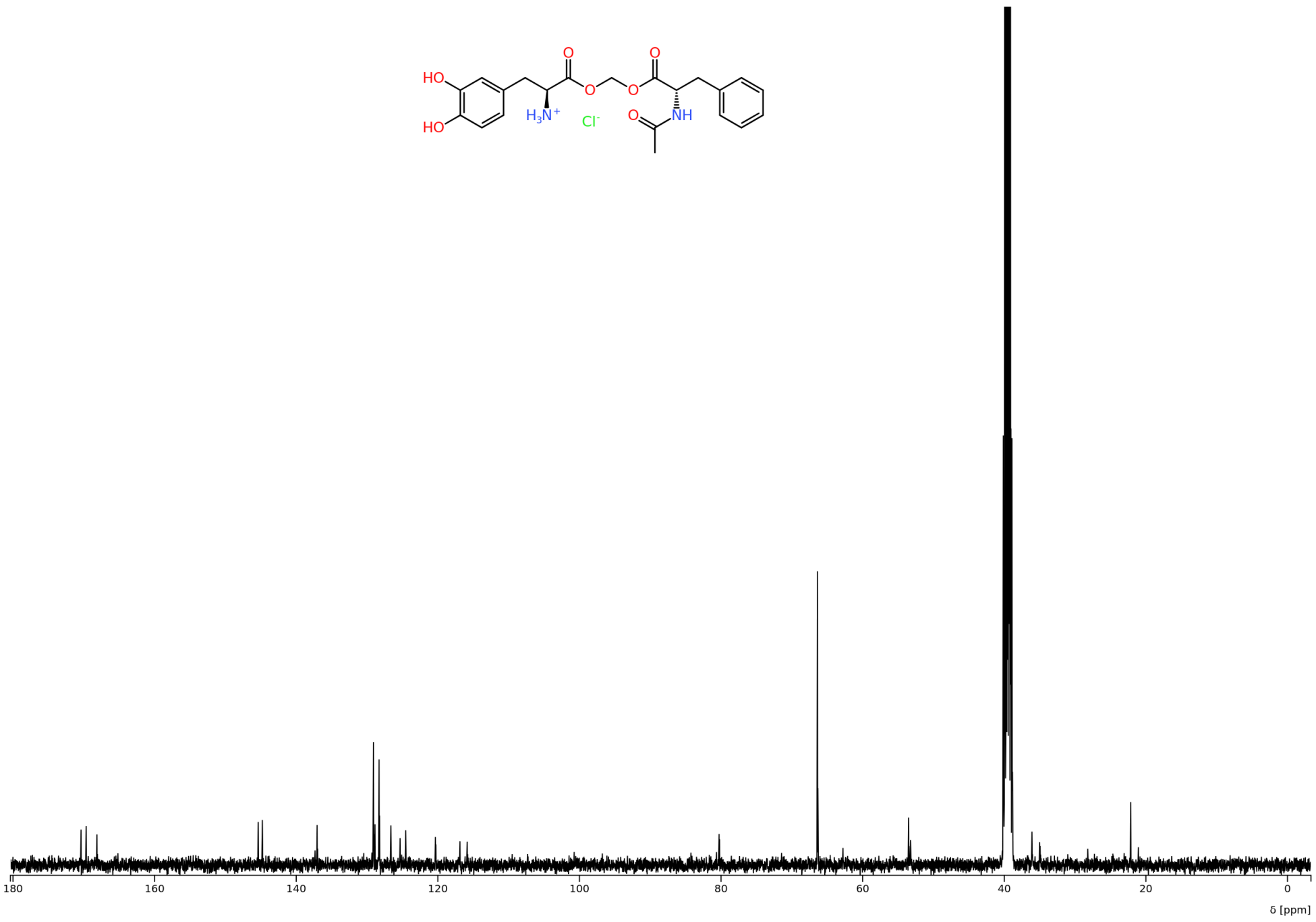

Compound 7l

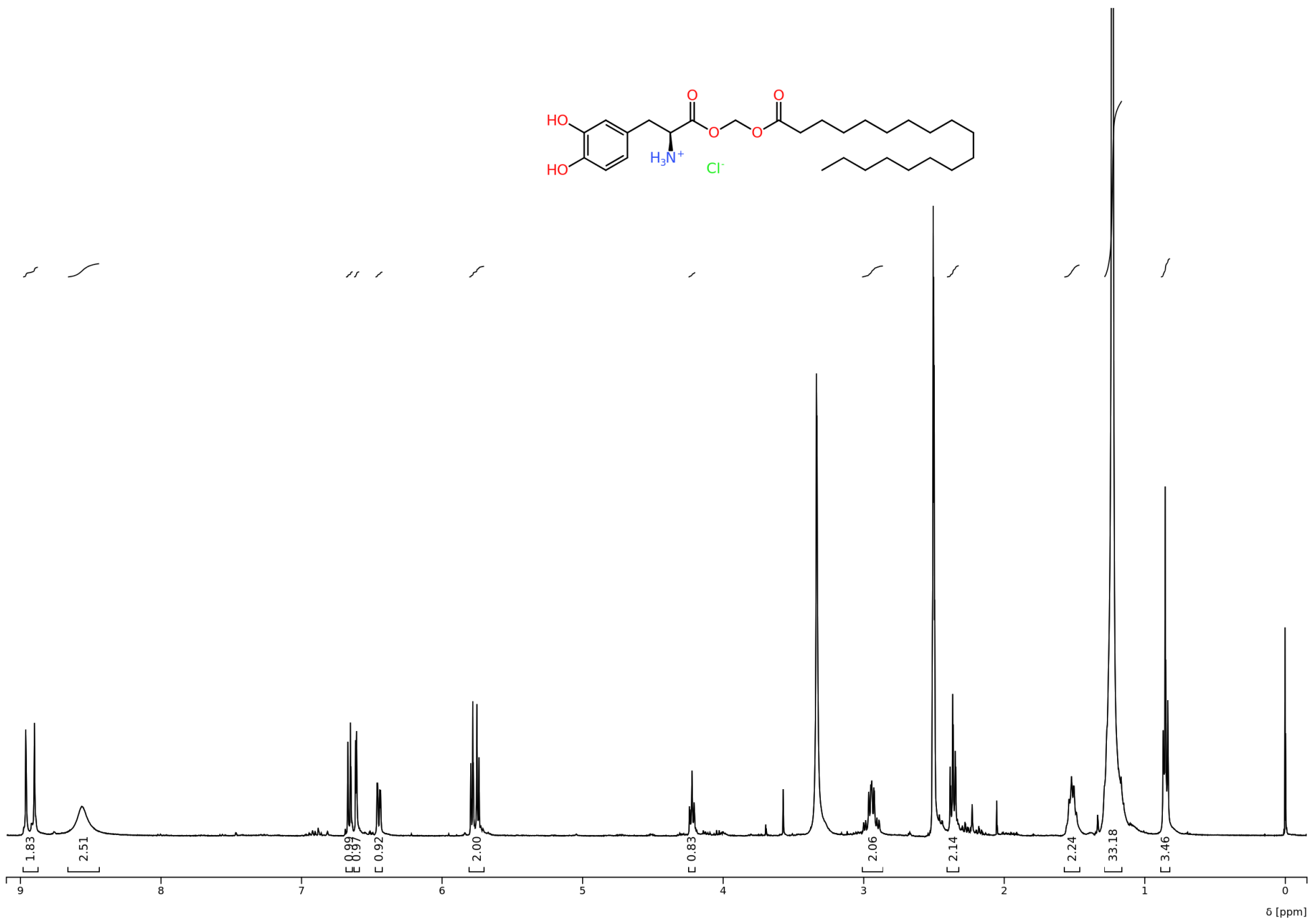

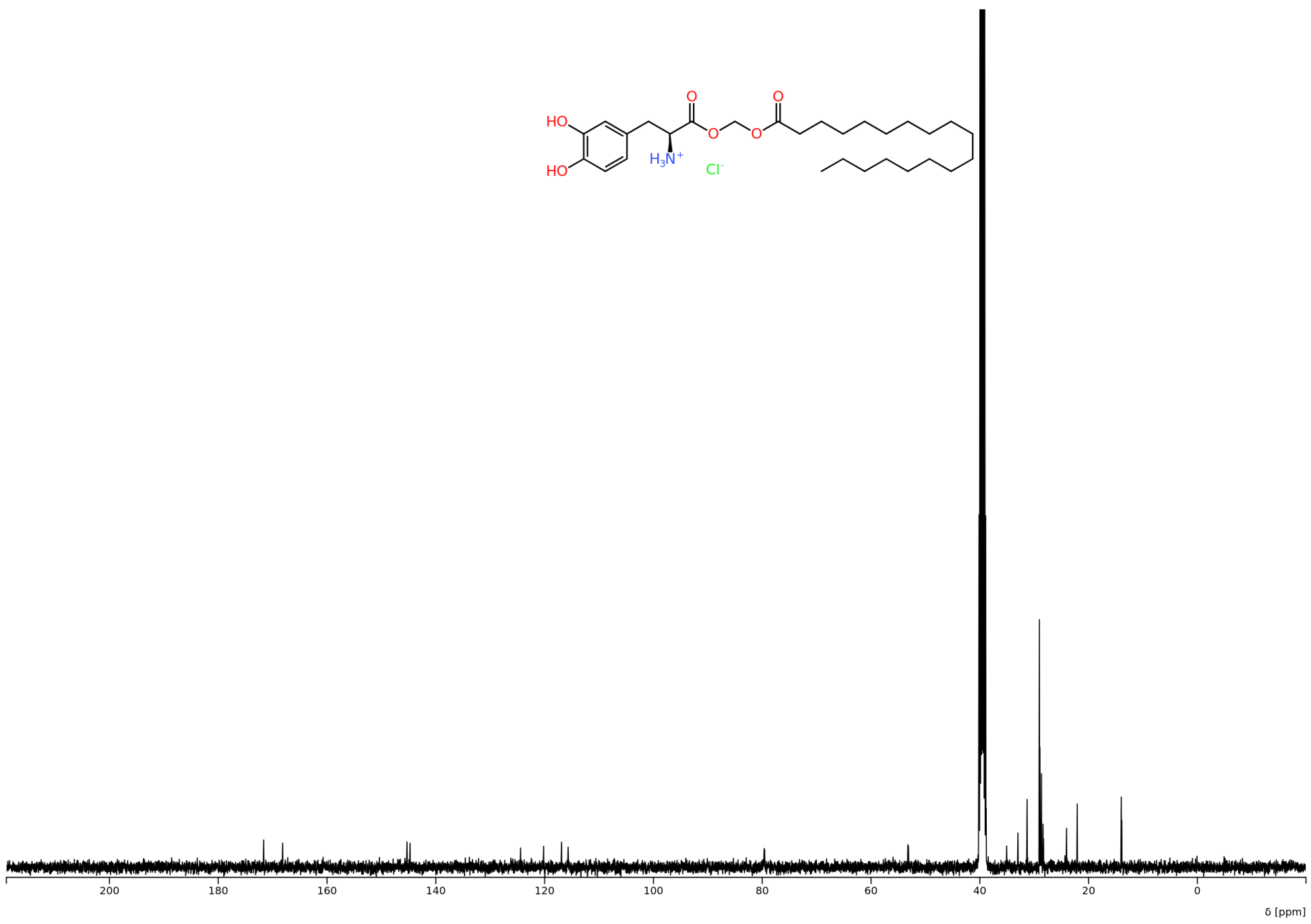

Compound 7m

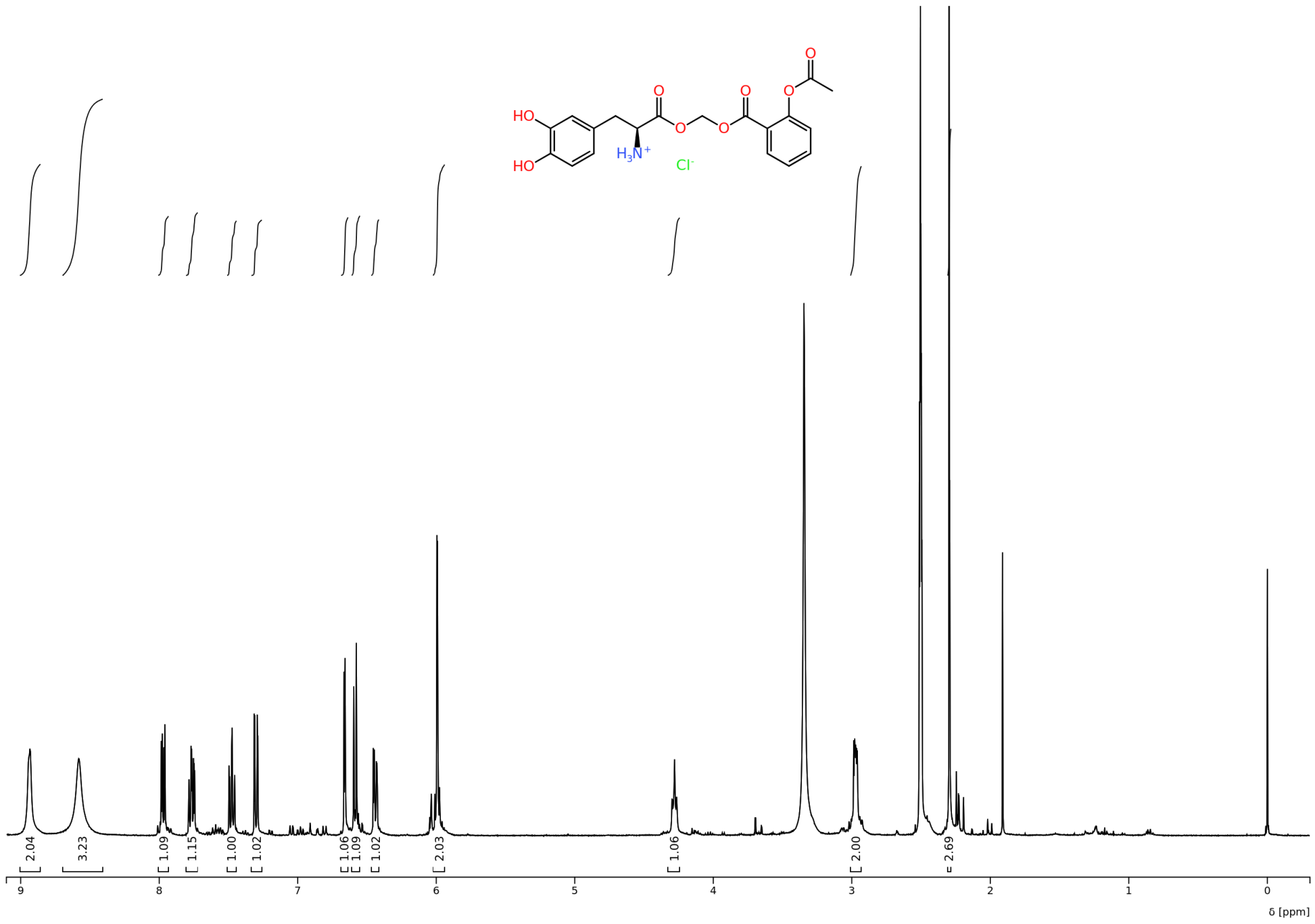

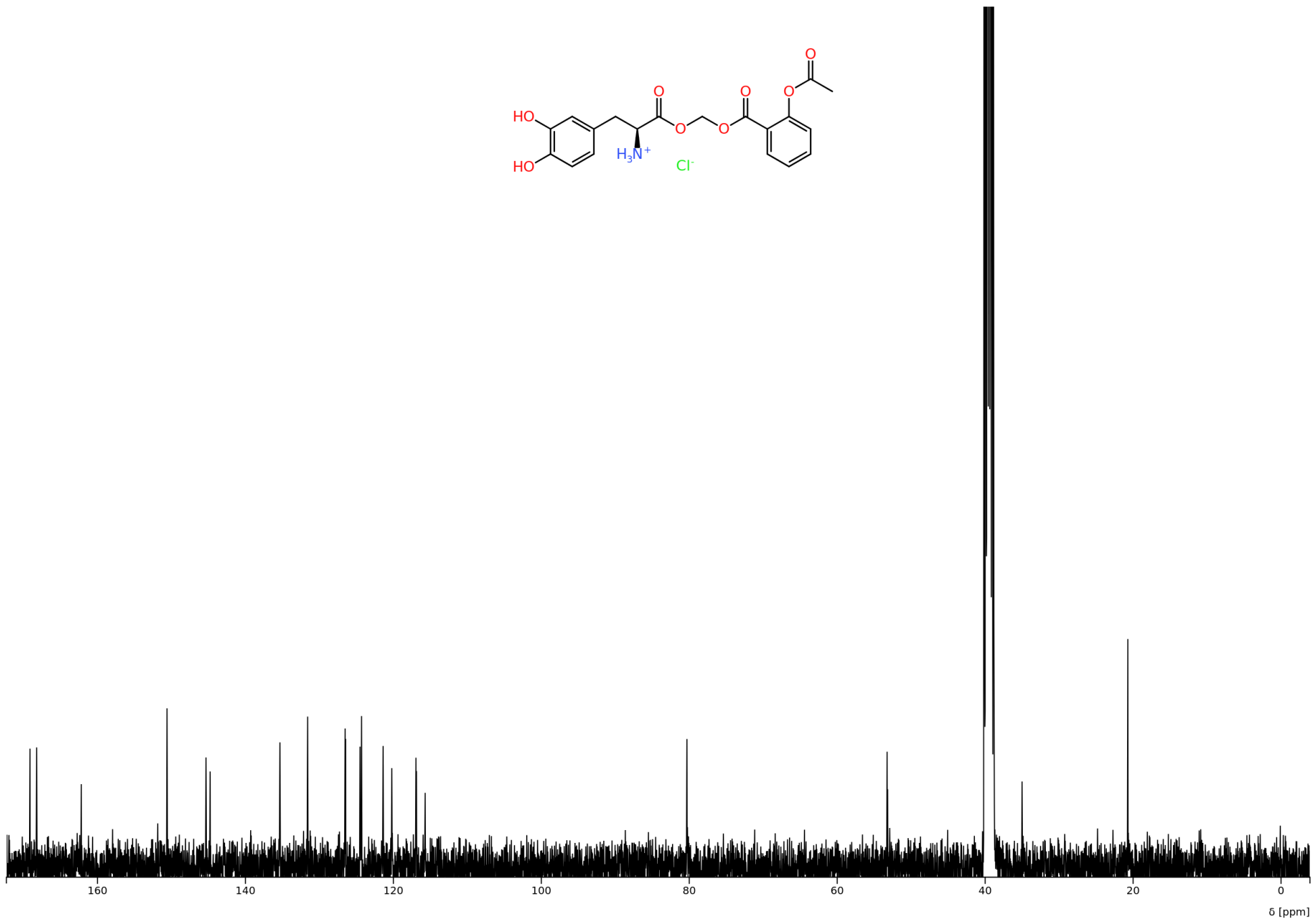

Compound 7n

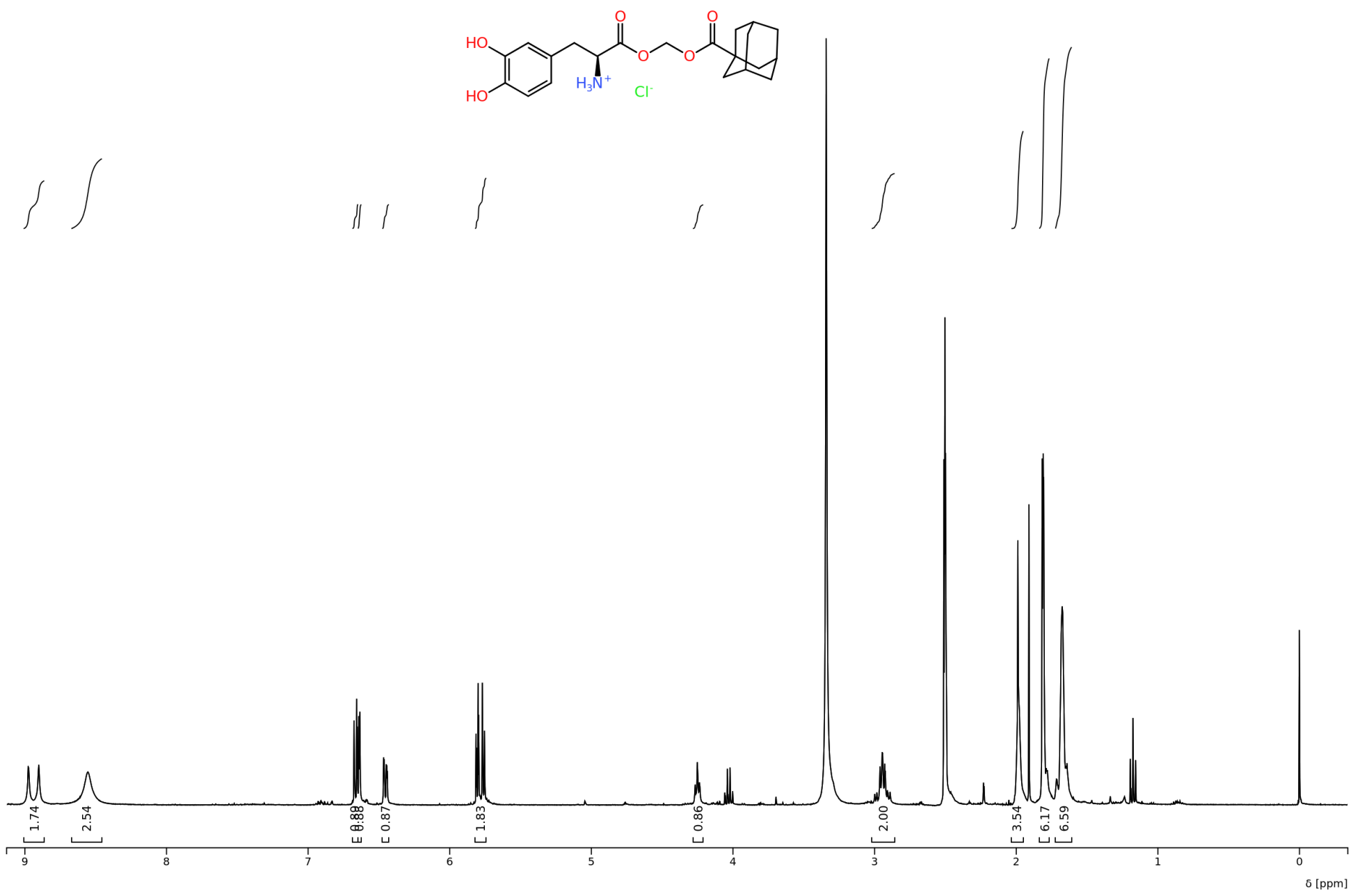

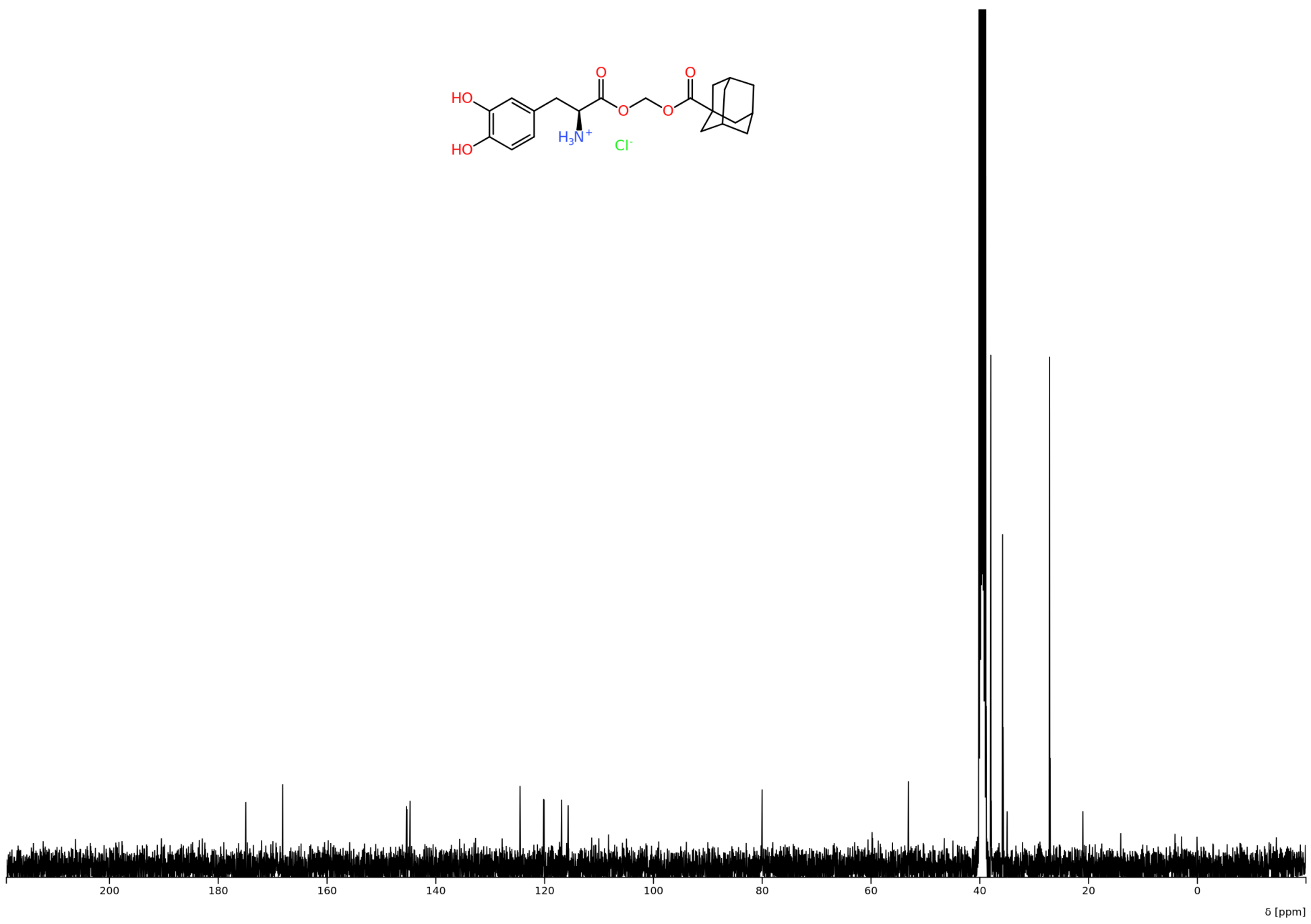

Compound 7o

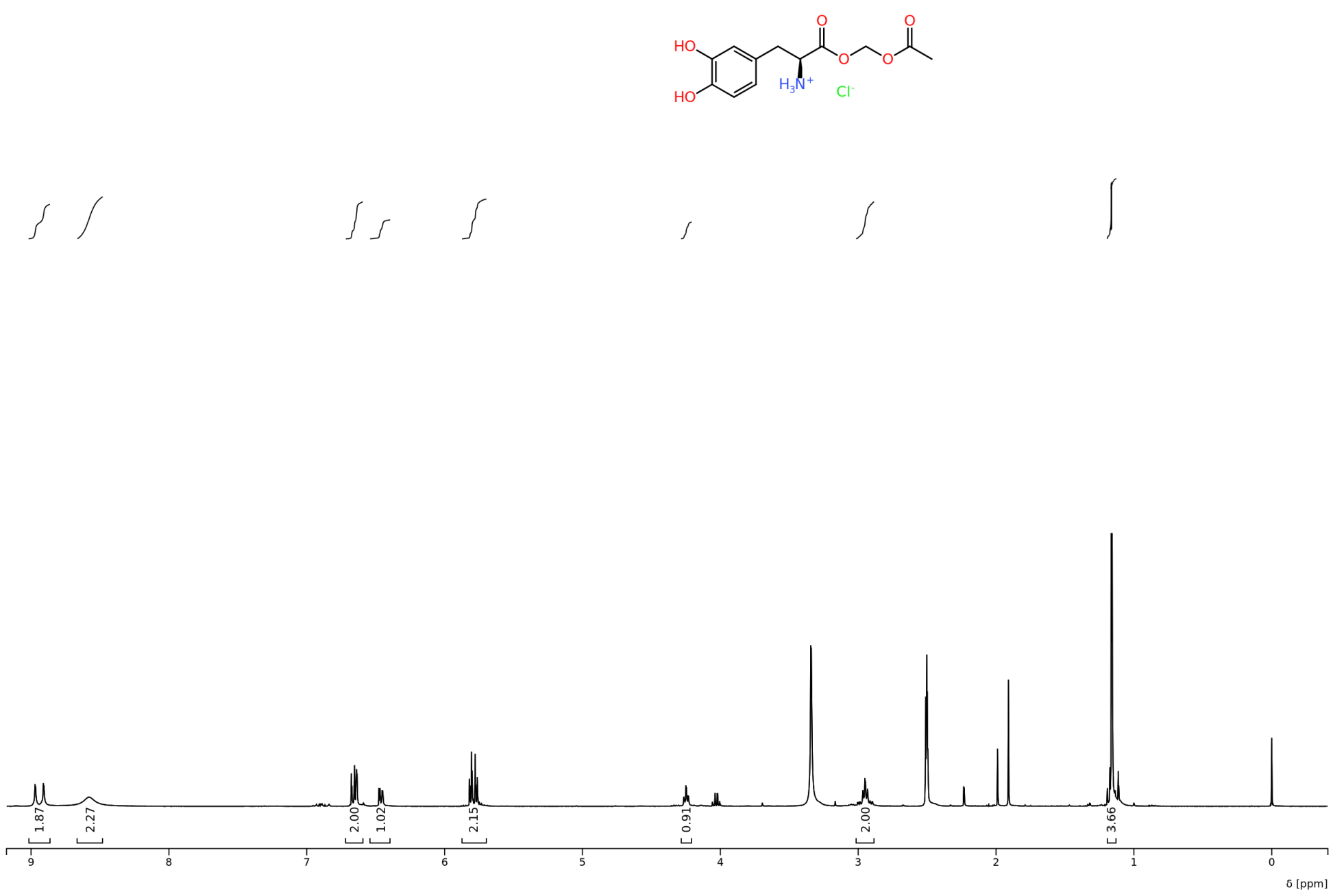
HO
HO
O
O
O
O
H3N+
Cl-
1.87
2.27
2.00
1.02
2.15
0.91
2.00
3.66
9
8
7
6
5
4
3
2
1
0
δ [ppm]

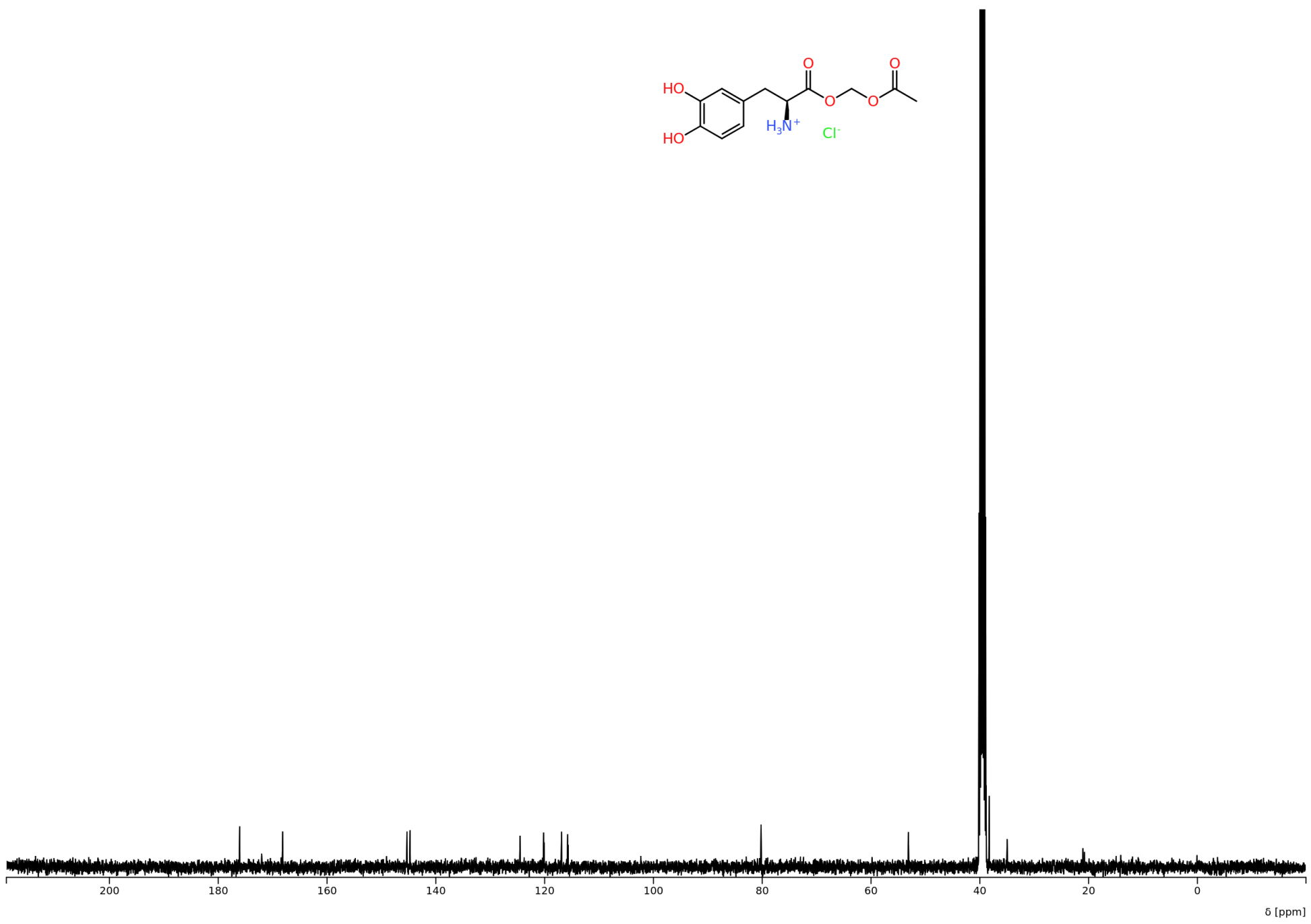
HO
HO
O
O
O
O
H3N+
Cl-
200
180
160
140
120
100
80
60
40
20
0
δ [ppm]
