# Supplementary material for: Clarifying Problems in the Synthetic Pathway Towards Methylene‐Type Levodopa Acylals as Potential Prodrugs
Source: Arch Pharm (Weinheim). 2026 Oct 26;359(10):e70376. doi: 10.1002/ardp.70376 (PMC13699375; doi:10.1002/ardp.70376)
Supplement: Supplementary file 2 [file ARDP-359-e70376-s002.pdf]

ARCH PHARM
Archiv der Pharmazie

Clarifying Problems in the Synthetic Pathway towards Methylene-Type Levodopa Acylals as Potential Prodrugs

Tobias Keydel[1], **Yann Theele**[2], and **Andreas Link**[3]*

[1] University Medical Center Rostock, Rostock, Germany
[2] Pharma Center Bonn, Pharmaceutical Insitute, Pharmaceutical & Medicinal Chemistry, University of Bonn, Bonn, Germany
[3] University of Greifswald, Greifswald, Germany

*Correspondence:
Prof. Dr. A. Link
Institute of Pharmacy
University of Greifswald
Friedrich-Ludwig-Jahn-Str. 17, 17489 Greifswald, Germany
Tel: (+49) (0)3834 4204892
E-mail: link@uni-greifswald.de

Supplemental Material: Novel Compounds

Table 1: Compound structures (InChI Codes)

Compound	InChI Code	Biological Activity
1	1S/C14H19NO6/c1-14(2,3)21-13(20)15-9(12(18)19)6-8-4-5-10(16)11(17)7-8/h4-5,7,9,16-17H,6H2,1-3H3,(H,15,20)(H,18,19)/t9-/m0/s1	not tested
2	1S/C35H37NO6/c1-35(2,3)42-34(38)36-30(33(37)41-25-28-17-11-6-12-18-28)21-29-19-20-31(39-23-26-13-7-4-8-14-26)32(22-29)40-24-27-15-9-5-10-16-27/h4-20,22,30H,21,23-25H2,1-3H3,(H,36,38)/t30-/m0/s1	not tested
3	1S/C28H31NO6/c1-28(2,3)35-27(32)29-23(26(30)31)16-22-14-15-24(33-18-20-10-6-4-7-11-20)25(17-22)34-19-21-12-8-5-9-13-21/h4-15,17,23H,16,18-19H2,1-3H3,(H,29,32)(H,30,31)/t23-/m0/s1	not tested
4a	1S/C29H32ClNO6/c1-29(2,3)37-28(33)31-24(27(32)36-20-30)16-23-14-15-25(34-18-21-10-6-4-7-11-21)26(17-23)35-19-22-12-8-5-9-13-22/h4-15,17,24H,16,18-20H2,1-3H3,(H,31,33)/t24-/m0/s1	not tested
4b	1S/C8H7ClO2/c9-6-11-8(10)7-4-2-1-3-5-7/h1-5H,6H2	not tested
4c	1S/C12H15ClO2/c13-10-15-12(14)9-5-4-8-11-6-2-1-3-7-11/h1-3,6-7H,4-5,8-10H2	not tested
4d	1S/C10H9ClO2/c11-8-13-10(12)7-6-9-4-2-1-3-5-9/h1-7H,8H2/b7-6+	not tested
4e	1S/C6H11ClO2/c1-6(2,3)5(8)9-4-7/h4H2,1-3H3	not tested
4f	1S/C11H13ClO3/c12-9-15-11(13)7-4-8-14-10-5-2-1-3-6-10/h1-3,5-6H,4,7-9H2	not tested
4g	1S/C11H13ClO2/c1-2-10(11(13)14-8-12)9-6-4-3-5-7-9/h3-7,10H,2,8H2,1H3	not tested
4h	1S/C6H5ClO3/c7-4-10-6(8)5-2-1-3-9-5/h1-3H,4H2	not tested
4i	1S/C14H11ClO3/c15-10-17-14(16)12-8-4-5-9-13(12)18-11-6-2-1-3-7-11/h1-9H,10H2	not tested
4j	1S/C14H11ClO3/c15-10-17-14(16)11-6-8-13(9-7-11)18-12-4-2-1-3-5-12/h1-9H,10H2	not tested
4k	1S/C14H19ClO2/c1-10(2)8-12-4-6-13(7-5-12)11(3)14(16)17-9-15/h4-7,10-11H,8-9H2,1-3H3	not tested
4l	1S/C12H14ClNO3/c1-9(15)14-11(12(16)17-8-13)7-10-5-3-2-4-6-10/h2-6,11H,7-8H2,1H3,(H,14,15)/t11-/m0/s1	not tested
4m	1S/C19H35ClO2/c1-2-3-4-5-6-7-8-9-10-11-12-13-14-15-16-17-19(21)22-18-20/h9-10H,2-8,11-18H2,1H3/b10-9-	not tested
4n	1S/C10H9ClO4/c1-7(12)15-9-5-3-2-4-8(9)10(13)14-6-11/h2-5H,6H2,1H3	not tested
4o	1S/C12H17ClO2/c13-7-15-11(14)12-4-8-1-9(5-12)3-10(2-8)6-12/h8-10H,1-7H2/t8-,9+,10-,12?	not tested
5a	1S/C36H37NO8/c1-36(2,3)45-35(40)37-30(34(39)44-25-43-33(38)29-17-11-6-12-18-29)21-28-19-20-31(41-23-26-13-7-4-8-14-26)32(22-28)42-24-27-15-9-5-10-16-27/h4-20,22,30H,21,23-25H2,1-3H3,(H,37,40)/t30-/m0/s1	not tested
5b	1S/C40H45NO8/c1-40(2,3)49-39(44)41-34(38(43)48-29-47-37(42)22-14-13-17-30-15-7-4-8-16-30)25-33-23-24-35(45-27-31-18-9-5-10-19-31)36(26-33)46-28-32-20-11-6-12-21-32/h4-12,15-16,18-21,23-24,26,34H,13-14,17,22,25,27-29H2,1-3H3,(H,41,44)/t34-/m0/s1	not tested
5c	1S/C38H39NO8/c1-38(2,3)47-37(42)39-32(36(41)46-27-45-35(40)22-20-28-13-7-4-8-14-28)23-31-19-21-33(43-25-29-15-9-5-10-16-29)34(24-31)44-26-30-17-11-6-12-18-30/h4-22,24,32H,23,25-27H2,1-3H3,(H,39,42)/b22-20+/t32-/m0/s1	not tested
5d	1S/C34H41NO8/c1-33(2,3)31(37)42-23-41-30(36)27(35-32(38)43-34(4,5)6)19-26-17-18-28(39-21-24-13-9-7-10-14-24)29(20-26)40-22-25-15-11-8-12-16-25/h7-18,20,27H,19,21-23H2,1-6H3,(H,35,38)/t27-/m0/s1	not tested
5e	1S/C39H43NO9/c1-39(2,3)49-38(43)40-33(37(42)48-28-47-36(41)20-13-23-44-32-18-11-6-12-19-32)24-31-21-22-34(45-26-29-14-7-4-8-15-29)35(25-31)46-27-30-16-9-5-10-17-30/h4-12,14-19,21-22,25,33H,13,20,23-24,26-28H2,1-3H3,(H,40,43)/t33-/m0/s1	not tested
5f	1S/C39H43NO8/c1-5-32(31-19-13-8-14-20-31)36(41)46-27-47-37(42)33(40-38(43)48-39(2,3)4)23-30-21-22-34(44-25-28-15-9-6-10-16-28)35(24-30)45-26-29-17-11-7-12-18-29/h6-22,24,32-33H,5,23,25-27H2,1-4H3,(H,40,43)/t32?,33-/m0/s1	not tested
5g	1S/C34H35NO9/c1-34(2,3)44-33(38)35-27(31(36)42-23-43-32(37)29-15-10-18-39-29)19-26-16-17-28(40-21-24-11-6-4-7-12-24)30(20-26)41-22-25-13-8-5-9-14-25/h4-18,20,27H,19,21-23H2,1-3H3,(H,35,38)/t27-/m0/s1	not tested

Table 1 – continued from previous page

Compound	InChI Code	Biological Activity
5h	1S/C42H41NO9/c1-42(2,3)52-41(46)43-35(40(45)50-29-49-39(44)34-21-13-14-22-36(34)51-33-19-11-6-12-20-33)25-32-23-24-37(47-27-30-15-7-4-8-16-30)38(26-32)48-28-31-17-9-5-10-18-31/h4-24,26,35H,25,27-29H2,1-3H3,(H,43,46)/t35-/m0/s1	not tested
5i	1S/C42H41NO9/c1-42(2,3)52-41(46)43-36(40(45)50-29-49-39(44)33-20-22-35(23-21-33)51-34-17-11-6-12-18-34)25-32-19-24-37(47-27-30-13-7-4-8-14-30)38(26-32)48-28-31-15-9-5-10-16-31/h4-24,26,36H,25,27-29H2,1-3H3,(H,43,46)/t36-/m0/s1	not tested
5j	1S/C42H49NO8/c1-29(2)23-31-17-20-35(21-18-31)30(3)39(44)49-28-50-40(45)36(43-41(46)51-42(4,5)6)24-34-19-22-37(47-26-32-13-9-7-10-14-32)38(25-34)48-27-33-15-11-8-12-16-33/h7-22,25,29-30,36H,23-24,26-28H2,1-6H3,(H,43,46)/t30?,36-/m0/s1	not tested
5k	1S/C40H44N2O9/c1-28(43)41-33(22-29-14-8-5-9-15-29)37(44)49-27-50-38(45)34(42-39(46)51-40(2,3)4)23-32-20-21-35(47-25-30-16-10-6-11-17-30)36(24-32)48-26-31-18-12-7-13-19-31/h5-21,24,33-34H,22-23,25-27H2,1-4H3,(H,41,43)(H,42,46)/t33-,34-/m0/s1	not tested
5l	1S/C47H65NO8/c1-5-6-7-8-9-10-11-12-13-14-15-16-17-18-25-30-44(49)54-37-55-45(50)41(48-46(51)56-47(2,3)4)33-40-31-32-42(52-35-38-26-21-19-22-27-38)43(34-40)53-36-39-28-23-20-24-29-39/h12-13,19-24,26-29,31-32,34,41H,5-11,14-18,25,30,33,35-37H2,1-4H3,(H,48,51)/b13-12-/t41-/m0/s1	not tested
5m	1S/C38H39NO10/c1-26(40)48-32-18-12-11-17-30(32)35(41)46-25-47-36(42)31(39-37(43)49-38(2,3)4)21-29-19-20-33(44-23-27-13-7-5-8-14-27)34(22-29)45-24-28-15-9-6-10-16-28/h5-20,22,31H,21,23-25H2,1-4H3,(H,39,43)/t31-/m0/s1	not tested
5n	1S/C40H47NO8/c1-39(2,3)49-38(44)41-33(36(42)47-26-48-37(43)40-21-30-16-31(22-40)18-32(17-30)23-40)19-29-14-15-34(45-24-27-10-6-4-7-11-27)35(20-29)46-25-28-12-8-5-9-13-28/h4-15,20,30-33H,16-19,21-26H2,1-3H3,(H,41,44)/t30-,31-,32?,33+,40?/m1/s1	not tested
5o	1S/C31H35NO8/c1-22(33)38-21-39-29(34)26(32-30(35)40-31(2,3)4)17-25-15-16-27(36-19-23-11-7-5-8-12-23)28(18-25)37-20-24-13-9-6-10-14-24/h5-16,18,26H,17,19-21H2,1-4H3,(H,32,35)/t26-/m0/s1	not tested
6a	1S/C17H17NO6/c18-13(8-11-6-7-14(19)15(20)9-11)17(22)24-10-23-16(21)12-4-2-1-3-5-12/h1-7,9,13,19-20H,8,10,18H2/t13-/m0/s1	not tested
6b	1S/C21H25NO6/c22-17(12-16-10-11-18(23)19(24)13-16)21(26)28-14-27-20(25)9-5-4-8-15-6-2-1-3-7-15/h1-3,6-7,10-11,13,17,23-24H,4-5,8-9,12,14,22H2/t17-/m0/s1	not tested
6c	1S/C19H21NO6/c20-15(10-14-6-8-16(21)17(22)11-14)19(24)26-12-25-18(23)9-7-13-4-2-1-3-5-13/h1-6,8,11,15,21-22H,7,9-10,12,20H2/t15-/m0/s1	not tested
6d	1S/C15H21NO6/c1-15(2,3)14(20)22-8-21-13(19)10(16)6-9-4-5-11(17)12(18)7-9/h4-5,7,10,17-18H,6,8,16H2,1-3H3/t10-/m0/s1	not tested
6e	1S/C20H23NO7/c21-16(11-14-8-9-17(22)18(23)12-14)20(25)28-13-27-19(24)7-4-10-26-15-5-2-1-3-6-15/h1-3,5-6,8-9,12,16,22-23H,4,7,10-11,13,21H2/t16-/m0/s1	not tested
6f	1S/C20H23NO6/c1-2-15(14-6-4-3-5-7-14)19(24)26-12-27-20(25)16(21)10-13-8-9-17(22)18(23)11-13/h3-9,11,15-16,22-23H,2,10,12,21H2,1H3/t15?,16-/m0/s1	not tested
6g	1S/C15H15NO7/c16-10(6-9-3-4-11(17)12(18)7-9)14(19)22-8-23-15(20)13-2-1-5-21-13/h1-5,7,10,17-18H,6,8,16H2/t10-/m0/s1	not tested
6h	1S/C23H21NO7/c24-18(12-15-10-11-19(25)20(26)13-15)23(28)30-14-29-22(27)17-8-4-5-9-21(17)31-16-6-2-1-3-7-16/h1-11,13,18,25-26H,12,14,24H2/t18-/m0/s1	not tested
6i	1S/C23H21NO7/c24-19(12-15-6-11-20(25)21(26)13-15)23(28)30-14-29-22(27)16-7-9-18(10-8-16)31-17-4-2-1-3-5-17/h1-11,13,19,25-26H,12,14,24H2/t19-/m0/s1	not tested
6j	1S/C23H29NO6/c1-14(2)10-16-4-7-18(8-5-16)15(3)22(27)29-13-30-23(28)19(24)11-17-6-9-20(25)21(26)12-17/h4-9,12,14-15,19,25-26H,10-11,13,24H2,1-3H3/t15?,19-/m0/s1	not tested
6k	1S/C21H24N2O7/c1-13(24)23-17(10-14-5-3-2-4-6-14)21(28)30-12-29-20(27)16(22)9-15-7-8-18(25)19(26)11-15/h2-8,11,16-17,25-26H,9-10,12,22H2,1H3,(H,23,24)/t16-,17-/m0/s1	not tested

Table 1 – continued from previous page

Compound	InChI Code	Biological Activity
6l	1S/C28H47NO6/c1-2-3-4-5-6-7-8-9-10-11-12-13-14-15-16-17-27(32)34-22-35-28(33)24(29)20-23-18-19-25(30)26(31)21-23/h18-19,21,24,30-31H,2-17,20,22,29H2,1H3/t24-/m0/s1	not tested
6m	1S/C19H19NO8/c1-11(21)28-17-5-3-2-4-13(17)18(24)26-10-27-19(25)14(20)8-12-6-7-15(22)16(23)9-12/h2-7,9,14,22-23H,8,10,20H2,1H3/t14-/m0/s1	not tested
6n	1S/C21H27NO6/c22-16(6-12-1-2-17(23)18(24)7-12)19(25)27-11-28-20(26)21-8-13-3-14(9-21)5-15(4-13)10-21/h1-2,7,13-16,23-24H,3-6,8-11,22H2/t13-,14-,15?,16+,21?/m1/s1	not tested
6o	1S/C12H15NO6/c1-7(14)18-6-19-12(17)9(13)4-8-2-3-10(15)11(16)5-8/h2-3,5,9,15-16H,4,6,13H2,1H3/t9-/m0/s1	not tested

Table 2: Compound structures (SMILES)

Compound	SMILES
1	OC(C=C1C[C@H](NC(=O)OC(C)(C)C)C(=O)O)=C(O)C=C1
2	O=C(OCC(C=CC1)=CC=1)[C@@H](NC(=O)OC(C)(C)C)CC(C=CC1OCC(C=CC2)=CC=2)=CC=1OCC(C=CC1)=CC=1
3	OC(=O)[C@@H](NC(=O)OC(C)(C)C)CC(C=CC1OCC(=CC=C2)C=C2)=CC=1OCC(=CC=C1)C=C1
4a	O=C(OCCl)[C@H](CC(=CC(OCC(C=CC1)=CC=1)=C1OCC(C=CC2)=CC=2)C=C1)NC(=O)OC(C)(C)C
4b	O=C(OCCl)C(C=CC1)=CC=1
4c	O=C(OCCl)CCCCC(C=CC1)=CC=1
4d	O=C(/C=C/C(=CC=C1)C=C1)OCCl
4e	O=C(OCCl)C(C)(C)C
4f	O=C(OCCl)CCCOC(C=CC1)=CC=1
4g	O=C(OCCl)C(CC)C(=CC=C1)C=C1
4h	O=C(OCCl)C(=CC1)OC=1
4i	O=C(OCCl)C(=CC1)C(=CC=1)OC(C=CC1)=CC=1
4j	O=C(OCCl)C(=CC=C1OC(=CC=C2)C=C2)C=C1
4k	O=C(OCCl)C(C)C(=CC=C1CC(C)C)C=C1
4l	O=C(OCCl)[C@@H](NC(C)=O)CC(=CC=C1)C=C1
4m	O=C(CCCCCCC/C=C\CCCCCCCC)OCCl
4n	O=C(C)OC(C=C1)=C(C=C1)C(=O)OCCl
4o	O=C(OCCl)C(CC(C1)C2)(CC2C2)CC21
5a	O=C(OCOC(=O)C(C=CC1)=CC=1)[C@H](CC(=CC(OCC(=CC=C1)C=C1)=C1OCC(=CC=C2)C=C2)C=C1)NC(=O)OC(C)(C)C
5b	O=C(OCOC(=O)CCCCC(=CC=C1)C=C1)[C@H](CC(C=CC1OCC(=CC=C2)C=C2)=CC=1OCC(=CC=C1)C=C1)NC(=O)OC(C)(C)C
5c	O=C(OCOC(=O)/C=C/C(=CC=C1)C=C1)[C@H](CC(C=CC1OCC(C=CC2)=CC=2)=CC=1OCC(=CC=C1)C=C1)NC(=O)OC(C)(C)C
5d	O=C(OCOC(=O)C(C)(C)C)[C@H](CC(=CC(OCC(C=CC1)=CC=1)=C1OCC(C=CC2)=CC=2)C=C1)NC(=O)OC(C)(C)C
5e	O=C(OCOC(=O)CCCOC(=CC=C1)C=C1)[C@H](CC(C=CC1OCC(C=CC2)=CC=2)=CC=1OCC(C=CC1)=CC=1)NC(=O)OC(C)(C)C
5f	O=C(OCOC(=O)C(CC)C(=CC=C1)C=C1)[C@@H](NC(=O)OC(C)(C)C)CC(=CC(=C1OCC(C=CC2)=CC=2)OCC(=CC=C2)C=C2)C=C1
5g	O=C(OCOC(=O)C(=CC1)OC=1)[C@H](CC(C=CC1OCC(C=CC2)=CC=2)=CC=1OCC(C=CC1)=CC=1)NC(=O)OC(C)(C)C
5h	O=C(OCOC(=O)C(C=CC1)=C(C=1)OC(C=CC1)=CC=1)[C@H](CC(C=CC1OCC(=CC=C2)C=C2)=CC=1OCC(C=CC1)=CC=1)NC(=O)OC(C)(C)C
5i	O=C(OCOC(=O)C(C=CC1OC(C=CC2)=CC=2)=CC=1)[C@@H](NC(=O)OC(C)(C)C)CC(=CC(=C1OCC(C=CC2)=CC=2)OCC(C=CC2)=CC=2)C=C1
5j	O=C(OCOC(=O)C(C)C(C=CC1CC(C)C)=CC=1)[C@@H](NC(=O)OC(C)(C)C)CC(C=CC1OCC(C=CC2)=CC=2)=CC=1OCC(C=CC1)=CC=1

Table 2 – continued from previous page

Compound	SMILES
5k	O=C(OCOC(=O)[C@@H](NC(=O)C)CC(C=CC1)=CC=1)[C@H](CC(=CC(OCC(=CC=C1)C=C1)=C1OCC(=CC=C2)C=C2)C=C1)NC(=O)OC(C)(C)C
5l	O=C(OCOC(=O)CCCCCCC/C=C\CCCCCCCC)[C@@H](NC(=O)OC(C)(C)C)CC(C=CC1OCC(=CC=C2)C=C2)=CC=1OCC(C=CC1)=CC=1
5m	O=C(OCOC(=O)C(C=CC1)=C(C=1)OC(=O)C)[C@H](CC(=CC(OCC(=CC=C1)C=C1)=C1OCC(=CC=C2)C=C2)C=C1)NC(=O)OC(C)(C)C
5n	O=C(OCOC(=O)C(CC(C1)C2)(CC1C1)CC21)[C@H](CC(=CC(=C1OCC(C=CC2)=CC=2)OCC(C=CC2)=CC=2)C=C1)NC(=O)OC(C)(C)C
5o	O=C(OCOC(C)=O)[C@H](CC(=CC(=C1OCC(C=CC2)=CC=2)OCC(=CC=C2)C=C2)C=C1)NC(=O)OC(C)(C)C
6a	OC(C=C(C1)C[C@H](N)C(=O)OCOC(=O)C(C=CC2)=CC=2)=C(O)C=1
6b	OC(C=C(C1)C[C@H](N)C(=O)OCOC(=O)CCCCC(C=CC2)=CC=2)=C(C=1)O
6c	OC(C=C(C1)C[C@H](N)C(=O)OCOC(=O)CCC(C=CC2)=CC=2)=C(O)C=1
6d	OC(C=C1C[C@H](N)C(=O)OCOC(=O)C(C)(C)C)=C(O)C=C1
6e	OC(C=C(C1)C[C@H](N)C(=O)OCOC(=O)CCCOC(=CC=C2)C=C2)=C(O)C=1
6f	OC(C=C(C1)C[C@H](N)C(=O)OCOC(=O)C(CC)C(C=CC2)=CC=2)=C(C=1)O
6g	OC(C=C(C1)C[C@H](N)C(=O)OCOC(=O)C(=CC2)OC=2)=C(O)C=1
6h	OC(C=C1C[C@H](N)C(=O)OCOC(=O)C(C=C2)=C(C=C2)OC(=CC=C2)C=C2)=C(O)C=C1
6i	OC(C=C(C1)C[C@H](N)C(=O)OCOC(=O)C(=CC=C2OC(=CC=C3)C=C3)C=C2)=C(O)C=1
6j	OC(C=C1C[C@H](N)C(=O)OCOC(=O)C(C)C(C=CC2CC(C)C)=CC=2)=C(O)C=C1
6k	OC(C=C(C1)C[C@H](N)C(=O)OCOC(=O)[C@@H](NC(C)=O)CC(=CC=C2)C=C2)=C(C=1)O
6l	OC(C=C1C[C@H](N)C(=O)OCOC(=O)CCCCCCCCCCCCCCCCC)=C(O)C=C1
6m	OC(C=C(C1)C[C@H](N)C(=O)OCOC(=O)C(C=CC2)=C(C=2)OC(C)=O)=C(C=1)O
6n	OC(C=C(C1)C[C@H](N)C(=O)OCOC(=O)C(CC(C2)C3)(CC2C2)CC23)=C(O)C=1
6o	OC(C=C(C1)C[C@H](N)C(=O)OCOC(=O)C)=C(C=1)O
